# The Synthesis and Glycoside Formation of Polyfluorinated Carbohydrates

**DOI:** 10.1021/acs.chemrev.2c00086

**Published:** 2022-05-25

**Authors:** Kler Huonnic, Bruno Linclau

**Affiliations:** † School of Chemistry, University of Southampton, Highfield, Southampton, SO17 1BJ, U.K.; ‡ Department of Organic and Macromolecular Chemistry, Ghent University, Campus Sterre, Krijgslaan 281-S4, Ghent, 9000, Belgium

## Abstract

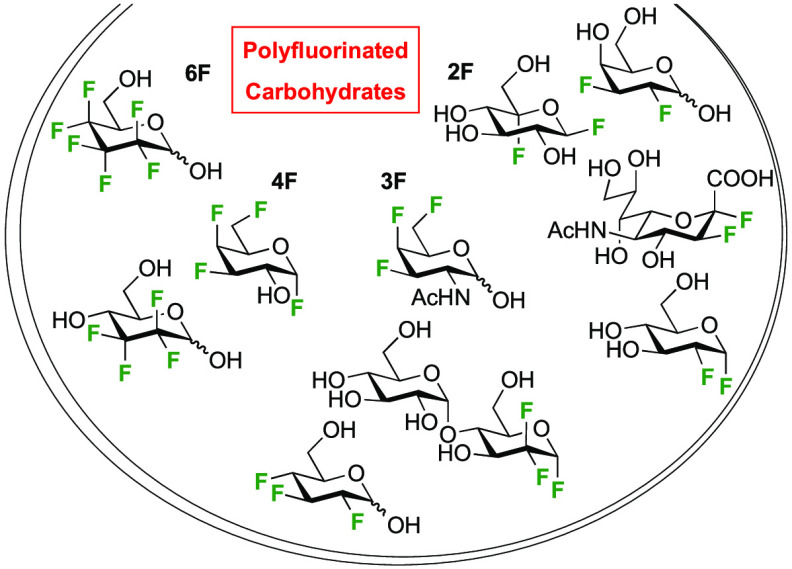

Fluorinated carbohydrates have found many applications in the glycosciences. Typically, these contain fluorination at a single position. There are not many applications involving polyfluorinated carbohydrates, here defined as monosaccharides in which more than one carbon has at least one fluorine substituent directly attached to it, with the notable exception of their use as mechanism-based inhibitors. The increasing attention to carbohydrate physical properties, especially around lipophilicity, has resulted in a surge of interest for this class of compounds. This review covers the considerable body of work toward the synthesis of polyfluorinated hexoses, pentoses, ketosugars, and aminosugars including sialic acids and nucleosides. An overview of the current state of the art of their glycosidation is also provided.

## 1 Introduction

Carbohydrates have essential roles in Nature as energy sources, structural matter, and as molecular recognition elements in cellular processes.^1^ Interactions between carbohydrates and proteins (such as enzymes, lectins, and antibodies) play a role in numerous biological processes related to health, reproduction, and disease, including fertilization, cell–cell interactions, and cell-pathogen interactions.^2−4^ Pathogen-specific glycans are recognized by the immune system, eliciting an immune response.^5−8^ The functioning of the enzymatic machinery responsible for carbohydrate modifications and their glycosidation is crucial to life, with many diseases originating in the malfunctioning of carbohydrate-related processes. Hence, the roles of protein–carbohydrate and carbohydrate–carbohydrate interactions, and how to manipulate them, are therefore intensively investigated.^9−17^

Fluorination of carbohydrates has long been one of the strategies to investigate protein-carbohydrate interactions, for example to investigate the contributions of individual sugar alcohol groups,^18−20^ or in the design of mechanism-based inhibitors.^21,22^ The favorable NMR properties of the ^19^F nucleus have been exploited to investigate protein–carbohydrate binding at the molecular level with ever more sophisticated NMR experiments.^14,23−30^ Fluorination has also been used to investigate intermolecular glycan–glycan hydrogen bonding in carbohydrate materials.^31^

The high hydrophilicity and metabolic susceptibility of carbohydrates generally results in low binding affinities and bioavailabilities, which reduces their application in drug discovery programs. This has led to the development of glycomimetics^32,33^ and the use of multivalent conjugates.^34−37^ However, fluorination of carbohydrates increases their enzymatic and chemical stabilities, and reduces their hydrophilicities, making this modification attractive for drug discovery purposes.^33,38−40^ This extends to applications such as synthetic carbohydrate vaccines.^41−46^

Carbohydrate analogues also have applications in molecular imaging, with ^18^F-2-deoxy-2-fluoroglucose currently being the most widely used PET tracer used for cancer and inflammatory disease diagnosis.^47−49^ The stability of 2-deoxy-2-fluoroglucose imparted by the fluorine atom is a key reason for its success.

In most of the aforementioned applications, monodeoxyfluorinated sugars are involved, including sugars in which a single carbon atom contains two or three fluorine substituents, whether part of a disaccharide/glycan or not. However, dideoxy-difluorinated sugars, notably 2-deoxy-2-fluorinated glycosyl fluorides, 2-deoxy-2,3-difluorinated sialic acids, and 5-fluorinated glycosyl fluorides, have been extensively investigated as mechanism-based glycosylation inhibitors.^21^ Glycoenzyme inhibition data of some of these sugars inspired the “polar hydrophobicity” concept formulated in 1998.^50,51^ This in turn has led to the investigation of the lipophilicity of fluorinated carbohydrates, with the first fluorosugar lipophilicities, obtained by a newly developed and convenient ^19^F NMR based log *P* determination method, reported in 2016.^52^

Nevertheless, the synthesis of polyfluorinated carbohydrates has a long history, with major initial applications being the study of ^19^F NMR spectroscopic properties and sugar conformations.^53−55^ The first dideoxy-difluorinated sugars, 3,5-dideoxy-3,5-difluoro-d-xylose,^56^ and 2-deoxy-2-fluoro-α-d-glucopyranosyl fluoride, and -β-d-mannopyranosyl fluoride,^57^ were synthesized in 1969, while the first trideoxy-trifluorinated sugars, 1,6-di-*O*-acetyl-2,3,4-trideoxy-2,3,4-trifluoro-d-glucose and -galactose, were synthesized in 1989.^58^ The first tetradeoxy-tetrafluorinated sugar, with four hydroxyl groups replaced by fluorine, was reported in 1982,^59^ and the first tetrafluorinated sugar with just two hydroxyl groups replaced, was reported in 2004.^60^ The most heavily fluorinated monosaccharide so far, 2,3,4-trideoxy-2,2,3,3,4,4-hexafluoroglucose, came on the scene in 1998.^50^

This review aims to provide a comprehensive overview of the synthesis of polyfluorinated carbohydrates published in the peer-reviewed literature. Polyfluorinated sugars are defined here as having >1 deoxyfluorination site, resulting in >1 fluorinated carbon atom within a monosaccharide, whether further glycosylated or not. It is organized first by sugar type (aldohexoses, pentoses, ketosugars, and aminosugars, with sialic acids being a separate section), and then by the carbons that are fluorinated. The focus is on the synthetic route(s) to polyfluorinated sugars. Their glycosidation is included as well, but only selective examples are included of other further functionalizations. While the synthesis of polyfluorinated nucleosides is included, nucleoside formation of polyfluorinated sugar donors is not exhaustively covered. Reviews discussing fluorinated nucleosides are available.^61−65^

Where relevant, improvements in synthetic procedures are mentioned, or old/redundant syntheses of precursors or early intermediates are updated. Syntheses of sugars will usually be shown starting from currently available, relatively inexpensive starting materials. This text generally aims to show the full synthesis of each polyfluorinated sugar derivative, but to avoid repetition of synthetic steps leading to common intermediates, the second structure in a synthetic scheme may be an advanced intermediate already discussed elsewhere, with a reference to the relevant scheme. However, it is not within the scope of this contribution to comprehensively review the synthesis of sugar precursors, whether monofluorinated or not. In general, it is aimed to show an efficient route to precursors for which a full experimental data set is available.

6-Deoxy-6-fluorogalactose and 6-fluorofucose have the same structure, as do 6-deoxymannose and rhamnose. These will be regarded as galactose/mannose analogues when in the d-configuration, and as fucose/rhamnose analogues when in the l-configuration, given that their synthesis can be very different.

Additional deoxygenation is not considered, with the exception for deoxygenation at the 6-position. Iminosugars, inositols, carbasugars, and C-glycosides are not included.

Several reviews covering the synthesis of fluorinated carbohydrates (mostly monofluorinated or geminal difluorinated), have been published within the last 10 years.^66−69^ A number of older reviews are also available,^70−82^ including discussion of the material’s NMR properties.^54,55^ While many reviews do include aspects of polyfluorinated carbohydrates, this review aims to provide an updated comprehensive overview of their synthesis.

## 2 Short Overview of Fluorinating Agents

In this section, the fluorination agents that feature in this review are briefly introduced.

### 2.1 Electrophilic Fluorination Agents

**Figure 1:**
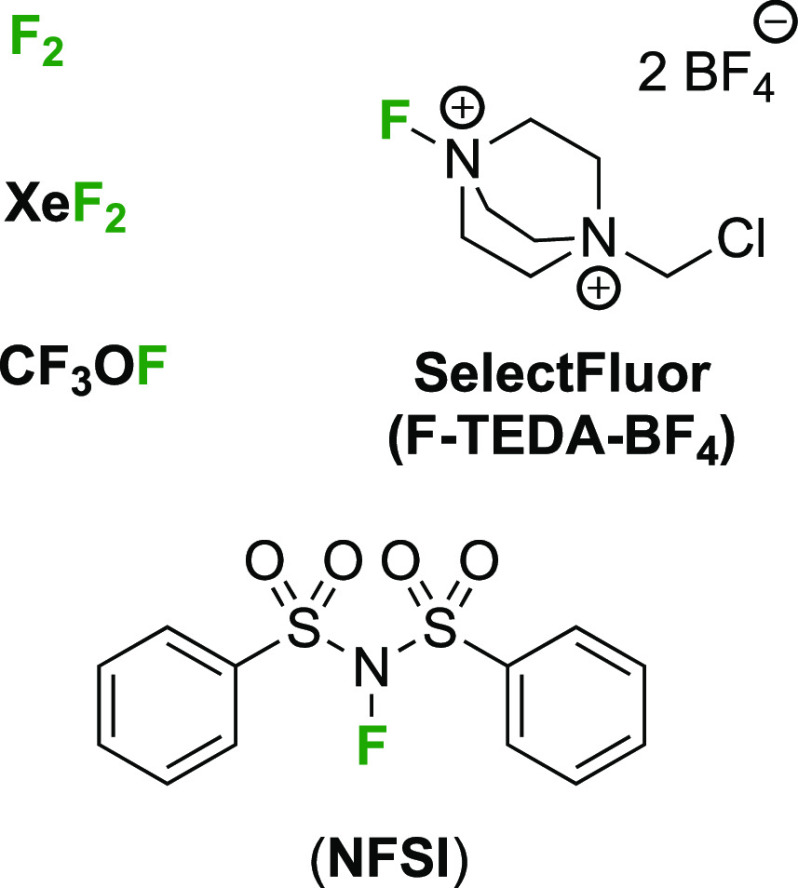
Electrophilic fluorinating agents featuring in this review.

The electrophilic fluorinating agents featuring in this review are listed in Figure 1. Fluorine (**F**_**2**_) is a highly reactive and toxic gas, usually used diluted with an inert gas (N_2_, He), and nowadays only employed by specialized laboratories. Trifluoromethyl hypofluorite (**CF**_**3**_**OF**), the reaction product of carbon monoxide with F_2_, is a highly toxic gas. Both are sources of electrophilic fluorine, and in carbohydrate chemistry have been mainly used for the reaction with glycals before the invention of more convenient electrophilic fluorinating agents. In contrast, xenon difluoride (**XeF**_**2**_), prepared from xenon and fluorine, is a solid. It is a milder fluorinating agent and although still very reactive, it requires use under an inert atmosphere.^83^ The development of N–F based electrophilic fluorination agents by a number of groups in the mid-1980s completely transformed the area of electrophilic fluorination,^84^ with 1-chloromethyl-4-fluoro-1,4-diazoniabicyclo[2.2.2]octane bis(tetrafluoroborate) (**SelectFluor**, also abbreviated as F-TEDA-BF_4_), developed by the Banks group,^85,86^ also used extensively in in carbohydrate chemistry.^87^ It is synthesized via reaction of F_2_ and DABCO. A milder electrophilic fluorination agent is *N*-fluorobenzenesulfonimide (**NFSI**), which was introduced in 1991 by the Differding group at Ciba-Geigy.^88,89^ This reagent is also derived from F_2_, by reaction with benzenesulfonimide, and is a crystalline powder. Relative reactivity scales that also include other electrophilic fluorination reagents have been determined.^90−92^

### 2.2 Nucleophilic Fluorination Agents

**Figure 2:**
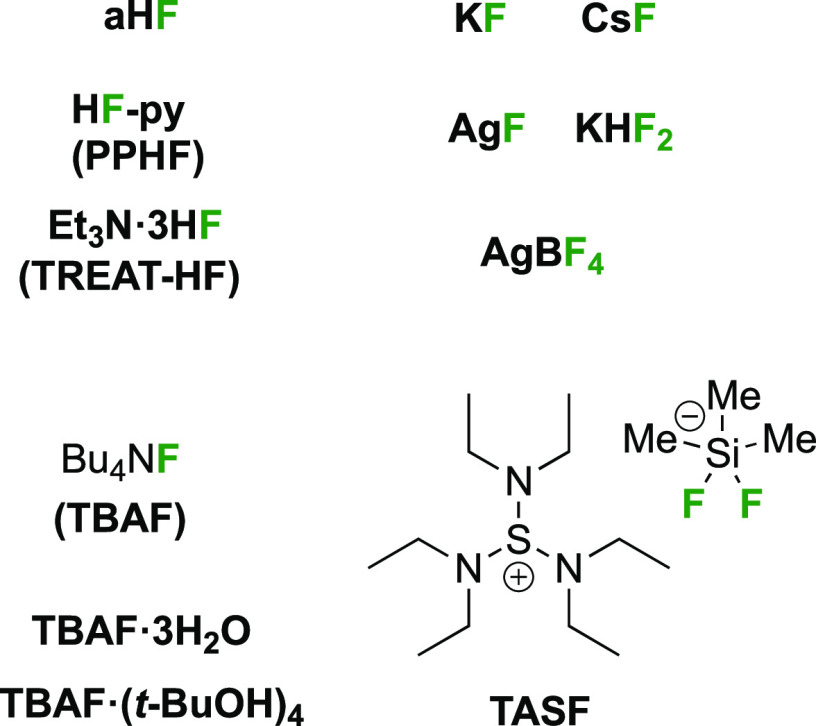
Nucleophilic fluorination reagents featuring in this review.

The nucleophilic fluorinating agents featuring in this review are listed in Figure 2. Anhydrous HF (**aHF**) is the primary source for all fluorination reagents, being obtained by sulfuric acid treatment of fluorospar (CaF_2_), sourced from mining operations.^93^ It is thus very inexpensive; however, it is extremely toxic, difficult to use, and etches glassware. It is nowadays mostly used in combination with an organic base.^94^ Anhydrous HF has seen use in carbohydrate chemistry for the synthesis of glycosyl fluorides, until a convenient method was developed using the milder Olah’s reagent by the Noyori and the Szarek groups.^95,96^ Olah’s reagent (pyridinium poly(hydrogen fluoride), **HF-py**, also abbreviated as PPHF) is a mixture of 70% HF in pyridine (py),^97^ equivalent to a 9:1 HF/py molar ratio, and is widely used in organic chemistry as a nucleophilic fluorinating agent.^94,98^ Another HF-derived reagent is Et_3_N·3HF, triethyl amine trishydrofluoride (**TREAT-HF**).^99^ While it has seen much use as fluorination agent through nucleophilic substitution reactions with activated alcohols and epoxides, as well as for halofluorination reactions, it has limited nucleophilicity.^94,100^ The addition of Et_3_N causes equilibration to Et_3_N·2HF and Et_3_N·HF, which were shown to lead to more nucleophilic reagents.^101−103^ It is worth adding that a combination of HF with the nonbasic 1,3-dimethyl- 3,4,5,6-tetrahydro-2(1*H*)-pyrimidinone (DMPU), leading to the hydrogen-bonded complex HF·DMPU, has been developed as a useful HF-derived reagent by the Hammond group.^104^

Alkali metal fluorides such as potassium and cesium fluoride (**KF**, **CsF**) have been widely used as inexpensive fluorinating agents, despite their limited solubility in organic media. It has been found that the use of bulky alcohols as solvents, such as *t*-BuOH, is beneficial for fluorinations with these reagents, including in carbohydrate applications.^105^ This has been attributed to stabilization of the fluoride anion by hydrogen bonding to provide a “controlled” environment balancing fluoride basicity and nucleophilicity. While tetraalkylammonium fluorides such as Bu_4_NF have enhanced solubility in organic solvents, its fluoride reactivity is also beneficially modified by using *t*-BuOH as solvent. The Kim group developed TBAF(*t*-BuOH)_4_ as an isolable reagent with excellent fluorination properties,^106,107^ and the Gouverneur group has developed other types of hydrogen-bonded fluoride reagents.^108−110^

The combination of HF and KF, leading to potassium hydrogen difluoride (**KHF**_**2**_), a reagent first used in fluorosugar synthesis,^111^ has proven to be a useful reagent for epoxide opening, although elevated temperatures are typically required.^112^ Silver fluoride (**AgF**) is soluble in acetonitrile and DMF,^113^ and has also seen use for glycosyl fluoride synthesis from glycosyl bromides or chlorides.^76^ Silver tetrafluoroborate (**AgBF**_**4**_) is soluble in water and many organic solvents, and is involved in a wide variety of transformations.^114^ Silver-based reagents use the precipitation of silver salts (such as silver bromide) as the driving force when used in halogen displacement reactions.

Finally, tris(dimethylamino)sulfonium difluorotrimethylsilicate (**TASF**) is a mild fluoride donor shown by the Szarek group to efficiently displace triflates in fluorosugar synthesis,^115^ although elimination products were observed in some cases. This reagent is also a useful alternative to the more basic TBAF for silyl ether cleavage.^116,117^

Nucleophilic fluorination by displacement of sulfonates is typically an S_N_2 reaction, with steric hindrance (often by protecting groups) an important consideration for the success of the reaction. In addition, the transition state of this reaction features two polar bonds, and in a carbohydrate context, the interaction between the resulting dipoles with those of C–O bonds in adjacent positions is also an important factor. This had been recognized early on in carbohydrate synthesis, with the formulation of the Richardson-Hough rules^118^ (recently updated by the Hale group to include triflate displacements,^119^ including for furanoses^120^). A summary is included in a recent review dealing more generally with controlled inversion strategies in carbohydrate synthesis.^121^ These rules are important to consider, given fluoride is a weak nucleophile, and its basicity can facilitate elimination side reactions.

The nucleophilic opening of epoxides with fluoride is a widely used process to synthesize fluorosugars. Regioselectivity is generally determined by the possibility to proceed via a chairlike transition state (the so-called Fürst-Plattner effect),^122,123^ which has proven to be especially useful with opening of epoxides within 1,6-anhydrosugars. With more conformationally flexible substrates, steric hindrance and the electron withdrawing effect of the anomeric center are typical factors determining regioselectivity.

### 2.3 Deoxyfluorination Agents

**Figure 3:**
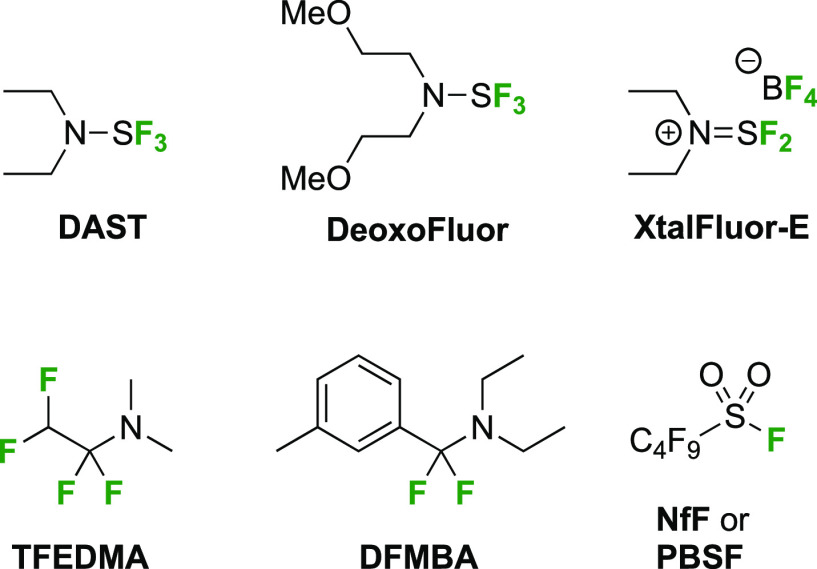
Deoxyfluorination reagents featuring in this review.

Deoxyfluorination reagents (Figure 3) represent a class of nucleophilic fluorination reagents that are also able to activate an alcohol into a leaving group, and by doing so, release fluoride which can then act as the nucleophile to effectively cause a “deoxyfluorination” reaction. Some reagents are able to convert a carbonyl group to a CF_2_-moiety, in which case the term ‘deoxofluorination’ is used.

Sulfur tetrafluoride (SF_4_) was the original (and very effective) deoxyfluorination agent, but its very high toxicity and gaseous nature prohibit its use in research laboratories. A number of SF_4_ derivatives have been developed, of which diethylaminosulfur trifluoride (**DAST**, Figure 3) has been the most important. It is fair to say that the availability of DAST has been a key turning point in the development of organofluorine chemistry. It was developed by Middleton in 1975,^124^ and to the best of our knowledge, was already first applied two years later for the synthesis of fluorinated sugars by the Korytnyk group (6-deoxy-6-fluoroglucose).^125^ However, DAST has the potential to decompose violently when heated above 80 °C,^126^ and in 1999, di(2-methoxyethyl)aminosulfur trifluoride (**DeoxoFluor**) was introduced by the Lal group as a broad-spectrum deoxyfluorination agent with enhanced thermal stability, and very similar reactivity as DAST.^127,128^

Both DAST and DeoxoFluor are liquids which slowly decompose over time. Stable, crystalline derivatives such as diethylaminodifluorosulfinium tetrafluoroborate (**XtalFluor-E**, pronounced “crystalfluor”), essentially the product of reaction from DAST with BF_3_, have been introduced by the Couturier team at OmegaChem.^129−131^ Because its reactive fluoride is now sequestered by BF_3_, a promoter such as Et_3_N·3HF or 1,8-diazabicyclo[5.4.0]undec-7-ene (DBU) is required for the reaction.

Nonsulfur based deoxyfluorination agents include 1,1,2,2-tetrafluoroethyl-*N*,*N*-diethylamine (**TFEDMA**),^132^ which is also volatile, and *N*,*N*-diethyl-α,α-difluoro(*m*-methylbenzyl)amine (**DFMBA**, Figure 3),^133,134^ which was shown to have a high thermal stability. Nonafluorosulfonyl fluoride (**NfF**, also known as perfluorobutylsulfonate **PBSF**) is another deoxyfluorinating agent, with which alcohol activation and fluoride displacement occur in the same reaction mixture. Additives are required for efficient reaction, such as hindered or non-nucleophilic bases,^135,136^ Et_3_N·3HF/Et_3_N,^103^ and tetrabutylammonium triphenyldifluorosilicate.^137^

**Scheme 1:**
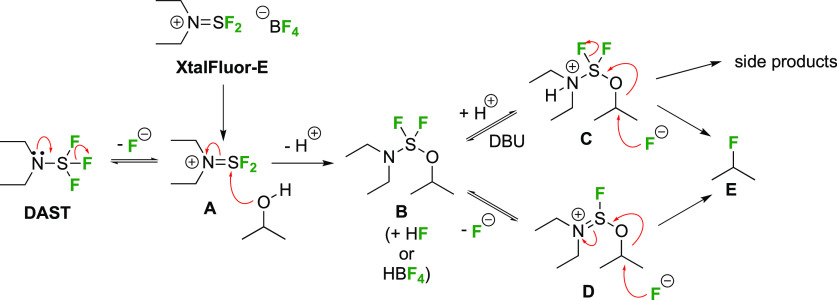
Possible Mechanism for DAST/DeoxoFluor and XtalFluor-Mediated Deoxyfluorination of Alcohols

Given the extensive use of DAST in fluorosugar chemistry, with its reactive nature in some cases leading to rearrangement reactions, it is worth discussing mechanistic aspects of this reaction. A possible deoxyfluorination mechanism is shown in Scheme 1. The availability of a pair of electrons on the nitrogen atom allows fluoride elimination to intermediate **A**, which essentially is the reactive part of XtalFluor-E. This is intercepted by an alcohol, which leads to **B** upon proton loss. Such intermediates have been isolated,^138^ and invoked as leaving groups in a subsequent nucleophilic substitution with fluoride,^124,139^ but the presence of HF could lead to protonation to give **C**, which could undergo substitution with fluoride. Alternatively, **B** could lose another fluoride to give **D**, which can then undergo nucleophilic substitution with fluoride. In many DAST reactions, an amine is added as additive, which would act as proton scavenger to promote formation of **D**. When XtalFluor-E is used, there is no free fluoride to scavenge the proton released in the formation of **B**, essentially leading to the formation of the strong acid HBF_4_, leading to **C**.^130^ Interestingly, this intermediate **C** was suggested not to be a good electrophile for reaction with fluoride, leading to side reactions, including reaction with another equivalent of alcohol to form symmetrical ethers, and loss of diethyl amine, ultimately leading to the formation of symmetrical sulfites (not shown).^130^ This can be mitigated by adding an external fluoride source, such as Et_3_N·3HF, or also by adding a base such as DBU, preventing the formation of **C**. Hence, formation of intermediate **D** with release of another fluoride is promoted, which can then undergo reaction with the released fluoride to give **E**.^130,140^

In DAST-mediated reactions a competition between S_N_2 and S_N_1 processes is often observed, certainly in the presence of structural factors that stabilize carbenium intermediates. In carbohydrates, apart from the anomeric position, an S_N_2 process with inversion of configuration is typically observed, hence the aforementioned Richardson-Hough rules also apply. However, the strong electrophilic nature of the intermediates and the rigidity imparted by the sugar ring frequently cause elimination and rearrangement processes, which will be illustrated throughout the review.^81,141−146^

Finally, it is worth mentioning that there are many other deoxyfluorination agents available that do not feature in this review.^140,147−149^

## 3 Aldohexoses: Fluorination at Two Positions

### 3.1 Fluorination at Positions 1 and 2

#### 3.1.1 Difluorinated at Positions 1 and 2

There have been many reports describing the synthesis of 1,2-difluorinated sugars, either as synthetic intermediates for 2-fluorinated sugar derivatives, or as desired substrates for enzyme or NMR studies. Only those reports that describe the isolation of the 1,2-difluorinated sugars will be detailed here and will be discussed for each type of sugar according to their fluorination method.

##### 3.1.1.1 1,2-Difluorinated Glucose/Mannose Derivatives

**Scheme 2:**
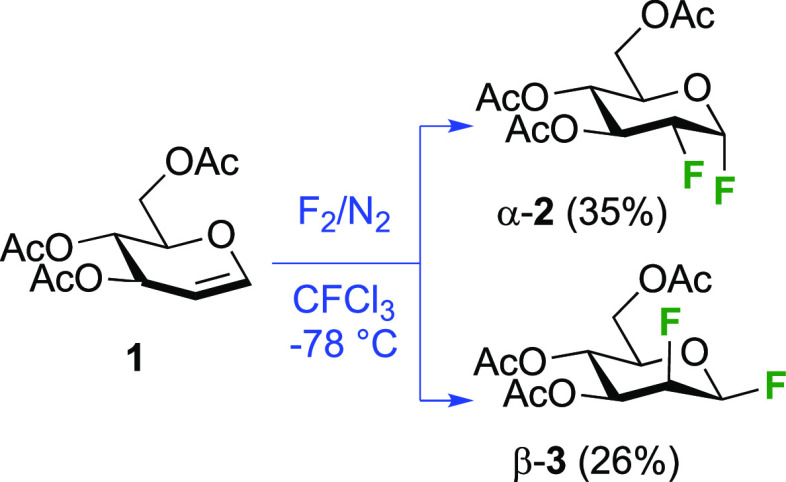
Synthesis of 1,2-Difluorinated Sugars by *syn*-Fluorine Addition^150^

The early work regarding the synthesis of 2-deoxy-2-fluoroglucosyl fluoride mainly centered around the development of effective syntheses of 2-deoxy-2-fluoroglucose, of which it is a possible precursor. Initial approaches involved the reaction of glucals with fluorine and CF_3_OF. The reaction of commercially available tri-*O*-acetyl-d-glucal **1** with F_2_ to give 1,2-difluorinated glucose and mannose derivatives has been described by the Fowler group (Scheme 2).^150^ Fluorine reacts in a *syn*-addition fashion with a slight preference from the α-face to give the *gluco*-compound **2** as the major isomer. These compounds are stable to chromatography and could be separated. The Satyamurthy group has investigated the solvent-dependency of the facial selectivity of the fluorine addition to **1**, and found that apolar solvents lead to a greater α-**2**/β-**3** ratio (as measured by the ratio of their hydrolysis products 2-deoxy-2-fluoroglucose and -mannose).^151^ The Schrobilgen group reported that when **1** was reacted with F_2_ in anhydrous HF, no 2-fluorinated glucose or mannose products were formed, but that after hydrolysis of the 1,2-difluorinated fluorination product, 2-deoxy-2-fluoroallose was obtained (not shown).^152^ This was explained by protonation of the C-3 OAc group, which then resulted in a cyclization with the 4OAc group, which after hydrolysis resulted in inversion of configuration at C-3. No 2-fluorinated allosyl fluoride was isolated however.

**Scheme 3:**
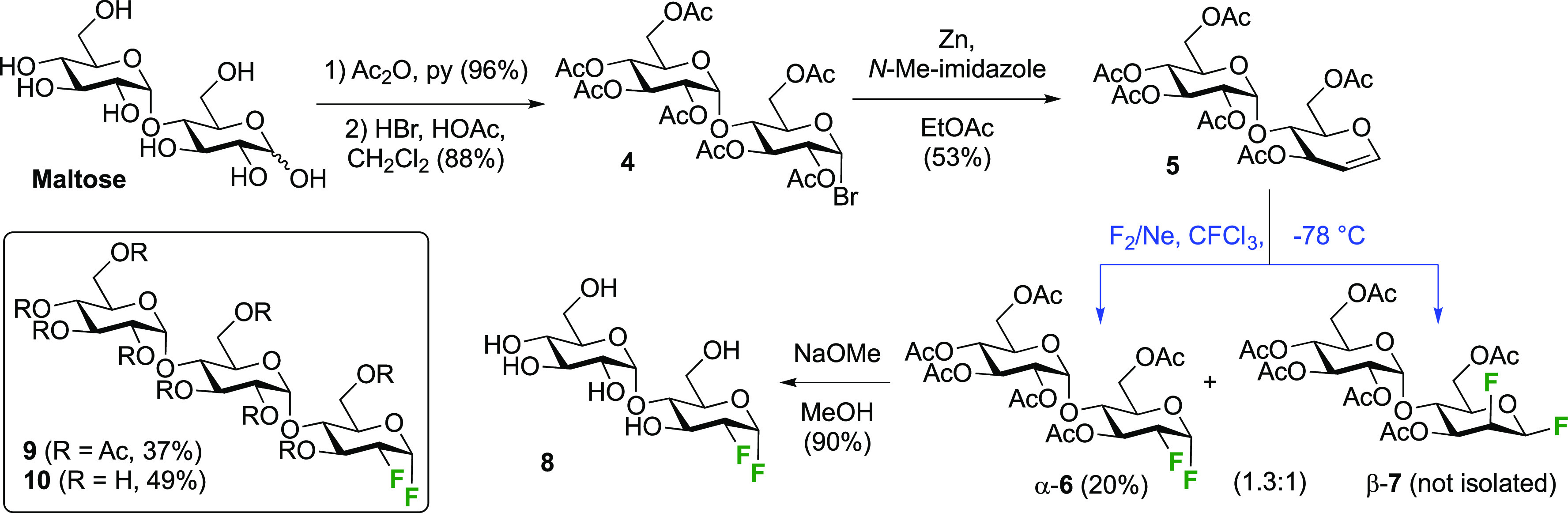
Application of *syn*-Fluorination to 1,2-Difluorinated Di- and Tri-saccharides^153^

The Withers group used this procedure to synthesize 2-deoxy-2-fluoromaltosyl fluoride and -maltotriosyl fluoride as inactivators of α-glycosidase enzymes (Scheme 3).^153^ The required peracetylated maltal **5** can be obtained in three standard steps from maltose involving peracetylation, conversion of the anomeric acetate into the anomeric bromide **4**, and Zn-mediated elimination of the C-2 OAc group.^154−156^ The addition of fluorine proceeded with a decreased facial selectivity (α-**6**/β-**7** 1.3:1) compared to triacetyl glucal, ascribed to the presence of the (1 → 4)-linked glucose tetraacetate. Chromatographic separation afforded **6** in 20% yield, which was successfully deprotected to give **8**. In a similar way, the maltotriose derivative **9** was isolated in 37% yield, and then deprotected to give **10**.

**Scheme 4:**
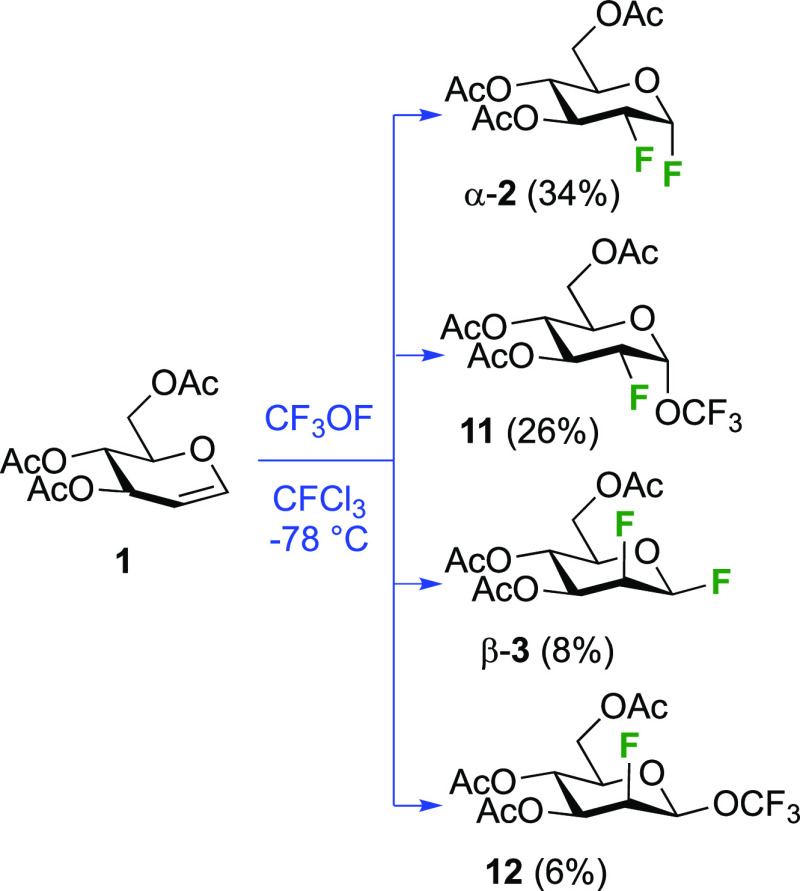
Synthesis of 1,2-Difluorinated Sugars by *syn*-CF_3_OF Addition^57,157^

The reaction of triacetyl-d-glucal with CF_3_OF was investigated by the Foster group and was found to lead to a mixture of four separable compounds (Scheme 4).^57,157^ The 2-deoxy-2-fluorinated glucosyl and mannosyl fluorides α-**2** and β-**3** are formed, alongside trifluoromethoxylated byproducts **11** and **12**. The reaction also proceeds via *syn*-addition, with increased facial selectivity toward the *gluco*-compounds compared to F_2_.^151^ The glycosyl fluorides and trifluoromethyl glycosides can be hydrolyzed in strong acid, for example, α-**2** and **11** were converted into 2-deoxy-2-fluoroglucose in 85 and 91% yield, respectively, and **12** to 2-deoxy-2-fluoromannose in 67% yield (not shown).^57^

**Scheme 5:**
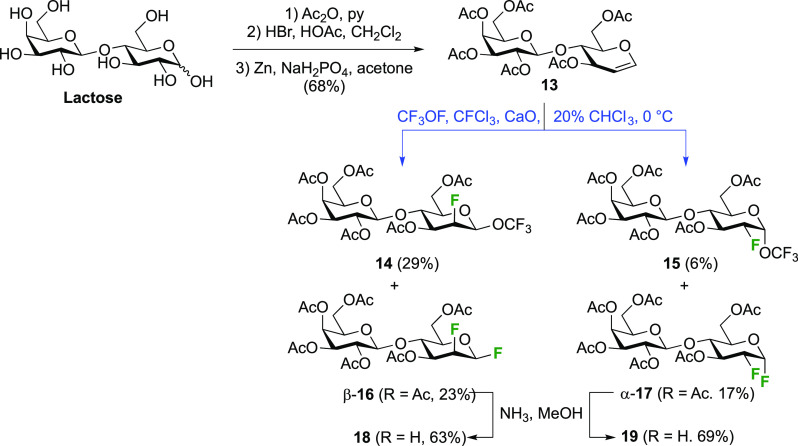
Application of *syn*-Addition of CF_3_OF to d-Lactal^158^

The Kent group employed this procedure with peracetylated lactal **13** (Scheme 5).^158^ Many syntheses of lactal are available, for example via peracetylation of lactose, anomeric bromination, and elimination with Zn.^159^ Compared to a reaction with peracetylated glucal, reaction of **13** with CF_3_OF was reported to require a higher temperature, and proceeded with a different facial selectivity. The *syn*-addition products arising from the β-face approach, the 2-epilactosyl products **14** and **16**, are now the major isolated isomers, which was explained by the steric influence of the second monosaccharide ring. These observations are consistent with the observed stereoselectivity difference between the reaction of peracetylated glucal and maltal with F_2_ (cf. Schemes 2 and 3). The difluorinated compounds **16** and **17** were then deprotected to give 2-deoxy-2-fluoro-β-epilactosyl fluoride and -α-lactosyl fluoride **18** and **19**.

**Scheme 6:**
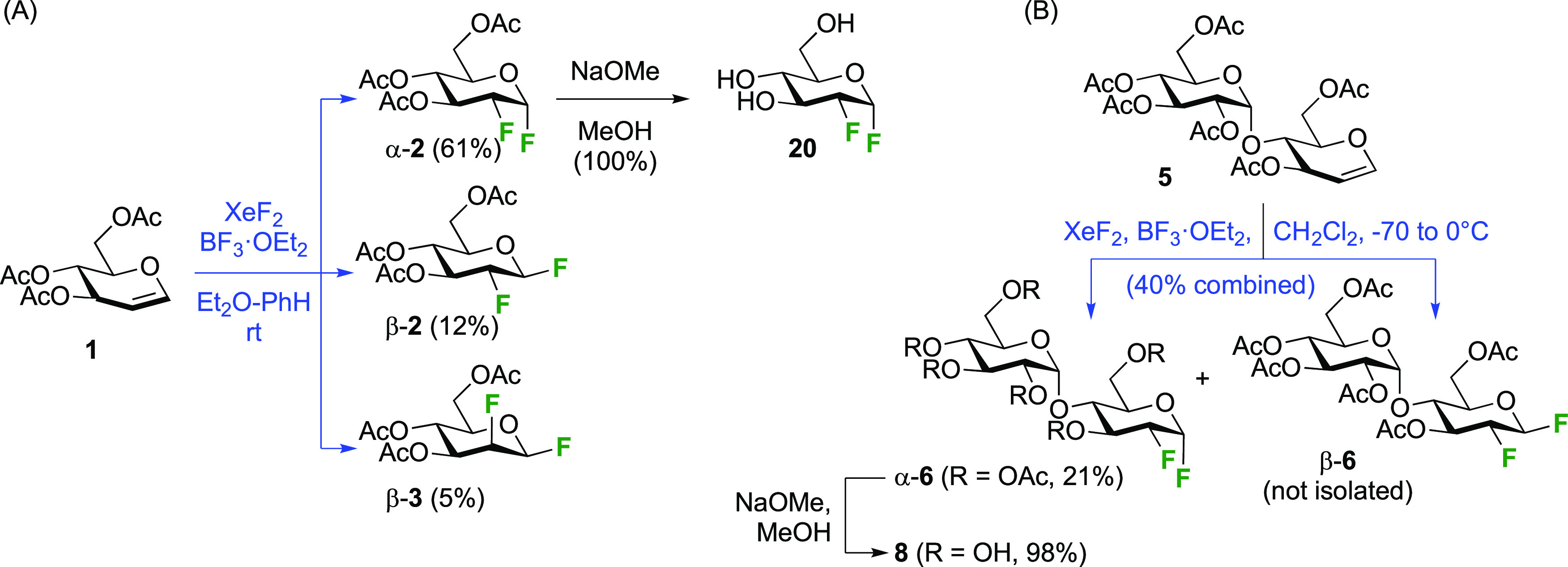
Synthesis of 1,2-Difluorinated Sugars by XeF_2_ Addition^160−163^

The Korytnyk group investigated the use of xenon difluoride as alternatives for F_2_ and CF_3_OF (Scheme 6A).^160,161^ Three products were obtained, with acetylated 2-deoxy-2-fluoroglucosyl fluoride α-**2** as the major isomer. Its β-anomer β-**2** and the β-anomer of the corresponding mannosyl product β-**3** were isolated in small amounts. The Quayle group obtained a total yield of 91% for this reaction, with less than 10% of β-**2** and β-**3** combined.^162^ A benzene–ether solvent mixture was found to be optimal, with the use of ether alone leading to a very slow reaction. To avoid BF_3_-catalyzed Ferrier-type rearrangements, this reagent had to be added slowly to the reaction mixture. The presence of β-**2** was shown not to arise from BF_3_-catalyzed anomerization, which indicates that the XeF_2_ reaction is not a concerted *syn*-addition process.^161^

The Bornemann group applied this process in the synthesis of the 1,2-difluorinated maltose **8** (Scheme 6B), but in dichloromethane as solvent.^163^ The formation of both anomers of 2-deoxy-2-fluoromaltyl fluoride was reported in a 40% combined yield. After separation, α-**6** was deprotected to give **8**, which was used for enzyme studies.

**Scheme 7:**
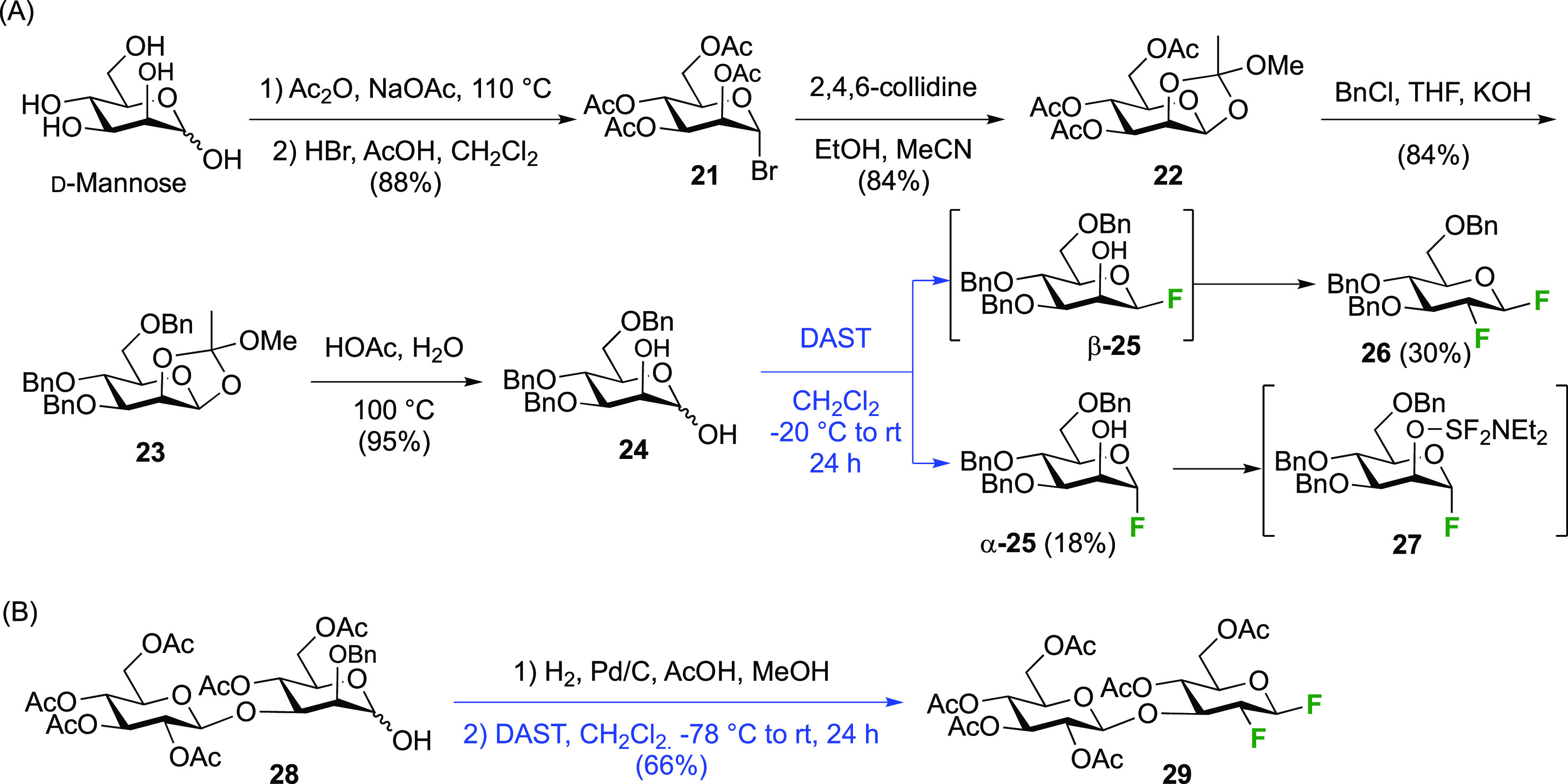
Direct Conversion of 2-Deprotected Mannopyranose with DAST to 2-Deoxy-2-fluoro-β-glucosyl Fluoride Derivatives^143,167^

The Withers group reported a direct conversion of the mannose derivative **24** using DAST (Scheme 7A).^143^ This intermediate was synthesized in five steps from mannose, first by obtaining the peracetylated α-mannosyl bromide **21**, then by *ortho*-ester formation **22**, and after a protecting group switch to **23**, hydrolysis of the *ortho*-ester. Treatment of **24** with DAST leads to the 2-deoxy-2-fluorinated β-glucosyl fluoride derivative **26** in 30% isolated yield, with the monofluorinated α-mannosyl fluoride α-**25** as the other isolated product. This reaction outcome was explained by initial conversion of **A** to both anomeric mannosyl fluorides β-**25** and α-**25**, but with the second deoxyfluorination process only proceeding for the β-anomer β-**25**, due to the—commonly observed—reluctance of α-configured mannose derivatives to undergo nucleophilic substitution at C-2. In the case of α-**25**, similar to the Richardson-Hough rules,^118,119^ and as established more generally for S_N_2 reactions adjacent to fluorine,^164−166^ the strong C–F dipole was thought to cause unfavorable dipole interactions with the transition state of the S_N_2 reaction at C-2. Hence, activated intermediate **27**, which would be formed by reaction of α-**25** with DAST, does not react and is hydrolyzed in the workup to give back α-**25**.

The Stick group applied this dideoxy-difluorination reaction with the disaccharide **28** (Scheme 7B) after benzyl hydrogenolysis, to give the 2-fluorinated β-laminaribiosyl fluoride **29** as the only isolated product in 66% yield.^167^

**Scheme 8:**
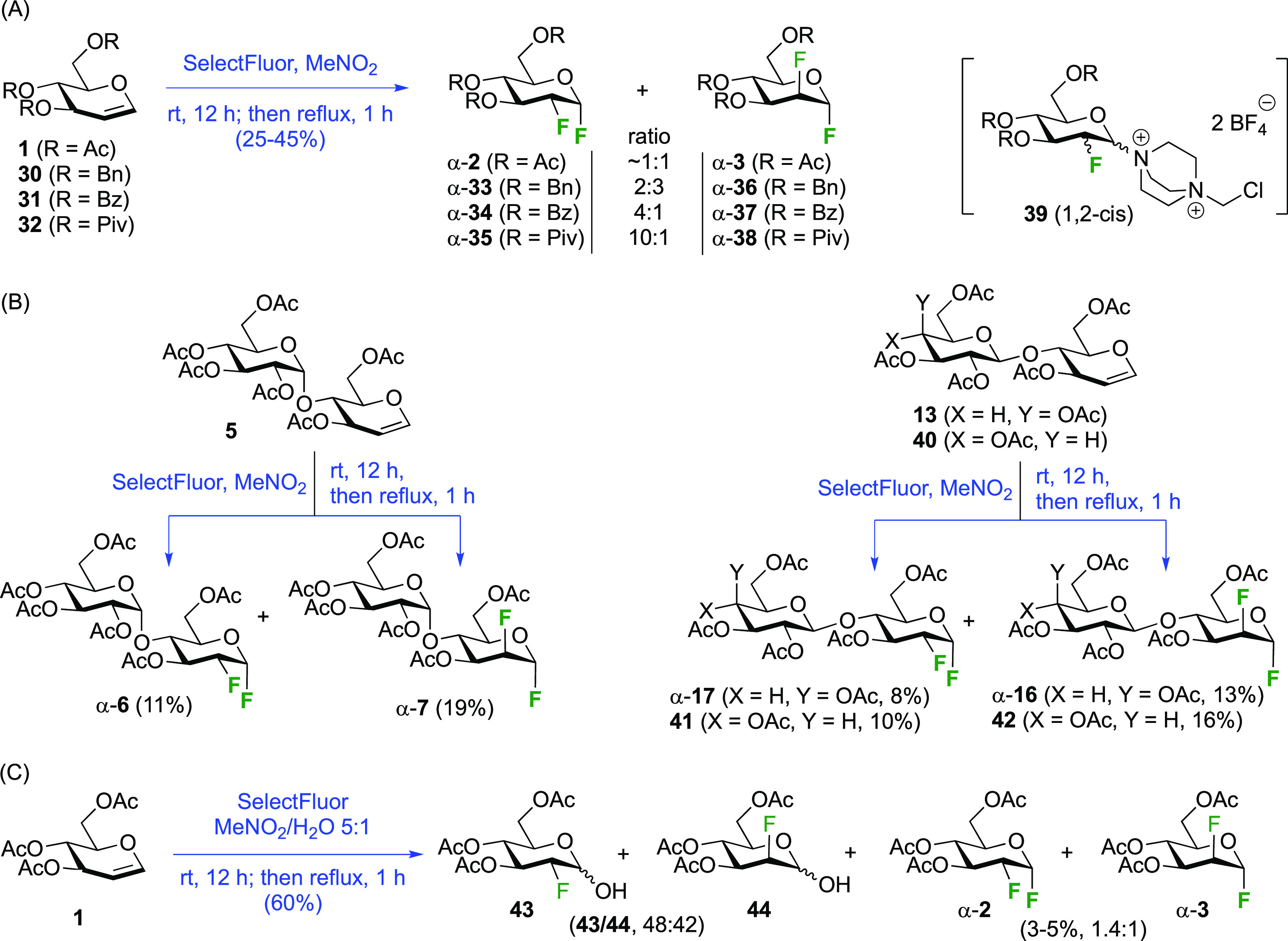
Synthesis of 1,2-*gluco*/*manno* Difluorides Using SelectFluor^168^

The Dax group found that reactions of glycals with SelectFluor in the absence of water led directly to 2-fluorinated glycosyl fluoride derivatives (Scheme 8A).^168^ This reaction proceeds via *syn*-addition of SelectFluor to give adducts **39**, which can then react with nucleophiles to effect substitution at the anomeric center,^168^ including glycosidations.^169^ In the absence of other nucleophiles, the tetrafluoroborate counterions in SelectFluor can act as fluoride donors. As such, glycals **1**,**30**–**32** gave mixtures of inseparable 2-deoxy-2-fluoro-α-glycosyl- and -α-mannosyl fluorides **33**–**35** and **36**–**38** in yields between 25 and 45%, and in ratio’s depending on the nature of the protecting groups.^168^ When starting from the glycal derivatives of maltose, lactose, and cellobiose **5**,**13**, and **40** (Scheme 8B),^168^ the resulting C-2-epimers were separable, with the 2-deoxy-2-fluoro-α-maltosyl-, -lactosyl-, and -cellobiosyl fluorides **6**, **17**, and **41** obtained in a lower yield compared to their 2-epi derivatives **7**, **16**, and **42**. When the SelectFluor reaction is carried out in a nitromethane (or DMF)–water mixture, as illustrated with the commercially available tri-*O*-acetyl-d-glucal **1** (Scheme 8C), then the hemiacetals **43**/**44** are obtained.^168,170^ In this case, water acts as nucleophile to react with **39**. The Priebe group reported this reaction on a large scale, in which α-**2** and α-**3** were still found to be minor products in the reaction mixture.^171^

**Scheme 9:**
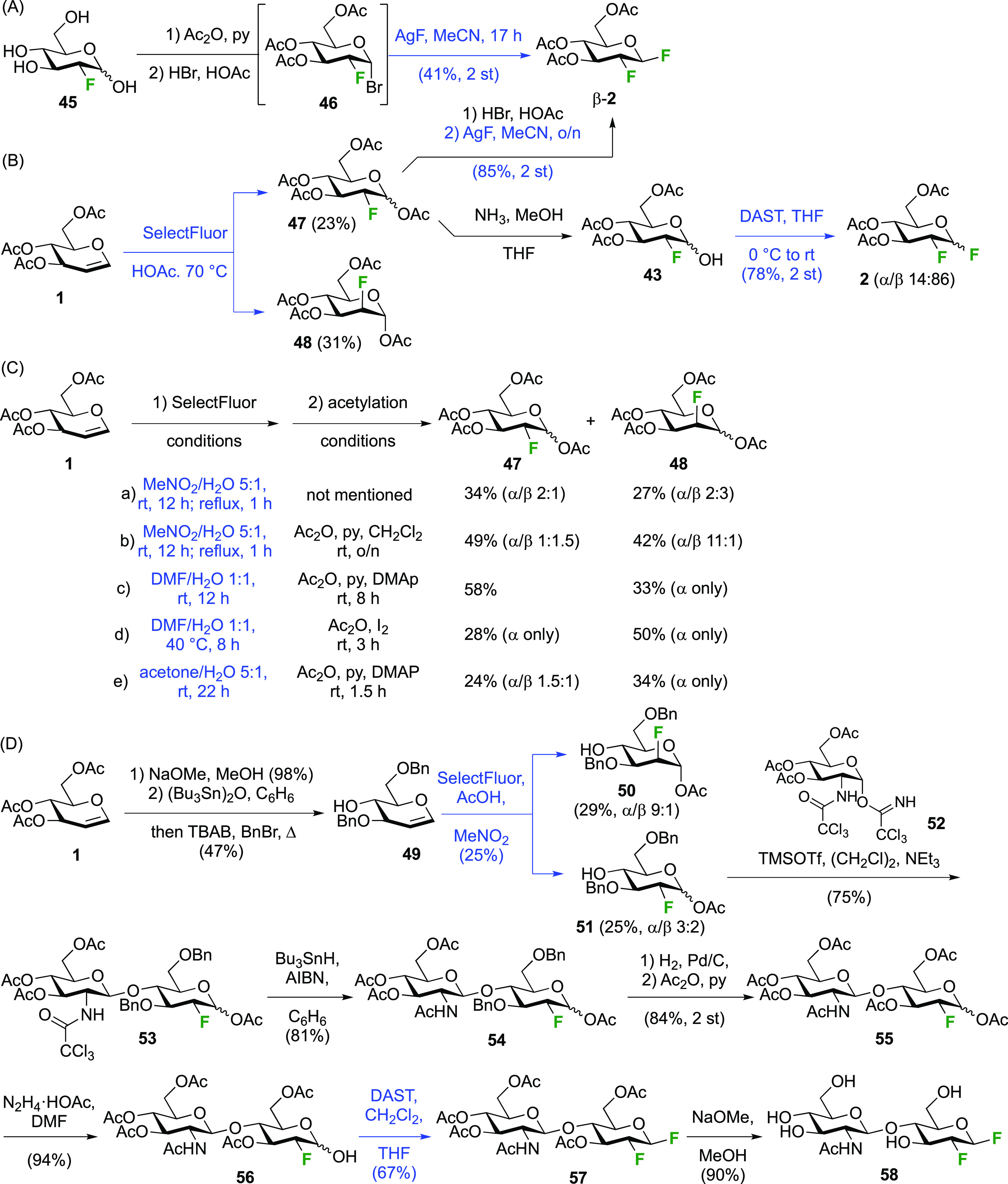
Sequential Syntheses of 2-Deoxy-2-fluoro Glucosyl Fluoride^172,174,175,179^

In addition to the direct difluorination methods described above, sequential methods have also been employed. Starting from 2-deoxy-2-fluoroglucose **45** (Scheme 9A), obtained as mentioned with Scheme 4 from the hydrolysis of **2**/**11**, the Foster group synthesized the glycosyl bromide **46** via the peracetate, which was subjected to AgF to afford β-**2** with the β-anomer as the only reported product.^172,173^ The Withers group used this procedure as well,^174^ with the peracetate intermediate **47** (Scheme 9B) obtained from tri-*O*-acetyl-d-glucal via treatment with SelectFluor in acetic acid (as opposed to nitromethane or water as shown in Scheme 8). This directly afforded a mixture of separable acetates **47** and **48**. From **47**, anomeric bromide formation and fluoride displacement led to 14 in 85% yield.^174^ Alternatively, DAST-mediated deoxyfluorination with 3,4,6-tri-*O*-acetyl-2-deoxy-2-fluoroglucose **43** was investigated as well.^175^ It was synthesized from the mixture of 2-fluoroglucose anomers **47** by treatment with methanolic ammonia to effect anomeric deprotection. Treatment with DAST then gave **2**, but as a mixture of anomers.^175^

It should be said that the reaction of tri-*O*-acetyl-d-glucal with SelectFluor in aqueous medium is currently the most employed method to obtain **47**, despite a separate acetylation step being required to separate the *gluco*- and *manno* isomers, often as a mixture of anomers.^168^ Given the importance of this method, some typical results are summarized in Scheme 9C. The *gluco*/*manno* ratio, as well as the ratio of their respective anomers, varies according to the reaction conditions, although it should be noted that typically isolated yields are reported (as opposed to analyses on crude reaction mixtures). With aqueous nitromethane as solvent (a,b), the *gluco*-product is the major isomer.^168,171^ In aqueous DMF at room temperature,^176^ a high *gluco*/*manno* ratio was obtained. However, when the reaction was carried out at 40 °C, the opposite result was reported.^177^ The reaction in aqueous acetone also delivered the *manno*-product as major isomer, albeit in a lower ratio.^175,178^

Finally, the Withers group applied the sequential SelectFluor/DAST difluoride introduction in the synthesis of the disaccharide **58** (Scheme 9D).^179^ Tri-*O*-acetyl-d-glucal **1** was first converted to the 3,6-di-*O*-benzylated glucal **49**,^180^ to which SelectFluor addition in nitromethane with acetic acid as nucleophile led to the separable **50** and **51** in, respectively, 29% and 25% yield.^179^ Glycosylation of **51** with donor **52** gave disaccharide **53**, after which the trichloroacetamide group was reduced to the acetamide **54**. Benzyl hydrogenolysis and reprotection of the uncovered alcohols as acetate gave **55**, which was then selectively deprotected at the anomeric position. DAST-mediated deoxyfluorination afforded **57** as the only reported anomer. Finally, global deprotection gave **58**.

**Scheme 10:**
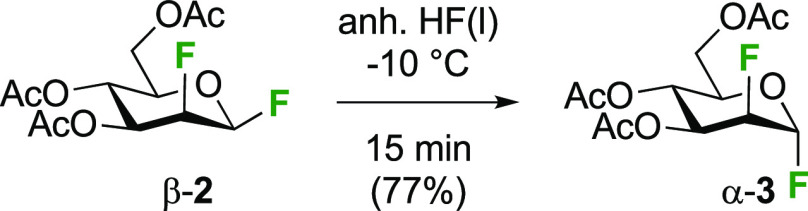
Anomerization to Obtain Pure Tri-*O*-acetyl-2-deoxy-2-fluoromanno-α-pyranosyl Fluoride^172,173^

Overall, some of the synthetic routes described above give access to both 2-deoxy-2-fluoroglucosyl fluoride anomers **2** as pure compounds, as well as to the β-anomer of 2-deoxy-2-fluoromannose **3**. The 2-deoxy-2-fluoromannose α-anomer α-**3**, obtained as described in Scheme 8, could not be separated from the α-anomer of 2-deoxy-2-fluoroglucose α-**2**. The Foster group described an anomerization process to access the α- from the β-anomer, by treatment with liquid HF at low temperature (Scheme 10), a process which turned out to be complete in 15 min on the 650 mg scale.^172^

##### 3.1.1.2 1,2-Difluorinated Quinovose/Rhamnose Derivatives

**Scheme 11:**
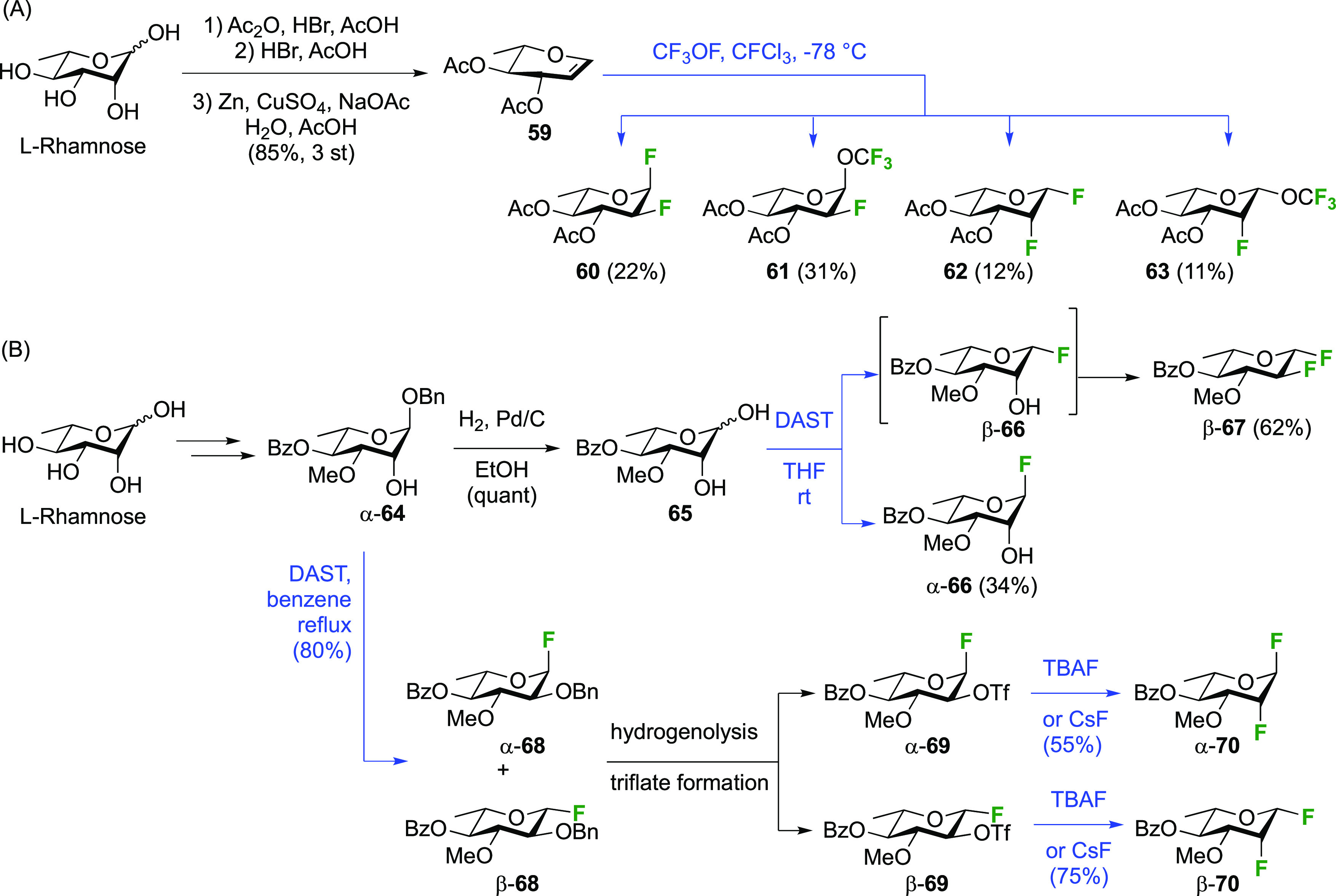
Direct and Sequential Vicinal Difluorination Approaches to 1,2-Difluorinated Quinovose and Rhamnose Derivatives^181,183^

The Kent group described the addition of CF_3_OF with 3,4-di-*O*-acetylated l-rhamnal **59** (Scheme 11A).^181^ The rhamnal derivative can be synthesized from rhamnose by peracetylation, anomeric bromide formation, and elimination, for example by the conditions shown.^182^ The reaction led to the l-quinovose derivatives **60** and **61** as the major addition products, with the l-rhamnose derivatives **62** and **63** as the minor products. As part of work aimed at the synthesis of fluorinated oleandrose analogues, the Lukacs group described a direct DAST-mediated 1,2-dideoxy difluorination of the l-rhamnose derivative **65** (Scheme 11B),^183^ itself synthesized from l-rhamnose.^184,185^ This reaction gave a separable mixture of 4-*O*-benzoyl-2-deoxy-2-fluoro-3-*O*-methyl-β-l-quinovosyl fluoride β-**67** and the corresponding α-l-rhamnosyl fluoride α-**66** in excellent overall yield. In full accordance with the corresponding dideoxy difluorination of the d-mannose derivative **24** (cf. Scheme 7), deoxyfluorination at the anomeric position precedes reaction at C-2, with the second deoxyfluorination only occurring when the anomeric fluorine substituent is in the equatorial position, so only β-**66** reacts and α-**66** is recovered after the workup.

The Lukacs group also reported a sequential approach in which the anomeric fluoride is introduced first (Scheme 11B). Reaction of α-**64** with DAST results in the two inseparable anomeric quinovosyl fluorides α-**68** and β-**68**, for which the anomeric benzyl group has migrated to the 2-position with inversion of configuration,^183^ a rearrangement originally described by the Lemieux group from 2-iodinated glycosides,^186^ and later more or less simultaneously described by a number of groups (Nicolaou, Withers, Kovac, and Lukacs) when using DAST.^77,141−143,187^ Removing the benzyl group allowed activation of the OH-2 as triflate (no conditions/yields provided), leading to the separable α-**69** and β-**69**. Displacement with fluoride was then achieved in both cases, with a lower yield for α-**70** vs β-**70** due to the electronic influence of the adjacent axial anomeric fluoride in α-**69** hampering the deoxyfluorination.^119,188^

##### 3.1.1.3 1,2-Difluorinated Galactose Derivatives

**Scheme 12:**
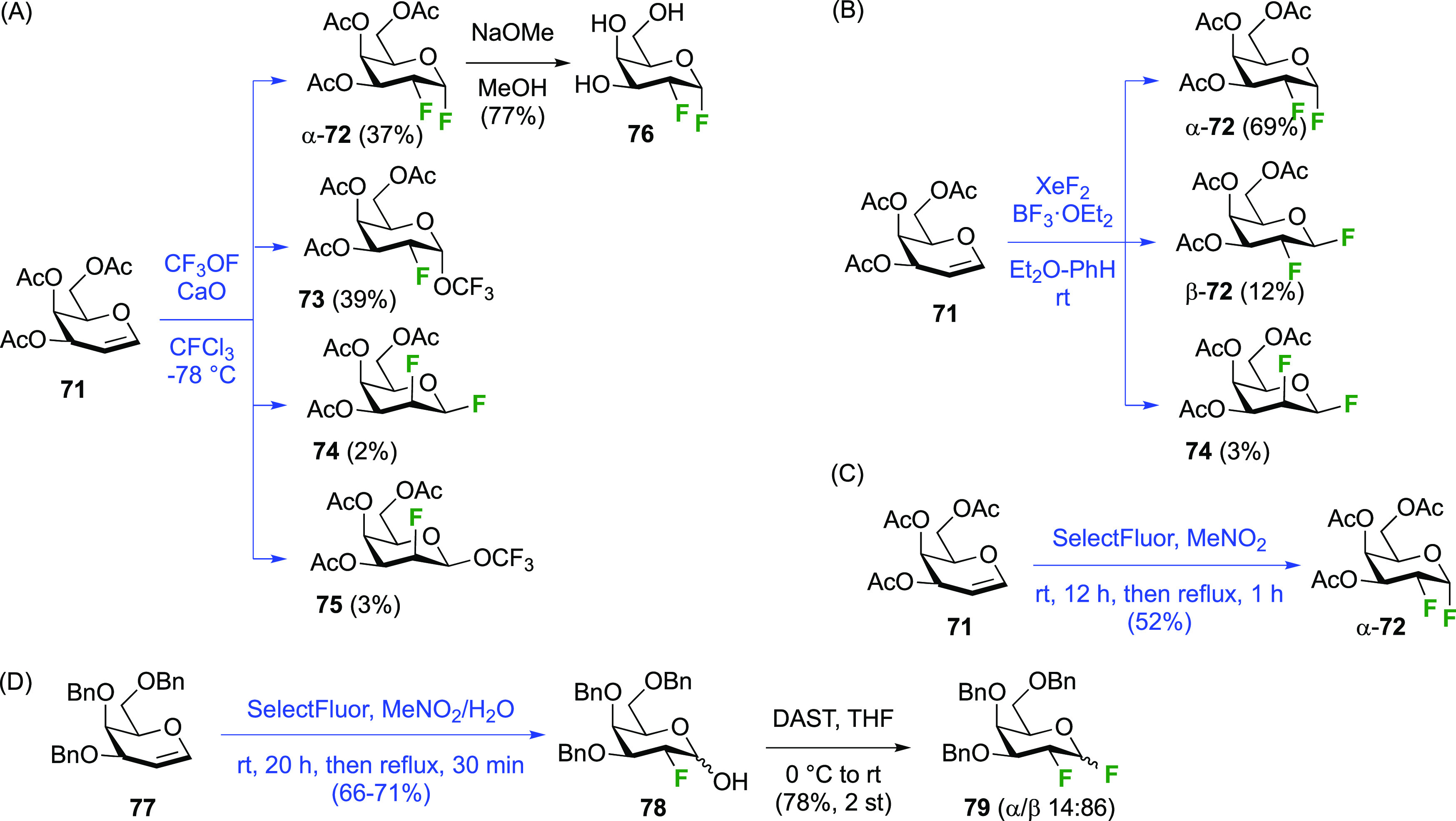
Vicinal Difluorinations with Galactal Derivatives^160,161,189−192,194,195^

Vicinal fluorinations starting from galactal derivatives are shown in Scheme 12. With tri-*O*-acetyl-d-galactal **71** (Scheme 12A), reaction with CF_3_OF led to the four types of compounds also seen with glucal. The *syn*-addition products from the α-face, α-**72** and **73**, were obtained as major isomers, with the β-talose derivatives **74** and **75** being the minor isomers.^189,190^ The yields represent the amounts of recrystallized material, with the authors noting that chromatographic isolation from the mother liquors would lead to higher yields for the *galacto*-derivatives. Dwek et al. reported yields of 55% and 40% for α-**72** and **73** as the only isolated products for this reaction (not shown).^191^ Nevertheless it is clear that the *galacto*:*talo* ratio is much larger than the *gluco*:*manno* ratio, which can be ascribed to the increased steric hindrance of the galactal 4-position compared to that of glucal.^190^ Compound α-**72** was deprotected to give 2-deoxy-2-fluoro-α-d-galactosyl fluoride **76**.^161^

Reaction of **71** with XeF_2_ was investigated in detail by the Korytnyk group (Scheme 12B).^160,161^ A very similar stereochemical outcome compared to d-glucal is now obtained, with the α-2-fluorogalactosyl fluoride product α-**72** being the major product alongside small amounts of its β-anomer, and of the product arising from β-face attack, being the β-talose derivative **74**. The Wong group reported a 78% yield of α-**72** for this reaction.^192^ Geilen et al. achieved the reaction with XeF_2_ in CFCl_3_ without Lewis acid catalysis, although the 1,2-difluoride was not isolated and immediately hydrolyzed to 2-deoxy-2-fluorolagactose (in 63% yield, not shown).^193^ Interestingly, they did not observe the formation of any talose isomers.

The Dax group reported that difluorination of **71** with SelectFluor was very selective as well (Scheme 12C), with α-**72** isolated as the only product in 52% yield.^194^

A sequential approach with 3,4,6-tri-*O*-benzyl-2-deoxy-2-fluorogalactose **78** has also been reported (Scheme 12D).^195^ This material can be obtained via SelectFluor addition to the corresponding galactal **77**, but with water added to the reaction mixture.^196,197^

##### 3.1.1.4 Difluorinated Fucose Derivatives

**Scheme 13:**
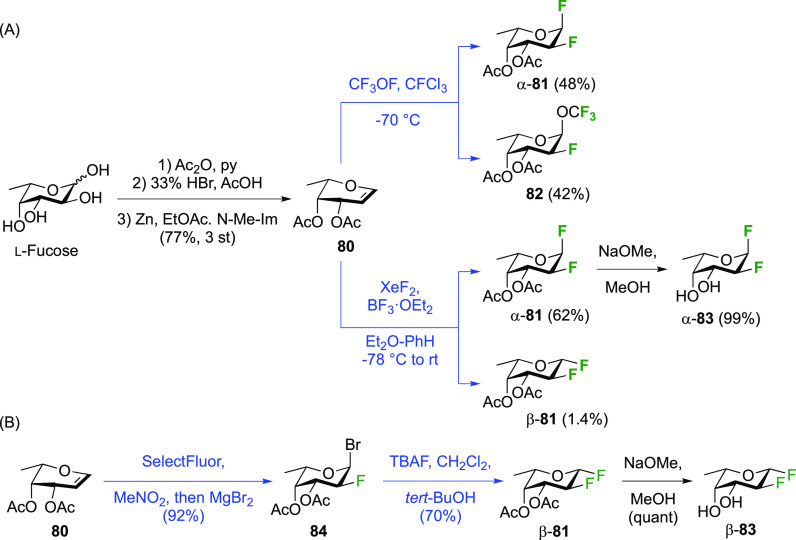
Synthesis of Both 1,2-Difluorinated Fucose Anomers via a Direct and a Sequential Approach^161,199,200^

The synthesis of both 1,2-difluorinated fucose anomers has been described via 3,4-di-*O*-acetyl-l-fucal **80** (Scheme 13). Fucal **80** can be synthesized from l-fucose via peracetylation, anomeric bromide formation, and elimination.^198^ Direct difluorination of **80** using CF_3_OF was described by the Kent group (Scheme 13A), giving the α-configured 2-fluorofucosyl fluoride α-**81** as the major product, alongside the unavoidable trifluoromethyl glycoside byproduct **82**.^199^ Korytnyk used XeF_2_ addition to achieve direct 1,2-difluorination, leading to α-**81** as the major product, with a minor amount of the β-anomer.^161^ Performed at room temperature, a 53% yield of α-**81** was obtained, but starting the process at low temperature improved the yield to 62%. Deprotection then afforded 2-deoxy-2-fluoro-α-d-fucosyl fluoride α-**83**. Alternatively, the Wang group achieved a sequential fluorination approach (Scheme 13B) to give the other anomer.^200^ The fucal derivative **80** was fluorinated in anhydrous SelectFluor, with bromide as an additional nucleophile to directly afford the 2-fluorinated anomeric bromide **84**.^194,198,201^ Bromide displacement with fluoride proceeded with inversion of configuration to give β-**89** as the only reported anomer, and deprotection then afforded 2-deoxy-2-fluoro-β-d-fucosyl fluoride β-**83**.^200^

##### 3.1.1.5 1,2-Difluorinated Uronic Acid Derivatives

**Scheme 14:**
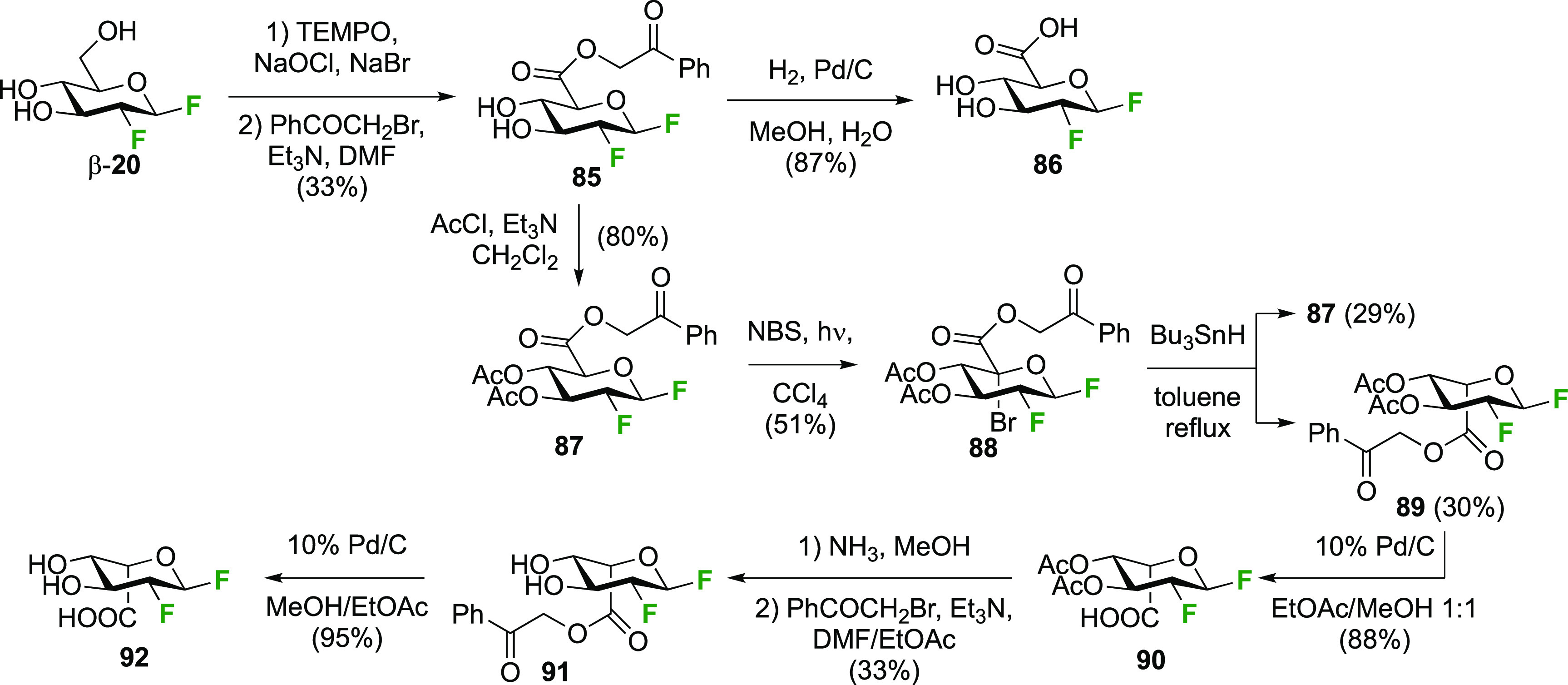
Synthesis of 1,2-Difluorinated Uronic Acid Derivatives^174,202^

The Withers group reported a synthesis of 2-deoxy-2-fluoro-β-d-glucopyranosyluronic acid **86** starting from the 1,2-difluorinated glucose derivative β-**20** (Scheme 14).^174^ TEMPO-mediated oxidation of the primary alcohol in β-**20** led to the corresponding glucuronic acid, which was protected as phenacyl ester **85** to aid purification. Its hydrogenolysis then afforded **86**.

The corresponding iduronic acid derivative **92** could also be prepared from **85**.^202^ Acetylation led to **87**, which was subjected to radical bromination conditions leading to **88**. Tributyl tin hydride-mediated radical reduction of the bromide led to a 1:1 mixture of C-5 epimers, which could be separated. The iduronic ester **89** was hydrogenolyzed to give **90**, after which the acetates were cleaved. However, pure **92** could only be obtained after reprotection of the carboxylic acid, chromatography, then deprotection.

##### 3.1.1.6 DAST-Mediated Rearrangement

**Scheme 15:**
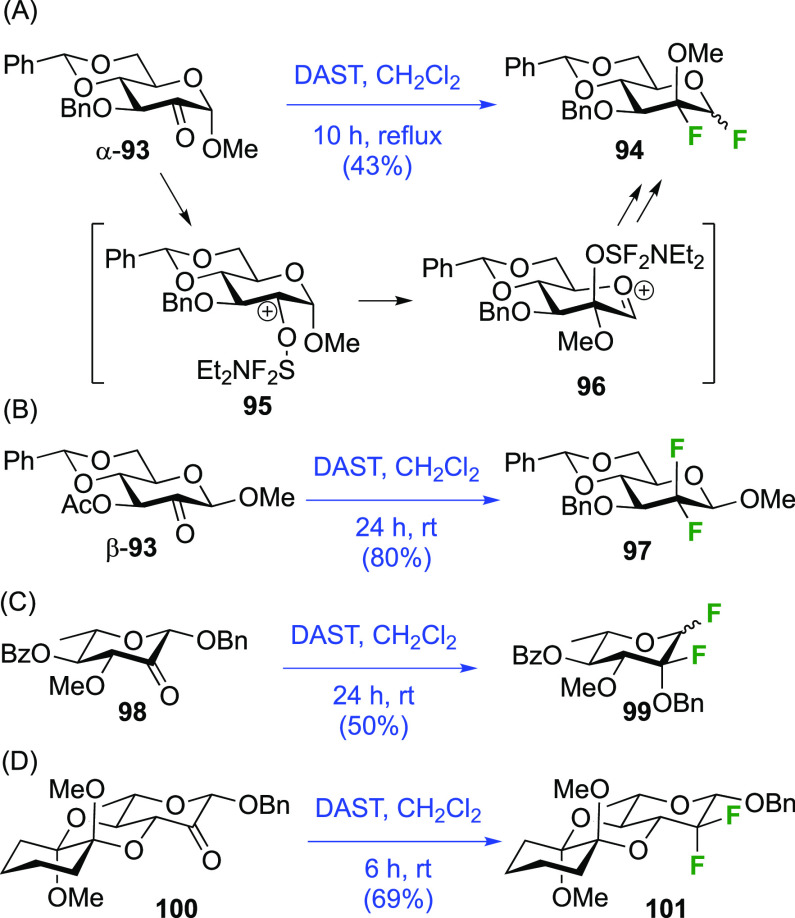
Synthesis of 1,2-Difluorinated Derivatives via a DAST-Mediated Rearrangement Process^203,204^

Based on the possible rearrangement initiated by DAST-mediated deoxyfluorination at the OH-2 position,^142,143^ the Castillòn group developed a synthetic approach toward 1,2-difluorinated sugar derivatives based on a DAST-mediated rearrangement process starting from 2-uloses (Scheme 15A).^203^ DeoxoFluorination of α-**93** was shown to lead to **94**. Given that the methoxy group ends up on the other pyranose face, it is proposed that the rearrangement occurs immediately upon DAST-activation of the carbonyl (**95**), followed by fluoride substitution of the resulting axial activated alcohol group (**96**) with inversion of configuration. When the corresponding β-anomeric substrate β-**93** (Scheme 15B) was subjected to DAST, a much faster reaction occurred, giving the expected *gem*-difluorinated **97** in good yield, with the equatorial anomeric substituent unable to initiate neighboring group participation. However, the ketone **98** derived from rhamnose (Scheme 15C) did lead to the rearrangement product **99**,^204^ which was explained by facile ring inversion of **98**. Indeed, with a protecting group locking the pyranose conformation as in **100** (Scheme 15D), the *gem*-difluorination product **101** was obtained.

#### 3.1.2 Trifluorinated at Positions 1 and 2

##### 3.1.2.1 1,1,2-Trifluorinated

**Scheme 16:**
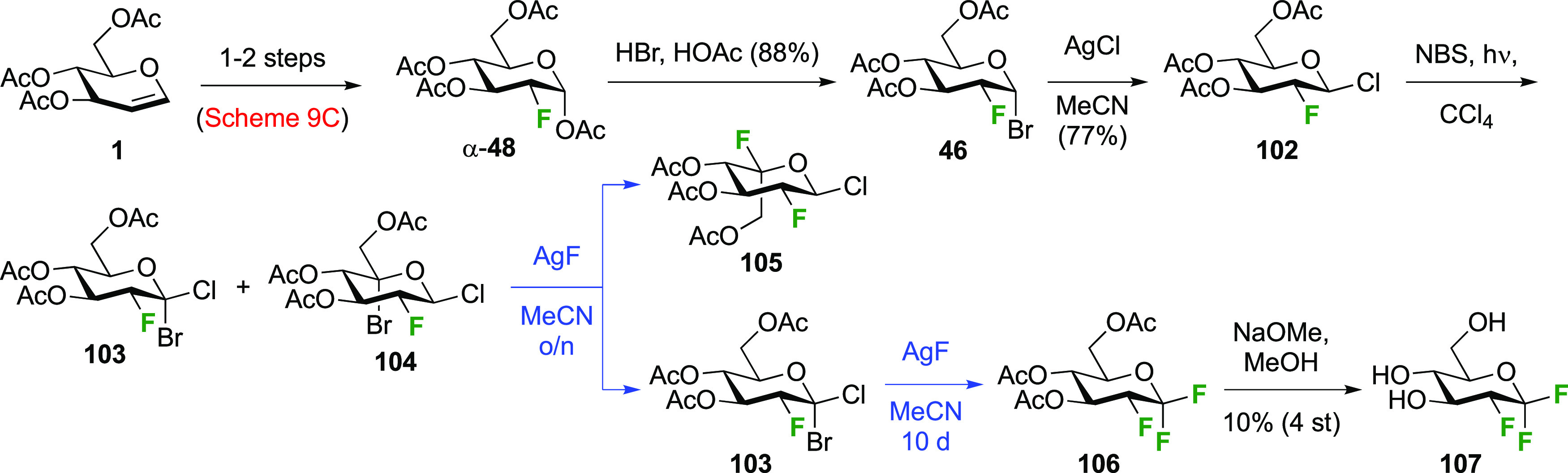
Synthesis of 2-Deoxy-1,2-difluoro-d-glucopyranosyl Fluoride^205^

As part of a study investigating the effect of different fluorine substitutions on the rates of glycosidation and deglycosylation upon reaction with glycosidase enzymes, the Withers group synthesized 2-deoxy-1,2-difluoro-d-glucopyranosyl fluoride **107** (Scheme 16) starting from the 2-fluorinated glucosyl bromide **46**.^205^ This is prepared from the corresponding tetraacetate α-**48**, which was obtained as shown in Scheme 9C from commercially available tri-*O*-acetyl-d-glucal.^177^ Conversion of α-**48** to the corresponding glycosyl bromide **46** was achieved by acetylation and HBr treatment.^172,206^ Halide exchange with inversion of configuration led to **102**, which was subjected to radical bromination. This reaction was not selective, leading to bromination at both C-1 and C-5. The inseparable mixture of **103** and **104** was subjected to silver fluoride. Upon being stirred overnight, only **104** reacted to give **105**, after which the unreacted **103** could be isolated cleanly. Interestingly, only the bromide at C-5 was displaced, with inversion of configuration. Subjecting **103** to the same AgF-mediated halide exchange reaction, but now for 10 days, led to displacement of both anomeric halides,^207^ giving **106**. The slow displacement at C-1 is a result of the electron withdrawing effect of the fluorination at C-2. Deprotection led to 2-deoxy-1,2-difluoro-d-glucopyranosyl fluoride **107** in 10% yield from **102**.

##### 3.1.2.2 1,2,2-Trifluorinated

**Scheme 17:**
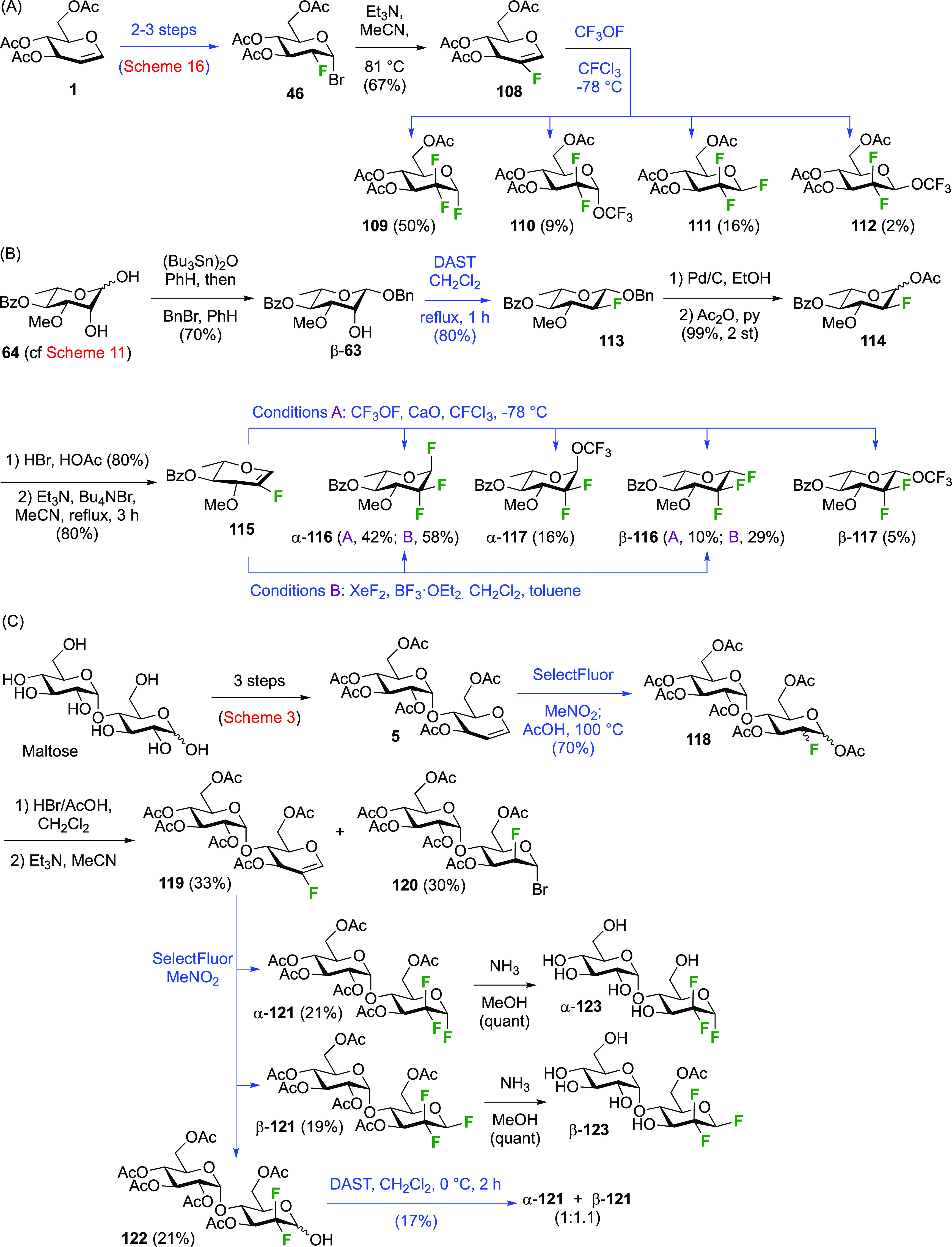
Synthesis of 1,2,2-Trifluorinated Sugar Derivatives^154,185,208^

The synthesis of 1,2,2-trifluorinated compounds has been achieved via fluorination of 2-fluoroglycal derivatives. Adamson et al. investigated the reaction between the 2-fluoroglucal derivative **108** and CF_3_OF (Scheme 17A).^208^ The fluoroglucal was synthesized from the 2-deoxy-2-fluoroglucosyl bromide **46**, for which the synthesis was shown above in Scheme 16, by base-mediated elimination.^162^ The reaction of **108** with CF_3_OF under the same conditions as for the corresponding glucal (cf. Scheme 4) resulted in a similar reaction outcome in that both the glucosyl fluorides and the trifluoromethyl glucosides were formed, with the α-anomer being the major product in both cases.

The Lukacs group reported a similar outcome for the reaction of the 2-fluororhamnal derivative **115** (Scheme 17B),^185^ with α-**116** isolated as the major product. With XeF_2_ as the reagent, α-**116** and β-**116** were isolated in excellent yield. The fluororhamnal was synthesized from the advanced intermediate **64**,^183^ with first installation of an equatorial anomeric substituent to facilitate deoxyfluorination of the axial OH-2. This was achieved by using stannylene acetal methodology, and DAST-treatment of β-**63** afforded the 2-deoxy-2-fluoroquinovose derivative **113** in excellent yield. Anomeric deprotection and acetylation was followed by anomeric bromination, upon which bromide elimination then gave the 2-fluororhamnal **115**.

The synthesis of 1,2,2-trifluorinated maltose as a nonhydrolyzable mimic of maltose-1-phosphate was achieved by Thanna et al. (Scheme 17C),^154^ with 2-fluoromaltal **119** as key intermediate. Its synthesis started from maltose as shown in Scheme 3. Reaction of peracetylated maltal **5** with SelectFluor followed by heating with acetic acid delivered **118** as a mixture of four stereomers.^209^ A two-step procedure in which **5** was first reacted with SelectFluor in water followed by acetylation was lower-yielding (not shown).^154^ From **118**, formation of the corresponding glycosyl bromide was followed by E2-elimination of the anomeric bromide to give peracetylated 2-fluoromaltal **119** alongside unreacted **120**, whose mannose stereochemistry prevented bromide elimination.^154,209^ Reaction of the 2-fluoromaltal derivative **119** with SelectFluor in nitromethane without an added nucleophile resulted in both peracetylated 2-deoxy-2,2-difluoromaltosyl fluoride anomers α-**121** and β-**121** in an 1.1:1 ratio of isolated yields, with hydrolyzed byproduct **122**. This byproduct could be converted to α- and β-**121** by a DAST-mediated deoxyfluorination, albeit in low yield. Finally, protecting group aminolysis gave 2-deoxy-2,2-difluoro-α-maltosyl fluoride α-**123** and 2-deoxy-2,2-difluoro-β-maltosyl fluoride β-**123**. The 2-fluoromaltal derivative **119** was also reacted with XeF_2_ to give a 12% yield of anomers **121** (not shown).

### 3.2 Fluorination at Positions 1 and 3

**Scheme 18:**
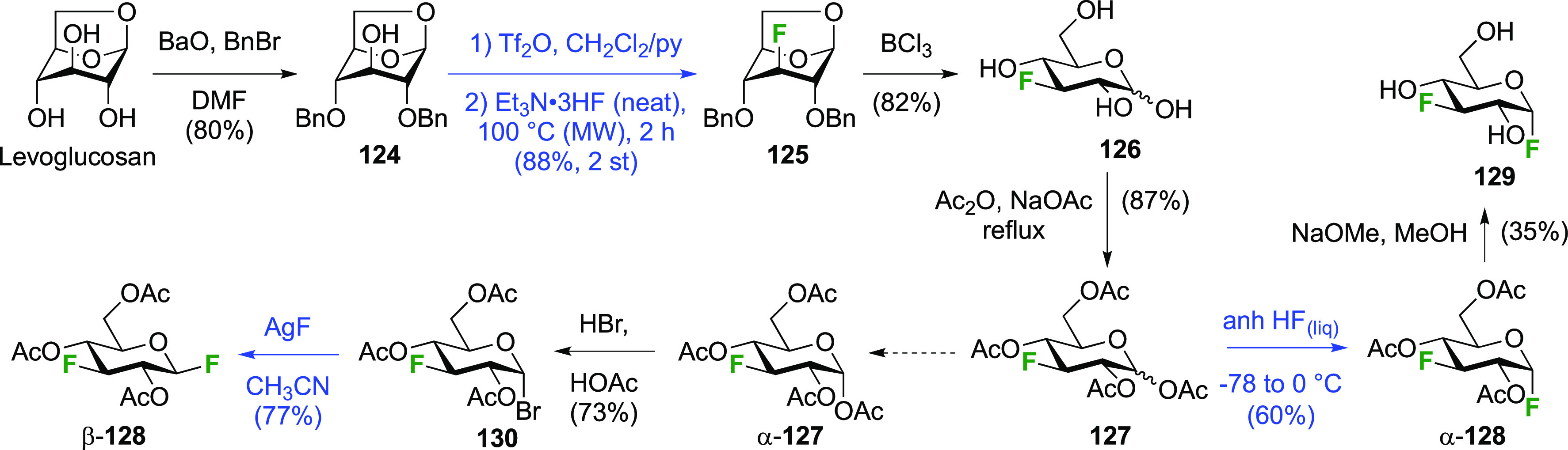
Synthesis of 3-Dideoxy-3-fluoro-glucosyl Fluorides^59,210−212^

The synthesis of 3-dideoxy-3-fluoro-α-glucosyl fluoride **129** was achieved from 3-deoxy-3-fluoroglucose **126** by a number of groups (Scheme 18).^59,210−212^ This starting material is commercially available but expensive. It can be synthesized from glucose diacetonide in 4–5 steps (compare Scheme 56, compound **413**),^139,213,214^ but a shorter alternative developed by Giguère involves selective benzylation of levoglucosan to give **124** followed by a 2-step retentive deoxyfluorination to **125** and anhydro-bridge opening.^215,216^ Acetylation of the resulting **126** led to the peracetate **127**, from which the anomeric acetate mixture was converted to the α-configured glucosyl fluoride α-**128** by treatment in liquid HF in good yield. Acetate removal then gave **129**.^59,211,212^ The corresponding β-anomeric glucosyl fluoride was obtained through conversion of α-**127** to the anomeric bromide **130**, followed by treatment with AgF in acetonitrile. This gave β-**128** in 77% yield.^210^

### 3.3 Fluorination at Positions 1 and 4

**Scheme 19:**
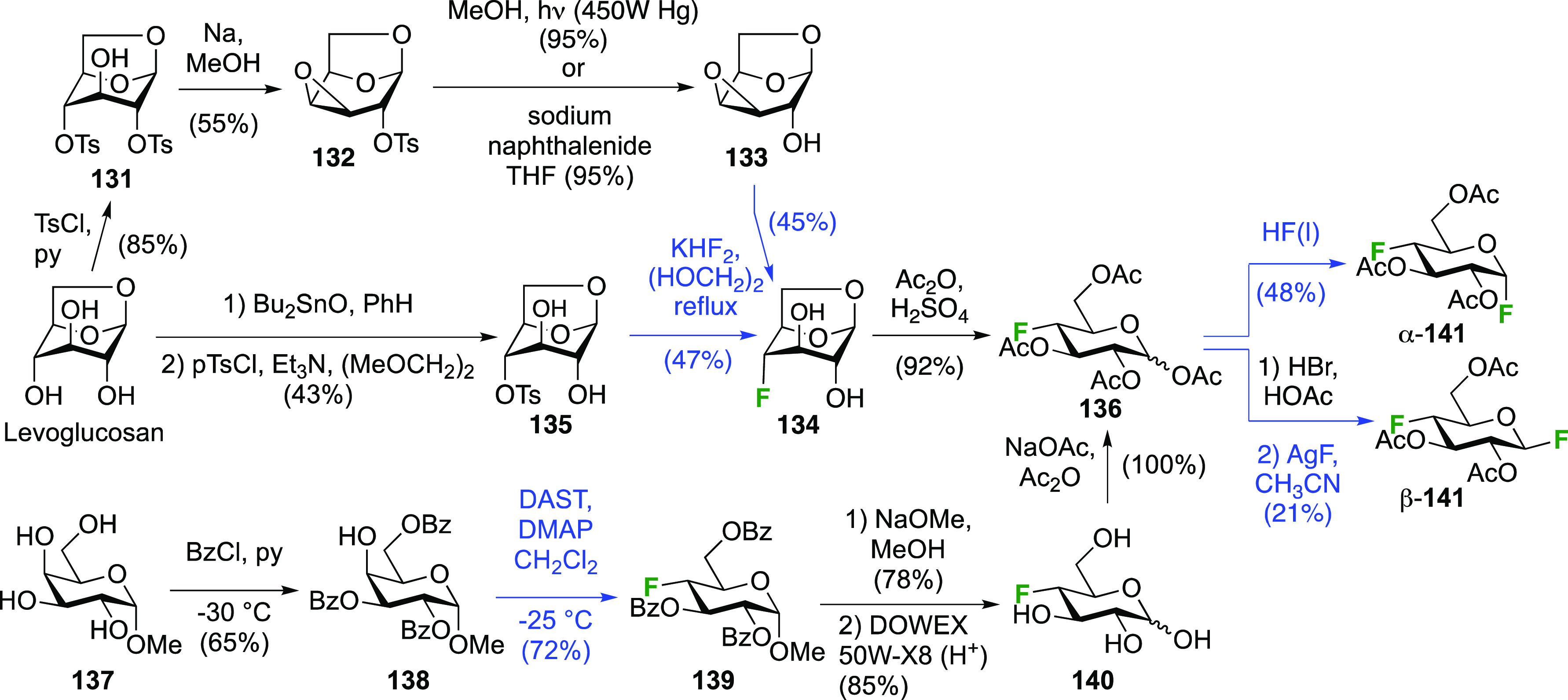
Synthesis of (Peracetylated) 4-Deoxy-α and -β-glucopyranosyl Fluorides^217^

The 1,4-difluorinated derivatives of glucose and galactose have been reported. Again, the anomeric fluoride is introduced last, and hence the 4-deoxy-4-fluoroglucose and -galactose precursors are obtained first. These, and their peracetates, are commercially available but expensive. Both anomeric glycosyl fluorides of 4-deoxy-4-fluoroglucose, **141**, can be prepared selectively from the corresponding peracetate **136** (Scheme 19), which in turn can be obtained from levoglucosan.^217^ Its selective tosylation at the 2,4-positions gave **131**,^215,218^ which upon deprotonation of the OH-3 selectively formed the 3,4-epoxide **132**.^219−222^ Photolytic cleavage of the tosyl group was used to deprotect the OH-2 group to give **133**.^217^ The Linclau group later used the Robins procedure^223^ to remove the tosyl group in **132** in equally high yield.^224^ This was followed by regioselective epoxide opening with fluoride to give **134** in good yield.^217^ The regioselectivity of the epoxide opening is governed by the so-called Fürst-Plattner effect,^122^ which originates from the formation of a chairlike transition state which cannot be obtained upon reaction at C-3. A more direct synthesis of **134** was possible from the monotosylate **135** using the same conditions. This reaction proceeds by in situ conversion of **135** to epoxide **133** by the basic fluoride, followed by epoxide opening. Monotosylation of levoglucosan was reported to be very low yielding and unselective,^225^ but a reasonable yield of **135** could be obtained on gram scale with prior formation of the 2,4-stannylene acetal. However, chromatographic separation of the corresponding 2,4-di-*O*-tosylate (6%) and the 2-*O*-tosylate (17%) side products was required.^226,227^ Acetolysis of **134** gave **136** in excellent yield. Alternatively, **136** can be synthesized from methyl α-d-galactopyranoside **137**,^214^ starting with a regioselective benzoylation to give **138**.^228^ Fluorination with inversion of configuration affords the 4-deoxyfluorinated glucose derivative **139**.^214,229,230^ These two steps have been conducted on the kilogram scale.^231^ Deprotections then give 4-deoxy-4-fluoroglucose **140**,^214^ which is acetylated to give **136**.^214,232^ Treatment of **136** with liquid HF led to the α-anomeric fluoride α-**141**.^217^ The β-anomeric fluoride β-**141** was obtained in a 2-step procedure via the anomeric bromide.

**Scheme 20:**
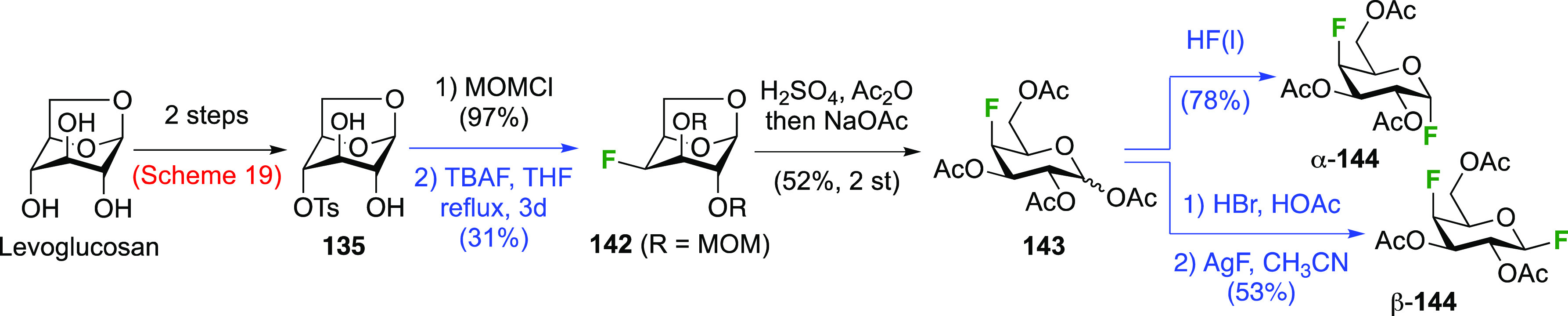
Syntheses of (Peracetylated) 4-Deoxy-α and -β-galactopyranosyl Fluorides^233^

The galactosyl fluorides α- and β-**144** were obtained in the same way from the peracetylated 4-deoxy-4-fluorogalactopyranose **143** (Scheme 20).^233^ There are many different syntheses toward **143** starting from methyl glucoside, with various protecting group manipulations to allow selective fluorination of OH-4 (either directly with DAST or using a variety of leaving groups), but the example shown from the Giguère group, starting from levoglucosan, is perhaps the most convenient.^227^ Selective 4-*O*-tosylation as described in Scheme 19 is followed by MOM-protection of the remaining alcohol groups. Fluorine displacement to give **142** can be achieved in modest yield, and a one-pot deprotection operation leads to the peracetate **143**,^227^ which can then be converted to α- and β-**144**.^233^ Alternatively, the tosylate group can be removed using sodium naphthalenide (92%, not shown), and fluorination can be achieved in much higher yield (80%) using Et_3_N·3HF via the triflate.^234^

### 3.4 Fluorination at Positions 1 and 5

#### 3.4.1 Difluorinated at Positions 1 and 5

Fluorination at C-5 has been well-investigated given the use of 5-fluorosugar derivatives as mechanism-based inhibitors of glycosidase enzymes,^21,235,236^ as pioneered by the Withers group.^21^ Its introduction is a two-step process, starting with a radical bromination at C-5.^237,238^ Given that halide introduction at C-5 prevents selective reactions at the anomeric position,^239^ glycosyl fluorides are used as substrates when additional fluorination at the anomeric position is required.

**Scheme 21:**
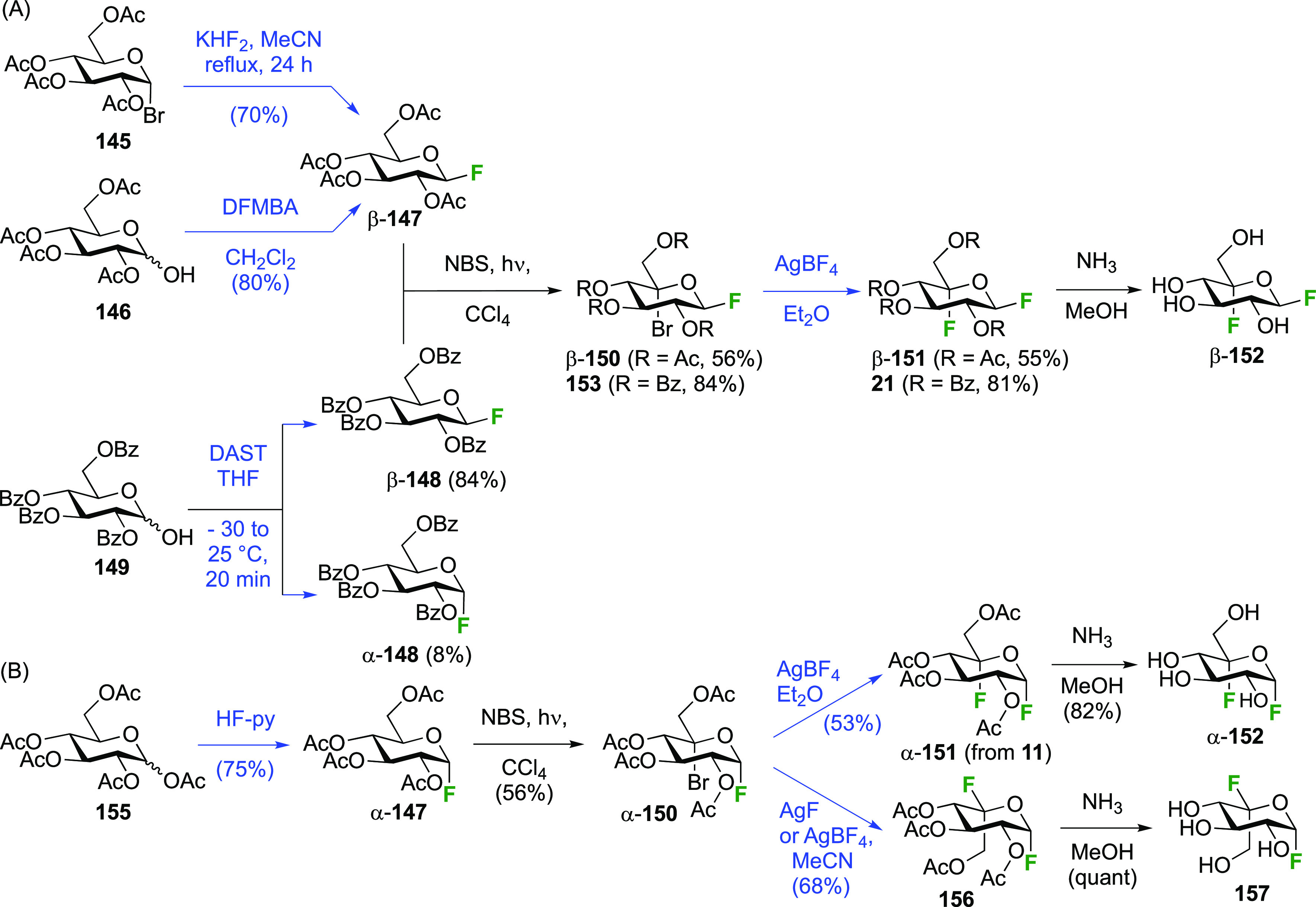
Synthesis of 5-Fluororinated Glucopyranosyl Fluoride and Idopyranosyl Fluoride^154,235,239,247^

Both anomers of 5-fluoroglycosyl fluoride **152** have been synthesized (Scheme 21).

The substrate β-glucosyl fluoride **147** can be obtained by a number of means,^240^ for example treatment of the anomeric bromide **145** with KHF_2_ or with AgF (not shown) in MeCN,^241−243^ or treatment of 2,3,4,6-glucose tetraacetate **146** with DFMBA.^133^ The 2,3,4,6-tetra-*O*-benzoyl-β-glucosyl fluoride β-**148** has been synthesized from **149** by deoxyfluorination with DAST.^244^ Radical bromination of β-**147** led to β-**150**,^207,235,239,245^ with only a small amount (4%) of anomeric bromination byproduct (not shown).^207^ Halide exchange with AgBF_4_ proceeded with retention of configuration to give β-**151**, with use of Et_2_O as the solvent found to be superior over toluene.^239^ Acetate aminolysis then gave β-**152**.^235^ The bromination of the tetrabenzoate β-**148** was reported to be slower, but better yields were obtained.^239^

Radical fluorination of the α-configured glucosyl fluoride α-**147** (Scheme 21B), which can be obtained in one step from **155**,^95,96,154,246^ proceeded equally well with no anomeric bromination,^154,207^ but this reaction on the corresponding tetrabenzoate was reported not to give clean conversion (not shown).^239^ Halide exchange was not hindered by the axial fluoride at C-1 and proceeded with retention as well, giving α-**151**. However, when AgF or AgBF_4_ were reacted with α-**150** in MeCN, inversion of configuration took place, leading to the l-ido configured **156**.^154^ All attempts to isomerize **156** to α-**151** using HF-py or with Lewis acids were not successful. Deprotection of α-**151** and **156** yielded α-**152** and **157** respectively.^154,235,239,247^

**Scheme 22:**
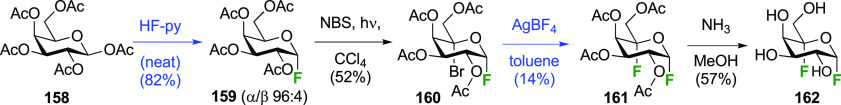
Synthesis of 5-Fluoro-β-d-galactopyranosyl Fluoride^248^

The Withers synthesis of the α-configured 5-fluorogalactosyl fluoride **162** is shown in Scheme 22.^248^ Reaction of peracetylated galactose **158** with HF-py afforded the corresponding α-galactosyl fluoride **159**,^246,249^ which was brominated at C-5 to give **160** in similar yields as shown above. Halide exchange at C-5 proved not possible with AgF but was achieved with AgBF_4_ in toluene, leading to **161** with retention of configuration, albeit in a low yield. Interestingly, when Et_2_O was used as the solvent, a 1:1 mixture of **161** and the corresponding l-altrose epimer (not shown) was obtained, resulting from inversion of configuration at C-5. Aminolysis finally provided 5-fluoro-β-d-galactopyranosyl fluoride **162**.

**Scheme 23:**
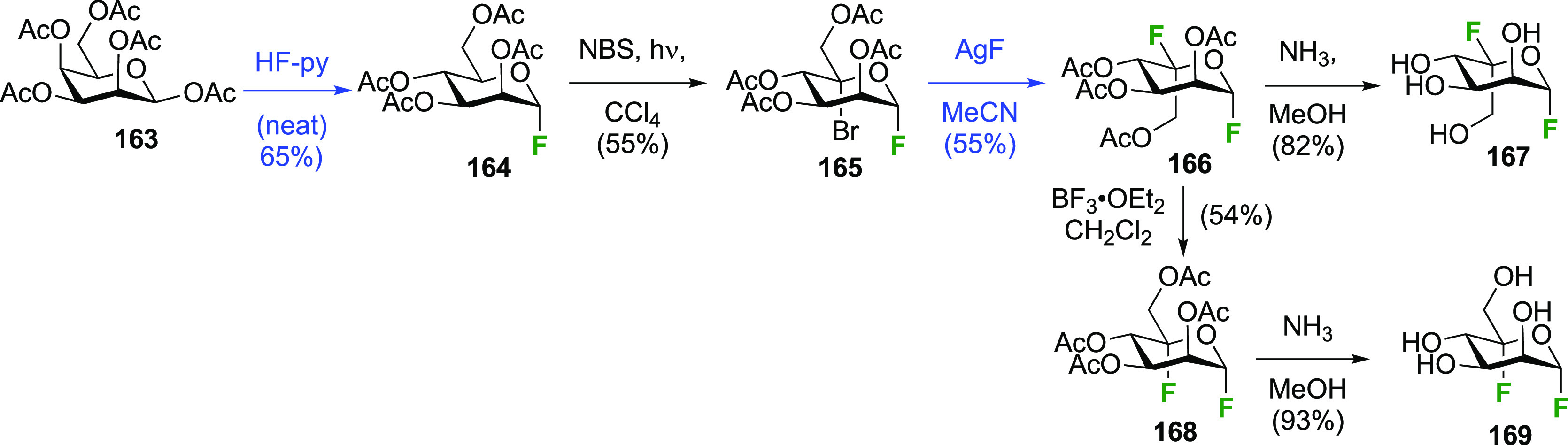
Synthesis of 5-Fluoro-α-d-mannopyranosyl Fluoride and 5-Fluoro-β-l-gulopyranosyl fluoride^250^

The sequence starting from α-configured peracetylated mannosyl fluoride **164**, which can be obtained from mannose pentaacetate **163**,^246^ is shown in Scheme 23.^250^ Radical bromination to **165** again proceeded with similar yields compared to the aforementioned glycosyl fluorides. Halide exchange was effected with AgF, which proceeded with inversion of configuration to give the l-gulose derivative **166**. Deprotection of **166** yielded 5-fluoro-β-l-gulopyranosyl fluoride **167**, which was shown by ^1^H NMR analysis to adopt a boat-like conformation. Interestingly, treatment of **166** with BF_3_-etherate caused epimerization at C-5 toward the more stable mannosyl derivative **168**, which upon aminolysis led to 5-fluoro-α-d-mannopyranosyl fluoride **169**. This compound was shown to adopt a ^4^*C*_1_ chair conformation in solution (^1^H NMR analysis), as well as in the solid state (X-ray crystallographic analysis).

**Scheme 24:**
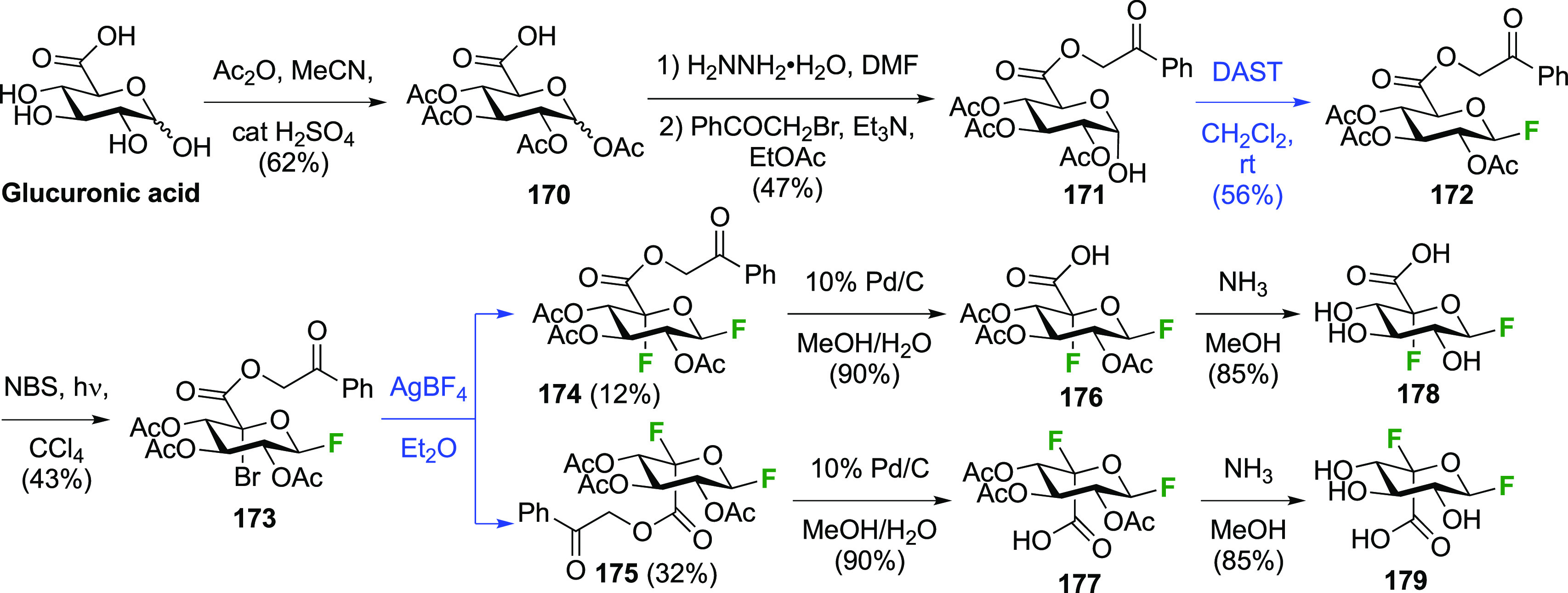
Synthesis of 5-Fluoro-β-d-glucopyranosyl and -α-l-idopyranosyl Uronic Acid Fluorides^202,251^

For the synthesis of 5-fluorinated uronic acids (Scheme 24),^251^ the carboxylic acid protecting group needed to be removable under conditions that were mild enough for the difluoride to survive, while also being compatible with the radical bromination and fluorination conditions. This ruled out methyl, benzyl, and silyl esters. In the event, after alcohol acetylation of glucuronic acid and anomeric deprotection, the Withers group used a phenacyl protecting group, leading to **172**. Then, the introduction of the anomeric fluoride was achieved with DAST to give a mixture of anomers, predominantly the β-anomer **172**. Radical bromination to give **173** proved to be much faster compared to the corresponding peracetylated glucosyl fluoride, which was ascribed to the stabilization of the intermediate C-5-radical by the carboxyl group. Halide exchange proceeded both with inversion and retention, leading to **174** and **175**.^251^ Both were deprotected in two separate steps to give 5-fluoro-β-d-glucopyranosyl uronic acid fluoride **178** and 5-fluoro-α-l-idopyranosyl uronic acid fluoride **179**.^202,251^

#### 3.4.2 Trifluorinated at Positions 1 and 5

**Scheme 25:**
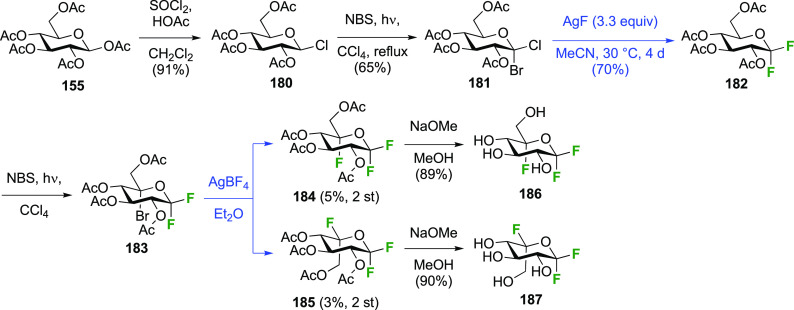
Synthesis of 1,5-Difluoro-d-glucosopyranosyl and -l-idopyranosyl Fluorides^205^

The synthesis of the trifluorinated 1,5-difluoroglycopyranosyl fluorides has also been achieved by the Withers group.^205^ Tetraacetylated β-acetyl chloride **180** (Scheme 25), synthesized from β-d-glucose pentaacetate **155** by treatment with thionyl chloride,^252^ was subjected to the radical bromination process. This gave predominantly the 1-brominated product **181**, alongside 14% of the corresponding C-5-bromination product (not shown).^207,245^ Reaction with AgF resulted in exchange of both anomeric halides to give **182**.^207^ A subsequent radical bromination led to **183**, which was subjected to a halide exchange reaction, giving both of the 1,1,5-trifluorinated products with retention (**184**) and inversion (**185**) of configuration in low yields.^205^ Both were deprotected to give 1,5-difluoro-d-glucosopyranosyl fluoride **186** and 1,5-difluoro-l-idopyranosyl fluoride **187**.

### 3.5 Fluorination at Positions 1 and 6

#### 3.5.1 Difluorinated at Positions 1 and 6

##### 3.5.1.1 1,6-Diluorinated Glucose Derivatives

**Scheme 26:**
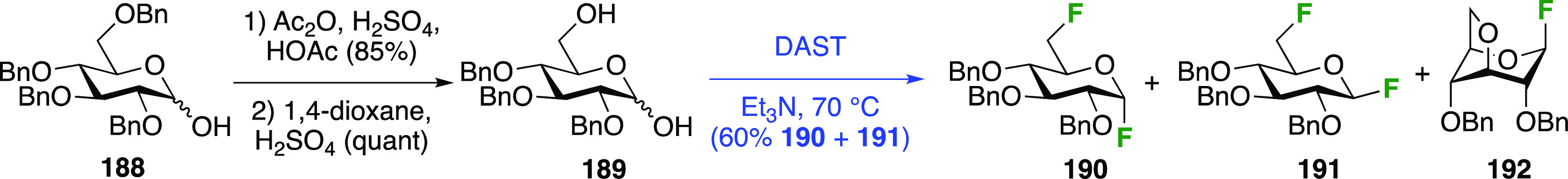
Direct Fluorination Strategy for the Synthesis of 6-Deoxy-6-fluoroglucosyl Fluorides^144^

The Kovac group reported a direct synthesis of 6-deoxy-6-fluoroglucosyl fluoride from 2,3,4-tribenzylated glucose **189** (Scheme 26).^144^ This was prepared by a modified selective acetylation procedure reported by Eby et al. starting from tetrabenzylated glucose **188**, followed by acetate hydrolysis.^253^ Treatment of **189** with DAST gave the desired **190** and **191**, which were separable, with the addition of Et_3_N required to obtain a good yield. Without addition of base, the 3,6-anhydro derivative **192** was isolated as the major product, with the C-3 OBn oxygen displacing the activated OH-6 group after ring inversion. Treatment of methyl 2,3,4-tri-*O*-benzyl galactopyranoside with DAST was also found to give the 3,6-anhydro derivative.^145,254^ In this case the required ring inversion for cyclization will have been facilitated by its anomeric configuration, as it resulted in the β-glucosyl fluoride becoming axial. The action of base was proposed to deprotonate any formed HF, thereby ensuring that fluoride substitution at C-6 outcompeted cyclization.

**Scheme 27:**
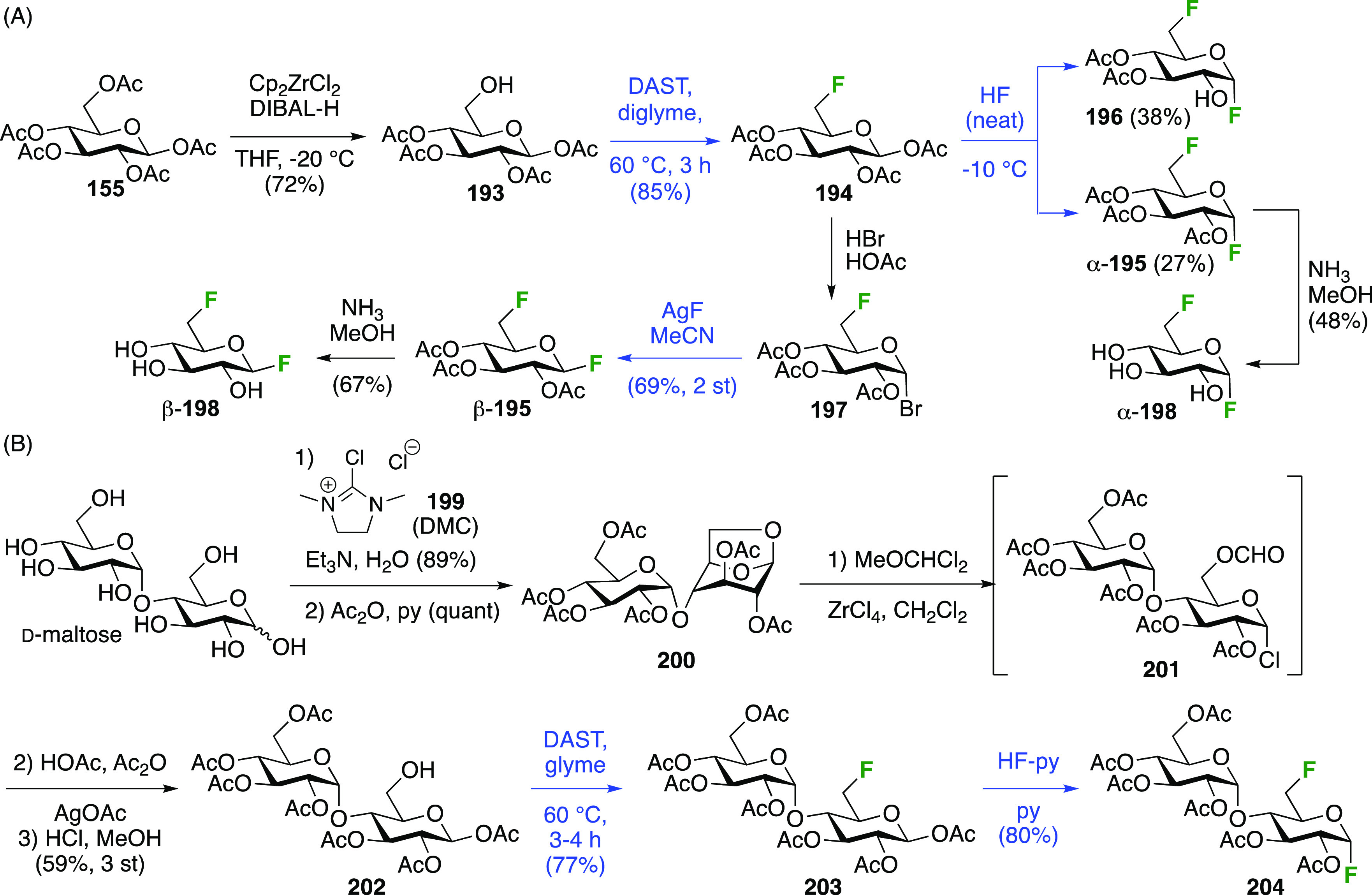
A Sequential Fluorination Approach to 1,6-Difluorinated Sugar Analogues^255,257^

A sequential synthesis approach for both anomeric glucosyl fluorides has also been reported, with 1,2,3,4-tetra-*O*-acetyl-6-deoxy-6-fluoro-β-d-glucopyranose **194** as a key intermediate (Scheme 27A).^255^ This is synthesized from **193**,^125^ for which a one-step synthesis from glucose pentaacetate **155** involving selective 6-deacetylation with Cp_2_ZrCl_2_ is now available.^256^ Treatment of **194** (as an anomeric mixture) with anhydrous HF at low temperature resulted in a mixture of products, from which α-**195** and a partially deprotected byproduct **196** were isolated. The β-anomer can be accessed by prior conversion of **194** to the anomeric bromide **197**, and subsequent treatment with silver fluoride. Deprotection of α- and β-**195** with ammonia affords the α- and β-6-deoxy-6-fluoroglucopyranosyl fluorides α-**198** and β-**198**.

Following similar methodology, the Driguez group synthesized a 1,6-difluorinated maltose analogue (Scheme 27B), with the 1,6-anhydromaltose hexaacetate **200** as key intermediate.^257^ This can be obtained from maltose by reaction with 2-chloro-1,3-dimethylimidazolinium chloride **199**,^258,259^ followed by acetylation.^260^ Selective opening of the 1,6-anhydro-bridge was achieved with dichloromethyl methyl ether in the presence of ZrCl_4_, leading to **201**. This was immediately treated with acetic acid and silver acetate to install the anomeric acetate, and then with hydrochloric acid in methanol for the selective hydrolysis of the 6-*O*-formyl group. This gave **202**, which was deoxyfluorinated with DAST and then treated with HF-py to install the anomeric fluoride to give **204**.

##### 3.5.1.2 1,6-Difluorinated Galactose Derivatives

**Scheme 28:**
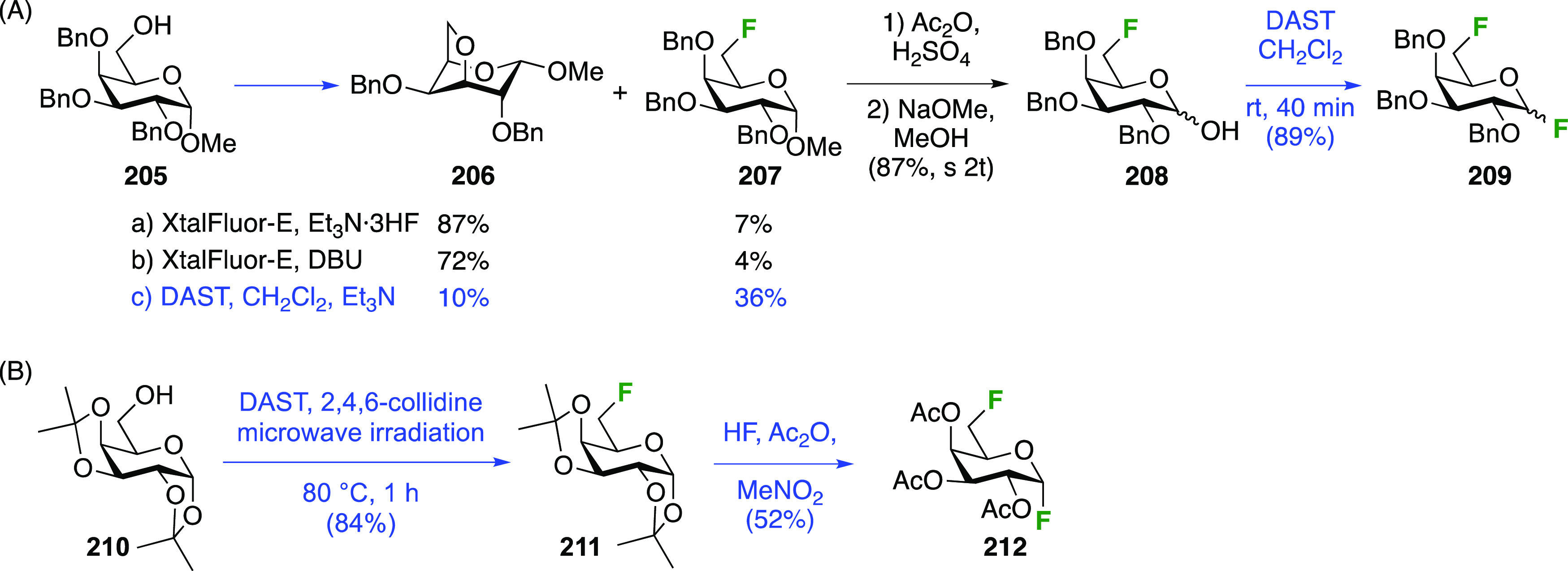
Sequential Fluoride Introduction for a 1,6-Difluorinated Galactose Derivative^261,262^

The Mori group described the synthesis of benzylated 6-deoxy-6-fluorogalactosyl fluoride **209** starting from **205** (Scheme 28A),^261^ which is obtained via a standard 3-step sequence involving tritylation, benzylation, and trityl hydrolysis of methyl α-d-galactopyranoside (not shown). Attempted deoxyfluorination with XtalFluor gave the 3,6-anhydro byproduct **206** as the major product, a side reaction also observed by Kovac as described in Scheme 26. Reaction with DAST also led to **206**, even as the only observable product. As described by Kovac,^144^ addition of base enabled deoxyfluorination at the 6-position to give **207**, albeit in a moderate yield. From **207**, anomeric acetolysis followed by methanolysis of the resulting acetate gave **208**, which was then subjected to anomeric deoxyfluorination to give **209** as a 2:3 α/β mixture of anomers.

A short synthesis of peracetylated 6-deoxy-6-fluoro-α-galactopyranosyl fluoride **212** was reported by the Miethchen group (Scheme 28B).^262^ Starting from commercially available 1,2:3,4-di-*O*-isopropylidene-α-d-galactopyranose **210**, deoxyfluorination is best effected by conditions established by the Hoffman-Roeder group to give **211**,^196,263^ upon which treatment with anhydrous HF in the presence of acetic anhydride gave **212**.^262^

#### 3.5.2 Trifluorinated at Positions 1 and 6

**Scheme 29:**
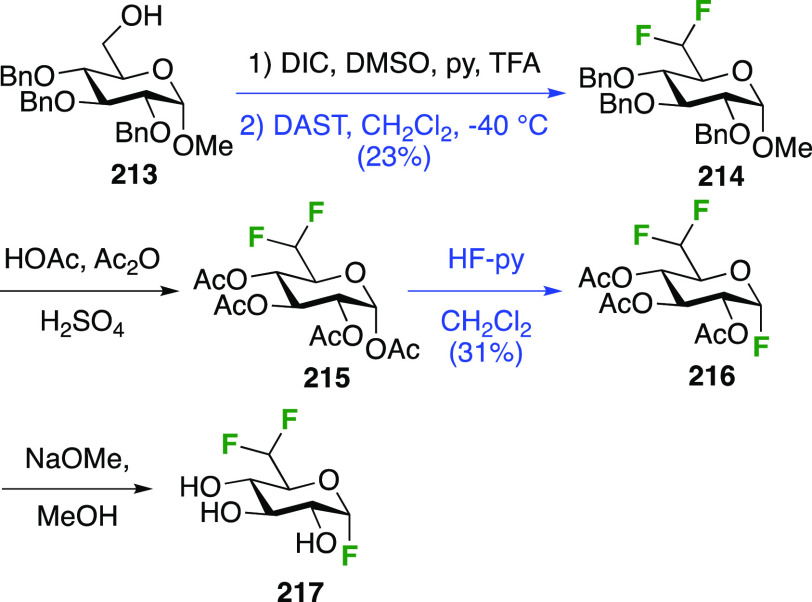
Synthesis of 6-Deoxy-6,6-Difluoroglucosyl Fluoride^264^

The Edwards group reported a synthesis of 6-deoxy-6,6-difluoro-α-d-glucosyl fluoride **217** starting from **213** (Scheme 29).^264^ Pfitzner-Moffatt oxidation with diisopropyl carbodiimide (DIC), directly followed by treatment of the resulting C-6-aldehyde hydrate with DAST, gave **214** in a low yield. Dealkylation by acetolysis resulted in the peracetate **215**, which was converted to the glycosyl fluoride **216**. Alcohol deprotection then gave **217**, which was directly used in enzyme assays.

### 3.6 Fluorination at Positions 2 and 3

#### 3.6.1 Difluorinated at Positions 2 and 3

##### 3.6.1.1 2,3-Difluorinated Glucose Derivatives

**Scheme 30:**
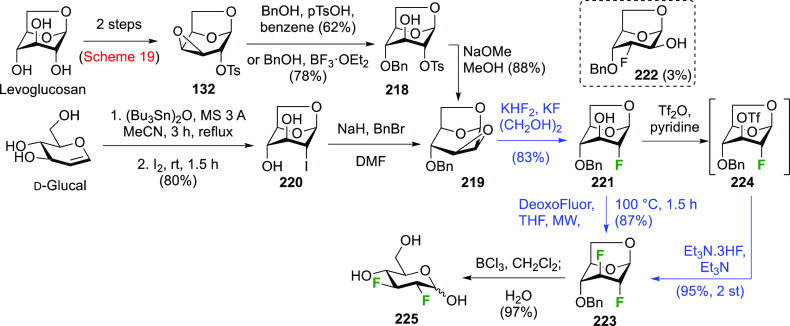
Synthesis of 2,3-Dideoxy-2,3-difluoro-d-glucose^215,265^

The synthesis of 2,3-dideoxy-2,3-difluoroglucose **225** was first reported by the Linclau group,^265^ followed by a Giguère synthesis featuring an improved deoxyfluorination procedure.^215^ Both syntheses involve the Cerny epoxide **219** as a key intermediate (Scheme 30). This can be synthesized either from levoglucosan in four steps, via **132**,^219,221,222,227^ or starting from glucal in three steps.^266−269^ Regioselective fluoride opening of the epoxide **219** with potassium hydrogen difluoride in ethylene glycol led to **221** in yields of 65 to 74%,^265,270,271^ but an improved 83% yield was obtained with the addition of KF as an extra fluoride source.^272^ In the original report of this epoxide opening, the formation of the regioisomer **222** in 3% yield was detailed.^271^ Fluorination of **221** at the 3-position using DAST proceeded with retention of configuration to give **223**, thanks to a neighboring group participation involving the 4-*O*-benzyl group. The original conditions involving refluxing a DAST solution in toluene^58^ gave a yield of 86%.^265^ Safer conditions involving DeoxoFluor in THF at 100 °C for 1.5 h under microwave irradiation gave a similar yield (87%),^273^ and a subsequent improvement by first obtaining the triflate intermediate **224** followed by displacement using TREAT-HF in triethyamine gave a 95% yield over two steps.^272^ This was followed by 1,6-anhydro-bridge opening and debenzylation with BCl_3_ in water to give 2,3-dideoxy-2,3-difluoro-glucose **225**.^215,265^

##### 3.6.1.2 2,3-Difluorinated Galactose Derivatives

**Scheme 31:**
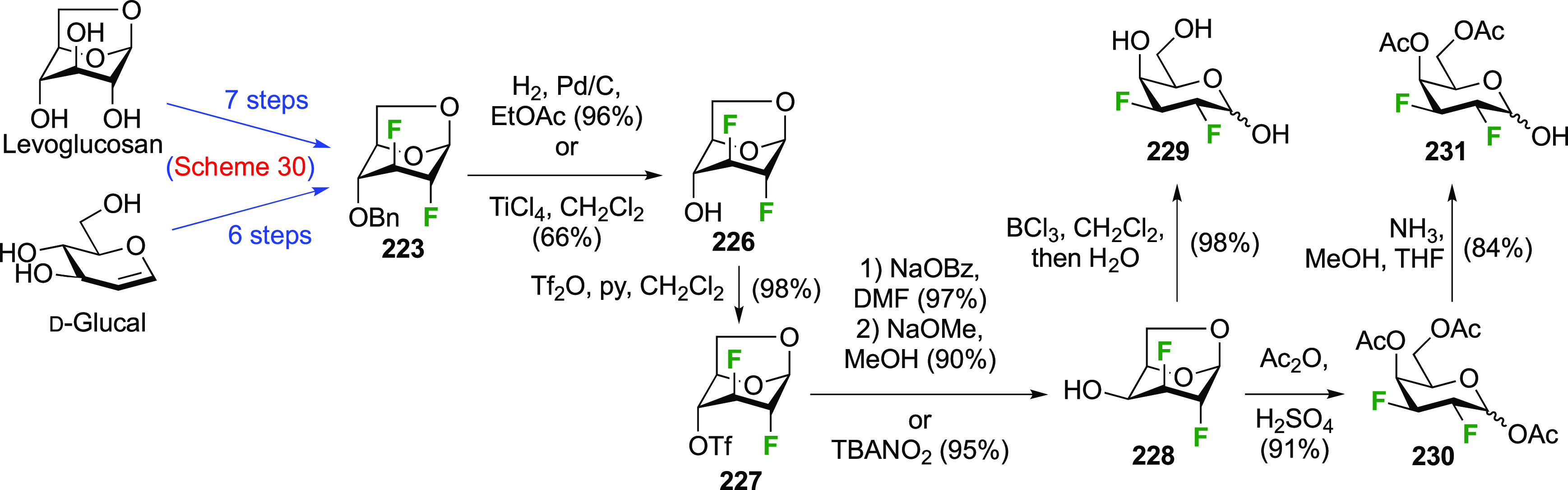
Synthesis of 2,3-Dideoxy-2,3-difluoro-d-galactose^274^

The synthesis of the corresponding 2,3-difluorinated galactose analogue **229** was reported by the Linclau group from the advanced intermediate **223** (Scheme 31).^274^ Deprotection of **223** leads to **226** using hydrogenolysis.^58^ While these conditions result in an excellent yield,^58^ they can be difficult to reproduce. The Giguère group showed that alternative deprotection conditions involving TiCl_4_ were effective as well.^275^ Inversion of the OH-4 by triflation, nucleophilic substitution with benzoate, and transesterification provides **228** in excellent yield.^58^ The inversion was also shown via a Lattrell-Dax reaction^276^ in equally excellent yield.^275^ Opening of the 1,6-anhydro-bridge with BCl_3_ led to 2,3-dideoxy-2,3-difluoro-d-galactose **229**, and with Ac_2_O in H_2_SO_4_ to the corresponding peracetylated **230**.^274^ The latter could be fully deprotected to give **229** (not shown), or selectively deprotected at the anomeric position to give **231**.

**Scheme 32:**
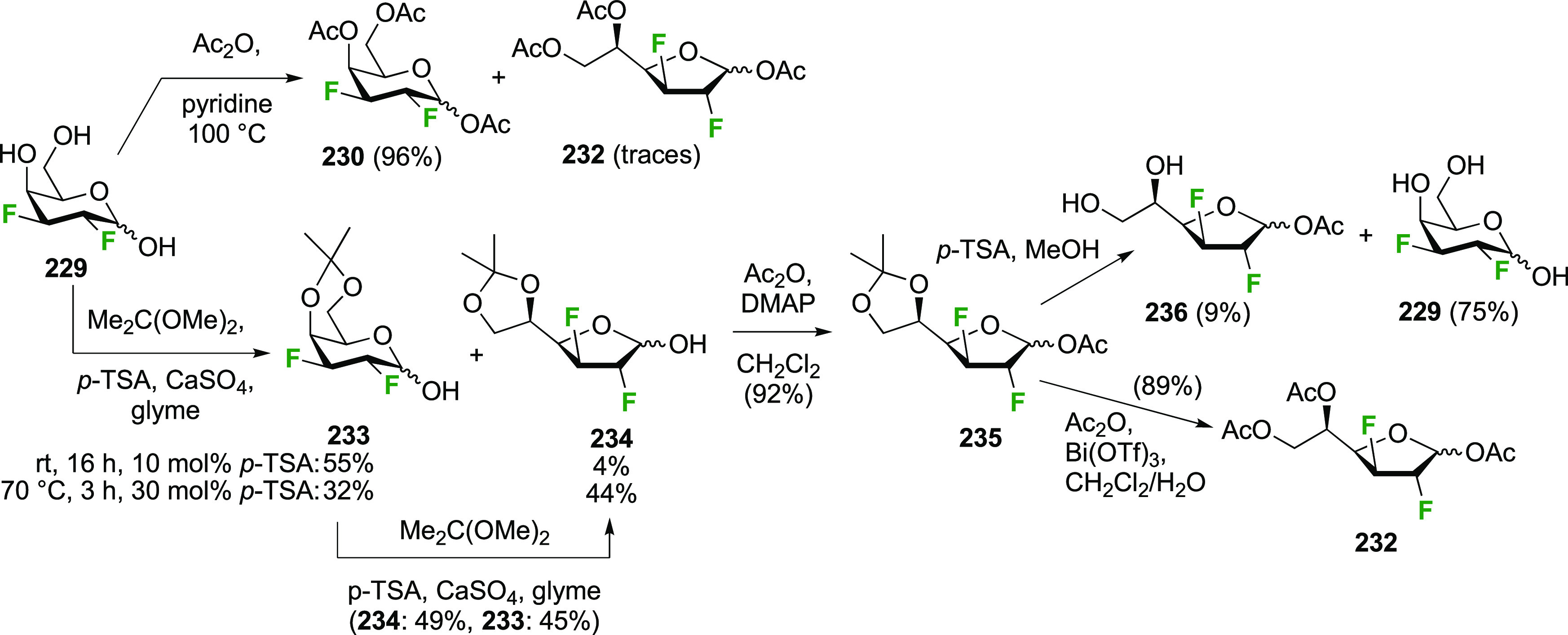
Protection of 2,3-Dideoxy-2,3-difluoro-d-galactose to Obtain the Furanose Form^274^

Given the importance of galactofuranoses,^277−279^ suitable protection of **229** to achieve ring isomerization was also investigated (Scheme 32).^274^ In contrast to a precedent from the Liu group, who showed that acetylation of 2-deoxy-2-fluorogalactose in pyridine at 100 °C gave a 1.6:1 ratio of pyranose to furanose (not shown),^280^ submitting **229** to these conditions only furnished traces of the furanose **232**. However, in accordance with precedent from Hricovíniová of protection of galactose with 2,2-dimethoxypropane,^281^**229** could be converted to the furanose acetonide **234**, which was the thermodynamically more stable acetonide as shown by the isomerization of **233** to **234**. After anomeric protection as acetate **235**, however, acetonide hydrolysis conditions caused anomeric deprotection, initiating ring isomerization back to the pyranose **229**. Finally, it was discovered that direct acetylation of **235** with bismuth triflate as catalyst did not lead to ring isomerization, and the furanose triacetate **232** was obtained as a suitable precursor for glycosylation reactions.

##### 3.6.1.3 2,3-Difluorinated Mannose Derivatives

**Scheme 33:**
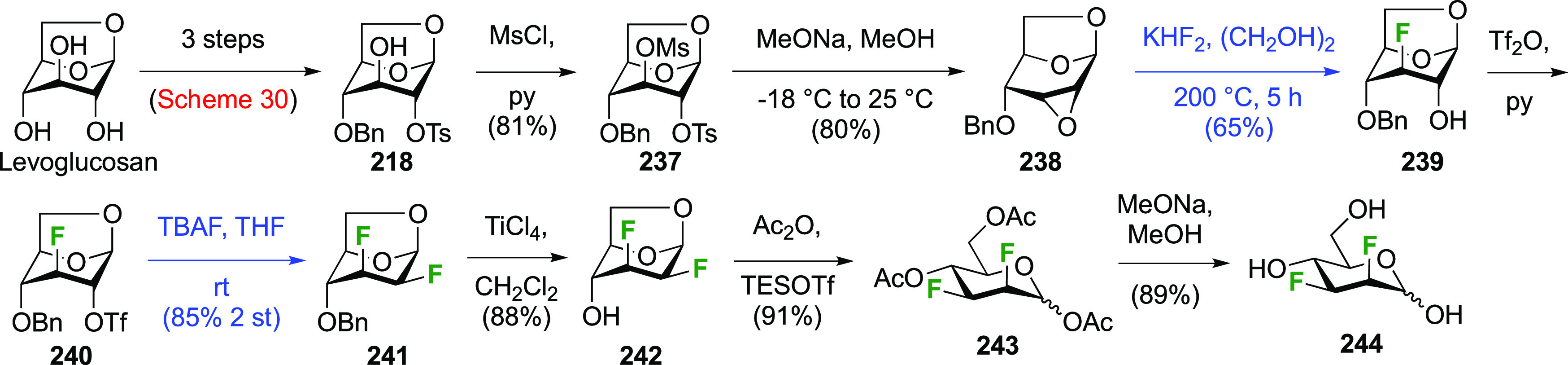
Synthesis of 2,3-Dideoxy-2,3-difluoro-d-mannose^282^

A synthesis of the 2,3-difluorinated mannose derivative **244** (Scheme 33) was reported by the Giguère group, with epoxide **238** as the key intermediate.^282^ This epoxide was synthesized via advanced intermediate **218**, which was obtained in three steps from levoglucosan,^221^ via mesylation and then slow addition of NaOMe, which significantly improved the yield of this reaction.^221,283^ The methoxide reacts with the 2-OTs group at the sulfur atom to generate the corresponding alkoxide, which then displaces the 3-OMs group to form the epoxide. This reaction was further improved by the use of dichloromethane as the solvent instead of chloroform.^227^ The epoxide **238** is then regioselectively opened with KHF_2_ to give **239**, after which the OH-2 group is activated to the triflate **240** and displaced by fluoride to give **241**.^275^ Deprotection, acetolysis, and acetate methanolysis then gave 2,3-dideoxy-2,3-difluoro-d-mannose **244**.^282^

##### 3.6.1.4 2,3-Difluorinated Talose Derivatives

**Scheme 34:**
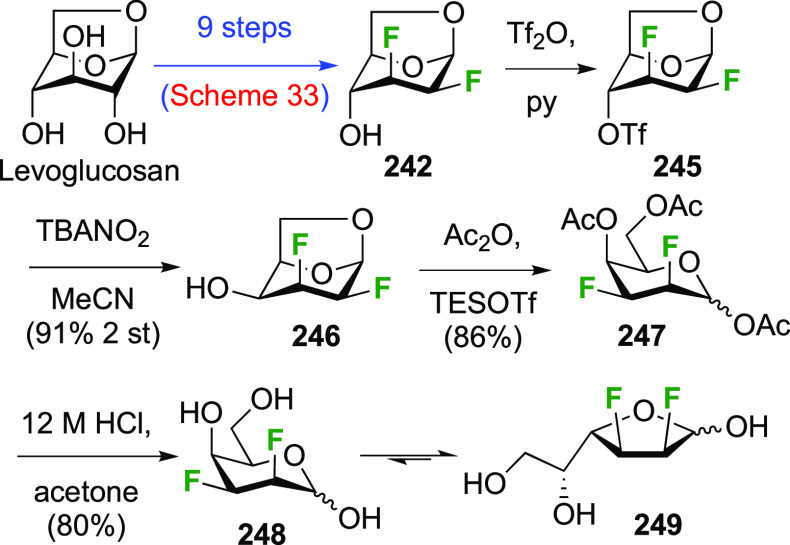
Synthesis of 2,3-Dideoxy-2,3-difluoro-d-talose^282^

The advanced intermediate **242** (cf. Scheme 33) was also used for the synthesis of the 2,3-difluorinated talose **248** (Scheme 34). Lattrell-Dax inversion^276^ at C-4 to give **246**(275) was followed by 1,6-anhydro-bridge acetolysis to **247**, then acetate hydrolysis to give 2,3-dideoxy-2,3-difluoro-d-talose as a mixture of pyranose and furanose tautomers **248** and **249**.^282^

**Scheme 35:**
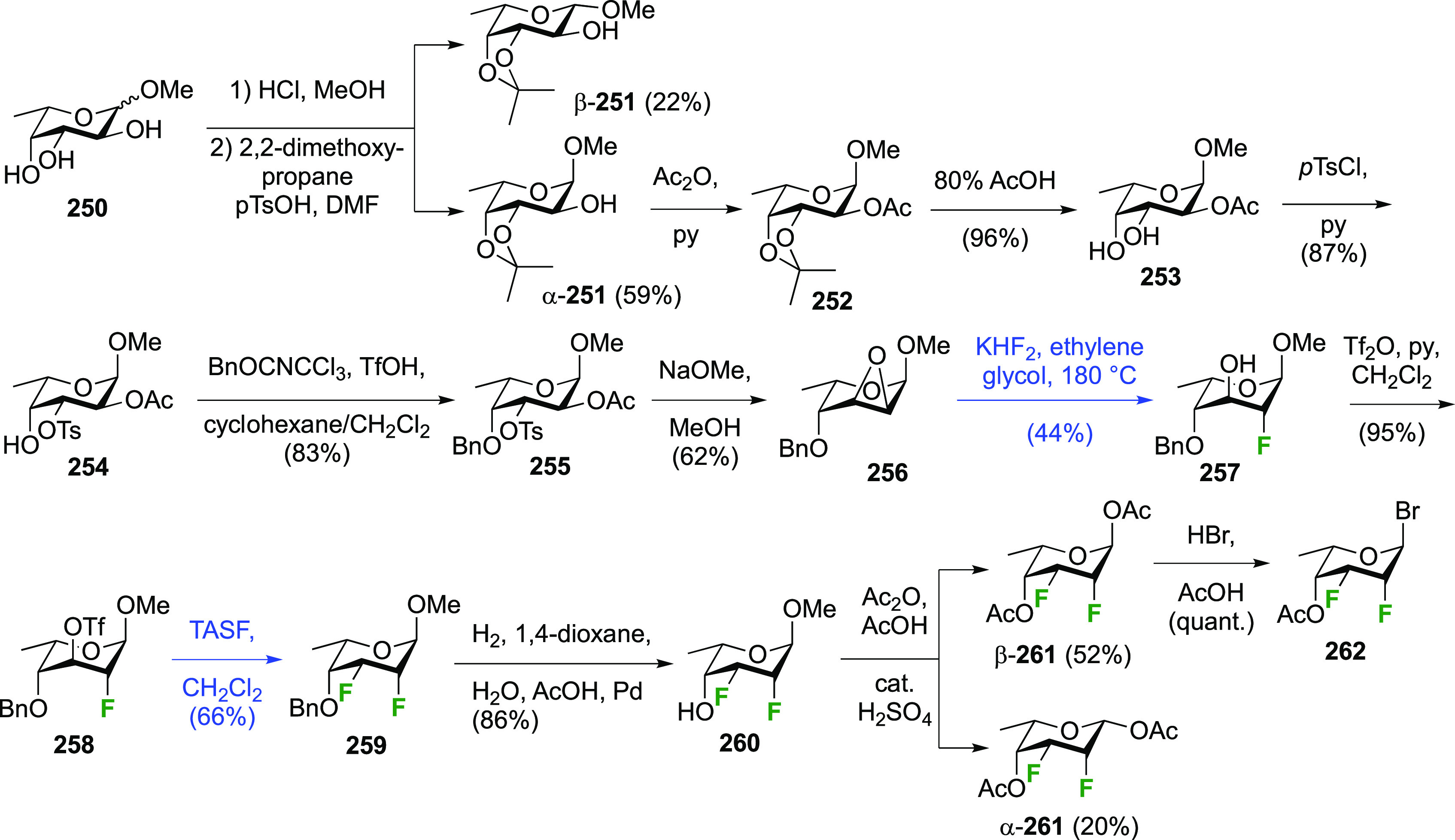
Synthesis of 2,3,6-Trideoxy-2,3-difluoro-α-l-talopyranosyl Bromide^284^

The synthesis of 4-*O*-acetyl-2,3,6-trideoxy-2,3-difluoro-α-l-talopyranose bromide **262** was described by the Tsuchiya group, starting from methyl α-l-fucopyranoside **250** (Scheme 35).^284^ After selective protection as its acetonide, which enabled separation of the anomers of **251**,^285^ the α-anomer was used to continue the synthesis. Position 2 was acetylated using a standard procedure,^286^ giving compound **252**, and the acetonide was subsequently hydrolyzed to give **253**. Selective tosylation at the equatorial position and benzylation of position 4 gave **255**, upon which treatment with sodium methoxide in methanol gave the l-gulo-2,3-epoxy derivative **256**. Fluorination of **256** was achieved with KHF_2_ in ethylene glycol by selective epoxide opening, giving the 2-fluoro-l-idopyranose derivative **257**.^285^ Deoxyfluorination of **257** using DAST led to a complex mixture, but activation of position 3 as triflate **258** followed by nucleophilic substitution using tris(dimethylamino)sulfonium difluorotrimethylsilicate (TASF) as the fluoride source successfully led to 2,3,6-trideoxy-2,3-difuoro-α-l-talopyranoside **259**.^284^ Hydrogenolysis and acetolysis gave a mixture of α- and β-**261** (α/β ratio: 2.5:1), and bromination of α-**261** gave crystalline 2,3,6-trideoxy-2,3-difluoro-α-l-talopyranosyl bromide **262**.

#### 3.6.2 Tetrafluorinated at Positions 2 and 3

**Scheme 36:**
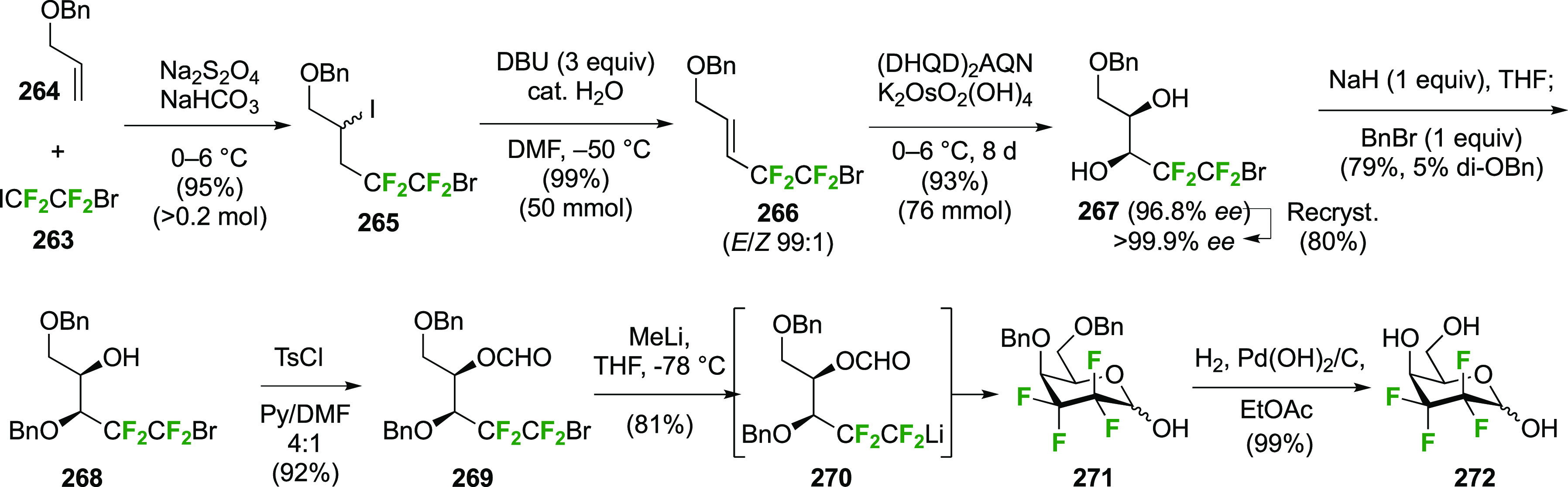
Synthesis of 2,3-Dideoxy-2,2,3,3-tetrafluoro-d-*threo*-hexopyranose^287,288^

The synthesis of 2,3-dideoxy-2,2,3,3-tetrafluorinated sugar derivatives was reported by Linclau et al. using a *de novo* approach, starting from commercially available fluorinated building block **263** (Scheme 36).^287,288^ Radical abstraction of iodine from **263** by a single electron transfer initiated by dithionite homolysis led to addition to alkene **264**, which resulted in **265** after the atom transfer propagation step. Elimination at low temperature with wet DMF led to the alkene **266** in excellent yield and stereoselectivity. A Sharpless asymmetric dihydroxylation reaction gave the *syn*-diol **267** in excellent yield. Due to the alkene deactivation by the fluorination,^289^ increased levels of osmium and ligand were required, but the ligand could be easily recovered.^290^ Subsequent recrystallization led to essentially enantiopure material. Selective protection was achieved by deprotonation of the most acidic alcohol group of **267** followed by benzylation, leading to **268** with minimal diprotection (5%, not shown). Formylation then gave the cyclization precursor **269**, which upon bromine–lithium exchange to **270** allowed cyclization to the protected tetrafluorinated sugar derivative **271**. Hydrogenolysis then led to 2,3-dideoxy-2,2,3,3-tetrafluoro-d-*threo*-hexopyranose **272**.^287,288^

**Scheme 37:**
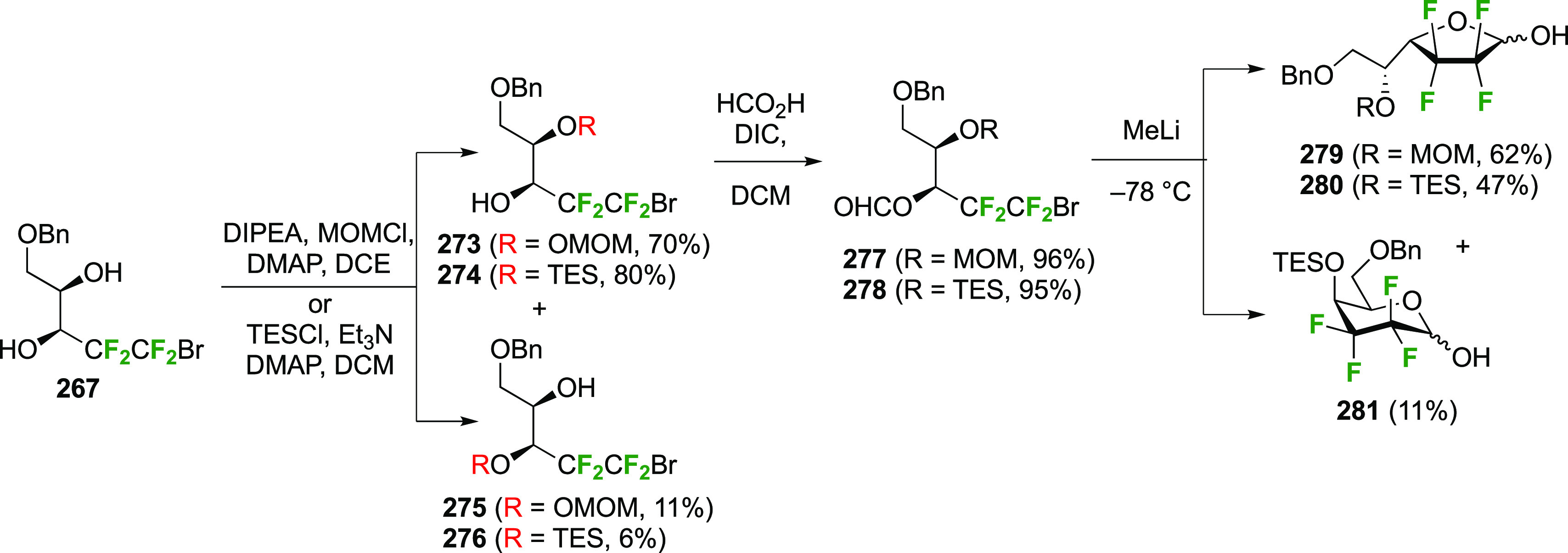
Synthesis of 2,3-Dideoxy-2,2,3,3-tetrafluoro-d-*threo*-hexofuranose Derivatives^288,291^

Protection of the other alcohol group in **267** allows synthesis of furanose derivatives.^288,291^ Reaction with MOM-Cl (Scheme 37) gave **273** as the major regiosiomer in 70% yield, as a result of the most nucleophilic alcohol group reacting in preference. With TESCl as the electrophile, **274** was obtained in 80% yield. The regioisomers **275** and **276** were obtained in 11% and 6% yields, respectively, and both reactions also returned fully protected product (5% with MOM, 10% with TES, not shown). Formylation and bromine–lithium exchange then allowed cyclization to give **279** and **280** depending on the protection.^288,291^ Interestingly, with TES protection a rearrangement took place to give the more stable pyranose analogue **281** as a side product.^288^

**Scheme 38:**
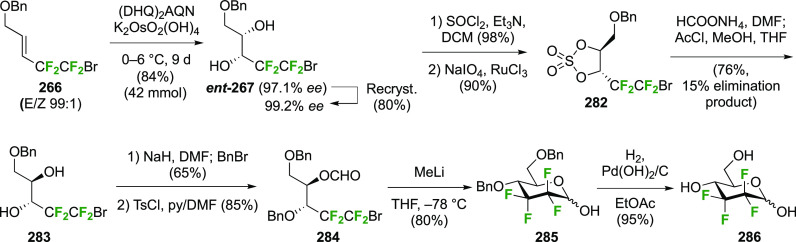
Completion of the Synthesis of 2,3-Dideoxy-2,2,3,3-tetrafluoro-d-*erythro*-hexo-pyranose (“Tetrafluorinated Glucose”)^287,288^

The corresponding *gluco*-configured diastereomer **286** was also synthesized from **266** (Scheme 38) and required inversion of configuration of one of the alcohols. Because adjacent fluorination hampers the S_N_2 reactions,^143,164−166^ inversion of the C-5 (sugar numbering) alcohol was targeted. This required carrying out the asymmetric dihydroxylation using the pseudoenantiomeric ligand and was achieved in similar yield and enantioselectivity, with recrystallization leading to highly enantioenriched *ent*-**267**. Inversion at C-5 was successfully achieved via a cyclic sulfate intermediate **282** in good yield, with 15% of a separable side-product arising from competing elimination (not shown). From **283**, **286** was obtained via the same four steps as described in Scheme 36.^287,288^

**Scheme 39:**
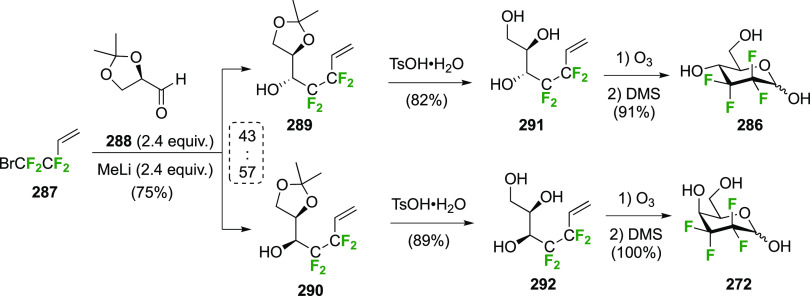
A Shorter Synthesis of 2,3-Dideoxy-2,2,3,3-tetrafluoro-d-*erythro*-hexo-pyranose (“Tetrafluorinated Glucose”) 2,3-Dideoxy-2,2,3,3-tetrafluoro-d-*threo*-hexo-pyranose (“Tetrafluorinated Galactose”)^292^

Perfluoroalkyl lithium species are unstable and rapidly lead to fluoride elimination. Clearly, the rate of cyclization toward **271** and **285** outstrips the rate of elimination. Konno and co-workers even achieved an intermolecular addition of a lithiated **287** to the protected glyceraldehyde **288** under Barbier conditions (Scheme 39),^292^ which allows a shorter sequence to **272** and especially **286**. A key condition was that LiBr-free MeLi was used. This intermolecular reaction afforded a 43:57 mixture of coupling adducts **289** and **290**, which were separated by column chromatography.^293^ Subsequently, acid catalyzed deprotection yielded triols **291** and **292**, which following ozonolysis spontaneously cyclized to afford the target sugars **286** and **272**. Despite only being described on a small scale, this three-step sequence constitutes the most efficient route to tetrafluoro glucose **286** and galactose **272**, obtained in 24% and 38% overall yields, respectively. However, a large excess of MeLi and aldehyde **288** is required (2.4 equiv each) due to competing addition of MeLi to the electrophilic aldehyde **288**.

**Scheme 40:**
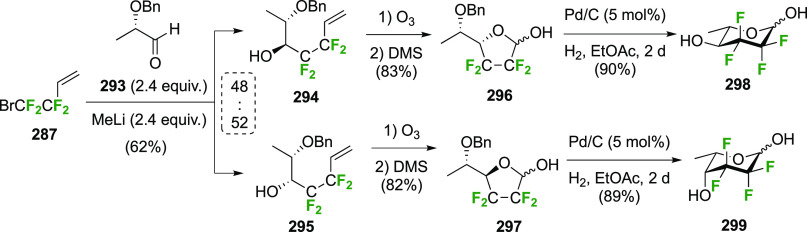
Synthesis of 2,3,6-Trideoxy-2,2,3,3-tetrafluoro-d-*erythro*-hexo-pyranose (“Tetrafluorinated Quinovose”) 2,3,6-Trideoxy-2,2,3,3-tetrafluoro-d-*threo*-hexo-pyranose (“Tetrafluorinated Fucose”)^292^

This methodology was further extended by the same group to the corresponding 6-deoxygenated sugar derivatives **298** and **299** (Scheme 40) using the ethyl lactate derived **293** as the electrophile. Chromatographic separation of the resulting diastereomers **294** and **295**, followed by ozonolysis and hydrogenolysis, led to **298** and **299** in excellent yields.

### 3.7 Fluorination at Positions 2 and 4

#### 3.7.1 Difluorinated at Positions 2 and 4

##### 3.7.1.1 2,4-Difluorinated Glucose Derivatives

**Scheme 41:**
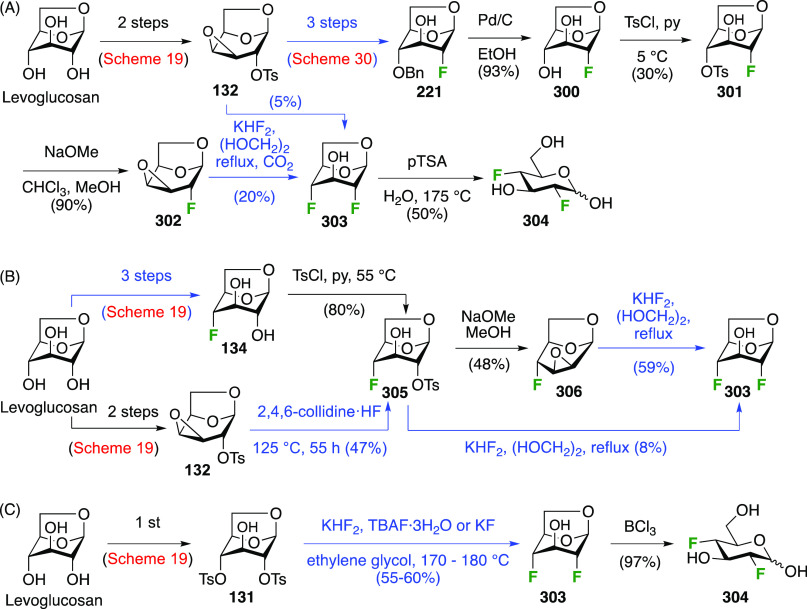
Approaches to 2,4-Dideoxy-2,4-difluorinated Glucose^215,217,271,297^

The first synthesis of 2,4-dideoxy-2,4-difluoroglucose was reported by the Cerny group (Scheme 41A).^271^ Starting from **221**, obtained from levoglucosan as discussed in Scheme 30, hydrogenolysis and selective tosylation afforded **301**, whereupon the second fluoride was installed after another epoxide introduction. This led to **303** in modest yield. Interestingly, the two fluorine atoms could be introduced directly from **132**,^219,220^ albeit in low yield (5%), which nevertheless is higher than the overall yield via **302**. Acid-catalyzed hydrolysis then gave the target sugar **304**.

Another synthesis of the pivotal 2,4-difluorinated intermediate **303** was published around the same time by Barford et al.,^217^ also starting from levoglucosan (Scheme 41B), but with the first fluorine introduction at C-4 involving intermediate **134** as described in Scheme 19. This was tosylated to give **305** and subsequent treatment with base gave epoxide **306**. This was then opened with fluoride to give **303** in 59% yield. Direct treatment of **305** with KHF_2_ in boiling ethylene glycol, which relied on in situ epoxide formation, only proceeded to give **303** in 8% yield. A shorter synthesis of **305** was later published by the Voznyi group by opening of the epoxide **132** with 2,4,6-collidine·HF.^294^

A good-yielding direct synthesis of **303** has only been recently achieved by the Giguère group,^275,295^ and then by our group,^296^ from the easily accessible ditosylate **131** (Scheme 41C). Using this procedure, Giguère achieved three-step synthesis of **304** in excellent yield via opening of the 1,6-anhydro-bridge in **303** with a strong Lewis acid.^215^

##### 3.7.1.2 2,4-Difluorinated Allose Derivatives

**Scheme 42:**
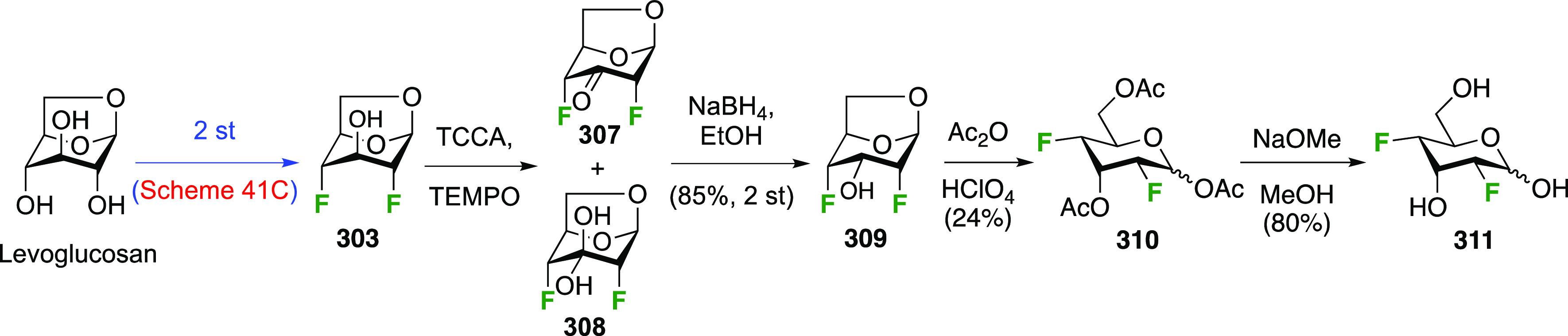
Synthesis of 2,4-Dideoxy-2,4-difluoroallose^298^

A 2,4-difluorinated allose derivative was also prepared from **303** by the Cerny group (Scheme 42).^298^ The required C-3 alcohol inversion was achieved by oxidation to give the ketone as the hydrate **308**, followed by NaBH_4_ reduction. The original oxidation conditions involved the use of CrO_3_, but the oxidation was found to work with TCCA/TEMPO,^296^ or with DMP as well.^215^ Ring opening of the thus obtained allose derivative **309** was not possible using 1% aq. pTSA, but acetolysis under perchloric acid did give triacetate **310**, albeit in low yield. Deacetylation then gave the free 2,4-dideoxy-2,4-difluoroallose **311**.^298^

##### 3.7.1.3 2,4-Difluorinated Talose Derivatives

**Scheme 43:**
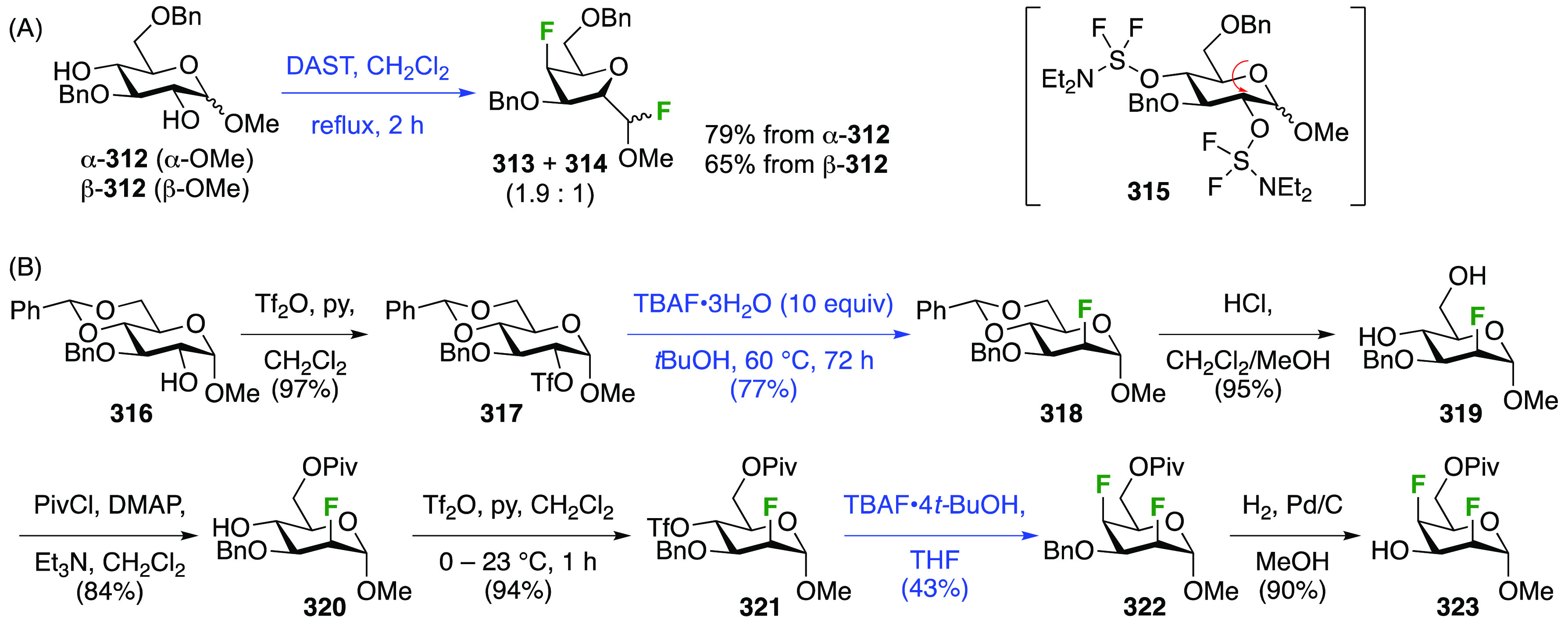
Synthesis of a 2,4-Dideoxy-2,4-difluorotalose Derivative^299,303^

The synthesis of 2,4-dideoxy-2,4-difluorotalose is possible from glucose through deoxyfluorination with inversion of configuration at C-2 and C-4. The Cabrera-Escribano group described the treatment of both anomers of methyl 3,6-di*-O*-benzyl glucopyranoside **312** with DAST (Scheme 43A) to achieve exactly that.^299^ However, while deoxyfluorination was observed at the 4-position, a ring contraction occurred at the 2-position to give epimers **313** and **314**, regardless of the anomeric configuration. A ring contraction had not been reported by the Somawardhana and Card groups in their deoxyfluorination experiments of unprotected methyl glucosides.^300−302^ Presumably the presence of a benzyl group in **312**, which is less electron withdrawing compared to an alcohol group activated by DAST, allows the intramolecular S_N_2 reaction by the endocyclic oxygen at C-2 (cf. **315**), with deoxyfluorination at C-4 likely to take place after the furanose ring is obtained (see later Scheme 144).

The Gouverneur group did achieve a synthesis of a 2,4-difluorinated talose derivative by a sequential fluorination method starting from known **316** (Scheme 43B),^303^ synthesized in two steps from methyl α-d-glucoside (not shown).^304^ Fluorination of **316** at the 2-position by displacement of the corresponding triflate **317** had been described.^305^ However, while this S_N_2 reaction using TBAF·3H_2_O in acetonitrile proceeds well with the β-anomer, a low 30% yield had been reported for the desired α-anomer. Extensive optimization led to a significantly improved yield of **318** (77%), by in situ conversion of TBAF·3H_2_O to TBAF·(*t*-BuOH)_4_ via stirring in *t*-BuOH in the presence of **317**. Because selective benzylidene acetal reduction in **318** was not successful, it was hydrolyzed and the OH-6 was selectively protected as the pivaloate ester **320** (11% of diester). Having established that **320** did not react with DAST, no doubt because the axial F-2 group prevented S_N_2 reaction with an equatorial C-4 leaving group, a second triflation with subsequent fluoride displacement was carried out, giving **322** with a 43% yield for the displacement step. The difficult substitution also led to the isolation of desulfonylated product **320** (8%), starting material **321** (13%), and a range of other byproducts (not shown). Finally, hydrogenolysis gave **323**, which was used in intramolecular hydrogen-bond studies.^306^

#### 3.7.2 Trifluorination at Positions 2 and 4

**Scheme 44:**
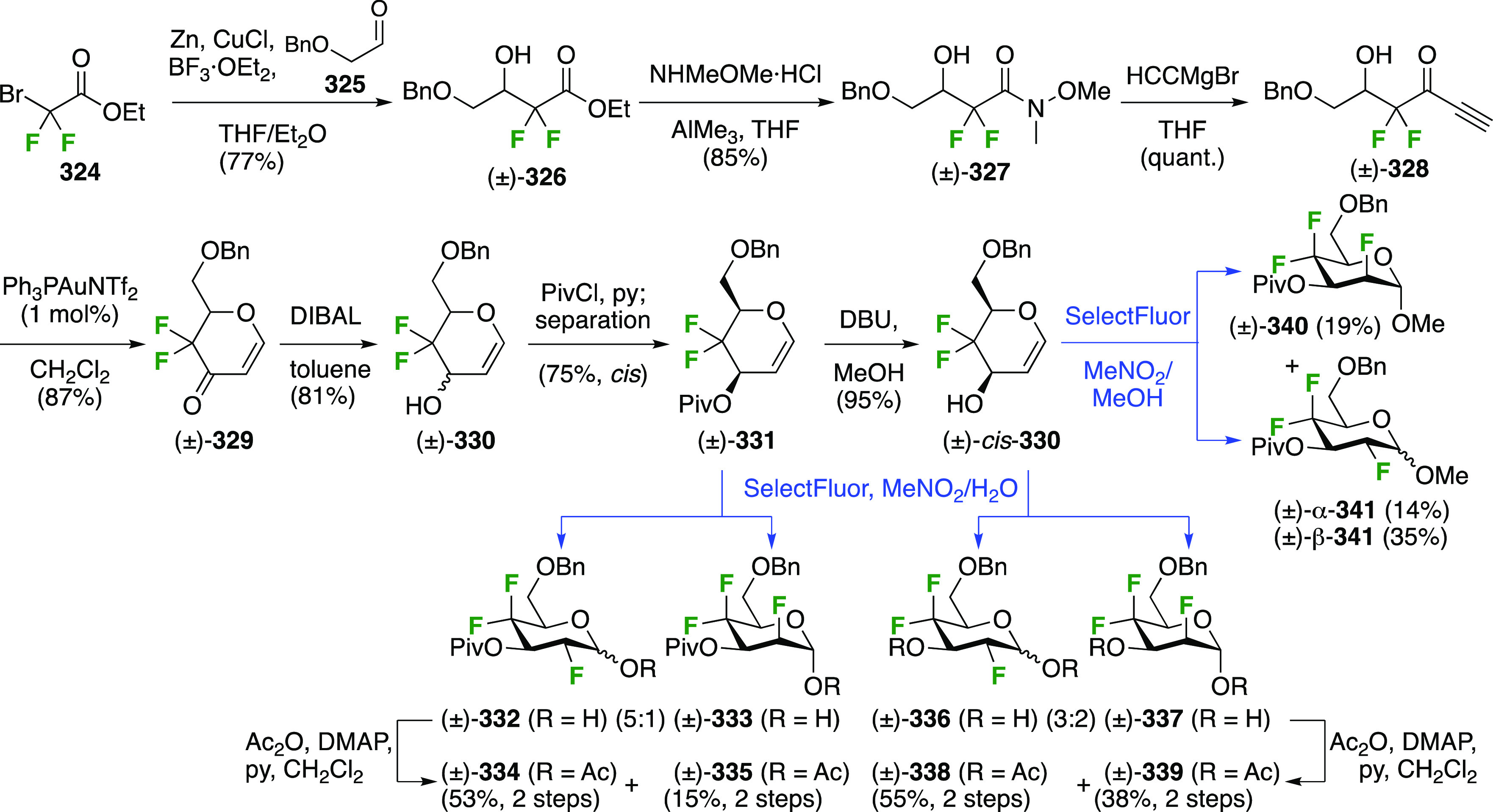
Synthesis of Racemic 2,4-Dideoxy-2,4,4-trifluorinated Sugar Derivatives^307^

The Gouverneur group also synthesized racemic 2,4-dideoxy trifluorinated sugar derivatives (Scheme 44),^307^ using a *de novo* synthesis approach starting from a difluorinated building block **324**, featuring a 6-*endo*-*dig* gold-catalyzed ring formation. A Reformatski reaction with (benzyloxy)acetaldehyde **325** led to racemic **326**, upon which alkyne introduction via the corresponding Weinreb amide intermediate (±)-**327** gave the cyclization precursor (±)-**328**. A high-yielding ring formation was achieved with the Gagosz catalyst, with the obtained dihydropyran ring (±)-**329** nicely set up for electrophilic fluorine introduction at C-2. Reduction of the keto group to give (±)-**330** was moderately selective (83:13) in favor of the desired C-3 configuration, and the diastereomers could be separated after pivaloyl protection. Due to the deactivation by the fluorine atoms, reaction of the glycals with SelectFluor required heating, but excellent yields were obtained. Starting from the pivaloate (±)-**331**, a 5:1 ratio of *gluco*:*manno* stereochemistry (±)-**332**, (±)-**333** was obtained, separable after acetylation to (±)-**334** and (±)-**335**. From (±)-*cis*-**330**, in which the 3-*O*-pivaloyl group was removed, the *gluco*:*manno* ((±)-**336** and (±)-**337**) ratio was reduced to 3:2, with a better overall yield. Acetylation to (±)-**338**/(±)-**339** again allowed separation. In both cases, (±)-**335** and (±)-**339** were obtained as α-anomers. When the reaction was conducted in nitromethane/methanol the corresponding methyl acetals (±)-**340** and (±)-**341** were formed directly, and were separable by chromatography.

### 3.8 Fluorination at Positions 2 and 5

There is no reported synthesis available of a 5-fluorinated 2-deoxyfluorinated hexose sugar, although a 2,5-difluorinated glucose derivative has been obtained as a byproduct in the synthesis of 2-deoxy-1,2-difluoroglucopyranosyl fluoride **4** (Scheme 16).

### 3.9 Fluorination at Positions 2 and 6

#### 3.9.1 Difluorinated at Positions 2 and 6

##### 3.9.1.1 2,6-Difluorinated Glucose Derivatives

**Scheme 45:**
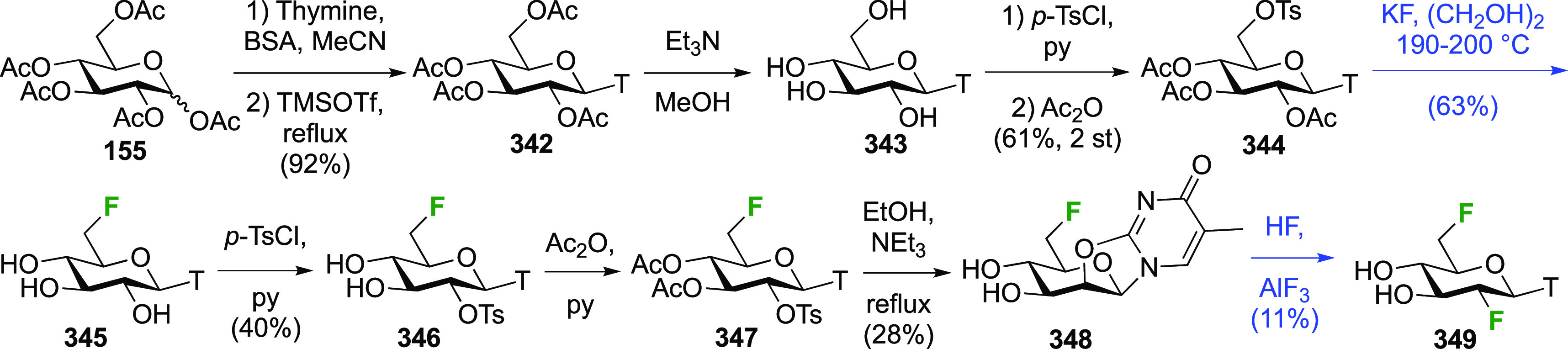
Synthesis of 1-(2′,6′-Dideoxy-2′,6′-difluoroglucosyl)thymine^308^

The first 2,6-difluorinated glucose derivative, nucleoside analogue **349**, was reported in 1978 by Etzold et al.^308^ The synthesis started from 1-glucosyl thymine **343** (Scheme 45), which can be obtained from glucose peracetate **155** in two steps.^309,310^ Tosylation was only moderately selective at the 6-position, and the mixture was acetylated to then separate the 2,6-ditosylated byproduct. This gave **344** in 61% yield.^310^ Fluorination with KF in ethylene glycol at high temperature was described to give the deacetylated 6-fluoroderivative **345**. Position 2 could now be activated as the *p*-toluenesulfonylate, giving **346** in 40% yield. After acetylation to give **347**, reaction with triethylamine in ethanol with inversion of configuration at C-2 gave the 2,2′-anhydro-nucleoside **348**. Reaction with HF under AlF_3_-catalysis in dioxane in a steel reactor gave the desired 2,6-dideoxy-2,6-difluoro glucopyranose **349** in 11% yield.^308,310^

**Scheme 46:**
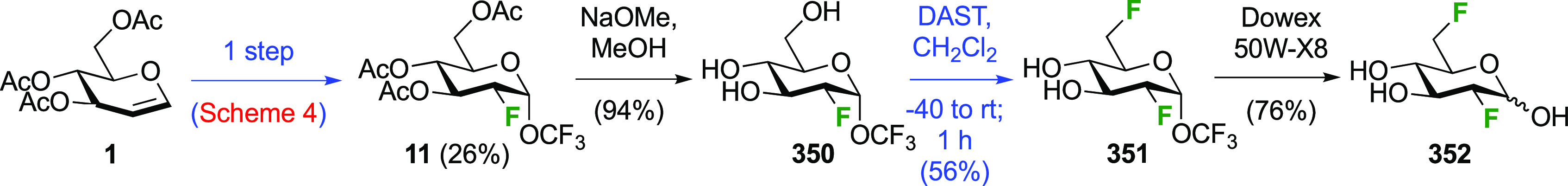
Synthesis of 2,6-Dideoxy-2,6-difluoro-d-glucopyranose^311^

The first synthesis of the free 2,6-dideoxy-2,6-difluoro-d-glucose sugar **352** was reported by Withers (Scheme 46),^311^ starting from trifluoromethyl 3,4,6-tri-*O*-acetyl-2-deoxy-2-fluoro-α-d-glucopyranose **11**, which was obtained from 3,4,6-tri-*O*-acetyl-d-glucal **1** by reaction with fluoroxytrifluoromethane (cf. Scheme 4).^157^ Deacetylation of **11** with NaOMe in MeOH to **350** allowed a selective reaction with DAST at the primary alcohol to give **351**. Acid-catalyzed hydrolysis then gave 2,6-dideoxy-2,6-difluoro-d-glucopyranose **352** in 76% yield.^311^

**Scheme 47:**
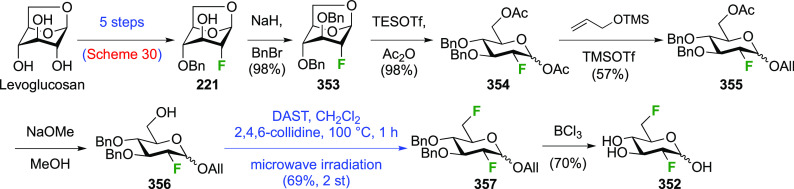
Synthesis of 2,6-Dideoxy-2,6-difluoro-d-glucose^215^

A synthesis of **352** avoiding the use of CF_3_OF was reported by Giguère et al. (Scheme 47).^215^ Intermediate **221**, obtained in five steps as discussed above (Scheme 30), was fully benzylated to give **353**. Anhydro-bridge acetolysis without benzyl removal led to **354** in 98% yield. Differentiation of the acetate groups was achieved by glycosidation to give **355**, after which acetyl deprotection allowed deoxyfluorination at C-6 with DAST. This afforded compound **357** without any observation of 3,6-anhydro side-product formation (cf. Schemes 26 and 28). In addition to the presence of base, presumably the configuration of the F-2 group, which will be antiperiplanar with the C-3 OBn bond in the ^1^*C*_4_ conformation required for 3,6-anhydro formation, will have deactivated the O3 for nucleophilic attack at the activated OH-6 group. Deprotection of **357** then led to 2,6-dideoxy-2,6-difluoro-d-glucose **352**.

##### 3.9.1.2 2,6-Difluorinated Galactose Derivatives

**Scheme 48:**
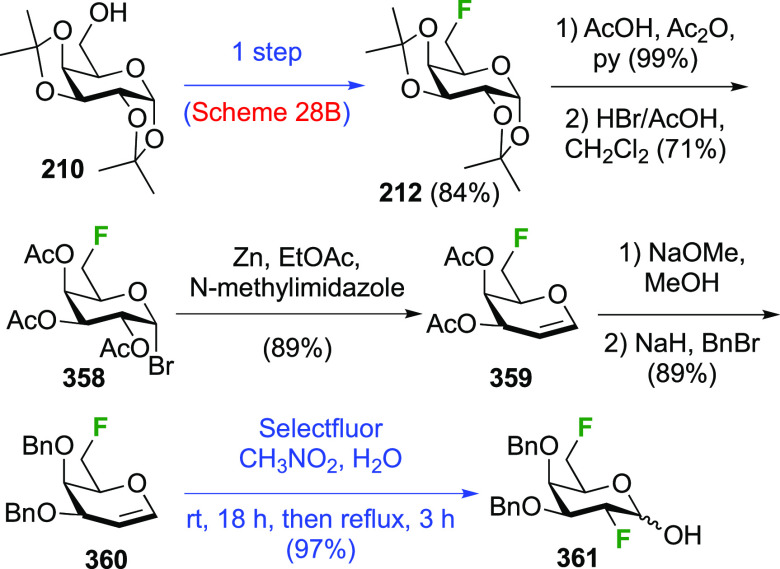
Synthesis of a 2,6-Dideoxy-2,6-difluorogalactopyranose Derivative^43^

In 2012, the Hoffmann-Röder group reported a synthesis of a protected 2,6-dideoxy-2,6-difluoro-d-galactopyranose **361** (Scheme 48).^43^ Starting from 1,2:3,4-di-*O*-isopropylidene galactose **210**, deoxyfluorination at C-6 (cf. Scheme 28B), acetolysis, and HBr treatment gave the corresponding galactopyranosyl bromide **358**.^196^ Zn-mediated reductive elimination of the 1-bromo and 2-acetoxy groups led to 6-deoxy-6-fluoro-galactal **359** in 89% yield. After an acetate-to-benzyl protecting group switch, electrophilic fluorination using SelectFluor in aqueous medium afforded 3,4-di-*O*-benzyl-2,6-dideoxy-2,6-difluoro-d-galactose **361** in 97% yield.^43^

##### 3.9.1.3 2,6-Difluorinated Fucose Derivatives

**Scheme 49:**
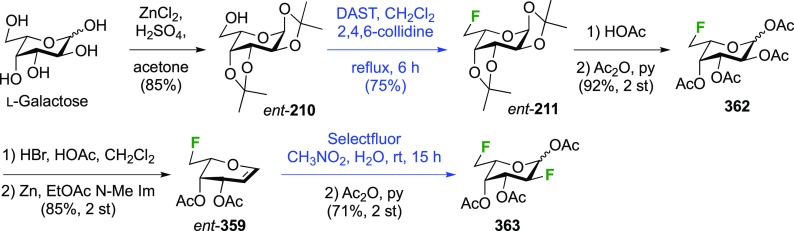
Synthesis of Peracetylated 2-Deoxy-2,6-difluorofucose^198^

This synthesis, published in 2020 by the Wang group (Scheme 49),^198^ is very similar to the 2,6-difluorinated galactose synthesis discussed above, but starting from l-galactose. Reaction with zinc chloride, sulfuric acid, and acetone gave 1,2:3,4-di-*O*-isopropylidene-α-l-galactopyranose *ent*-**210** in 85% yield. Deoxyfluorination of *ent*-**211** with DAST in the presence of 2,4,6-collidine according to Hoffmann-Röder’s procedure (cf. Scheme 28B),^196^ but in refluxing dichloromethane as opposed to under microwave conditions, led to *ent*-**211** in a slightly lower yield. Acid-catalyzed hydrolysis followed by acetylation gave the peracetylated 6-fluoro-l-fucose **362** in 92% yield over two steps. Treatment of **362** with hydrogen bromide and subsequent reductive elimination afforded 6-fluoro-l-fucal *ent*-**359**, which was subjected to SelectFluor, and protected to give peracetylated 2,6-difluoro-l-fucose **363**.^198^

##### 3.9.1.4 2,6-Difluorinated Mannose Derivatives

**Scheme 50:**
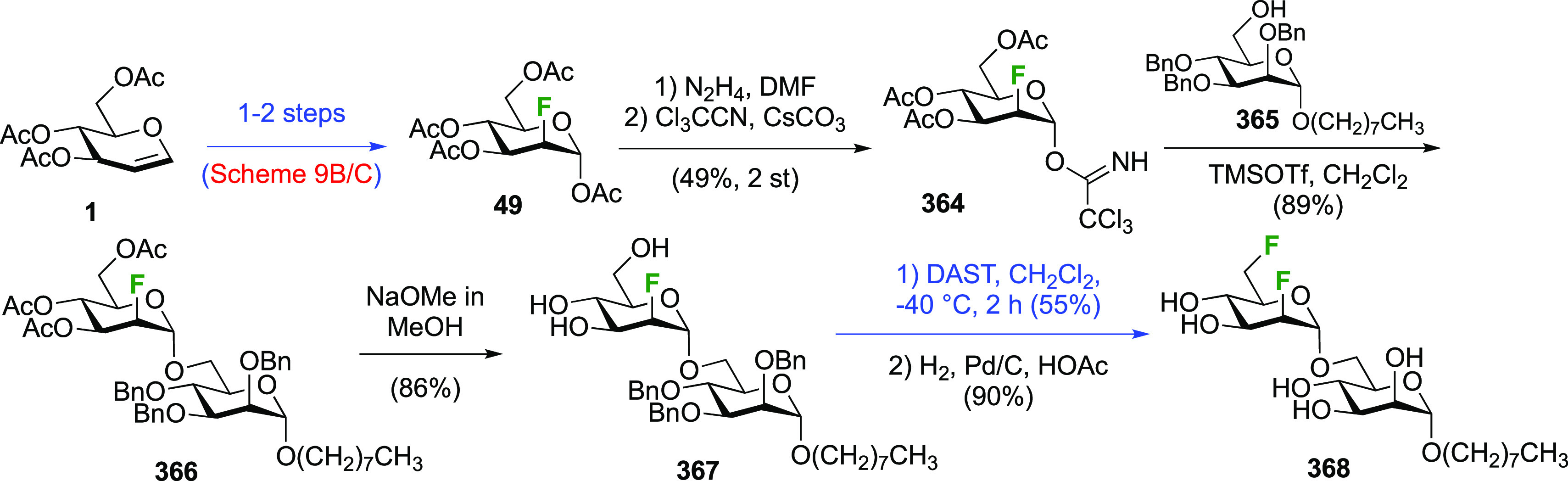
Synthesis of Octyl 2,6-Dideoxy-2,6-difluoro-α-d-mannopyranosyl-(1→6)-α-d-mannopyranoside^312^

The synthesis of a 2,6-difluorinated mannose derivative was reported by the Lowary group from 2-deoxy-2-fluoro-α-mannose peracetate **49** (Scheme 50),^312^ which is most efficiently prepared from 3,4,6-tri-*O*-acetylglucal **1** involving reaction with SelectFluor, followed by acetylation to achieve separation from the 2-fluoroglucose stereomers as shown in Scheme 16.^170,177^ Anomeric deprotection and activation as the trichloroacetimidate **364**(313) allowed mannosylation with acceptor **365** to give the monofluorinated disaccharide **366**.^314^ Deacetylation to **367** allowed selective fluorination at the 6′-position (with S_N_2 reaction at the 3- and 4- positions prevented by the axial C-1 and C-2 substituents, respectively) and, after benzyl hydrogenolysis, the 2′,6′-dideoxy-2′,6′-difluorinated dimannoside **368** was obtained.^312^

##### 3.9.1.5 2,6-Difluorinated Altrose Derivatives

**Scheme 51:**
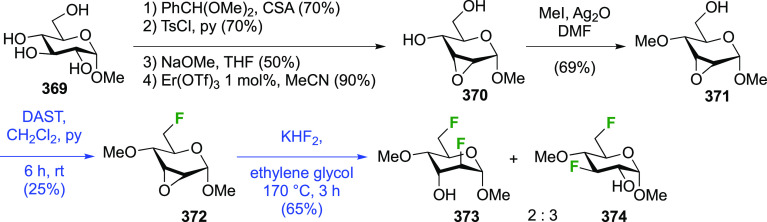
Synthesis of a 2,6-Dideoxy-2,6-difluoroaltrose Derivative^315^

Studies by the Tsuchiya group regarding the regioselective opening of 2,3-anhydroallopyranoside derivatives such as **372** led to the synthesis of 2,6-dideoxy-2,6-difluoroaltrose derivatives (Scheme 51).^315^ The epoxy intermediate **370**, which can be obtained from methyl glucoside **369** in four steps via selective OH-3 tosylation, base-mediated epoxide formation,^316^ and hydrolysis,^317^ was selectively methylated at the 4-position to give **371**.^315^ Its deoxyfluorination led to **372**, which was further fluorinated by reaction with KHF_2_ in ethylene glycol to give an inseparable mixture of the *altro*-derivative **373** and the *gluco*-derivative **374** in a 2:3 ratio.^315^ The surprisingly low ratio may be due to the electronic influence of the more electronegative anomeric center (cf. Scheme 95A below), disfavoring substitution at C-2 despite the chairlike conformation associated with the latter.

#### 3.9.2 Tetrafluorinated at Positions 2 and 6

**Scheme 52:**
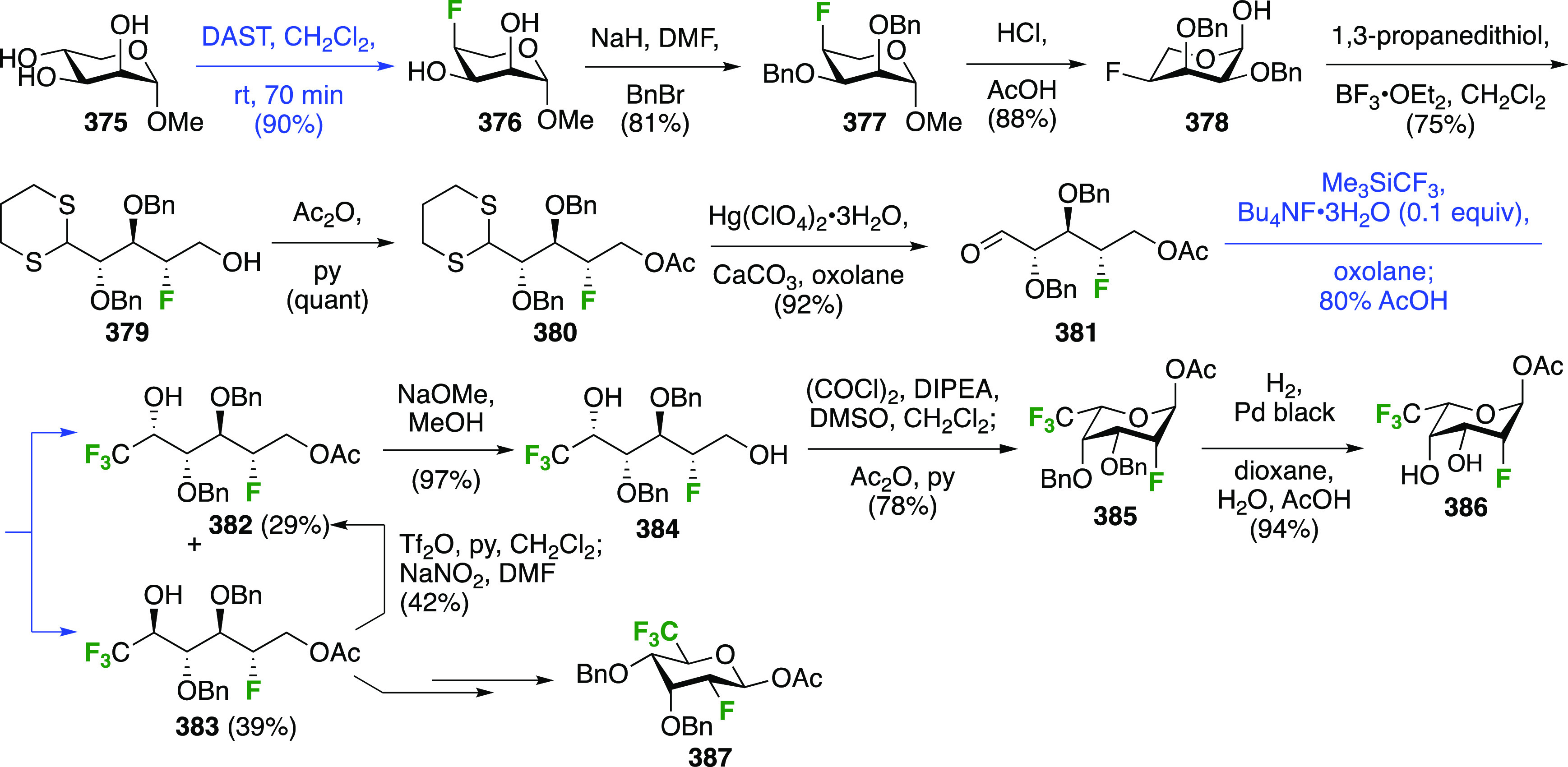
Synthesis of 2,6-Dideoxy-2,6,6,6-tetrafluorinated Talose and Allose Derivatives^318^

The tetrafluorinated derivative **386** (Scheme 52) was synthesized by the Takagi group from methyl-α-d-lyxopyranoside **375** using a head-to-tail strategy through the addition of a CF_3_ group at the precursor C-1 position.^318^ Hence, nucleophilic fluorination of **375** at C-4 with inversion of the configuration was achieved through a reaction with DAST, to afford 4-deoxy-4-fluoro-β-l-ribopyranoside **376** in an excellent yield. The observed regioselectivity mirrored that of the fluorination of methyl α-mannoside as reported by Somawardhana (see below, Scheme 65), with the OH-2 group promoting fluorination at C-4.^301^ Benzylation of **376** with benzyl bromide to give **377** was followed by anomeric hydrolysis to give the reducing sugar **378**, the major α-anomer of which is depicted (4:1 ratio in chloroform). Treatment with 1,3-propanedithiol and BF_3_·OEt_2_ gave the ring-opened dithioacetal **379**, and protection of the terminal alcohol as the acetate, followed by deprotection of the aldehyde group, gave **381** ready for trifluoromethylation. This was achieved by reaction with Me_3_SiCF_3_ and catalytic TBAF,^319^ which after hydrolysis of residual TMS-ether formed in situ led to a mixture of epimers **382** and **383**. The d-allitol derivative **383**, which was undesired in this case, could be converted to **382** by alcohol inversion. Deacetylation of **382** allowed oxidation to the aldehyde, which was achieved in chemoselective fashion thanks to the reduced reactivity of the trifluorocarbinol group.^320^ The aldehyde spontaneously converted to the corresponding α-l-talopyranose, isolated as the anomeric acetate **385**, upon which hydrogenolysis delivered **386**. In parallel, **383** went through the same route to give the β-d-allose **387** (not shown).

### 3.10 Fluorination at Positions 3 and 4

#### 3.10.1 Difluorinated at Positions 3 and 4

**Scheme 53:**
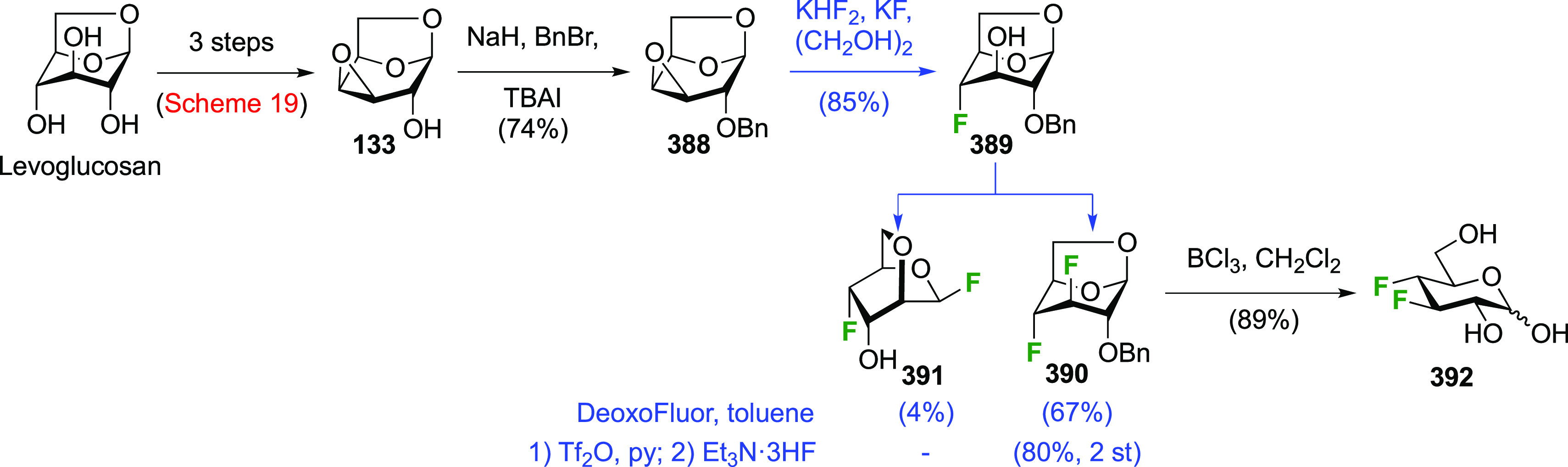
Synthesis of 3,4-Dideoxy-3,4-difluoro-d-glucopyranose^215,224^

The synthesis of the 3,4-dideoxy-3,4-difloro glucose **392** (Scheme 53) was initially described by the Linclau group,^224^ and later improved by the Giguère group,^215^ starting from known **133** (cf. Scheme 19). Benzylation of the alcohol group was achieved by adding the NaH base to a premixed solution of **133** and BnBr while keeping the temperature at 0 °C to avoid the Payne rearrangement.^321^ Epoxide opening using a 1:1 mixture of KHF_2_/KF resulted in the formation of **389** in 85% yield. The alcohol **389** was then treated with DAST in refluxing CH_2_Cl_2_ for 20 h resulting in the dideoxy difluorinated levoglucosan analogue **390** in 54% yield, while the use of DeoxoFluor in toluene at 70 °C for 2 h gave compound **390** in 67% yield, together with only 3% of unreacted **389**, and 4% of byproduct **391**, which arose from neighboring group participation of O6. An improved fluorination was reported by Giguère: a 2-step triflation and fluoride substitution with HF·3HF.^215,272^ Finally, concomitant deprotection of OH-2 and opening of the anhydro-bridge were achieved with BCl_3_ to give the desired difluorinated glucose analogue **392**.^215,224^

**Scheme 54:**
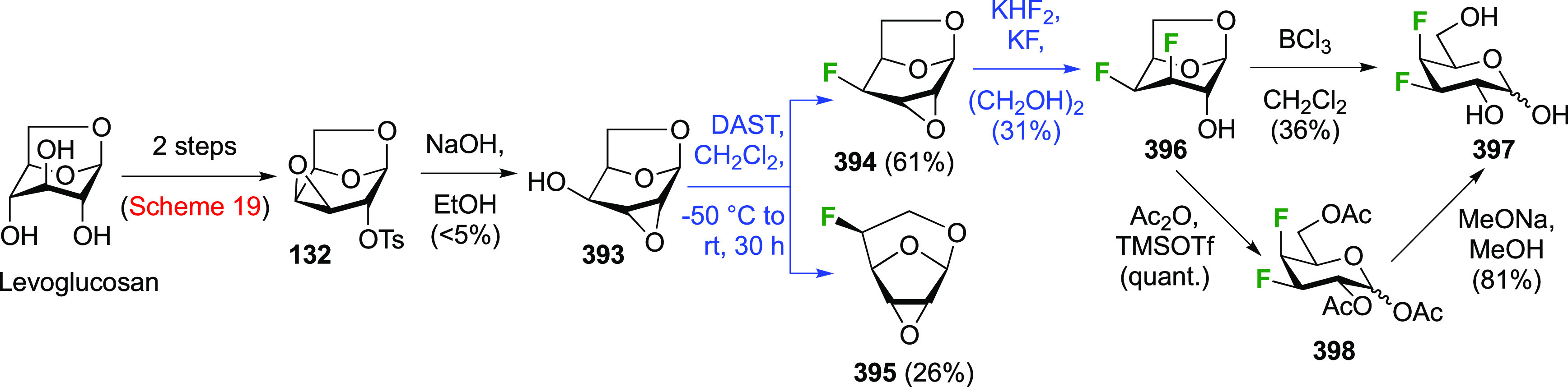
Synthesis of 3,4-Dideoxy-3,4-difluoro-d-galactopyranose^274^

The synthesis of 3,4-dideoxy-3,4-difluorogalactose **397** was published by the Linclau group (Scheme 54).^274^ Starting from known crystalline tosylate **132** (cf. Scheme 19), reaction with sodium hydroxide and ethanol gave the 1,6:2,3-dianhydro derivative **393**. As described by Karban et al.,^146^ reaction of the epoxide **393** with DAST gave the desired compound **394** (61% yield) along with the byproduct **395** (26%). The fluoride-mediated epoxide opening of **394** gave the desired difluorinated **396** in 31% yield. Opening of the 1,6-anhydro-bridge was achieved using BCl_3_ to form the corresponding glycosyl chloride, which was directly hydrolyzed to give **397**, in 36% yield. A much higher-yielding procedure involved TMSOTf-catalyzed acetolysis to give **398**, which could then be deprotected to give **397** in 81% yield over two steps.

#### 3.10.2 Tetrafluorinated at Positions 3 and 4

**Scheme 55:**
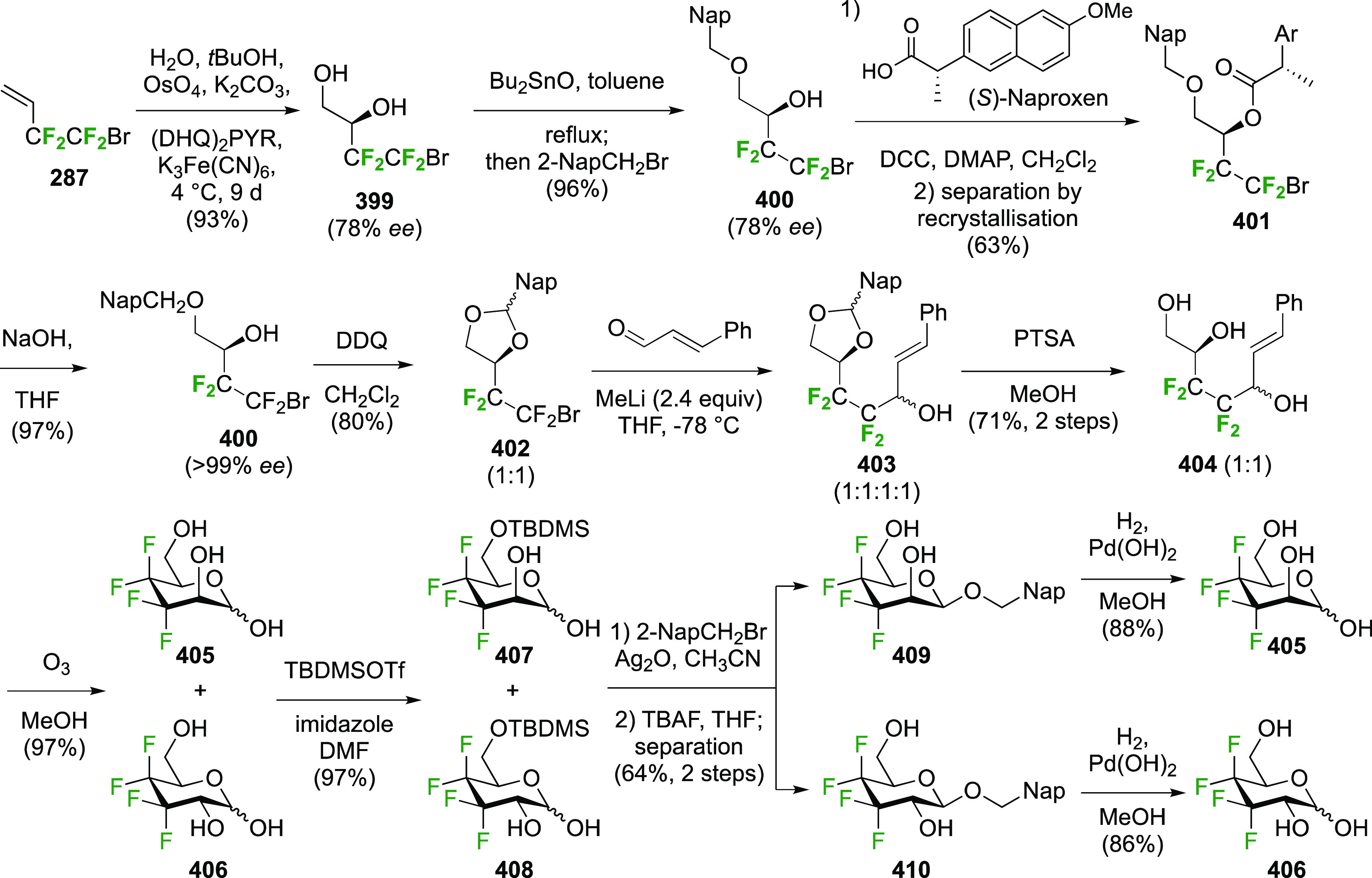
Synthesis of 3,4-Dideoxy-3,3,4,4-tetrafluoro-d-*threo*-hexopyranose **405** and 3,4-Dideoxy-3,3,4,4-tetrafluoro-d-*erythro*-hexopyranose **406**(288)

The synthesis of 3,4-dideoxy-3,3,4,4-tetrafluorinated sugar derivatives was reported by Linclau et al. using a fluorinated building block approach (Scheme 55).^288^ Sharpless asymmetric dihydroxylation of **287** required modification with enhanced levels of OsO_4_ and ligand to accommodate the reduced reactivity of the deactivated alkene (cf. Scheme 36), as well as the use of (DHQ)_2_PYR instead of the usual (DHQ)_2_PHAL ligand. This gave **399** in an excellent yield and, as expected for terminal alkenes, moderate enantioselectivity after 9 days.^290^ Protection of the primary alcohol led to **400**, and its functionalization with (*S*)-Naproxen allowed separation of the thus formed diastereomers to get, after ester cleavage, **400** in >99% enantiopurity. The expensive (*S*)-Naproxen could be recovered and recycled. Protection of the secondary alcohol group by DDQ-mediated cyclization gave **402** as a mixture of acetal diastereomers,^288^ which could now be lithiated and reacted with cinnamaldehyde. This addition was not diastereoselective and gave **403** as a 1:1:1:1 mixture of diastereomers. Acetal hydrolysis and alkene ozonolysis led to the formation of the desired tetrafluorinated sugar derivatives **405** and **406**, which were not separable. Selective silylation at the primary position, anomeric alkylation with 2-naphthyl methyl bromide, and silyl removal gave the separable **409** and **410**, each of which could now be deprotected to give the pure **405** and **406**.^288^

### 3.11 Fluorination at Positions 3 and 6

#### 3.11.1 3,6-Difluorinated Glucose Derivatives

**Scheme 56:**
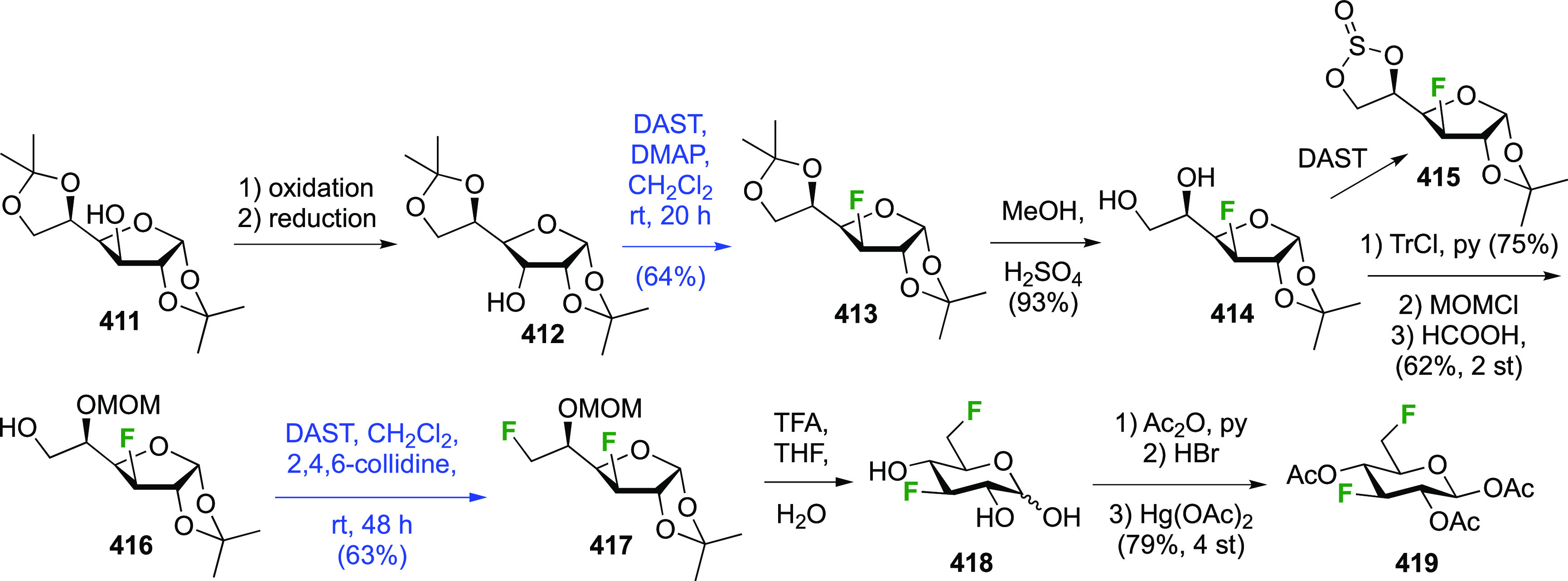
Synthesis of 3,6-Dideoxy-3,6-difluoro-β-d-glucopyranose^311^

The Withers group reported a synthesis of 3,6-dideoxy-3,6-difluoro glucopyranose **418** (Scheme 56) from glucose diacetonide **411**.^311^ Conversion to the corresponding allose **412** using an oxidation–reduction sequence was then followed by DAST-mediated deoxyfluorination at C-3 to give **413**.^139,214^ Selective deprotection of the terminal acetonide in **413** was achieved using sulfuric acid in methanol, giving **414** in 93% yield. Direct fluorination at C-6 was unsuccessful and only led to a 5,6-cyclic sulfite byproduct **415**. Hence, a three-step protecting group manipulation sequence was carried out to give **416**. Treatment with DAST gave the difluorinated product **417**, which upon deprotection gave the 3,6-dideoxy-3,6-difluoro-d-glucopyranose **418**. Successive acetylation of the free hydroxyl groups, bromination of the anomeric position, and displacement with acetate gave the peracetylated 3,6-dideoxy-3,6-difluoro-β-d-glucopyranose **419** in 79% yield over four steps.

**Scheme 57:**
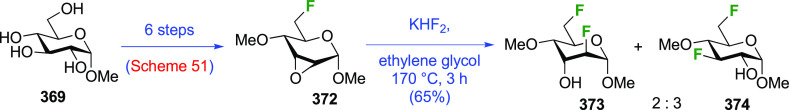
Synthesis of a 3,6-Dideoxy-3,6-difluoro Glucopyranoside Derivative^315^

As discussed in Scheme 51, a 3,6-dideoxy-3,6-difluorinated glucose was also obtained by fluoride opening of the 6-deoxy-6-fluoro-2,3-epoxy allose derivative **372** with potassium hydrogen difluoride in ethylene glycol, which gave a mixture of inseparable methyl 2,6-dideoxy-2,6-difluoro-4-*O*-methyl-α-d-altropyranose **373** and methyl 3,6-dideoxy-3,6-difluoro-4-*O*-methyl-α-d-glucopyranose **374** in 65% yield (Scheme 57).^315^

**Scheme 58:**
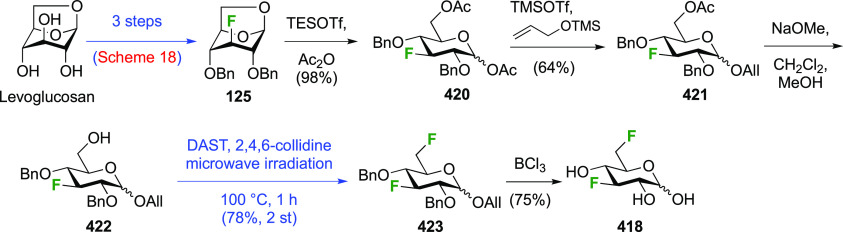
Synthesis of 3,6-Dideoxy-3,6-difluoro-α-d-glucopyranose^215^

Giguère’s group prepared 3,6-dideoxy-3,6-difluoroglucopyranose **418** (Scheme 58), starting from commercially available levoglucosan.^215^ Conversion to **125** as detailed in Scheme 18 was followed by 1,6-anhydro-bridge opening and selective acetolysis, giving compound **420** in 98% yield. The anomeric position was protected using glycosidation with allyloxytrimethylsilane to afford intermediate **421**. This allowed acetate removal at C-6, followed by deoxyfluorination to afford **423**. Final deprotection with BCl_3_ afforded the desired 3,6-difluoroglucose analogue **418** in 75% yield.^215^

#### 3.11.2 3,6-Difluorinated Allose Derivatives

**Scheme 59:**
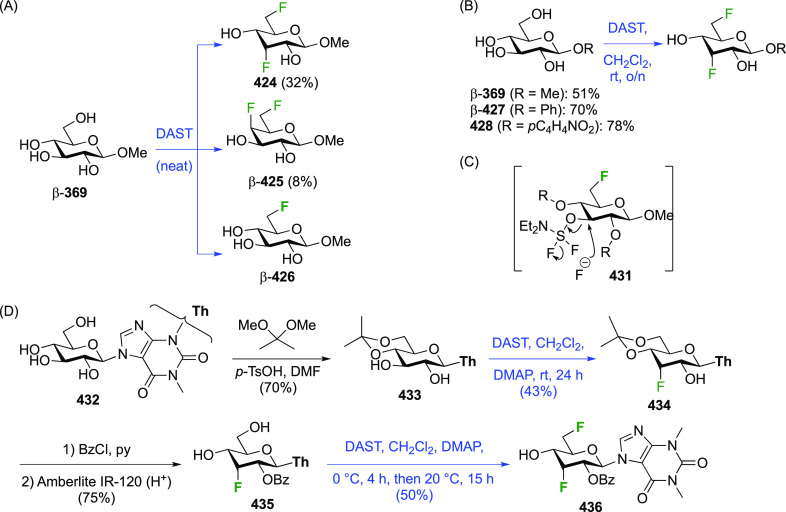
Synthesis of 3,6-Dideoxy-3,6-difluoro-allopyranosides Using Direct and Sequential Difluorination^301,302,322^

The Somawardhana group reported that reaction of unprotected methyl β-d-glucopyranoside β-**369** with neat DAST at room temperature (Scheme 59A) gave 3 main products: methyl 3,6-dideoxy-3,6-difluoro-β-d-allopyranoside **424** in 32% yield, methyl 4,6-dideoxy-4,6-difluoro-β-d-glucopyranoside **425** in 8% yield, and methyl 6-deoxy-6-fluoro-β-d-glucopyranoside **426** (no yield reported).^301^

The same year, the Card group also published direct fluorination of unprotected glucosides using DAST,^302^ but with CH_2_Cl_2_ as the solvent, at −40 °C (Scheme 59B). Methyl β-d-glucopyranoside β-**369**, phenyl β-d-glucopyranoside **427**, and *p*-nitrophenyl β-d-glucopyranoside **428** gave their corresponding 3,6-dideoxy-3,6-difluoro-allopyranoside products in 51%, 70%, and 78% yields, respectively. There was no mention of fluorination at the 4-position. This dideoxy difluorination process was reported to be facile, and the remarkable regioselectivity explained by activation of all alcohol groups by DAST with the primary position reacting first, leading to **431** (Scheme 59C). The reactivity at the 2- and 4-positions is reduced due to the presence of an antiperiplanar C–O bond, as well as to the higher electron withdrawing effect of the acetal center. With a β-configured glycoside, approach of the fluoride nucleophile toward C-3 is unhindered, hence leading to a facile reaction.

A sequential fluorination approach for the synthesis of a 3,6-dideoxy-3,6-difluoroallose derivative **436** has also been reported,^322^ starting from 7-β-d-glucopyranosyl theophylline **432** (Scheme 59D). Positions 4 and 6 were first protected as the acetonide,^323^ which allowed for selective fluorination of position 3 using DAST to give **434** in 43% yield. Benzoyl protection of position 2 and acetonide removal, to give **435**,^324^ was then followed by fluorination of position 6 in 50% yield.^322^

### 3.12 Fluorination at Positions 4 and 6

#### 3.12.1 4,6-Difluorinated Galactose Derivatives

**Scheme 60:**
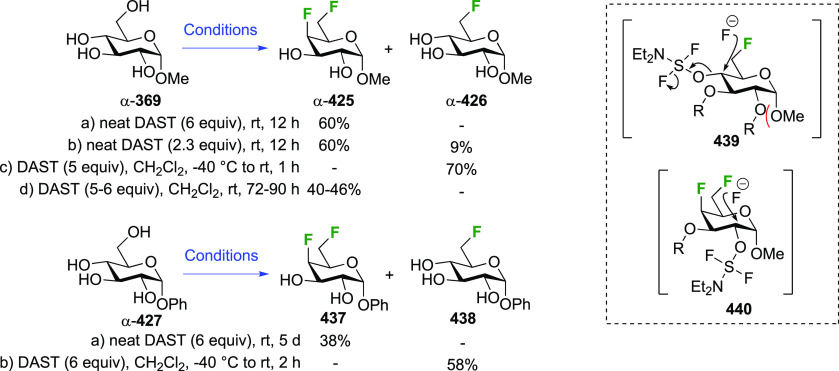
Direct Dideoxy Difluorination of α-Glucosides^300−302,325^

The synthesis of 4,6-dideoxy-4,6-difluorinated galactose is possible in one step from α-configured glucosides (Scheme 60). The Somawardhana group reported that reaction of methyl α-d-glucopyranoside α-**369** with neat DAST gave the 4,6-difluorinated methyl galactoside α-**425** in 60% yield (a).^300^ Reducing the number of equivalents (b) also led to α-**425** in 60% yield, this time with 9% of the monofluorinated methyl glucoside α-**426**.^301^ While the Card group initially reported that when dichloromethane is used as solvent only α-**426** is obtained (c),^229^ they later found that stirring at room temperature for 3–4 days led to the difluorinated α-**425** in 40–46% yield.^302,325^ Similar observations were made with phenyl α-glucoside α-**427**, which transformed to **437** in neat DAST, and to **438** when dichloromethane was used as solvent.^302^ The regioselectivity of the difluorination reaction was explained as follows (also see Scheme 59 with the explanation of the selectivity starting from the β-anomer):^301^ activation of all glucoside alcohol groups would occur, with the primary position reacting fast, to the 6-deoxy-6-fluorinated derivative **439**. Reaction at the 3-position is sterically hindered by the axial anomeric substituent, and reaction at the 2-position is disfavored due to the electron withdrawing acetal center. This makes the 4-position the next-fastest to react, leading to **440**. Now, reaction at the 2-position is additionally hindered by the axial fluorine at C-4, and workup then leads to α-**425**.

**Scheme 61:**
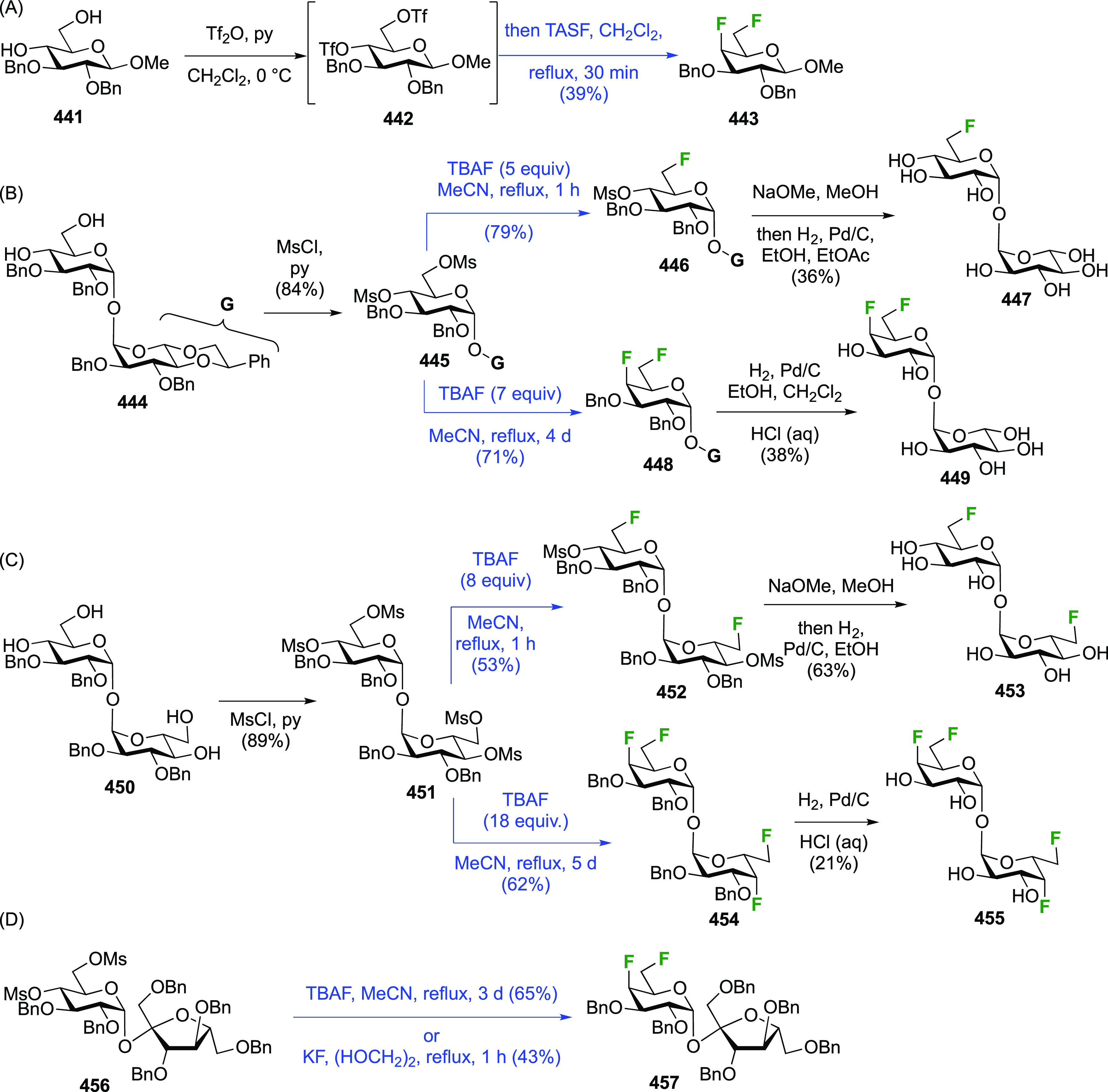
Difluorination of Glucoside Derivatives at the 4- and 6-Positions via Displacement of Sulfonates^115,327,329,330^

Deoxyfluorinations at the 4- and 6-positions not relying on DAST have also been developed. These require protection at C-2 and C-3, although the anomeric configuration is now not important. The Szarek group converted **441** (Scheme 61A), which can be obtained from methyl β-glucoside in three standard steps (not shown), in a one-pot operation to **443** via the bis-triflate **442** in moderate yield with TASF.^115^ The Richardson group investigated the synthesis of fluorinated trehalose derivatives via di-*O*-mesylate fluorination. Starting from **444** (Scheme 61B), which can be obtained from trehalose in three steps, mesylation gave the fluorination substrate **445**.^326^ Treatment of **445** with excess TBAF in refluxing acetonitrile for 1 h only led to monofluorination, resulting in **446** which, after mesylate methanolysis and deprotection, gave 6-deoxy-6-fluorotrehalose **447**. In contrast, refluxing **445** for 4 days yielded the difluorination product **448** in 71% yield,^327^ which after deprotection resulted in 4,6-dideoxy-4,6-difluoro-α-d-galactopyranosyl-α-d-glucopyranoside **449**.

The Richardson group also synthesized the 4,4′,6,6′-tetramesylated trehalose derivative **451** as a substrate, from the readily available **450** (Scheme 61C).^328^ Subjecting the tetramesylate **451** to 8 equiv of TBAF in refluxing acetonitrile for 1 h led to the formation of the difluorinated **452** in moderate yield. After methanolysis with methoxide and benzyl hydrogenolysis, this resulted in the 6,6′-dideoxy-6,6′-difluorotrehalose **453**. In contrast, when **451** was heated for 5 days with a larger excess of TBAF, the tetrafluorinated **454** was obtained in 62% yield.^329^ Hydrogenolysis then gave 4,6-dideoxy-4,6-difluoro-α-d-galactopyranosyl 4,6-dideoxy-4,6-difluoro-α-d-galactopyranoside **455**.

Finally, the same group also investigated difluorination on the dimesylated sucrose derivative **456** (Scheme 61D),^330^ the reaction of which with either TBAF in refluxing acetonitrile or KF in refluxing ethylene glycol gave the difluorinated **457**.

#### 3.12.2 4,6-Difluorinated Glucose Derivatives

**Scheme 62:**
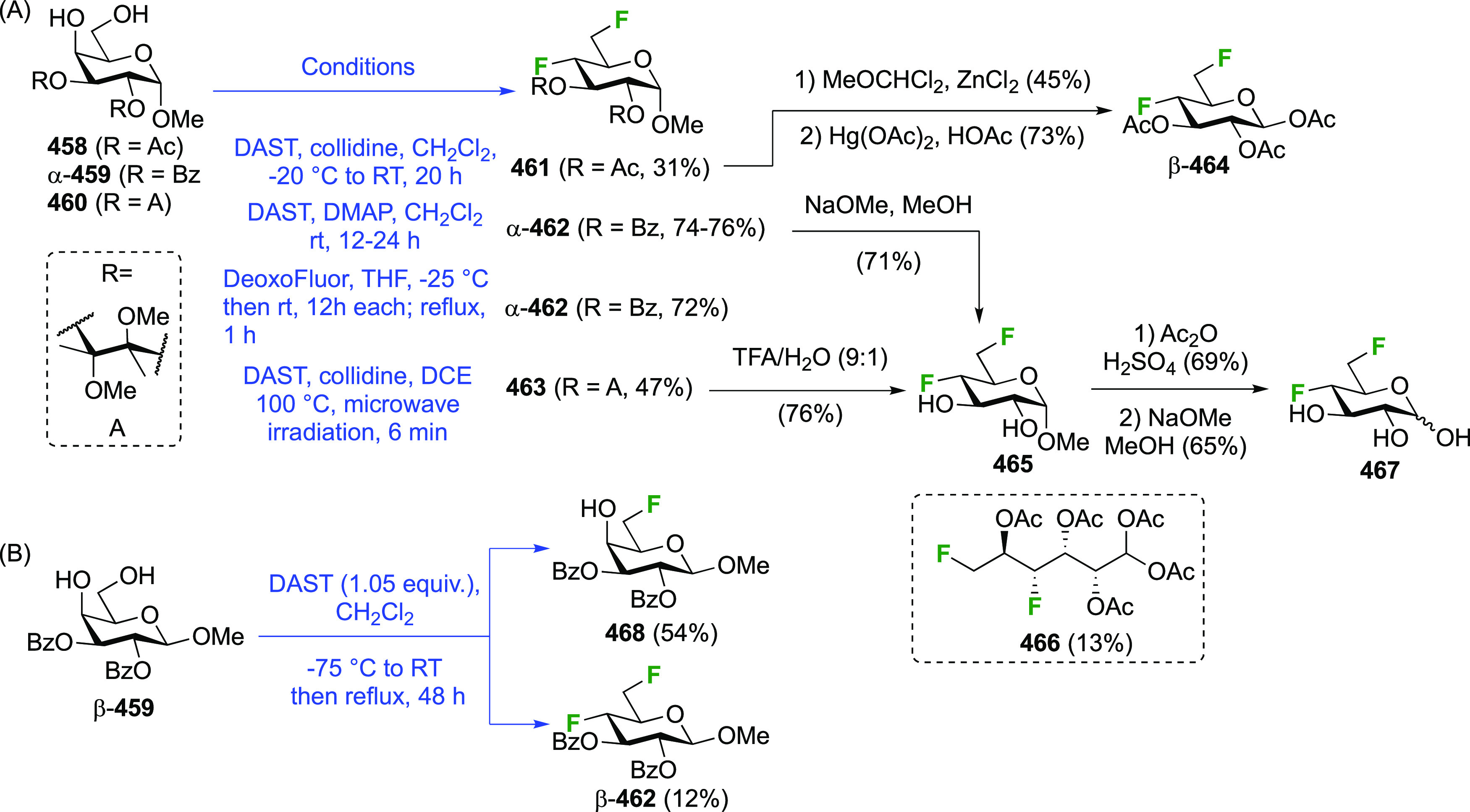
DAST-Mediated Dideoxy Difluorination Approaches to 4,6-Difluorinated Glucose Derivatives^230,311,325,331^

Direct DAST-mediated dideoxy difluorination leading to 4,6-difluorinated *gluco*-configured derivatives requires galactoside protection at the 2,3-position. Hence, the 2,3-di-*O*-acetate **458** (Scheme 62A) was shown by the Withers group to lead to **461** in a moderate 31% yield,^311^ but the Hoff group reported much higher yields from the benzoate α-**459**, either using DAST/DMAP at room temperature or with DeoxoFluor at reflux temperature.^230,325^ With the 2,3-butanedioxyacetal protected **460**, the Linclau group obtained a lower yield (47%),^325^ which is to a certain extent offset by its more efficient preparation (1 step from methyl α-galactoside) as opposed to three steps for **458**/α-**459**.

The diacetate **461** was converted to its glycosyl chloride, and then to the β-triacetate **464**.^311^ The benzoate α-**462** was debenzoylated to give **465**, which was also obtained by hydrolysis of the butanediacetal protecting group in **463**.^325^ Anomeric acetolysis and acetate methanolysis then gave 4,6-dideoxy-4,6-difluoro-d-glucopyranose **467**. Interestingly, the acetolysis reaction also yielded a ring opened 1,1-diacetoxy containing product **466**.^325^

With the 2,3-positions protected, DAST-mediated deoxyfluorination at the 4 and 6-positions is also possible from β-galactosides. The Magnusson group applied this process to the corresponding methyl 2,3-di-*O*-benzoyl-β-galactopyranoside β-**459** (Scheme 62B), albeit with only 1.05 equiv of DAST.^331^ Even so, it was found that the difluorinated β-**462** is still isolated in 12% yield, alongside 54% of the 6-fluorinated product **468**, which gives an indication of the reactivity at the 4-position.

**Scheme 63:**
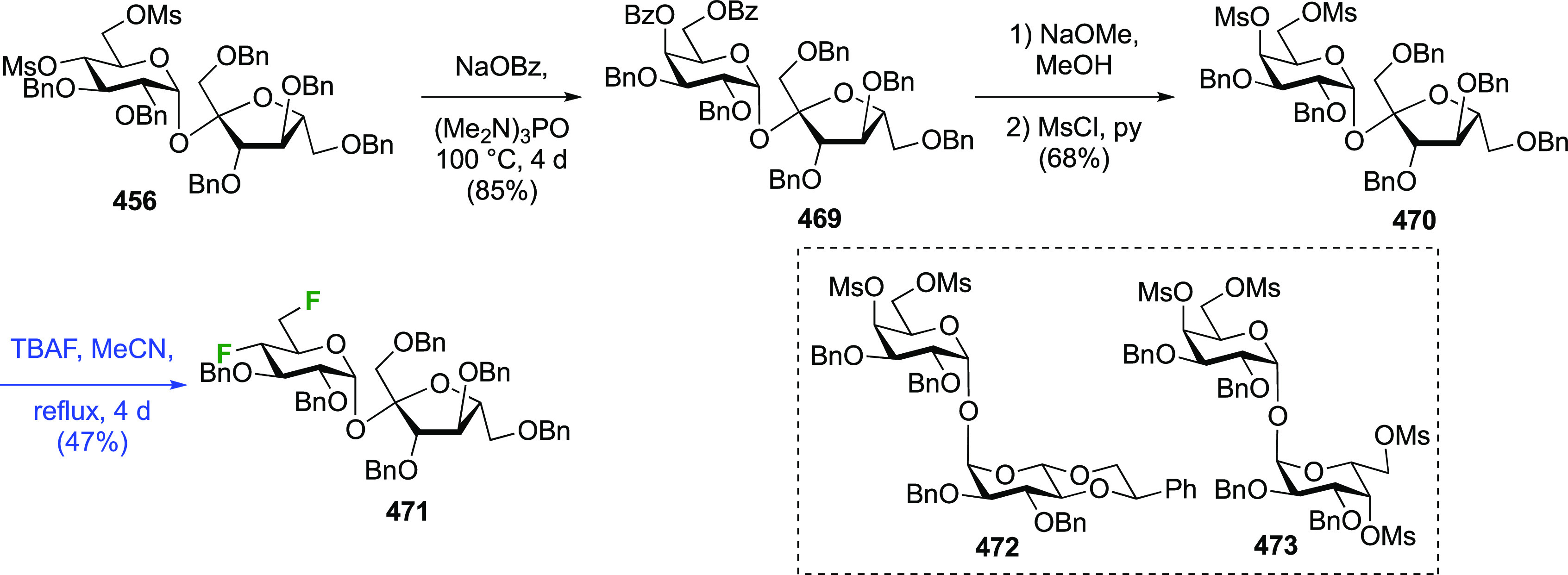
Synthesis of 4,6-Difluorinated Glucose Derivatives by Mesylate Displacement^330^

Difluorination using 4,6-di-*O*-mesylate derivatives has also been explored. The Richardson group synthesized 4,6-dideoxy-4,6-difluorosucrose **471** (Scheme 63) via displacement of the dimesylate **470** with TBAF. This dimesylate was synthesized from **456** (see Scheme 61) by nucleophilic substitution with sodium benzoate, benzoate methanolysis, and mesylation. Compared to the corresponding dimesylate **456**, fluorine substitution with **470** proved more difficult, with an unidentified elimination product isolated as well.^330^ Fluoride displacement of the 4,6-dimesylated *galacto*-configured trehalose derivatives **472** and **473** was reported to give mainly elimination products.^327,329^

**Scheme 64:**
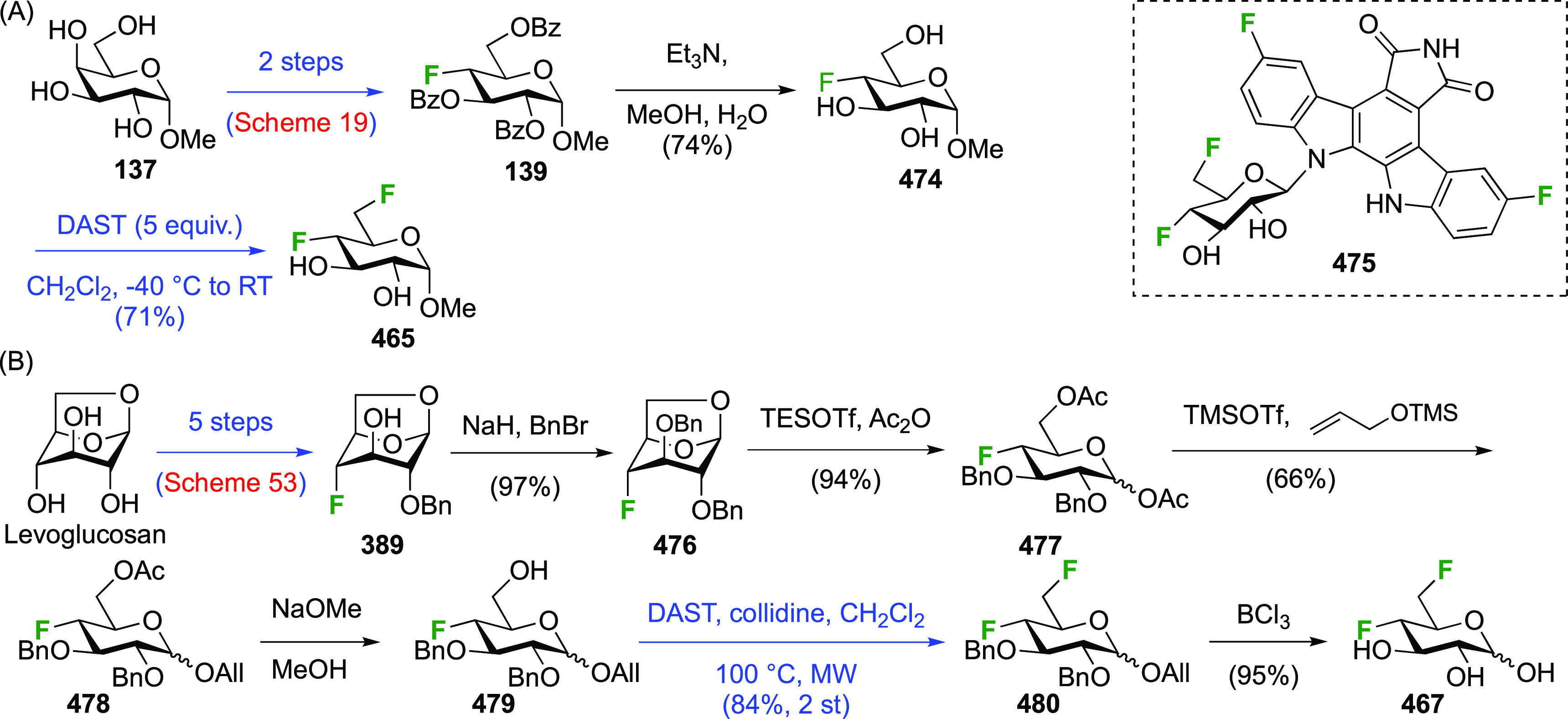
Sequential Fluorination Approach to 4,6-Difluorinated Glucose Derivatives^215,302^

Finally, a sequential approach has also been used. The Card group obtained **139** (Scheme 64A) in two steps from methyl α-d-galactoside **137** (as described in Scheme 19), which was then debenzoylated to give **474**.^302^ Treatment with DAST gave the 4,6-difluorinated glucoside **465** in good yield. The same approach was followed by the Saulnier/Balasubramanian group at Bristol Myers Squibb toward the antitumor compound analogue **475**,^332^ and also by the Giguère group in their synthesis from levoglucosan (Scheme 64B).^215^ The intermediate **389**, obtained in five steps as described in Scheme 53, was fully protected, and the resulting **476** subjected to anhydro-bridge opening to give **477**. Protection at the anomeric position to give **478** allowed selective deprotection at C-6, upon which deoxyfluorination resulted in **480**. Global deprotection then gave 4,6-dideoxy-4,6-difluoroglucose **467**.

#### 3.12.3 4,6-Difluorinated Talose Derivatives

**Scheme 65:**
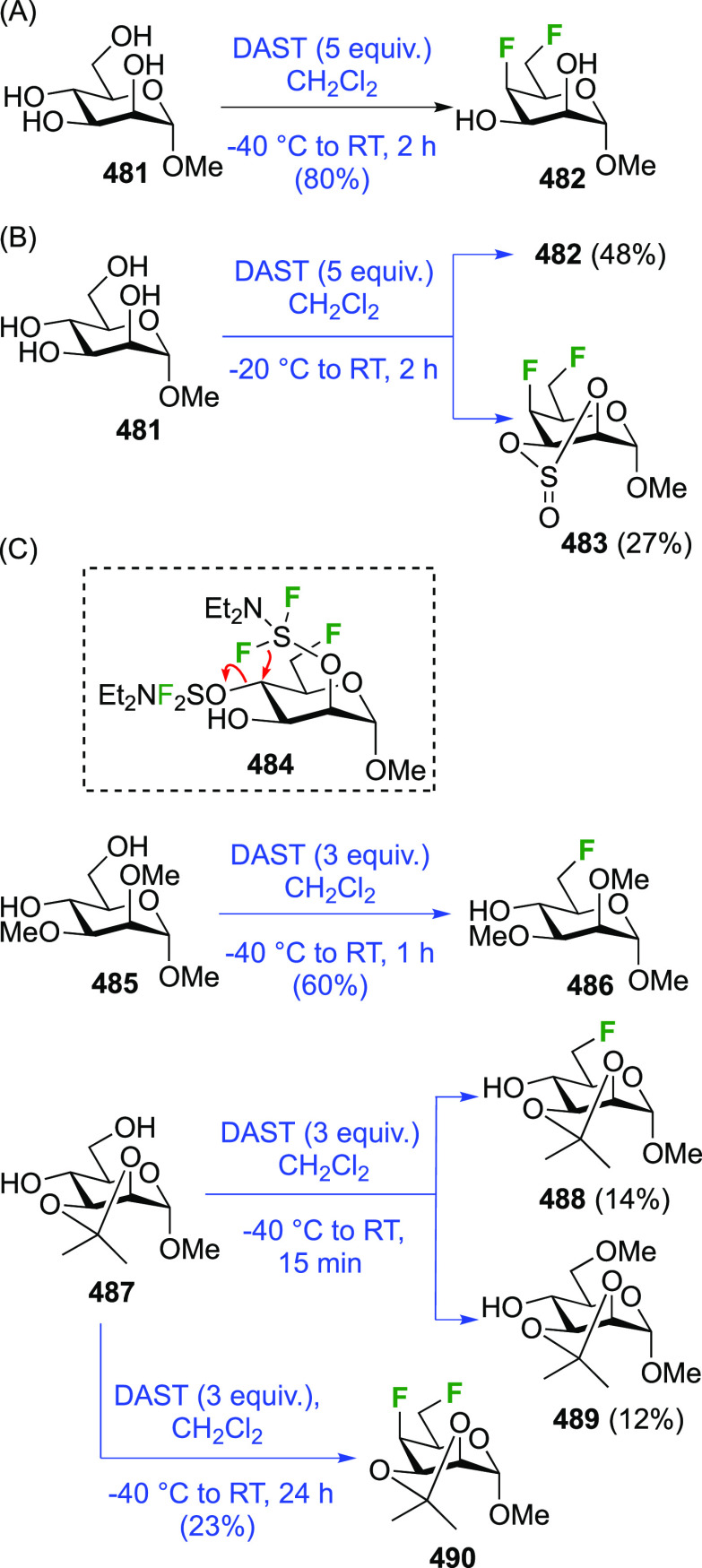
Direct Dideoxy Difluorination Reactions toward 4,6-Difluorinated Talose Derivatives^229,230,301,302,333^

The Somawardhana and Card groups achieved the conversion of unprotected methyl α-mannopyranoside **481** (Scheme 65A) to the 4,6-difluorinated talopyranoside **482** in excellent yields, either in neat DAST (72%, not shown),^301^ or with dichloromethane as the solvent (80%).^229,333^ The reaction was reported to be more facile than reaction with methyl α-glucopyranoside (see Scheme 60). The Hoff group reported that under very similar reaction conditions **482** was isolated in only 48% yield (Scheme 65B),^230^ and that cyclic sulfite **483** was also obtained, which may be due to a difference in workup conditions. Nevertheless, the combined yield of 75% does indicate the ease of mannose difluorination.

The Card group further reported that this double fluoride displacement was so facile that monofluorination at the 6-postion of **481** could not be achieved,^302^ in contrast to the reaction with methyl α-glucopyranoside (see Scheme 60). This was explained by the involvement of intermediate **484** (Scheme 65C), which would allow an intramolecular fluoride delivery to displace the activated OH-4 group. This was further investigated by subjecting the 2,3-di-*O*-methyl mannoside **485** to the reaction conditions, which indeed only returned the monofluorinated **486**. It is nevertheless surprising that in the reaction of **481** with DAST, no neighboring group participation of the *trans*-diaxial anomeric methoxy group, with subsequent formation of the glycosyl fluoride, has been reported (the Somawardhana group reported an unidentified side product in 1% yield).^301^ In order to achieve the synthesis of 6-deoxy-6-fluoromannosides, the reaction was also carried out with **487**, which has a more easily removable protecting group at the 2,3-positions. However, only low yields of **488** were obtained, with the formation of side-product **489** explained by methanol displacement of the activated OH-6 during the workup. Subjecting **487** to DAST with a longer reaction time did lead to the 4,6-difluorinated talose derivative **490**, albeit in a low yield.^302^

### 3.13 Fluorination at Positions 5 and 6

**Scheme 66:**
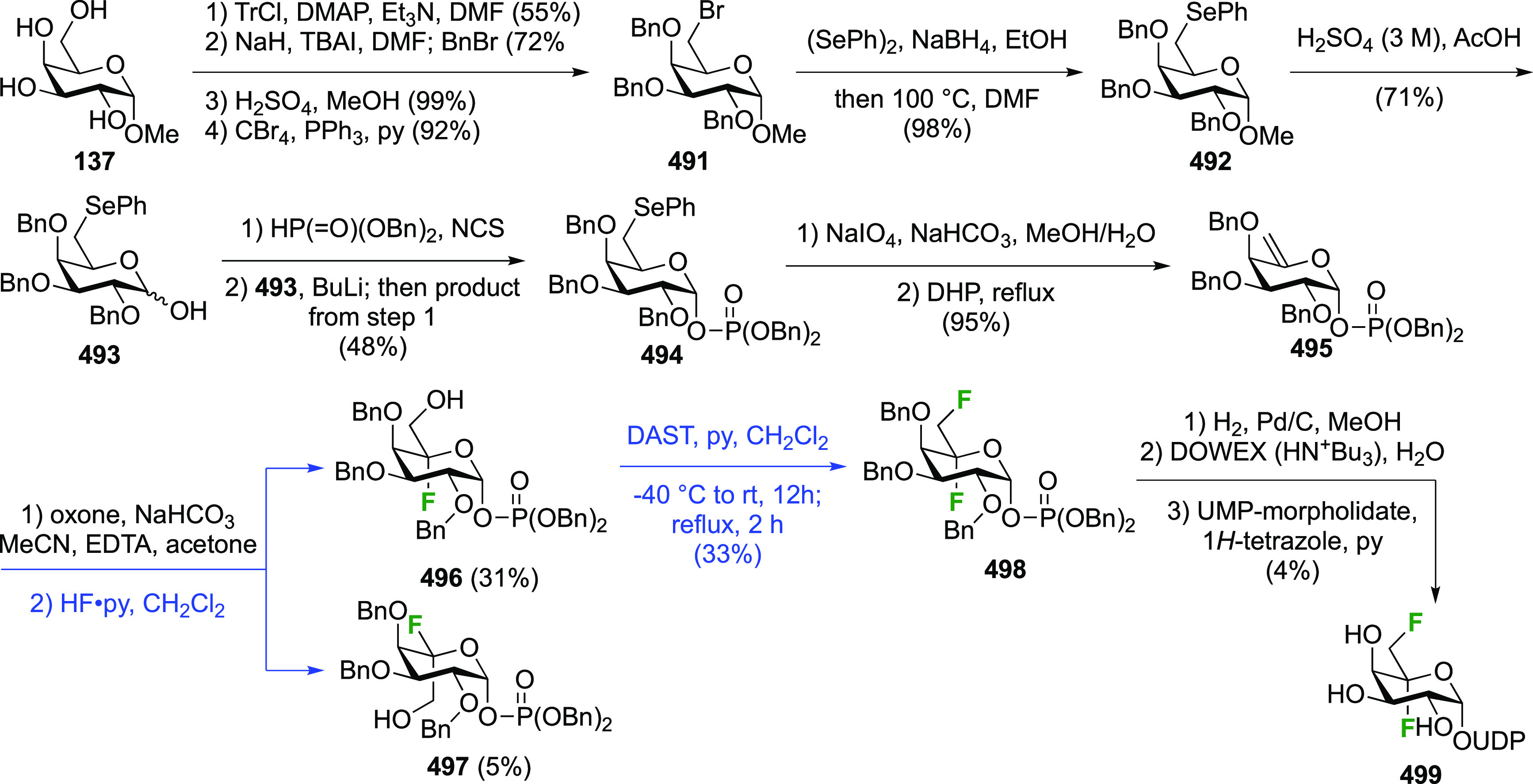
Synthesis of UDP-6-Deoxy-5,6-difluoro-α-d-galactopyranose^334^

A 5,6-difluorinated UDP-galactosyl derivative **499** was synthesized by the Liu group to investigate the mechanism of UDP-galactopyranose mutase.^334^ The fluorine at the 5-position was not introduced via a radical bromination step (as seen for the other 5-fluorinated derivatives discussed above), but by using Coward’s 5,6-epoxide fluoride opening.^335^ Starting from methyl α-d-galactoside **137** (Scheme 66),^334^ a standard protection–deprotection sequence was followed by C-6-bromination to give **491**. Displacement of bromide by phenyl selenide (generated in situ) led to **492**, upon which the anomeric dibenzyl phosphate group was introduced after anomeric hydrolysis to **493**. Elimination of the resulting selenide **494** to give the C-5–C-6 exocyclic double bond **495** allowed formation of the corresponding epoxide which, upon treatment with HF-py, led to regioselective opening to give both C-5-fluoro epimers **496** and **497**. These were separable, and the desired major isomer was subjected to DAST to get the vicinal difluoro moiety in **498**. Deprotection and UDP introduction finally gave **499**. The Coward methodology allowed a C-5 fluoride introduction that was compatible with the phosphate protecting group. This sequence of events was necessary given the phosphate was introduced via the hemiacetal, and given the instability of reducing 5-fluoropyranoses, C-5 fluorination was required after the desired anomeric functionalization was completed.^335^

## 4 Aldohexoses: Fluorination at Three Positions

### 4.1 Fluorination at Positions 1,2,5

**Scheme 67:**
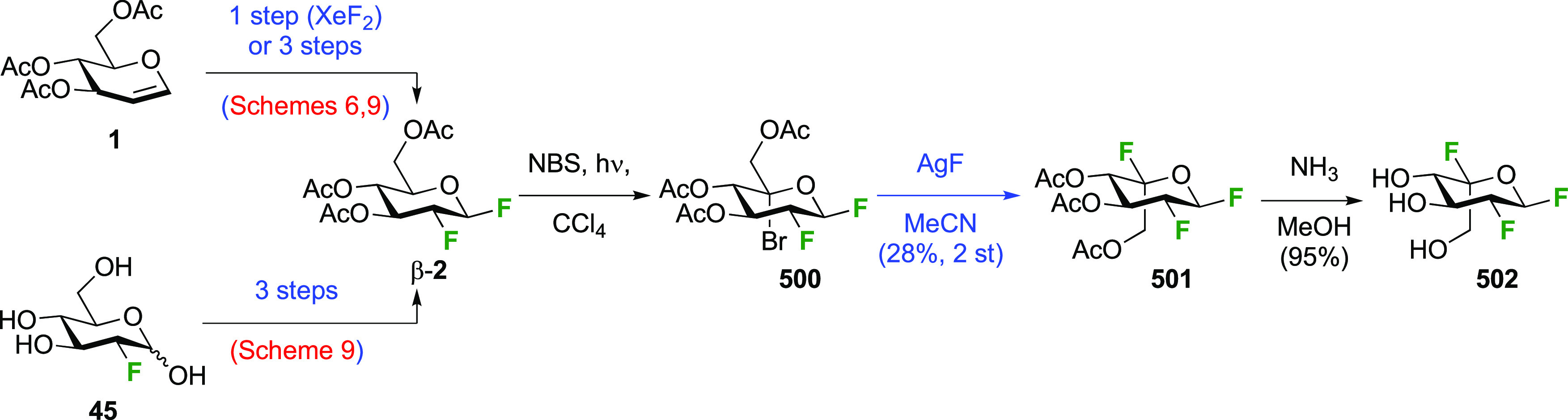
Synthesis of 2-deoxy-2,5-difluoro-α-l-idopyranosyl Fluoride^205^

The Withers group synthesized the trifluorinated idose derivative **502** (Scheme 67) as a glycosidase inactivator, starting from the 1,2-difluorinated glucose derivative β-**2**. This can be accessed as detailed in Schemes 6 and 9 either from tri-*O*-acetyl glucal **1** in one step (CF_3_OF) albeit in low yield (12%), or in three steps involving reaction with SelectFluor and AgF-mediated fluorine introduction of the corresponding glucosyl bromide, or from 2-deoxy-2-fluoroglucose **45** also via its glycosyl bromide. Radical bromination was selective for the 5-position to give **500** only, and fluoride displacement with inversion of configuration led to the l-ido configured **501**, with both reactions seemingly unaffected by the presence of the fluorine at the 2-position. Deprotection then gave 2-deoxy-2,5-difluoro-α-l-idopyranosyl fluoride **502**.^205^

### 4.2 Fluorination at Positions 1,2,6

**Scheme 68:**
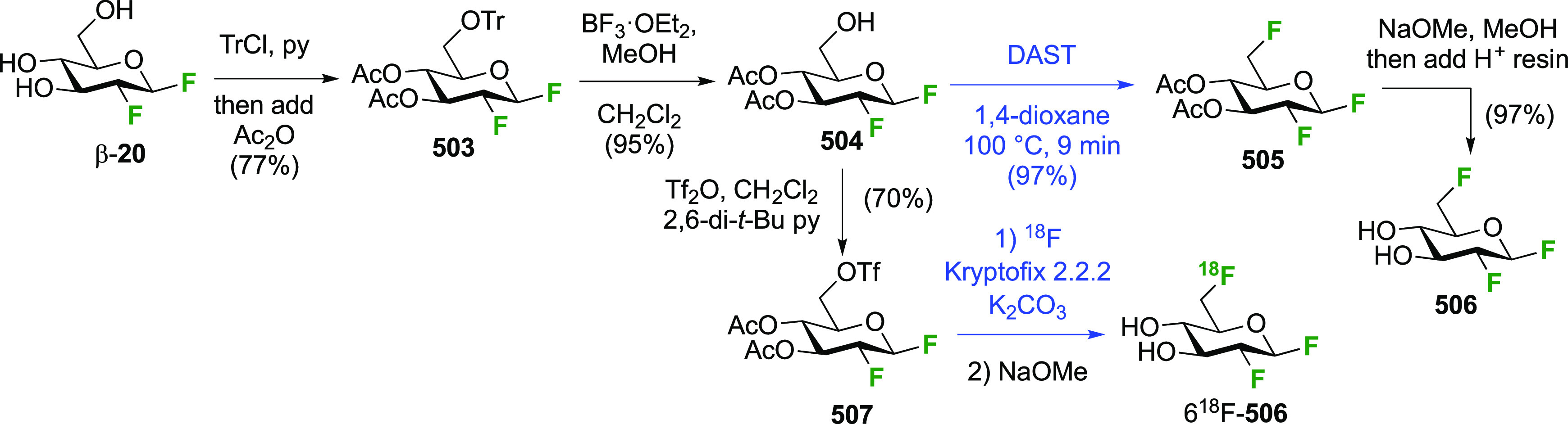
Synthesis of 6-[^18^F]-2,6-dideoxy-2,6-difluoro-β-d-glucopyranosyl Fluoride^336^

The Withers group synthesized the 1,2,6-trifluorinated glucose **506** as a potential imaging probe for glucocerebrosidase (Scheme 68) starting from β-**20** (see section 3.1.1).^336^ Selective tritylation and protection of the remaining alcohol groups gave **503**, from which the trityl group was then removed to expose the OH-6 group ready for deoxyfluorination. This could be achieved with DAST to give **505** in excellent yield, and deprotection then gave 2,6-dideoxy-2,6-difluoro-β-d-glucopyranosyl fluoride **506**. Alternatively, ^18^F radiolabeling at the 6-position was achieved via the triflate **507** via Kryptofix 2.2.2/K_2_CO_3_ assisted nucleophilic fluorination with fluoride-18, followed by acetate deprotection. A 9% radiochemical yield for 6-[^18^F]-**506** was reported, for a synthesis/purification time of 2 h and 43 min.

### 4.3 Fluorination at Positions 2,3,4

#### 4.3.1 Trifluorinated at Positions 2,3,4

##### 4.3.1.1 2,3,4-Trifluorinated Glucose Derivatives

**Scheme 69:**
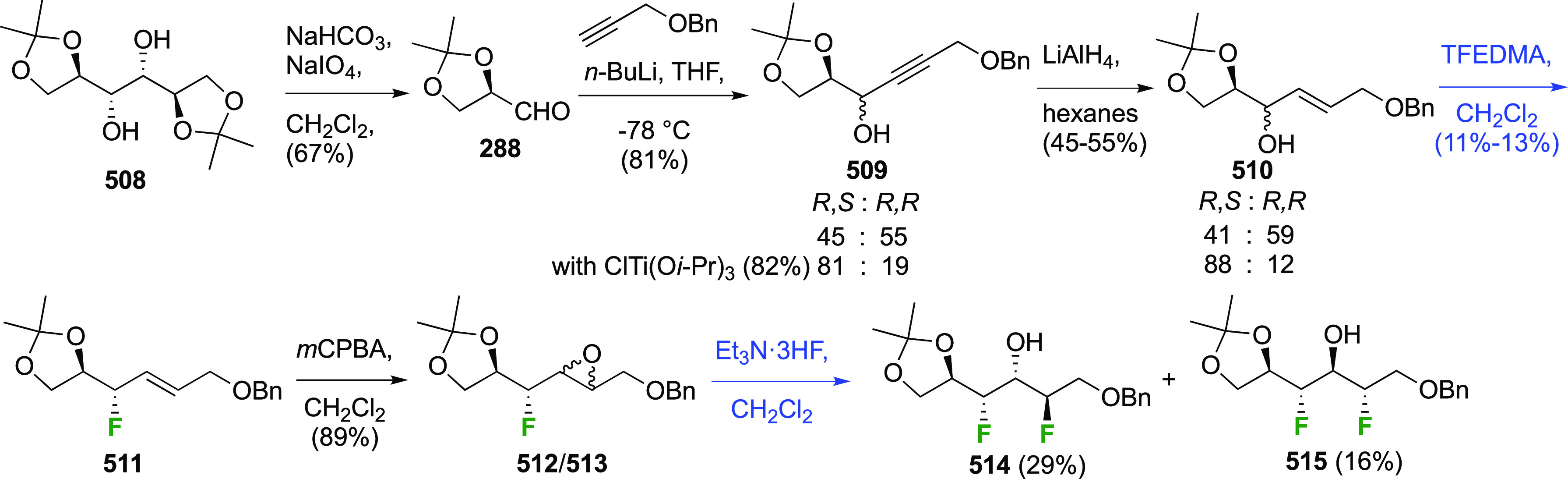
Synthesis of an Advanced Precursor toward 2,3,4-Trideoxy-2,3,4-trifluoro-d-glucopyranose^339^

The first synthesis of fully deprotected 2,3,4-trideoxy-2,3,4-trifluoro-d-glucopyranose was achieved by the O’Hagan group via a *de novo* synthesis approach, with **515** as a key advanced intermediate (Scheme 69).^337^

A first-generation approach to generate **515** started from aldehyde **288**, made from periodate cleavage of 1,2:5,6-di-*O*-isopropylidene-d-mannitol **508**. The addition of deprotonated benzyl propargyl ether gave the adduct **509** in low stereoselectivity, which could be improved by employing ClTi(O*i*-Pr)_3_ as a nonchelating Lewis acid.^338^ Propargylic alcohol reduction with LiAlH_4_ gave **510**. Deoxyfluorination was then performed on the diastereomeric mixtures with tetrafluoroethyl dimethylamine (TFEDMA) in dichloromethane. It was found that this reaction proceeded with significant S_N_1 character, giving all four possible allylic fluoride regio/stereomers in similar ratios regardless of the ratio of alcohols. The desired stereomer **511** could be obtained pure in 11–13% yield.^339^ Epoxidation gave the two diastereomers **512** and **513** in 89% yield. This mixture was treated with Et_3_N·3HF to give the separable diastereoisomers **514** and **515** in, respectively, 16 and 29% yield, with the desired stereomer being the minor isomer.^337^

**Scheme 70:**
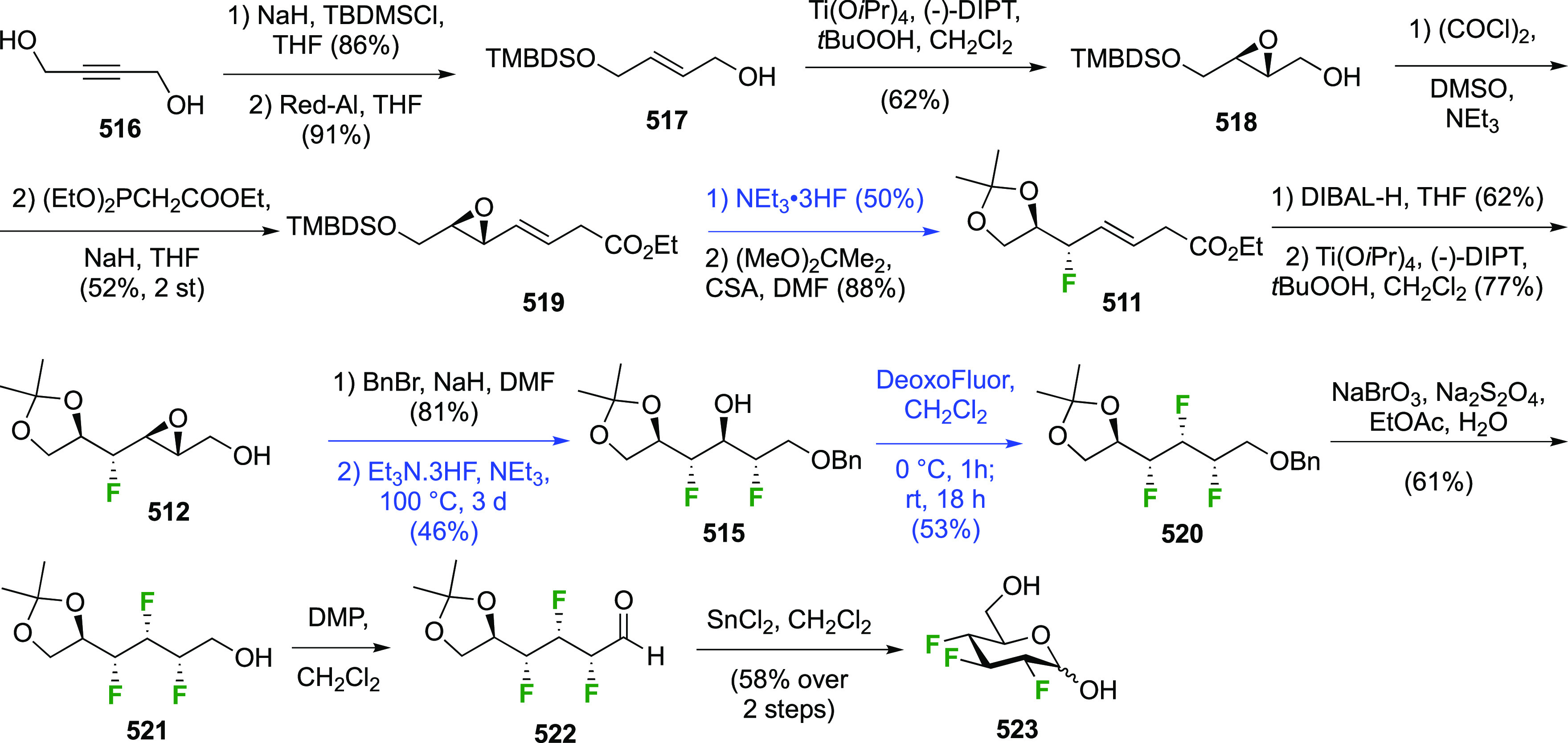
O’Hagan’s Synthesis of 2,3,4-Trideoxy-2,3,4-trifluoro-d-glucopyranose^337,340^

A second generation approach to **515** was successful in avoiding the formation of stereomeric mixtures (Scheme 70).^340^ It started from the commercially available butynediol **516**, which was selectively mono- protected with TBDMS chloride, and reduction of the resulting derivative with Red-Al gave *trans*-allylic alcohol **517** in 91% yield. Sharpless epoxidation of allylic alcohol **517** provided epoxide (2*R*,3*R*)-**518** in 62% yield in 89% ee. Swern oxidation of epoxide **518**, followed by the reaction with triethyl phosophonoacetate, gave ester **519** in 52% yield over the two steps. Treatment of enone **519** with Et_3_N·3HF resulted both in deprotection of the TBDMS group, and in the opening of the epoxide to provide, after diol protection as acetonide, the first fluorinated intermediate **511**. The opening of the epoxide proceeded in a 10:1 regioselectivity, and the protection as the acetonide allowed separation of the regioisomers, giving pure **511**. Reduction of the ester with DIBAL-H gave an allylic alcohol as a suitable substrate for a Sharpless epoxidation to introduce the remaining stereochemistry. This led to epoxide **512** in a 10:1 stereomeric ratio. After protection of the free alcohol, Et_3_N·3HF-mediated opening of the epoxide generated **515** in 46% yield as a single diastereoisomer.

Then treatment of **515** with DeoxoFluor gave the trifluoroacetal **520** in 53% yield. Deprotection of the benzyl was performed using Adinolfi’s method to give the deprotected trifluoroacetal **521** in 61% yield. This was oxidized with Dess-Martin periodinane to give an α-fluoroaldehyde **522** which was directly reacted with SnCl_2_ in dichloromethane to cleave the acetonide and perform the cyclization to generate 2,3,4-trideoxy-2,3,4-trifluoro-d-glucopyranose **523** in 58% yield over two steps. This synthesis was completed in 15 synthetic steps in an overall yield of 0.37%.

**Scheme 71:**
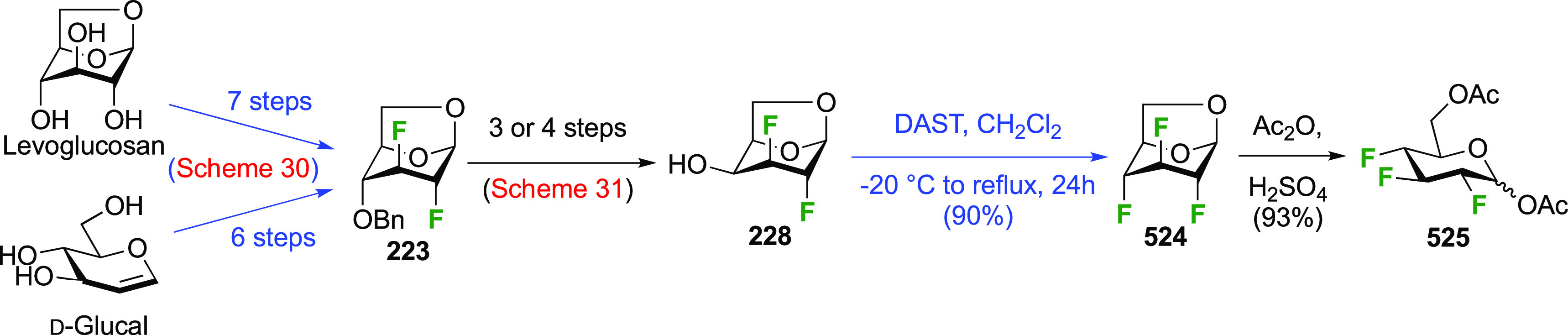
Sarda Synthesis of Peracetylated 2,3,4-Trideoxy-2,3,4-trifluoro-d-glucopyranose^58^

In 1989, the Lukacs group had reported the synthesis of the protected 2,3,4-trideoxy-2,3,4-trifluoro-d-glucopyranose **525** (Scheme 71).^58^ This synthesis started from levoglucosan, proceeding via the key intermediates **223** and **228**, described in Schemes 30 and 31. Reaction of **228** with DAST in dichloromethane occurred with inversion of configuration to give **524** in excellent yield. Opening of the 1,6-anhydro-bridge and acetyl protection were performed with acetic anhydride and sulfuric acid, giving the desired compound **525** in 93% yield

**Scheme 72:**
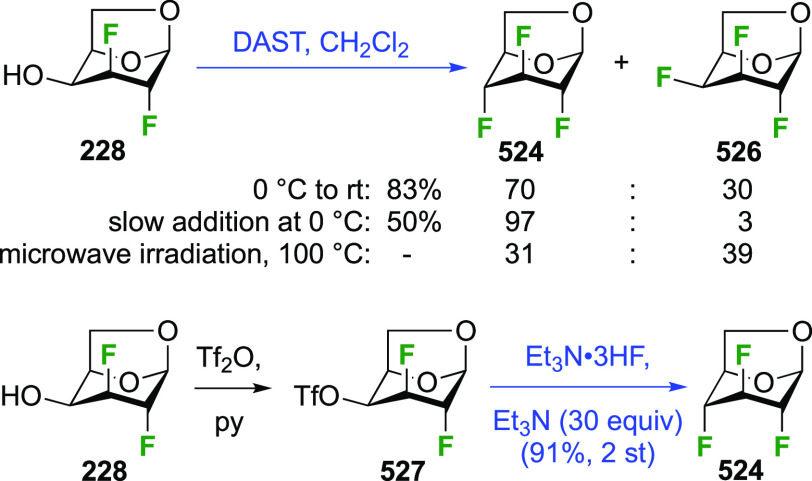
Deoxyfluorination of **20**(102,296)

However, the final DAST-mediated fluorination was found difficult to reproduce. Linclau et al. found that deoxyfluorination gave an inseparable mixture of inversion and retention of configuration (Scheme 72), with 30% of the galacto-configured **526** formed.^296^ Slower addition of DAST led to a much improved ratio, but at the expense of yield. This was also found by Giguère et al., who obtained **526** as the major product under microwave conditions.^102^ They eventually found a successful alternative, in that triflation followed by the addition of in situ formed Et_3_N·1HF yielded **524** in excellent yield.^102^ They also reported that the use of TBAF·3H_2_O was successful in displacing the triflate (not shown).^275^

**Scheme 73:**
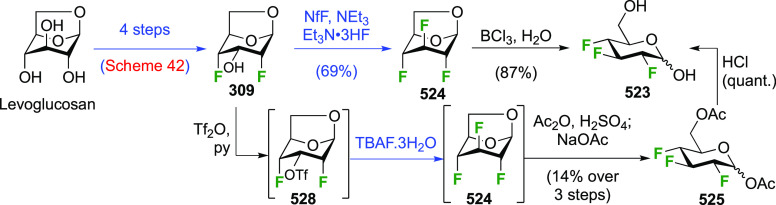
Linclau and Giguère Syntheses of 2,3,4-Trideoxy-2,3,4-trifluoro-d-glucopyranose^215,296^

The Linclau group later reported a shorter synthesis of 2,3,4-trideoxy-2,3,4-trifluoro-d-glucopyranose employing **309** as a key intermediate (Scheme 73).^296^ Fluorination of **309** to give **524** was achieved with the use of nonafluorobutyl sulfonyl fluoride (NfF) in the presence of Et_3_N·3HF as the external fluoride source. Finally, BCl_3_-mediated opening of the 1,6-anhydro-bridge gave the desired **523** in excellent yield. This constituted a six-step synthesis of 2,3,4-trideoxy-2,3,4-trifluoro-d-glucose **523** from levoglucosan in 24% overall yield.^296^ Giguère et al. reported a similar synthesis of **523** using an alternative fluorination method.^215^ Here, **309** was first converted to the corresponding triflate, allowing nucleophilic fluorination to give **524**, which was immediately subjected to acetolysis to give **525** in 14% yield over three steps. Acetyl hydrolysis with HCl afforded the desired compound **523** in quantitative yield.

##### 4.3.1.2 2,3,4-Trifluorinated Galactose Derivatives

**Scheme 74:**
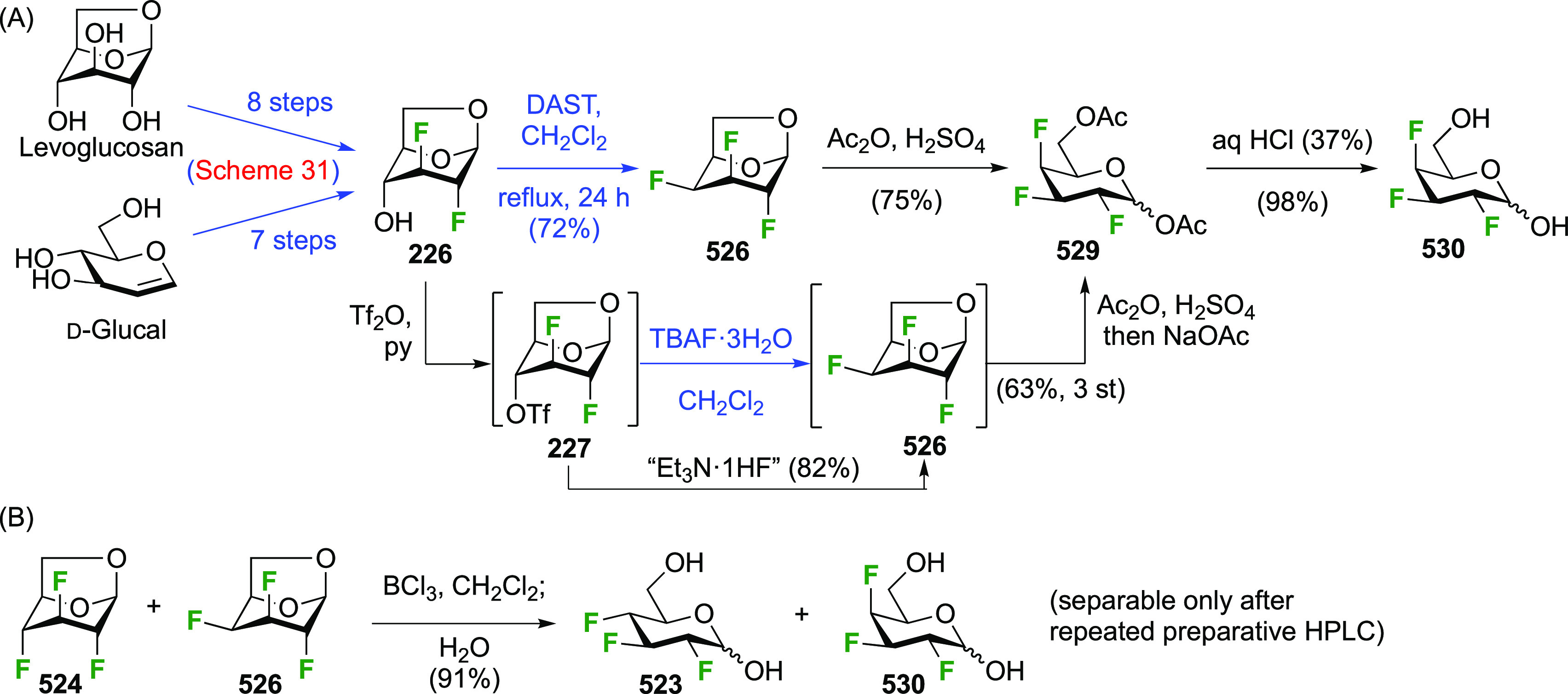
Synthesis of 2,3,4-Trideoxy-2,3,4-trifluoro-d-galactopyranose^58,102,275,296^

The synthesis of peracetylated 2,3,4-trideoxy-2,3,4-trifluoro-d-galactopyranose **529** (Scheme 74A) was first described by Lukacs, from the intermediate **226** also used for their corresponding glucose synthesis.^58^ Treatment of **226** with DAST was reported to give **526**, after which 1,6-anhydro-bridge acetolysis led to **529**. Alternatively, Giguère carried out the deoxyfluorination via the triflate **227** by reaction with TBAF·3H_2_O.^275^ The thus formed **526** was then directly converted to **529** in 63% overall yield. Using in situ formed Et_3_N·1HF, fluoride displacement went cleanly in 82% yield.^102^ Acetate hydrolysis of **529** was described by Giguère to give 2,3,4-trideoxy-2,3,4-trifluoro-d-galactopyranose **530** in excellent yield.

Opening of the 1,6-anhydro-bridge directly to give the free trifluorinated galactose was reported by Linclau et al., using BCl_3_ (Scheme 74B).^296^ This was carried out on the mixture of **524** and **526** (see Scheme 72), and the trifluorinated derivatives **523** and **530** proved just about separable by preparative HPLC.

##### 4.3.1.3 2,3,4-Trifluorinated Talose Derivatives

**Scheme 75:**
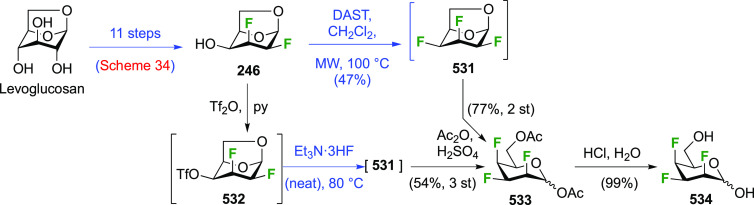
Synthesis of 2,3,4-Trideoxy-2,3,4-trifluoro-d-talopyranose^275^

The synthesis of 2,3,4-trideoxy-2,3,4-trifluoro-d-talopyranose **534** (Scheme 75) was realized by the Giguère group from the advanced intermediate **246**, which had been used for the synthesis of 2,3-dideoxy-2,3-difluorinated talose (Scheme 34).^275^ Interestingly, deoxyfluorination of **246** with DAST was shown to proceed with retention of configuration to give **531** in 47% yield.^102^ When this reaction was immediately followed by acetolysis, losses due to evaporation of the volatile **531** were avoided, and **533** was obtained in 77% yield.^275^ Fluorination at C-4 via the corresponding triflate **532** using Et_3_N·3HF also led to retention of the configuration,^102^ but 3% of the inversion product was also isolated (not shown). Acetolysis of the mixture thus obtained led to **533** in 54% yield over three steps. Deprotection finally gave 2,3,4-trideoxy-2,3,4-trifluoro-d-talopyranose **534**.^275^

##### 4.3.1.4 2,3,4-Trifluorinated Mannose Derivatives

**Scheme 76:**
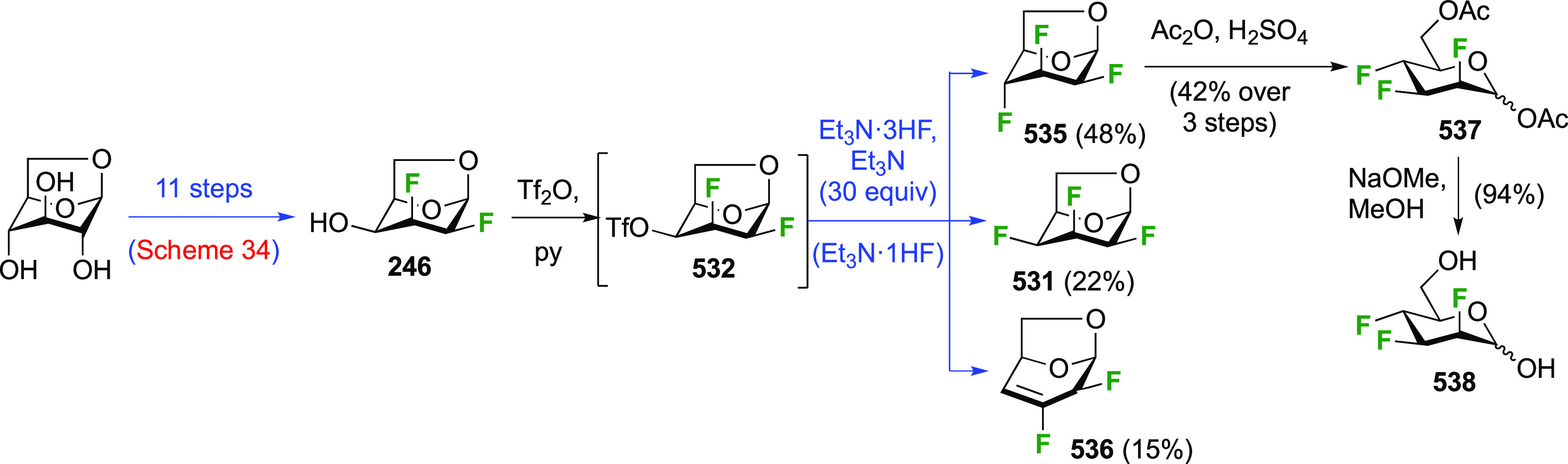
Synthesis of 2,3,4-Trideoxy-2,3,4-trifluoro-d-mannopyranose^275^

For the 2,3,4-trifluorinated mannose synthesis (Scheme 76), further investigation of the fluorination of the triflate **532** by the Giguère group led to the use of Et_3_N·1HF, which gave the product **535** resulting from inversion of configuration as the major product,^275^ although the *talo*-configured derivative **531** resulting from retention of configuration and the elimination side product **536** were formed in appreciable quantities.^102^ Acetolysis of **535** led to **537**, and final deprotection gave 2,3,4-trideoxy-2,3,4-trifluoro-d-mannopyranose **538**.^275^

##### 4.3.1.5 2,3,4-Trifluorinated Altrose Derivatives

**Scheme 77:**
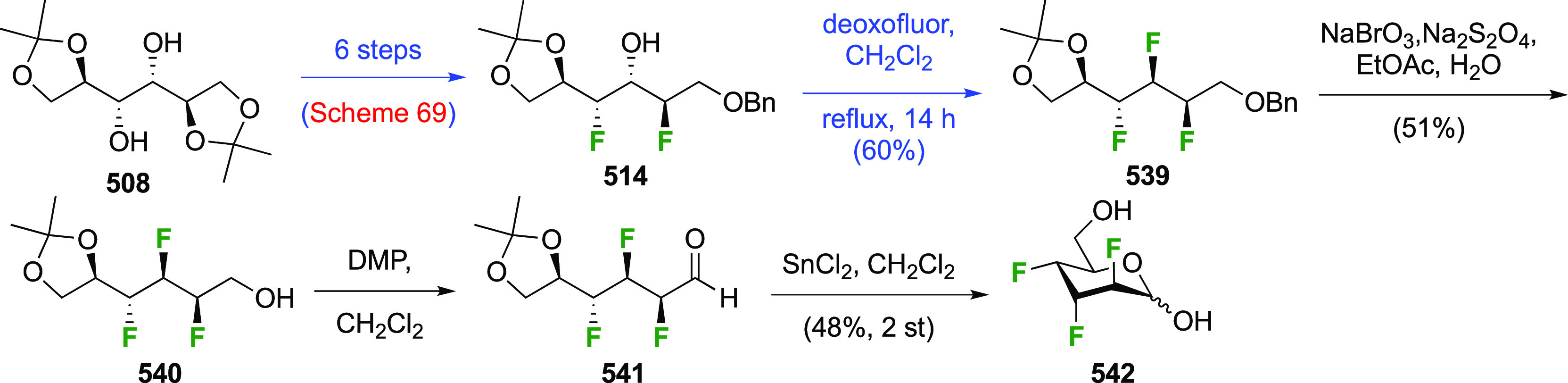
Synthesis of 2,3,4-Trideoxy-2,3,4-trifluoro-d-altropyranose^337^

The synthesis of 2,3,4-trideoxy-2,3,4-trifluoro-d-altropyranose **542** was reported by O’Hagan as part of their first generation 2,3,4-trideoxy-2,3,4-trifluoro-d-glucopyranose synthesis (cf. Scheme 69).^337^ In this synthesis, **514** was obtained as a byproduct originating from an unselective epoxidation reaction. Treatment of **514** with DeoxoFluor (Scheme 77) led to the introduction of the third fluorine to give **539** in 60% yield. Deprotection of the primary alcohol was followed by oxidation with Dess-Martin periodinane to give the aldehyde **541**, which was directly reacted with SnCl_2_ in dichloromethane to effect cyclization to generate 2,3,4-trideoxy-2,3,4-trifluoro-d-altropyranose **542** in 48% yield over two steps.

##### 4.3.1.6 2,3,4-Trifluorinated Allose Derivatives

**Scheme 78:**
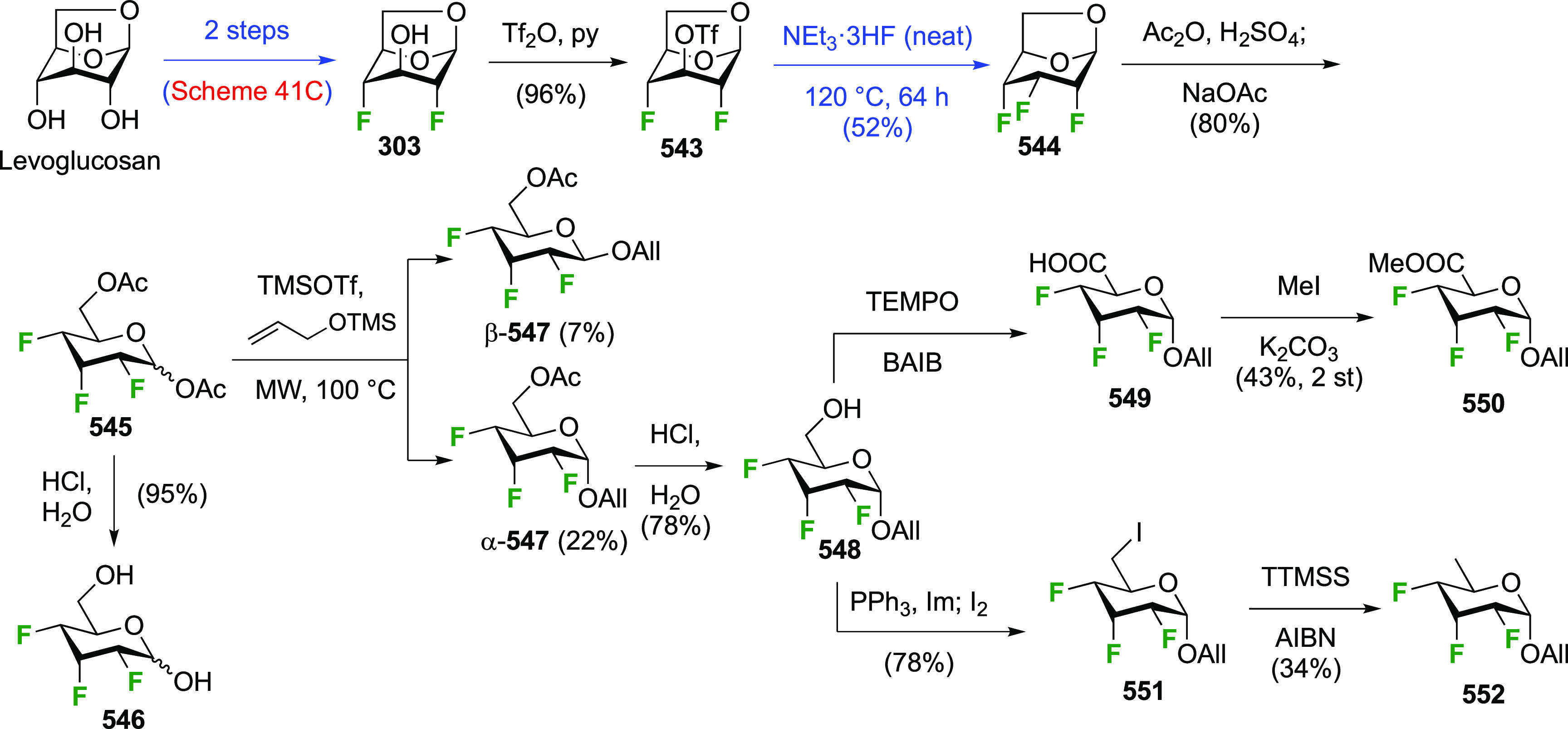
Synthesis of 2,3,4-Trideoxy-2,3,4-trifluoro-d-allopyranose^275,295^

The Giguère group also disclosed the synthesis of a number of 2,3,4-trifluorinated allose derivatives (Scheme 78),^295^ from the difluorinated levoglucosan derivative **303** that was efficiently obtained in a 2-step procedure as described in Scheme 41C.^275,296^ Introduction of the third fluorine atom via triflation and treatment with Et_3_N·3HF led to **544**, upon which acetolysis resulted in the formation of **545**.^295^ Deprotection then gave 2,3,4-trideoxy-2,3,4-trifluoro-d-allopyranose **546**.^275^

From **545**, anomeric protection via allyloxylation gave a mixture of separable anomers **547**, and the α-anomer was deacetylated to allow oxidation with 2,2,6,6-tetramethyl-1-piperidinyloxy (TEMPO) and (diacetoxyiodo)benzene (BAIB) to the alluronic acid **549**, which was isolated as its methyl ester **534**. Alternatively, deoxyiodination led to **551**, which was subsequently reduced with tris(trimethylsilyl)silane (TTMSS) under 2,2′-azobis(2-methylpropionitrile) (AIBN) initiation to give the 6-deoxy-trifluorallopyranoside **552**.^295^

#### 4.3.2 Hexafluorinated at Positions 2,3,4

**Scheme 79:**
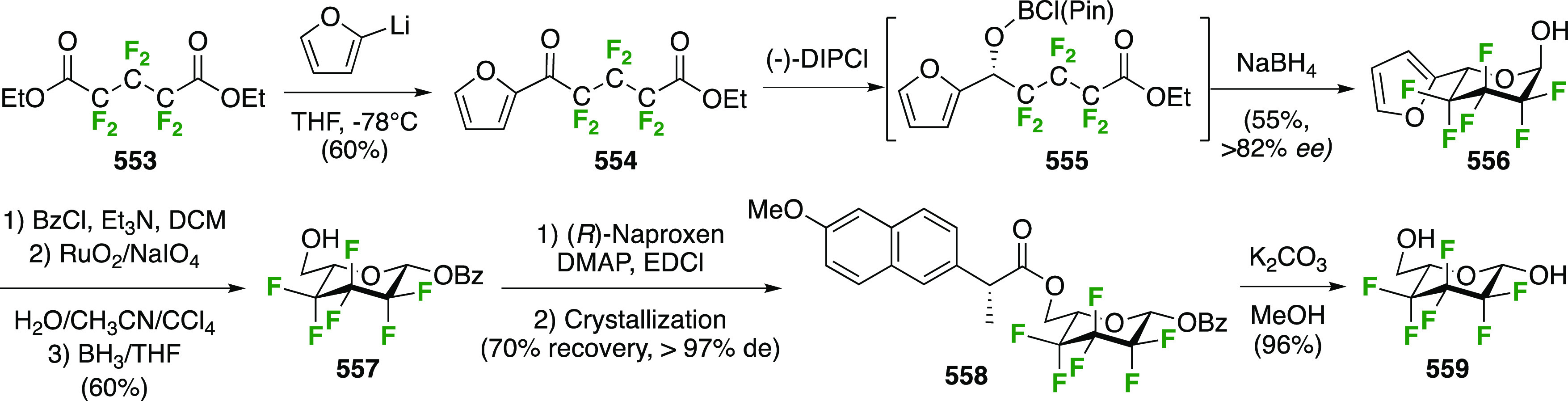
Synthesis of the 2,2,3,3,4,4-Hexafluorinated Sugar Derivative^50,51^

DiMagno reported an enantioselective *de novo* approach for the synthesis of a hexafluorinated sugar derivative, starting from the commercially available fluorinated building block **553**, here illustrated with the l-sugar derivative **559** (Scheme 79).^50,51^ Diethyl hexafluoroglutarate **553** was reacted with 1 equiv of furanyl lithium, leading to the keto ester **554** in 60% yield. Enantioselective reduction of the keto group using (−)-DIPCl resulted in the intermediate **555**, the ester group of which was directly reduced with NaBH_4_ to the corresponding aldehyde, causing cyclization to give the lactol **556** in good yield. After anomeric protection, the aromatic moiety was oxidized to a carboxylic acid, which was selectively reduced to the primary alcohol in **557**. The moderate enantioselectivity required further resolution, which was achieved by various crystallizations of the (*R*)-Naproxen derivative **558**. Finally, methanolysis of both ester groups gave the l-sugar derivative **559**. The synthesis with (+)-DIPCl and (*S*)-Naproxen was shown to lead to the corresponding d-sugar derivative.

### 4.4 Fluorination at Positions 2,3,6

**Scheme 80:**
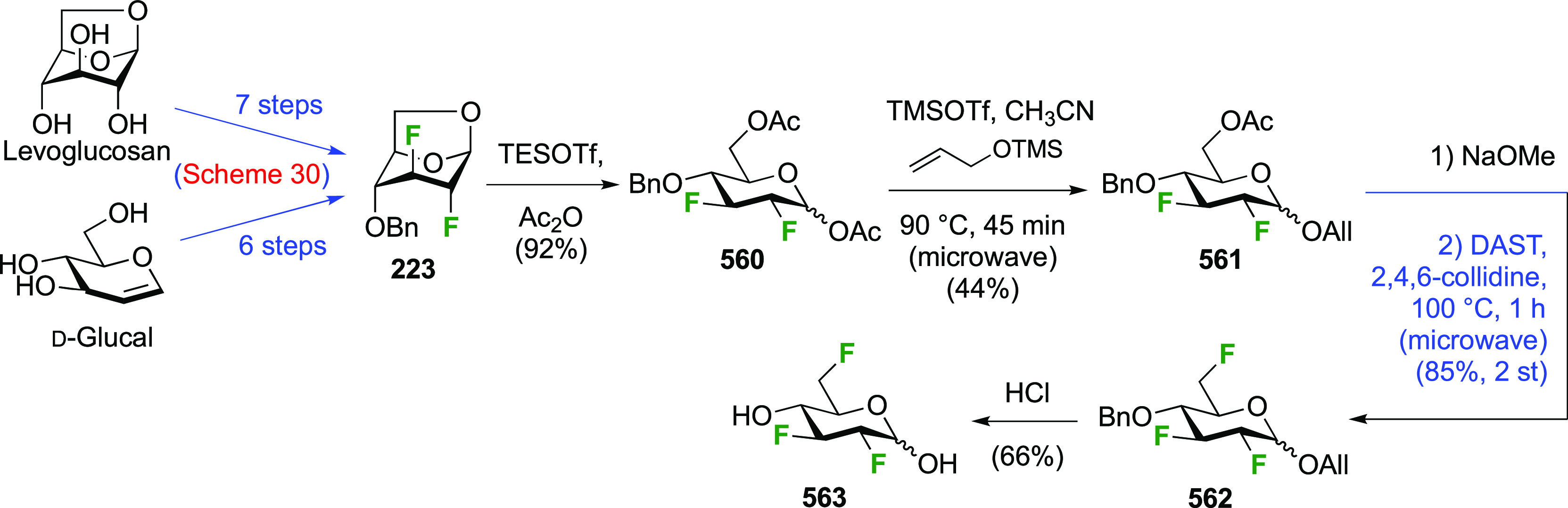
Synthesis of 2,3,6-Trideoxy-2,3,6-trifluoro-d-glucopyranose^272^

The synthesis of 2,3,6-trideoxy-2,3,6-trifluoro-d-glucopyranose **563**, by the Giguère group, involved the advanced intermediate **223** (Scheme 80), already described for the synthesis of 2,3-difluorinated sugars (cf. Scheme 30).^272^ Acetolysis of the 1,6-anhydro-bridge without cleaving the benzyl ether was achieved with TESOTf as catalyst, leading to **560**. Differentiation of the two acetate groups in **560** was possible with a TMSOTf-catalyzed anomeric allylation to **561**, which allowed acetate removal and fluorination at the 6-position to give **562**. Anomeric deprotection via acid hydrolysis then led to **563**.^272^

### 4.5 Fluorination at Positions 2,4,6

**Scheme 81:**
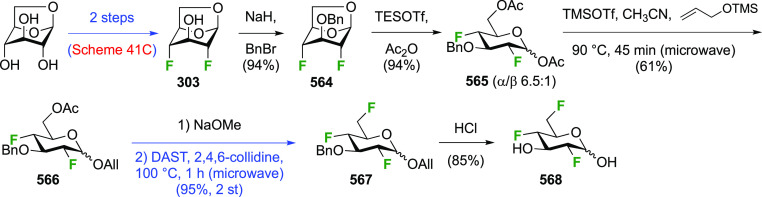
Synthesis of 2,4,6-Trideoxy-2,4,6-trifluoro-d-glucopyranose^272^

The synthesis of the 2,4,6-trifluorinated glucose derivative **568** followed the same strategy as described above for the 2,3,6-derivative, and started from **303** (Scheme 81),^272^ itself obtained in two steps from levoglucosan (cf. Scheme 41C). Hence, upon protection of the OH-3 group to **564**, selective acetolysis and anomeric differentiation to **566**, acetate methanolysis allowed deoxyfluorination at the 6-position to give **567**. Deprotection then gave 2,4,6-trideoxy-2,4,6-trifluoroglucopyranose **568** in 85% yield.^272^

### 4.6 Fluorination at Positions 3,4,6

**Scheme 82:**
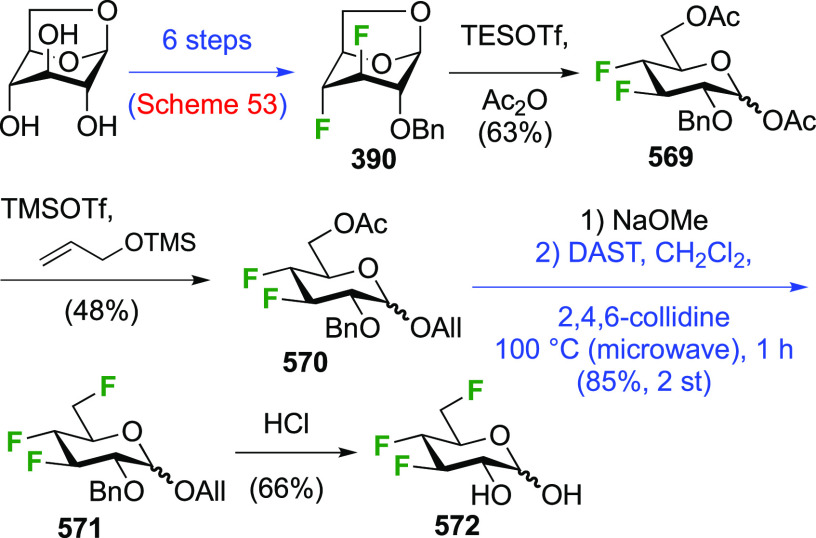
Synthesis of 3,4,6-Trideoxy-3,4,6-trifluoro-α-d-glucopyranose^272^

A synthesis of 3,4,6-trideoxy-3,4,6-trifluoro glucopyranose **572** by Giguère et al. is shown in Scheme 82, which involved the advanced intermediate **390** (cf. Scheme 53).^272^ Triethyl silyl triflate-catalyzed acetolysis of the 1,6-anhydro-bridge in **390** provided compound **569** in 63% yield. After protection of the anomeric center as glycoside **570**, deoxyfluorination at C-6 was achieved with a deprotection–deoxyfluorination sequence, giving compound **571** in 85% yield over two steps. Final deprotection under acidic conditions allowed the formation of product **572**.^272^

## 5 Aldohexoses: Fluorination at Four Positions

### 5.1 Fluorination at Positions 1,3,4,6

**Scheme 83:**
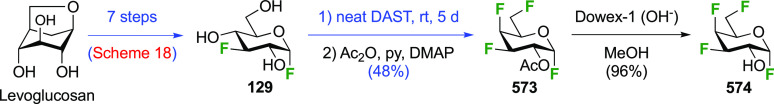
Synthesis of 3,4,6-Trideoxy-3,4,6-trifluoro-α-d-glucosyl Fluoride^59^

The Sidhu group at Monsanto published a synthesis of the first tetradeoxy-tetrafluorinated sugar derivative with 3-deoxy-3-fluoro-α-d-glucosyl fluoride **129** (Scheme 83), discussed above in Scheme 18, as a key intermediate.^59^ Treatment of **129** with neat DAST and subsequent acetylation gave **573** in 48% yield. Deprotection gave the desired 3,4,6-trideoxy-3,4,6-trifluoro-α-d-galactopyranosyl fluoride **574** in 96% yield.^59^

### 5.2 Fluorination at Positions 2,3,4,6

**Scheme 84:**
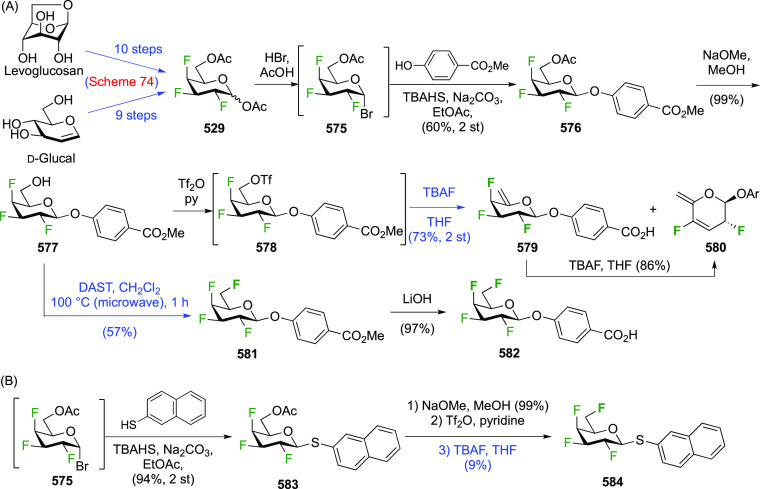
Synthesis of 2,3,4,6-Tetradeoxy-2,3,4,6-tetrafluoro-α-d-galactopyranoside Derivatives^273,275^

The Giguère group published a synthesis of 2,3,4,6-tetradeoxy-2,3,4,6-tetrafluoro-α-d-galactopyranoside derivatives (Scheme 84),^273,275^ which involved the advanced intermediate **529** (discussed above in Scheme 74A). To achieve C-6 fluorination, an aryl group was first installed to block the anomeric position. The α-galactosyl bromide **575** was slowly generated (2 days) using an excess of hydrogen bromide in acetic acid from **529**. Treatment of **575** with methyl *p*-hydroxybenzoate gave the β-galactoside **576**. Deprotection at position 6 was now possible to give **577**. Deoxyfluorination via the corresponding triflate **578** proved difficult, with elimination to **579** and to **580** being the major reaction pathways.^275^ Only a trace amount of desired tetrafluorinated product was detected. The doubly eliminated **580** was easily obtained from **579**. However, a DAST-mediated deoxyfluorination generated 2,3,4,6-tetradeoxy-2,3,4,6-tetrafluorohexopyranoside **581** in 57% yield.^273,275^ The benzoate aglycone was ultimately transformed into the corresponding carboxylic acid **582** with the use of aqueous 1 M LiOH solution.

A similar synthesis was employed for 2,3,4,6-tetradeoxy-2,3,4,6-tetrafluoro-α-d-thiogalactopyranoside **584** (Scheme 84B). The aglycone was installed using the same strategy as before, leading to compound **583** via bromide **575**, followed by de-*O*-acetylation. Due to the instability of the thionaphthyl moiety under the DAST-mediated deoxyfluorination conditions, the triflation method needed to be applied, which gave **584** in 9% yield. The major side product of this transformation was the elimination of the C-6 leaving group as explained above.^273^

**Scheme 85:**
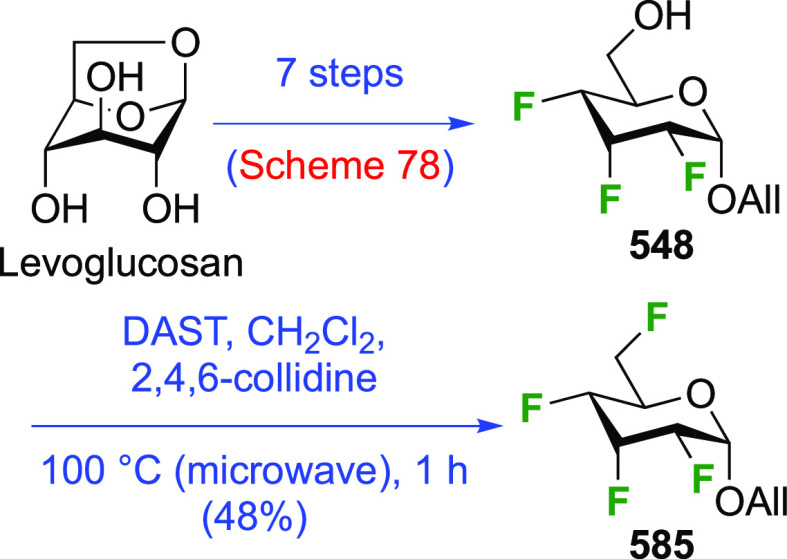
Synthesis of the 2,3,4,6-Tetradeoxy-2,3,4,6-tetrafluoro-α-d-allopyranoside Derivative^295^

The tetrafluorinated allose derivative **585** (Scheme 85) was synthesized from the advanced intermediate **548**, the synthesis of which was described in Scheme 78, by deoxyfluorination.^295^

## 6 Pentoses: Two Hydroxyl Groups Replaced by Fluorine

### 6.1 Fluorination at Positions 1 and 2

**Scheme 86:**
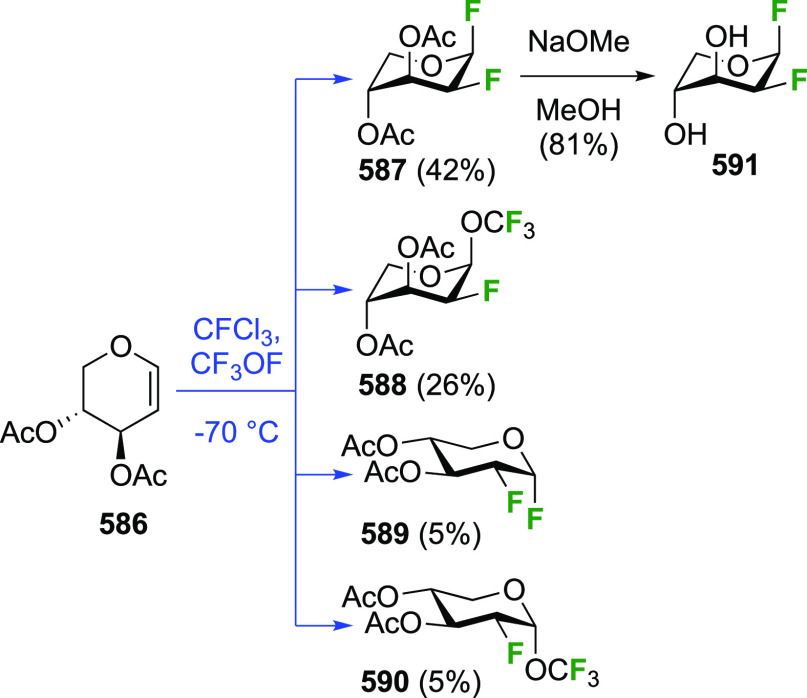
Synthesis of 2-Deoxy-2-fluoro-β-d-lyxo and α-d-Xylohexapyranosyl Fluorides from d-Xylal^191,341^

The Dwek group reported that the reaction of 3,4-di-*O*-acetyl-d-xylal **586** (Scheme 86) with fluoroxytrifluoromethane led to 3,4-di-*O*-acetyl-2-deoxy-2-fluoro-β-d-lyxopyranosyl fluoride **587** and 2-deoxy-2-fluoro-α-d-xylopyranosyl fluoride **589** in 42% and 5% yield, respectively, alongside their trifluoromethyl glycosides **588** and **590**.^191^ Deprotection of **587** gave 2-deoxy-2-fluoro-α-d-lyxopyranosyl fluoride **591** in 81% yield.^341^ The α-d-*lyxo* configured **587**, **588**, and **591** were all found to exist in the ^1^*C*_4_ conformation, and the xylose derivatives **589** and **590** in the ^4^*C*_1_ conformation.

**Scheme 87:**
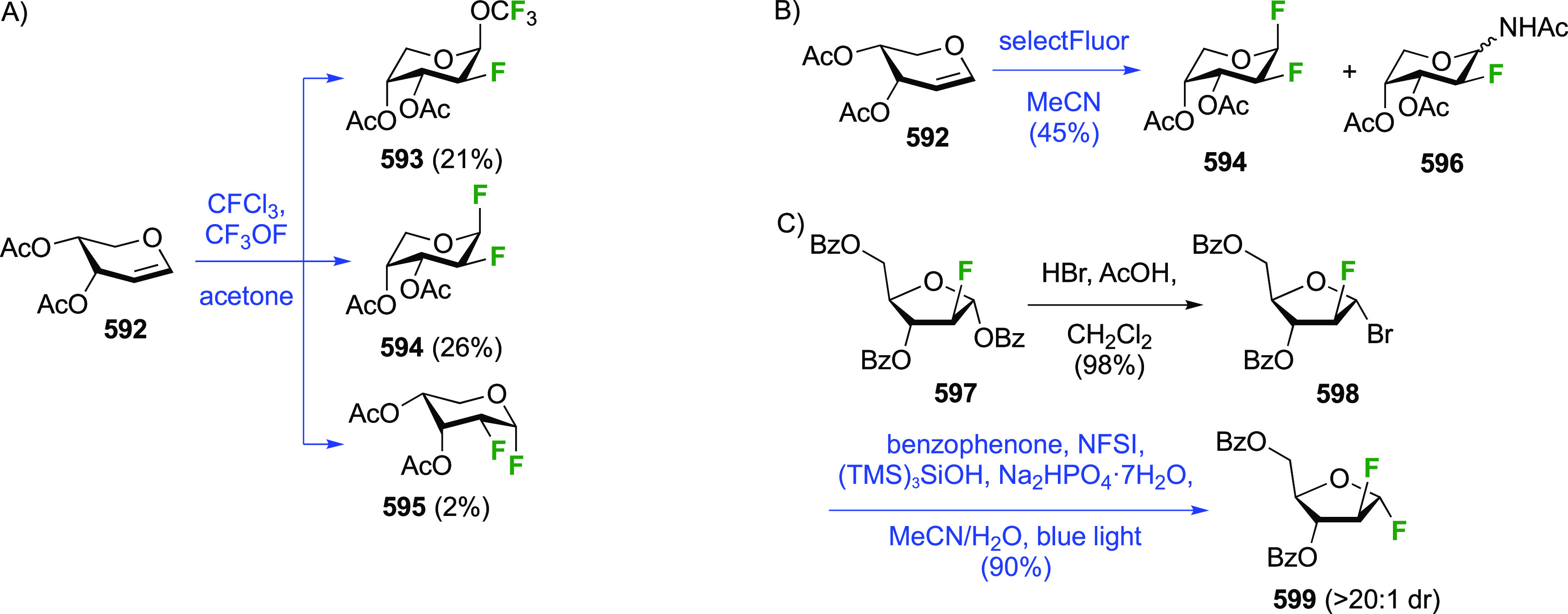
Synthesis of 2-Deoxy-2-fluoro-d-arabinopyranosyl and -furanosyl Fluoride^191,194,342,344^

Starting from 3,4-di-*O*-acetyl-d-arabinal **592** (Scheme 87A), 3,4-di-*O*-acetyl-2-deoxy-2-fluoro-β-d-arabinopyranosyl fluoride **594** and its corresponding trifluoromethyl glycoside **593** were isolated,^191,342^ with 3,4-di-*O*-acetyl-2-deoxy-2-fluoro-α-d-ribopyranosyl fluoride **595** as a minor product.^342^ Interestingly, both the fluorination of d-xylal **586** and d-arabinal **592** is thus reported to occur via the β-face, regardless of the configuration at C-3. This is consistent however with the outcome of the reaction of **586** and **592** with acetyl hypofluorite as reported by Dax et al. (not shown).^343^ The Dax group also reported a significantly improved synthesis of **594** by reaction of **592** with SelectFluor (Scheme 87B), which only gave d-arabino configured **594**, alongside an undisclosed amount of **596**, formed via Ritter reaction with the solvent.^194^ Finally, the McMillan group demonstrated the conversion of glycofuranosyl bromide **598** to 3,5-di-*O*-benzoyl-2-deoxy-2-fluoro-α-d-arabinofuranosyl fluoride **599** via a radical-mediated halogen atom abstraction and benzophenone photosensitization involving *N*-fluorobenzenesulfonimide (NFSI), with excellent yield and stereoselectivity.^344^ Compound **598** can be obtained in one step from the commercially available 1,3,5-tri-*O*-benzoyl-2-deoxy-2-fluoro-α-d-arabinofuranose **597**.^345^

**Scheme 88:**
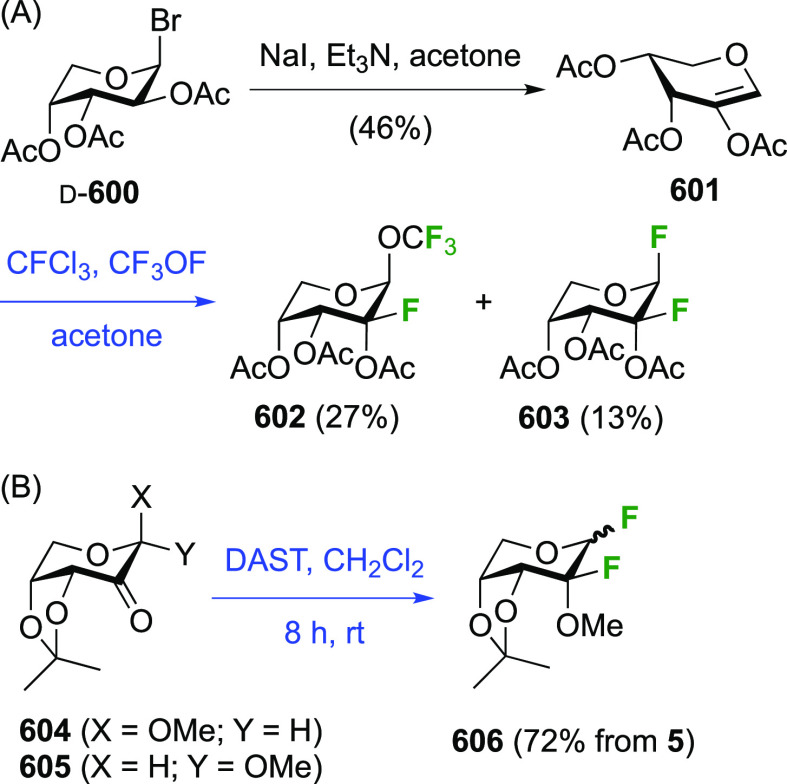
Synthesis of 2,3,4-Tri-*O*-acetyl-2-fluoro-β-d-ribopyranosyl Fluoride Derivatives^203,342^

Fluorination of 2-acetoxy-d-arabinal **601** (Scheme 88A), synthesized from **600** via bromide elimination, was also shown to proceed from the β-face, leading to 2,3,4-tri-*O*-acetyl-2-fluoro-β-d-ribopyranosyl fluoride **603**, albeit as the minor product.^342^ The same types of compounds could be obtained by a DAST-mediated rearrangement process (cf. also Scheme 15C): treatment of **604** with DAST (Scheme 88B) led to **606** as a mixture of anomers.^203^ The same outcome—with a different anomeric ratio—was observed starting from **605**, which has an equatorial OMe group, which was explained by the facile ring inversion of this *cis*-fused ring system.

### 6.2 Fluorination at Positions 1 and 3

**Scheme 89:**
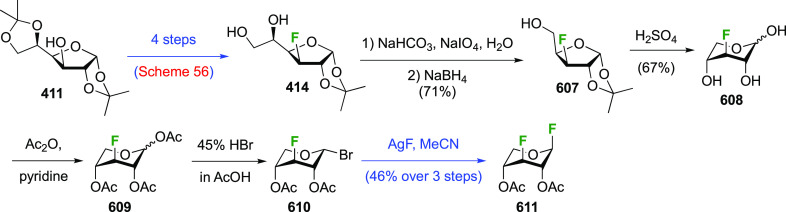
Synthesis of 2,4-Di-*O*-acetyl-3-deoxy-3-fluoro-β-d-xylopyranosyl Fluoride^210^

The Hall and Foster groups published the synthesis of 2,4-di-*O*-acetyl-3-deoxy-3-fluoro-β-d-xylopyranosyl fluoride **611** in three steps from 3-deoxy-3-fluoro-β-d-xylopyranoside **608** (Scheme 89).^210^ This compound was synthesized in seven steps from glucose diacetonide **411** involving advanced intermediate **414** (cf. Scheme 56), by its treatment with sodium periodate and sodium borohydride to give 3-deoxy-3-fluoro-1,2-*O*-isopropylidene-α-d-xylofuranose **607**, upon which hydrolysis of the 1,2-acetonide led to 3-deoxy-3-fluoro-β-d-xylopyranose **608**.^213^ Peracetylation followed by anomeric bromination afforded **610**, upon which anomeric fluorination gave the desired 2,4-di-*O*-acetyl-3-deoxy-3-fluoro-β-d-xylopyranosyl fluoride **611** in 46% yield over three steps.

**Figure 4:**
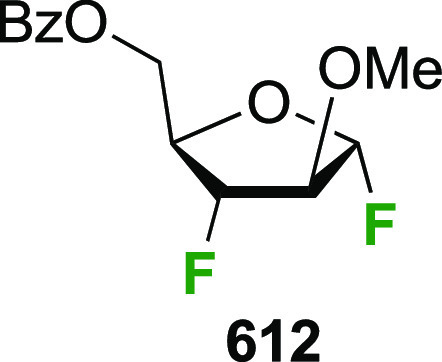
Structure of 5-*O*-benzoyl-3-deoxy-3-fluoro-2-*O*-methyl-α-d-arabinofuranosyl fluoride.^346^

Sivets et al. reported the formation of the 1,3-difluorinated arabinose derivative **612** (Figure 4) as a byproduct of a deoxyfluorination reaction (see Scheme 103 below), which was however fully characterized.^346^

**Scheme 90:**
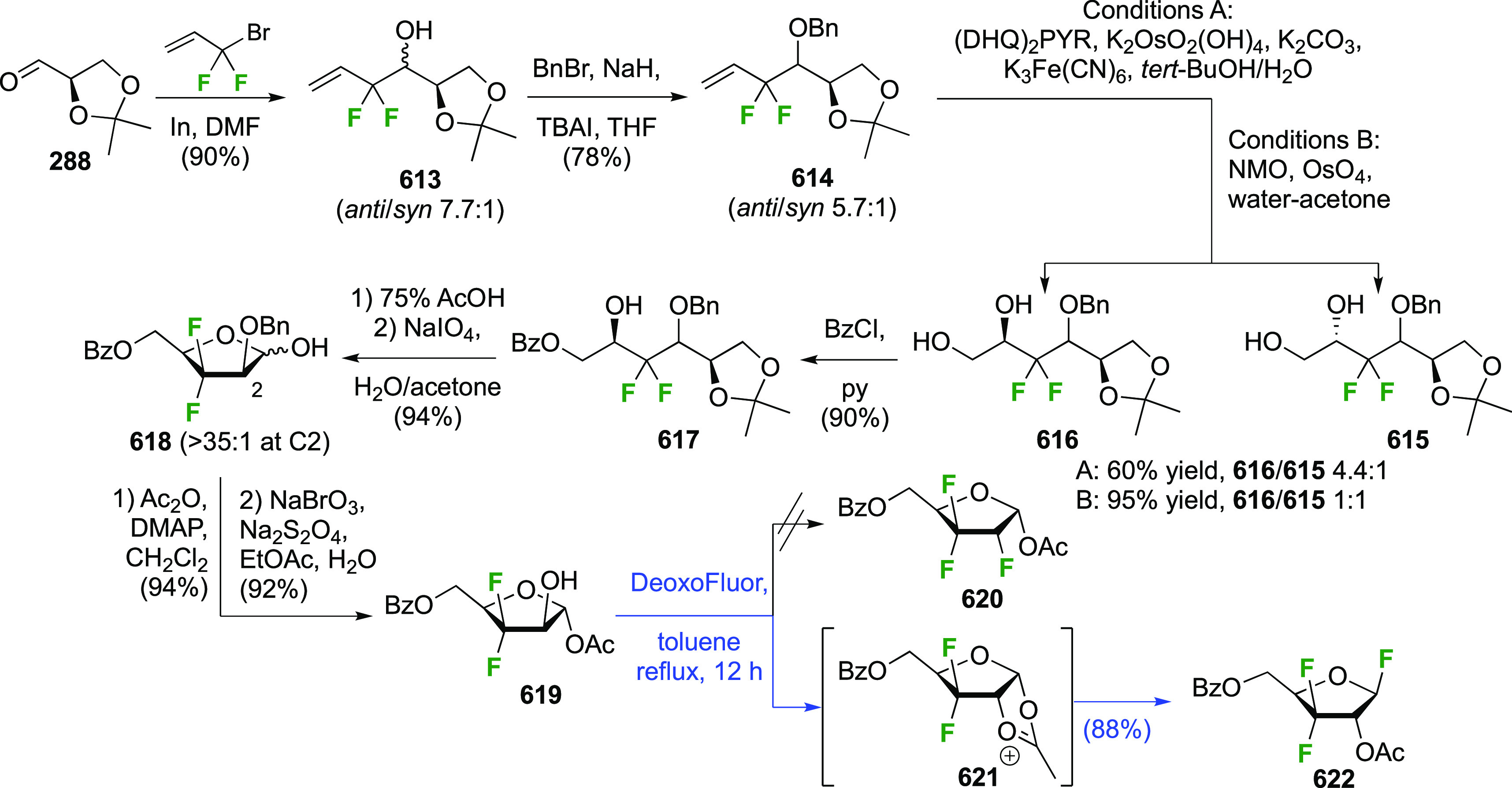
Synthesis of 3-Deoxy-3,3-difluoro-α-d*-erythro*-pentofuranosyl Fluoride^348^

The Qing group published an enantioselective synthesis of a 1,3,3-trifluorinated pentose as donor **622** for nucleoside synthesis (Scheme 90) using a *de novo* synthesis approach starting from glyceraldehyde acetonide **288**.^347,348^ The addition of 1,1-difluoroallyl indium led to **613** in excellent yield in a 7.7:1 diastereomeric ratio in favor of the *anti*-diastereomer, which decreases to 5.7:1 in **614** after benzylation, which was attributed to NaH-mediated epimerization of **614**. Next, the alkene was dehydroxylated. Under Upjohn conditions, a 1:1 ratio of diastereomers at the newly formed stereocenter was obtained, but under Sharpless conditions with (DHQ)_2_PYR, a 4.4:1 ratio was obtained in favor of **616**. After protection of the terminal alcohol to **617**, the acetonide was hydrolyzed and the resulting diol cleaved with periodate, causing the furanose **618** to form in excellent yield. Interestingly, the diastereomeric ratio at C-2 turned out to be >35:1, up from 5.7:1 at the benzyl ether center in **617**. This was attributed to an epimerization process at C-2. Anomeric acetylation followed by debenzylation gave **619**. The enantiomer of **619** was obtained in the same way from **616** (not shown).^348^ Attempted OH-2 deoxyfluorination to give **620** failed. Instead, neighboring group participation of the anomeric acetate involving displacement of the activated OH-2 intermediate took place, giving **621**, which then reacted with fluoride to give the β-configured furanosyl fluoride **622**. Nucleoside formation from **622** was successful (not shown).^348^

### 6.3 Fluorination at Positions 1 and 5

**Scheme 91:**
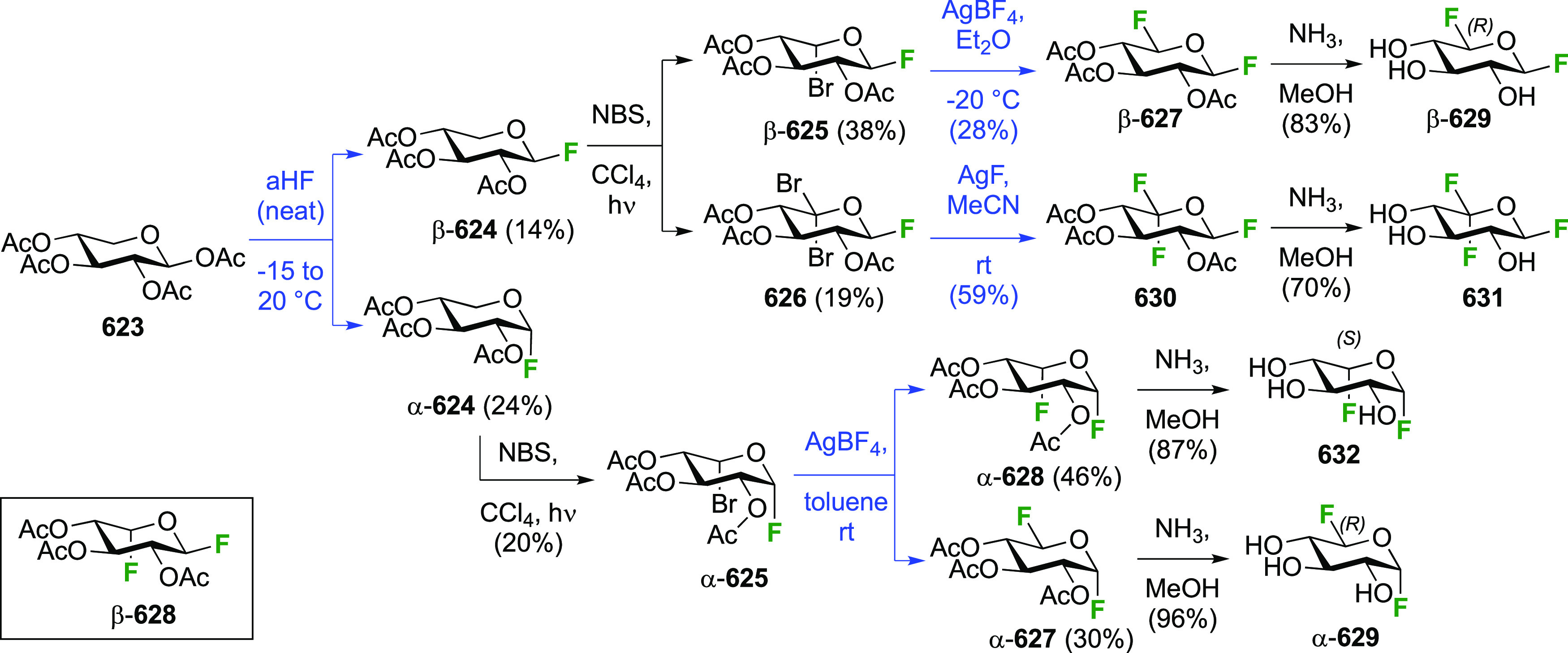
Synthesis of 5-Fluorinated Xylopyranosyl Fluorides^349^

The Withers group reported the synthesis of a number of 5-fluorinated pentopyranosyl fluorides as part of a mechanistic study.^349^ Like the synthesis of 5-fluorinated hexopyranosyl fluoride derivatives (section 3.1), the anomeric fluoride was introduced first. Hence, treatment of β-d-xylose tetra-*O*-acetate **623** (Scheme 91) with neat anhydrous HF led to the formation of both anomeric xylopyranosyl fluorides **624**, which were separable.^350^ Using Olah’s reagent (HF-py), this reaction was reported to only lead to the α-anomer in 95% yield (not shown).^95^ From β-**624**, radical bromination was selective for the 5-position, with the formation of the axial bromide β-**625** as the only monobrominated product, alongside the 5,5-dibrominated xylose derivative **626**. Fluorination of β-**625** led to a mixture of β-**627** and β-**628**, but only the desired β-**627** was isolated. Deprotection furnished (5*R*)-5-fluoro-β-d-xylopyranosyl fluoride β-**629**. Fluorination from **626** led to **630** in good yield, and deprotection then gave 5,5-difluoro-β-d-xylopyranosyl fluoride **631**.

From the α-configured xylopyranosyl fluoride α-**624**, radical bromination only gave the monobrominated α-**625**. Treatment with AgBF_4_ in toluene now gave the two fluoride epimers at C-5 α-**627** and α-**628**. After separation, their deprotection gave (5*R*)-5-fluoro-α-d-xylopyranosyl fluoride α-**629** and (5*S*)-5-fluoro-α-d-xylopyranosyl fluoride **632**.^349^

### 6.4 Fluorination at Positions 2 and 3

The chemistry of 2,3-dideoxy-2,3-difluoropentoses is intimately linked with that of its nucleosides. Hence, nucleoside chemistry is included, although the emphasis is not on nucleobase introduction, but on establishing the 2′,3′-fluorination pattern. Where applicable, approaches that introduce both fluorine before nucleobase introduction will be mentioned first.

#### 6.4.1 Difluorinated at Positions 2 and 3

##### 6.4.1.1 Riboconfigured

**Scheme 92:**
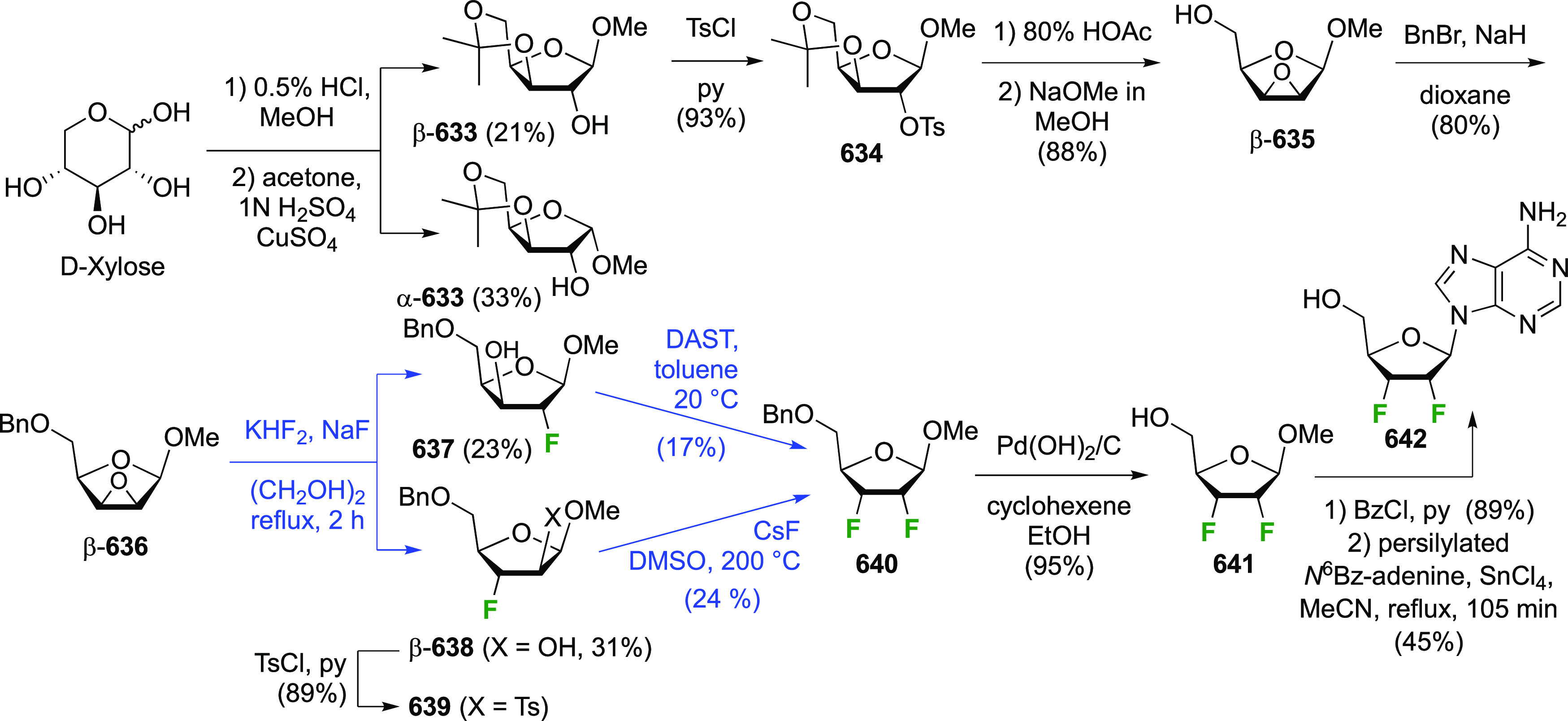
Convergent Synthesis of a 2,3-Dideoxy-2,3-difluorinated Ribose Derivative Using a Sequential Fluorination Approach^351^

The synthesis of a 2,3-dideoxy-2,3-difluorinated ribose derivative **641** was described by Mikhailopulo, with subsequent transformation to an adenosine analogue (Scheme 92).^351^ Treatment of d-lyxose with 0.5% HCl in methanol resulted in methyl lyxofuranoside formation, which was protected as its acetonide followed by separation of the anomers **633**.^352^ Tosylation of the OH-2 group to **634**(353) and acetonide hydrolysis led to the 2,3-anhydro lyxofuranoside derivative β-**635**. An improved large-scale synthesis of β-**635** and its α-anomer, in which a 93% yield was obtained for the conversion of xylose to **633**, and which were not separated until after epoxide formation, is available.^354^ Protection of the remaining alcohol in β-**635** then led to benzyl ether β-**636**.^353,355,356^ Epoxide opening with fluoride resulted in the formation of the 2-fluorinated xylose derivative **637** in 23% yield, and in the 3-fluorinated arabinose derivative β-**638** in 31% yield.^355^ However, the De Clercq group reported that in their hands, only β-**638** was obtained, also in 31% yield.^356^ Tosylation of β-**638** to give **639**(356) allowed for displacement with fluoride, giving **640** in 24% yield.^351^ Direct deoxyfluorination of **637** gave **640** in 17% yield.^351^ The low yields can be attributed to the congested environment, with unfavorable dipole interactions. Benzyl hydrogenolysis then gave methyl 2,3-dideoxy-2,3-difluoro-d-ribofuranoside **641**. Finally, glycosylation of adenine was achieved after benzoyl protection, giving **642**, with only the formation of the β-anomer reported.

**Scheme 93:**
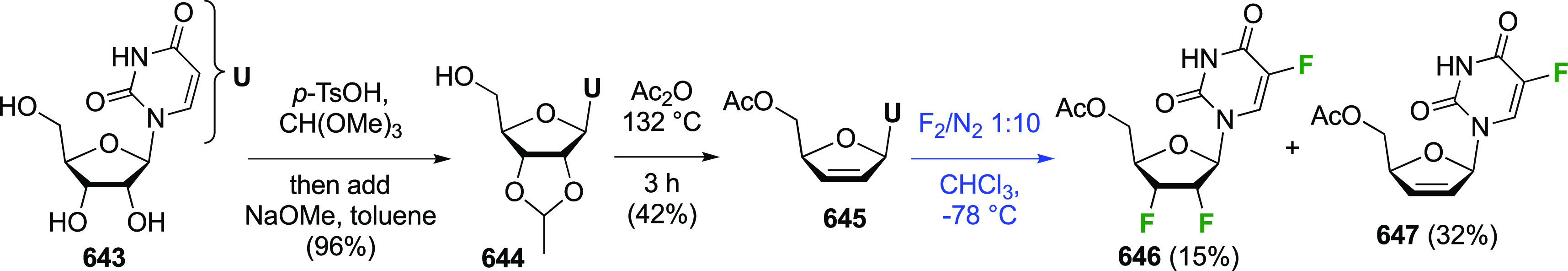
A Direct Fluorination Approach to Give 5-Fluoro-2′,3′-dideoxy-2′,3′-difluoro uridine^357^

A number of nucleosides based on 2′,3′-dideoxy-2′,3′-difluoro-d-ribofuranose have been prepared with at least one fluorine introduction achieved after nucleobase introduction. The most direct method was reported by Coe et al.^357^ Uridine **643** was converted to the 2,3-dehydro derivative **645** (Scheme 93) via the corresponding ethylidene acetal intermediate **644**.^358^ Reaction of **645** with diluted fluorine gas resulted in diastereoselective vicinal *syn*-fluorination at the furanose double bond to give the *ribo*-configured **646**, but the uracil moiety was also fluorinated at the 5-position. Presumably the uracil double bond was also difluorinated, followed by a fluoride elimination. The byproduct of this reaction was **647**, suggesting fluorination at the uracil ring was the fastest process.

**Scheme 94:**
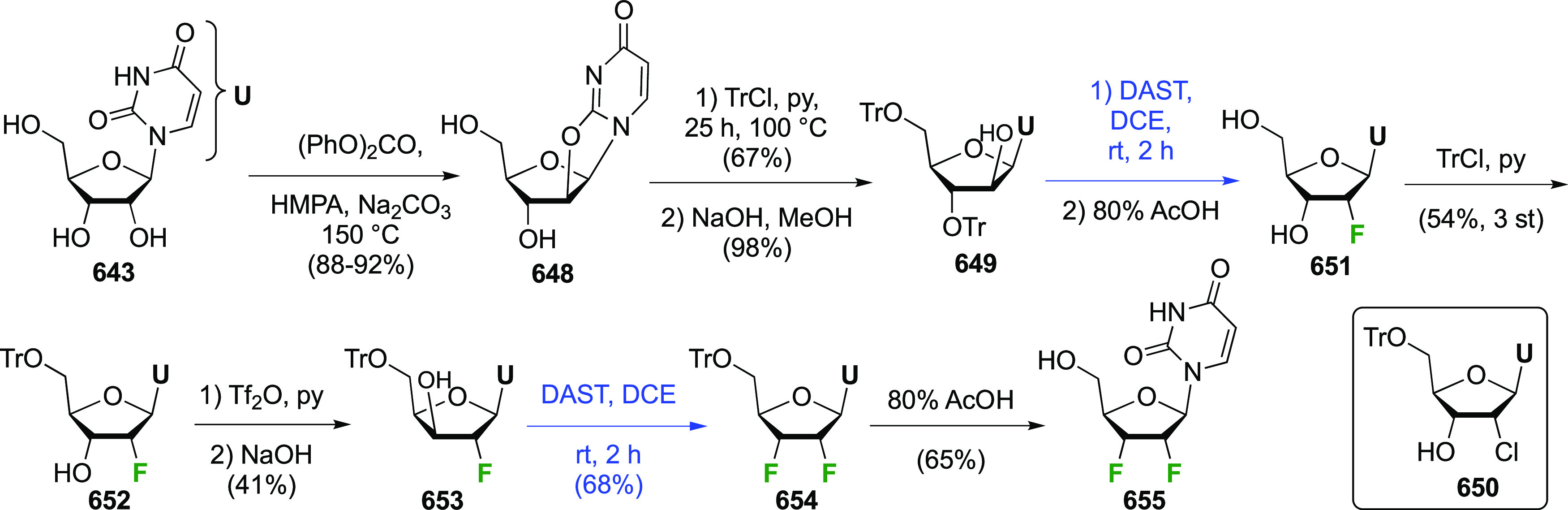
Synthesis of 2′,3′-Dideoxy-2′,3′-difluoro Uridine Starting from Uridine Using DAST-Mediated Sequential Fluorination^359^

The Herdewijn group also reported a synthesis of 2′,3′-dideoxy-2′,3′-difluoro uridine starting from uridine **643** (Scheme 94), which involved two successive alcohol inversions before deoxyfluorination.^359^ Inversion at C-2 was achieved by conversion to the 2,2′-anhydro uracil derivative **648**.^360^ This allowed protection of the remaining alcohol groups as trityl ethers, which required forcing conditions. Anhydro opening with hydroxide then resulted in inversion at C-2 to give d-arabinofuranosyluracil **649**.^361^ Interestingly, 2′-deoxy-2′-chlorouridine **650**, resulting from opening of the anhydro group by the chloride that was released upon trityl protection, was initially reported as the main product of this sequence.^362^ From **649**, DAST-mediated deoxyfluorination installed the F-2 group, and a detritylation-selective OH-5 tritylation sequence then gave **652** with the OH-3 available for reaction. Inversion of configuration was achieved with a triflation, hydroxide displacement sequence, leading to the 2-fluoro-d-*xylo* derivative **653**. The second DAST-mediated deoxyfluorination produced **654** in a much higher yield compared to the equivalent DAST reaction of the OH-3 in **637**, despite the mild conditions and short reaction time (see Scheme 92). This was followed by deprotection which then gave the desired 2′,3′-dideoxy-2′,3′-difluoro uridine **655**, which in fact was the first synthesis of a difluororibose based nucleoside.^359^ A similar synthesis of 2′,3′-dideoxy-2′,3′-difluoro thymidine, with similar yields, was also reported by the same group.^363^

**Scheme 95:**
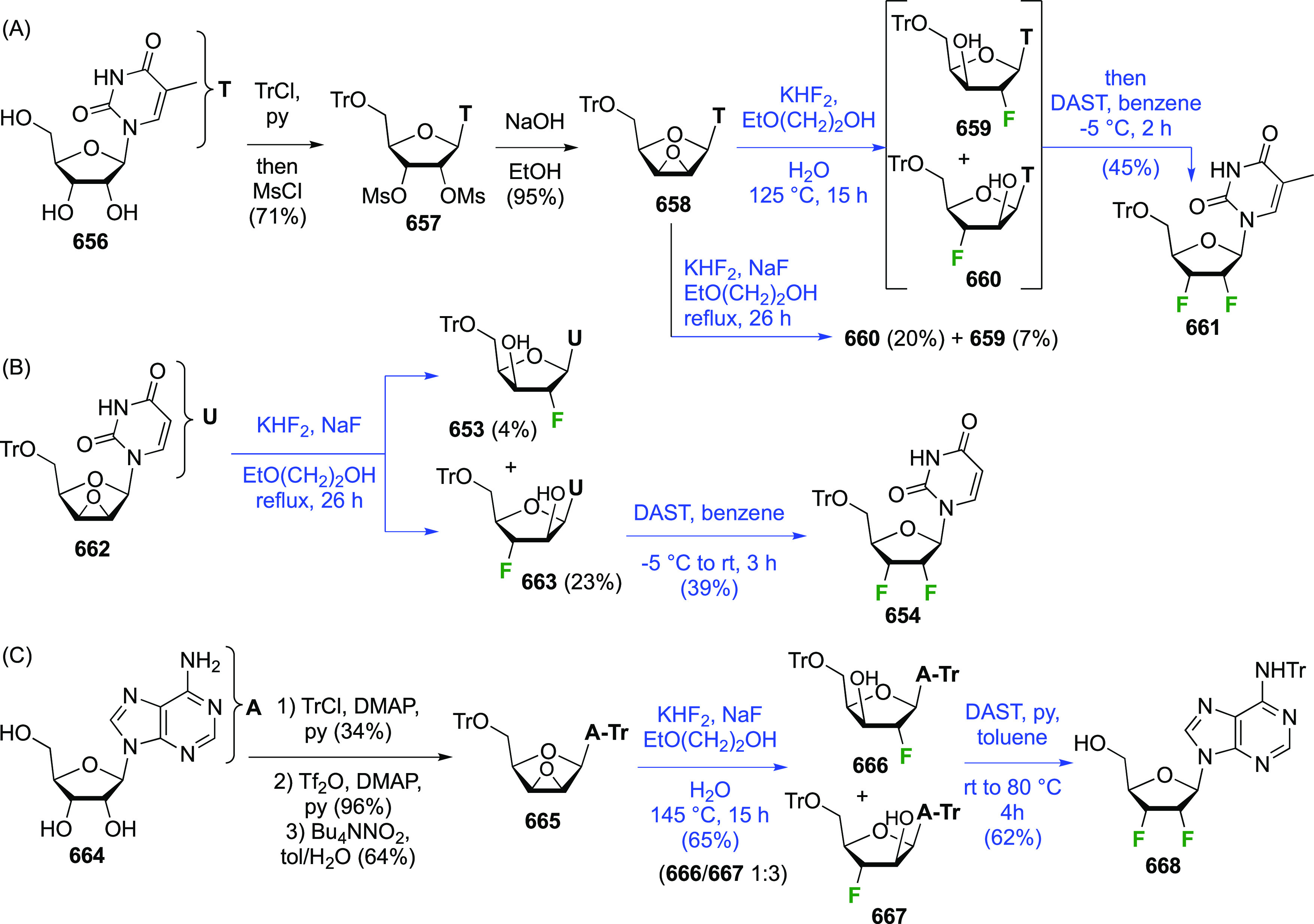
Linear Synthesis of 2′,3′-Dideoxy-2′,3′-difluorinated Ribonucleosides Starting from Nucleosides Using Sequential Fluorination via Epoxide Opening^364,368,369^

A number of groups have described the synthesis of 2,3-dideoxy-2,3-difluororibose-based nucleosides from their parent ribonucleosides via a 2,3-anhydro approach (cf. Scheme 92). The Watanabe group was the first to demonstrate this short synthesis method (Scheme 95A).^364^ Thymidine **656** was protected at the primary alcohol, and dimesylated to give **641**. Treatment with hydroxide, in which an intermolecular displacement was followed by cyclization, led to the 2,3-*lyxo*-configured anhydro nucleoside **658**. Opening with fluoride was not regioselective, but separation of the regioisomers **659** and **660** was not required as the subsequent DAST-mediated deoxyfluorination led to the same product **661** in an overall 45% yield. The Kumar group also reported the fluoride opening of **658**, which was synthesized in a similar way as shown here,^365^ with isolated yields of 20% and 7% for **660** and **659**.^366^ The opening at the 3′-position was found to predominate, which was explained for 2,3-*lyxo*-epoxides by the influence of the electron withdrawing effect of the anomeric center.^367^ This was also illustrated for the *lyxo*-anhydro uridine derivative **662** with the 3-fluoroarabino nucleoside **663** as the major product (Scheme 95B),^366^ which was further deoxyfluorinated to give tritylated 2′,3′-dideoxy-2′,3′-difluoro uridine **654**.^368^ This C-2-deoxyfluorination proceeded in lower yield than the corresponding C-3-deoxyfluorination as shown with **653** in Scheme 94.

Finally, the synthesis of a (protected) 2′,3′-dideoxy-2′,3′-difluorinated adenosine with the epoxide strategy was shown by the Aldrich group (Scheme 95C).^369^ The required 2′,3′-anhydro substrate **665** was synthesized from adenosine **664** by tritylation of the ribose OH-5 and the adenine amino group, followed by triflation of both remaining alcohols, and epoxide formation.^370^ Fluoride opening of **665** gave a 3:1 ratio of regioisomers with the 3′-fluoro-*arabino***651** as the expected major product.^369^ These were not separated, and DAST-mediated deoxyfluorination, which was conducted at a higher temperature than that of **663**, with a much better yield as a result, led to the formation of **668** with concomitant detritylation at the OH-5 group (but not of the nucleobase).

**Scheme 96:**
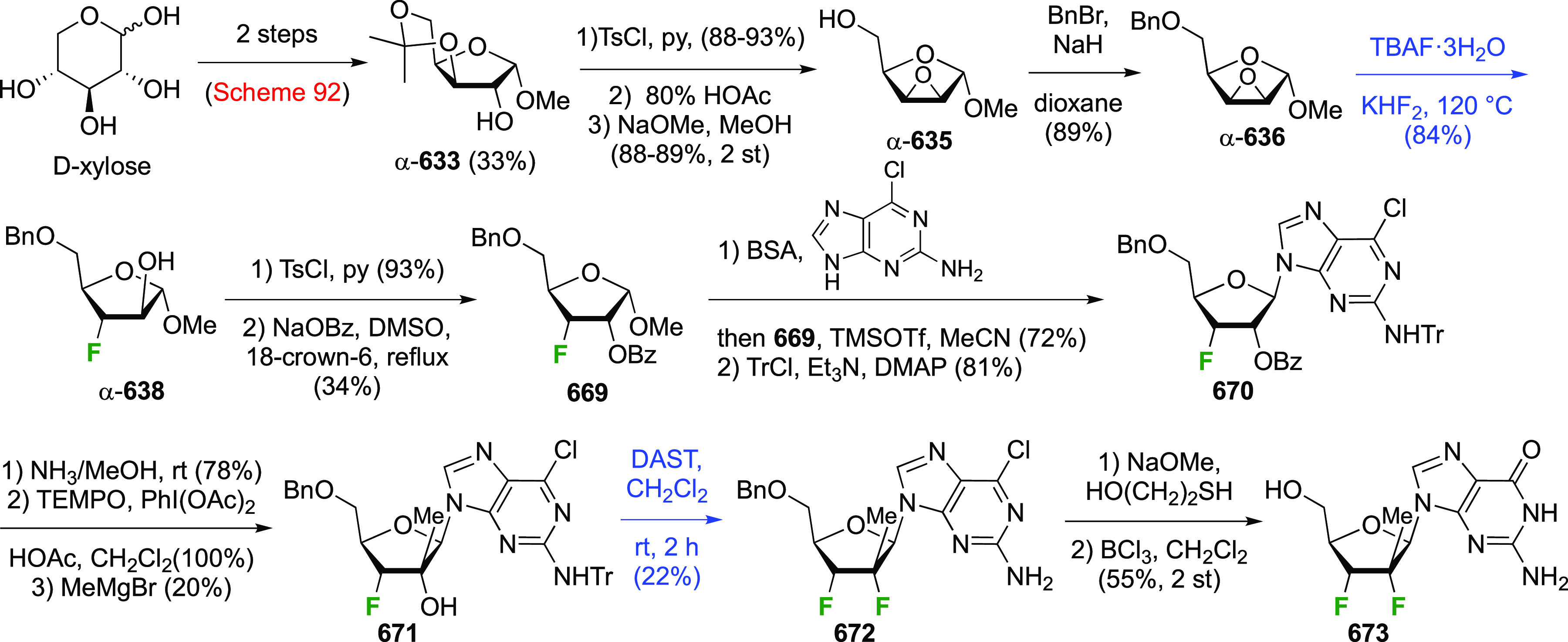
Linear Synthesis of a C-2-Branched 2′,3′-Dideoxy-2′,3′-difluorinated Guanosine Using Sequential Fluorination via Epoxide Opening^371^

Finally, the Dyatkina group at Janssen Biopharma synthesized the branched nucleoside **673** with 2’3′-*ribo*-difluorination (Scheme 96).^371^ Starting from α-**633**, synthesized as shown in Scheme 92,^355^ conversion to the 2′,3′-anhydro derivative α-**635** was achieved via tosylation, acetonide removal, and cyclization, which was then protected as the benzyl ether α-**636**.^353,354,356^ Opening of the epoxide with fluoride now proceeded with complete regioselectivity to give α-**638**,^372^ which will be due to the combined steric and electronic effects of the α-configured anomeric center.^367^ Tosylation of the OH-2 group allowed inversion of configuration with sodium benzoate in moderate yield.^371^ However, this allowed introduction of the nucleobase to isolate the β-nucleoside in 72% yield. Tritylation of the amino group then gave **670**. The benzoate group was removed, the resulting alcohol was oxidized, and the subsequent Grignard reaction afforded **671** as the only reported diastereomer. DAST-mediated deoxyfluorination led to **672** as a single diastereomer, which was converted to 2′,3′-dideoxy-2′,3′-difluoro-2′-*C*-methylguanosine **673**.^371^

##### 6.4.1.2 Lyxo-configured

**Scheme 97:**
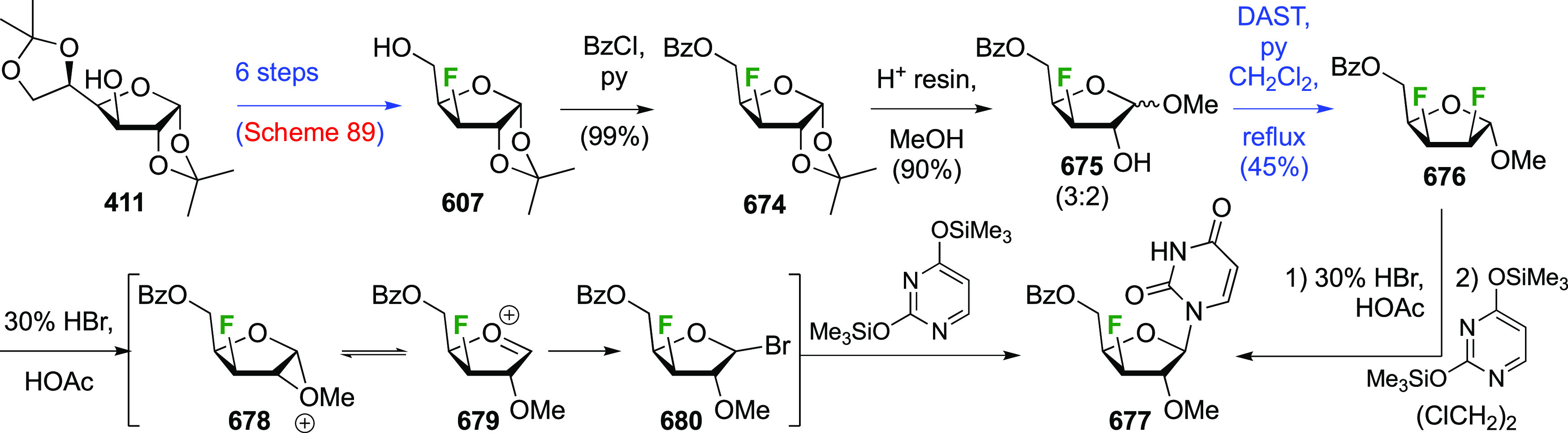
Synthesis of a 2,3-Dideoxy-2,3-difluoro-d-lyxofuranoside Derivative, and Its Unsuccessful Nucleobase Introduction^374^

The synthesis of d*-lyxo*-configured nucleosides was achieved by the Marquez group in 1995.^373^ In a first approach (Scheme 97), the fluorination was planned before nucleoside formation.^374^ Intermediate **607** was obtained from glucose diacetonide **411** in six steps as detailed above in Scheme 89. Protection of the primary alcohol group as the benzoate and acetal methanolysis gave the d-*xylo* derivative **675** as a 3:2 ratio of anomers. This was deoxyfluorinated with DAST to give methyl 5-*O*-benzoyl-2,3-dideoxy-2,3-difluoro-d-lyxofuranoside **676** as a single α-anomer. It was noted that reflux temperature was required to achieve conversion, which was attributed to repulsive dipole–dipole interactions between the *cis*-vicinal fluorines.^143,164−166^ This contrasts with the low/room temperature DAST reactions required to arrive at the *ara*- and even the *ribo*- configurations. Unfortunately, nucleoside introduction attempts with **676** led to decomposition, and with attempts to form the anomeric bromide, a d-*xylo* configured rearrangement product **677** with loss of F-2 was obtained. It was proposed that the electron withdrawing effect of the fluorines hampered reaction at the anomeric center, and that instead neighboring group participation of the antiperiplanar anomeric OMe group facilitated loss of HF, leading to **678**. This could be in equilibrium with the oxonium ion **679**, leading to **680** upon bromide addition. The anomeric configuration of **680** could not be ascertained, but only the β-nucleoside **677** was obtained upon subsequent glycosidation.

**Scheme 98:**
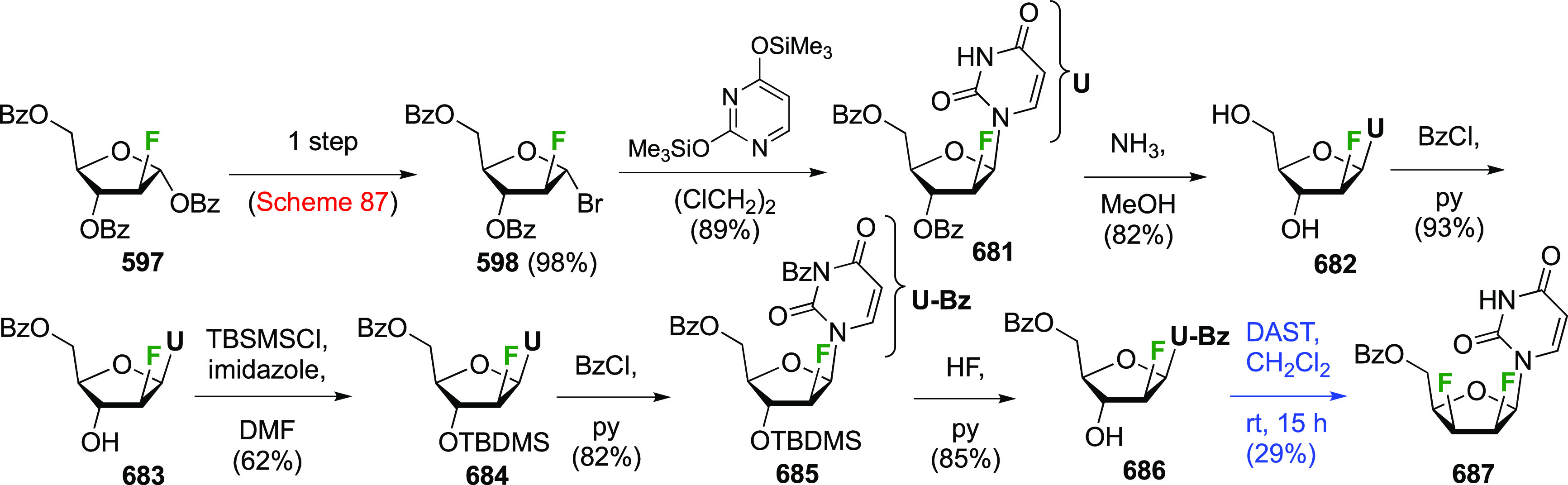
Linear Synthesis of a 2′,3′-Dideoxy-2′,3′-difluorinated d-*lyxo* Configured Nucleoside with Nucleobase Introduction Preceding the Second Fluorination^373^

Hence, the nucleobase was introduced before the second fluorine (Scheme 98).^373^ Uracil introduction with **598** only gave the β-anomer **681**, and benzoyl aminolysis led to **682**.^375^ Selective rebenzoylation at the 5′-position and *tert*-butyl dimethyl silyl (TBDMS) protection of the 3′position, to give **684**, allowed uracil protection as the *N*^3^-benzoyl derivative **685**. This was required to avoid the very facile cyclization of pyrimidine nucleosides with activated *trans*-positioned alcohols at C-2 or C-3, which leads to the corresponding anhydro derivatives. The silyl group was then removed with fluoride, allowing DAST-mediated deoxyfluorination of the 3′OH group with concomitant *N*^3^-benzoyl cleavage to give the d-*lyxo* configured nucleoside **687**. Interestingly, although the yield was low, this DAST reaction proceeded at −40 °C despite the resulting highly congested substitution in **686**, and the dipole repulsion from F-2.

##### 6.4.1.3 Xylo-configured

**Scheme 99:**
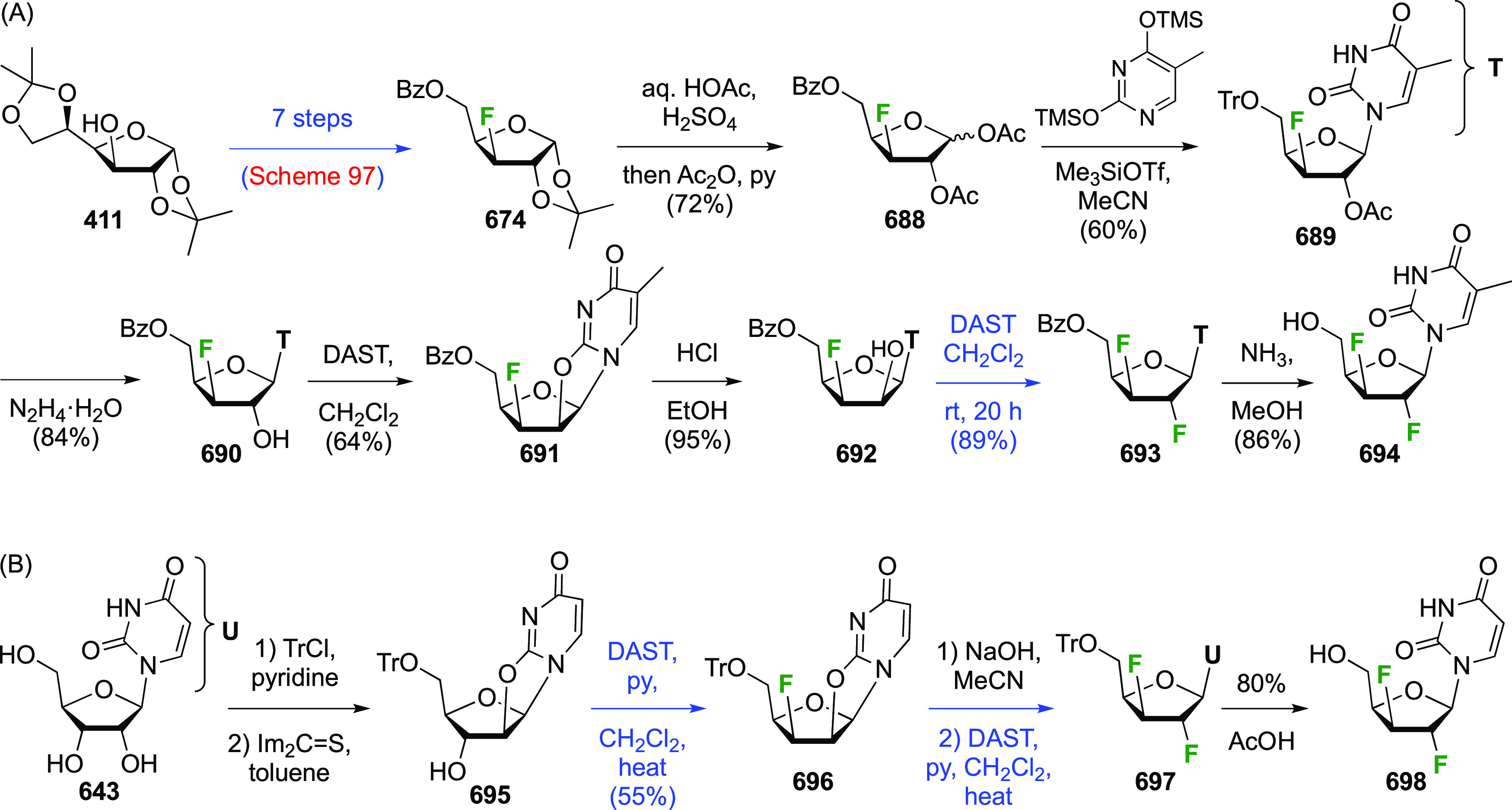
Linear Synthesis of (2′,3′-Dideoxy-2′,3′-difluoro-β-d-xylofuranosyl) Nucleosides via 2,2′-Anhydro Intermediates^376,377^

Gosselin et al. reported the synthesis of 2′,3′-dideoxy-2′,3′-difluoroxylofuranosyl nucleosides starting from glucose diacetonide **411** (Scheme 99A),^376^ the conversion of which to the advanced intermediate **674** is shown in Scheme 97. Direct acetolysis of **674** led to the open chain aldehyde–diacetate as a major byproduct (not shown), but acetonide hydrolysis followed by nucleophilic acetate formation resulted in the desired **688**. Its condensation with silylated thymine under Vorbruggen conditions afforded the nucleoside derivative **689** as the only reported anomer. Acetate hydrazinolysis revealed the OH-2 group, but attempted deoxyfluorination with DAST to arrive at the *ribo*-configured 2′,3′-difluorinated nucleoside failed, because the activated intermediate was intercepted by the (unprotected, cf. Scheme 98 for the relevance of this) thymine carbonyl to give the 2,2′-anhydro derivative **691**. Hydrolysis of the anhydro-bridge then led to the *lyxo*-configured **692**, upon which deoxyfluorination proceeded in excellent yield to give, after benzoate removal, 1-(2′,3′-dideoxy-2′,3′-difluoro-β-d-xylofuranosyl)thymine **694**.^376^

A related, shorter synthesis was reported by the Marquez group (Scheme 99B).^377^ The 5-tritylated 2,2′-anhydrouridine derivative **695** was synthesized in two steps from uridine **643** (no yields were given). The remaining hydroxyl group in **695** was then displaced with fluorine to give **696**, after which the synthesis converged with the Gosselin synthesis: anhydro hydrolysis allows for a second fluorination to the difluorinated *xylo*-derivative **697**, which upon deprotection gave 1-(2′,3′-dideoxy-2′,3′-difluoro-β-d-xylofuranosyl)uracil **698** (no yields were given).^377^

**Scheme 100:**
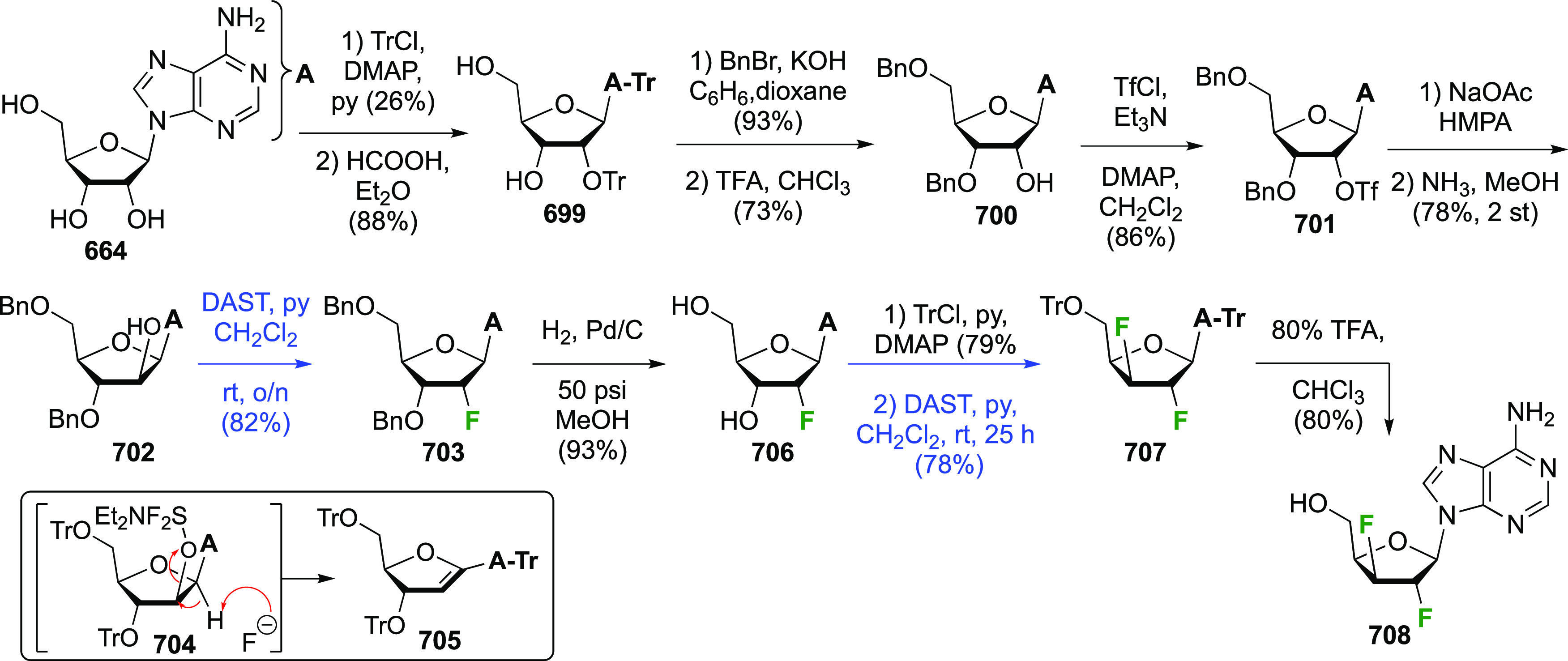
Linear Synthesis of 9-(2′,3′-Dideoxy-2′,3′-difluoro-β-d-xylofuranosyl) Adenine via Sequential Fluorine Introduction of Adenosine^369^

The Aldrich group reported the synthesis of 2′,3′-dideoxy-2′,3′-difluoro-β-d-xylofuranosyl)adenosine **708** starting from adenosine **664** (Scheme 100).^369^ The fluorine at C-2 was introduced first, as published by the Pankiewicz group.^378^ Adenosine was first subjected to tritylation, with the 2,5-di-*O*-trityl protection product isolated in 26% yield. The primary trityl group was selectively cleaved, leading to **699** with concomitant adenine deprotection. Protection of the alcohols as benzyl ethers was followed by removal of the 2-*O*-trityl group. Inversion of configuration at C-2 in **700** via an oxidation–reduction protocol was not successful, and instead an S_N_2 reaction with sodium acetate on the corresponding triflate **701** was carried out. This afforded, after acetate aminolysis, the required *arabino*-configuration in **702**. DAST-mediated deoxyfluorination proceeded in excellent yield, resulting in **703**. Interestingly, when the 3′,5′-hydroxyl groups were protected as trityl ethers, the DAST reaction resulted in the formation of **705** as a side-product (30% yield). The bulky trityl group hampers fluoride approach at C-2, promoting an E2 elimination process involving the antiperiplanar H-1 in **704**. With benzyl protection, elimination was not observed. Benzyl hydrogenolysis gave 2-deoxy-2-fluoroadenosine **706**.^378^ Protection of the primary alcohol, with concomitant adenine amine protection, then allowed deoxyfluorination at C-3 to give **707**. Final deprotection afforded 1-(2′,3′-dideoxy-2′,3′-difluoro-β-d-xylofuranosyl)adenosine **708**.^369^

**Scheme 101:**
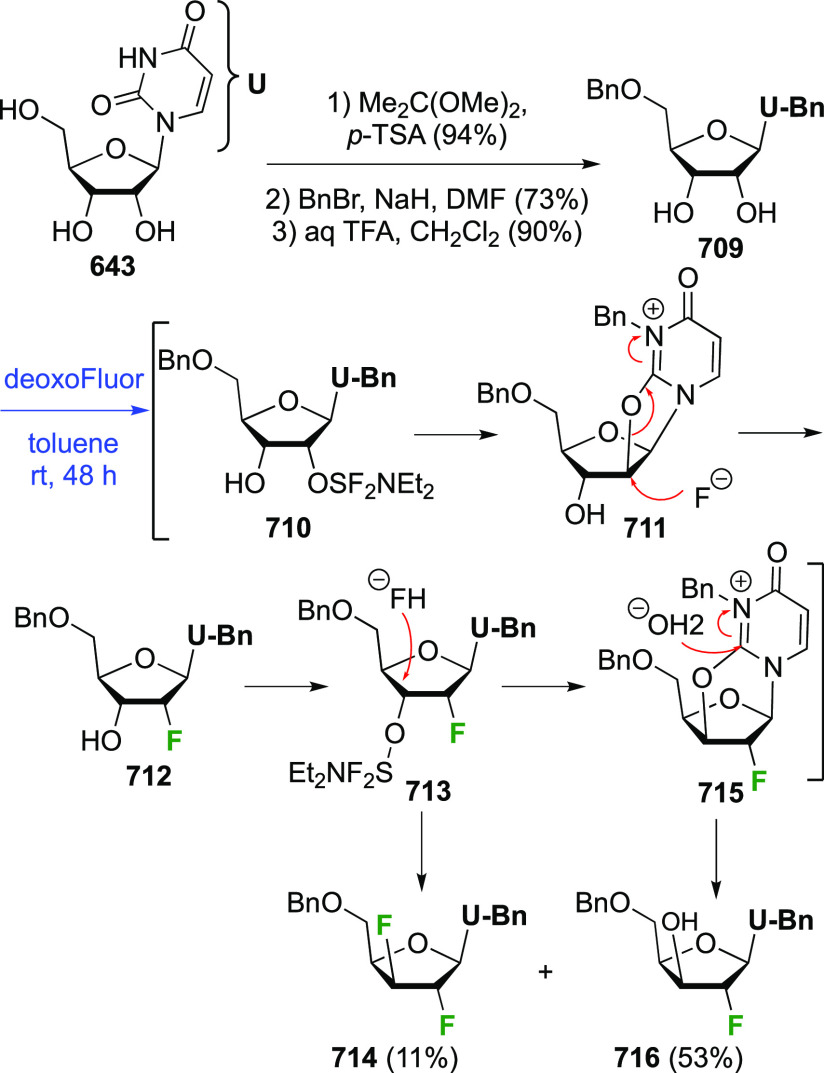
A direct DAST-Mediated Deoxyfluorination Approach to 2′,3′-Difluorinated Nucleosides^379^

Finally the Goss group reported a direct DAST-mediated deoxyfluorination with 5-protected uridine (Scheme 101).^379^ The substrate **709** was synthesized via acetonide protection of the ring alcohols, benzylation at OH-5, and acetonide hydrolysis. Reaction of DAST with the diol led to the formation of (protected) 1-(2′,3′-dideoxy-2′,3′-difluoro-β-d-xylofuranosyl) uracil **714** in 11% yield, alongside the corresponding C-2-monofluorinated derivative **716** in 53% yield. This outcome was explained by reaction of the OH-2 group with DAST, leading to **710**. This was intercepted by a uracil carbonyl group leading to the 2,2′-anhydro derivative **711**, similar to what was observed in the Gosselin synthesis (**690** → **691**, Scheme 99). However, because the uracil benzyl protecting group maintained the positive charge, **711** then reacted with fluoride to give the 2′-fluorouridine derivative **712**. This can be compared with uracil protection using a benzoyl group (cf. Scheme 98), which deactivates the heterocycle from 2,2′-anhydro formation. From **712**, a second activation by DAST gave **713**, which could either undergo displacement with fluoride to give **714** or be intercepted again by the uracil carbonyl to give the 2,3′-anhydro compound **715**. This did not undergo S_N_2 reaction with fluoride at C-3 to give (protected) 2′,3′-dideoxy-2′-3′-difluorouridine, but was hydrolyzed during the basic workup to give **716**.

##### 6.4.1.4 Arabino-configured

**Scheme 102:**
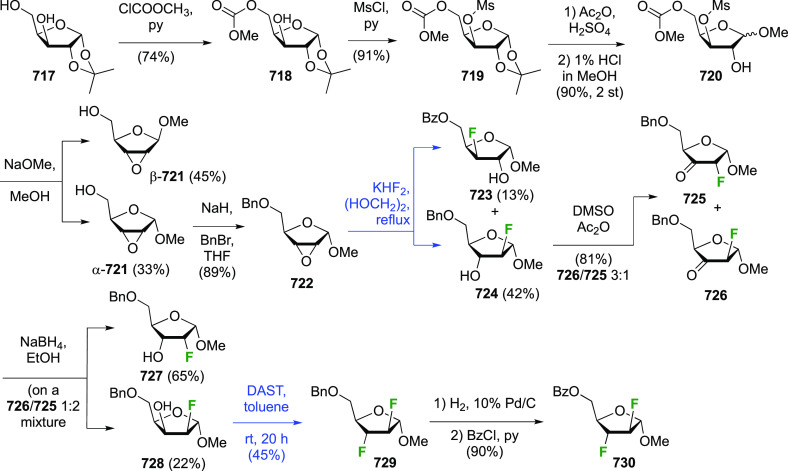
Sequential Fluorination Approach to Give Methyl 5-*O*-benzoyl-2,3-dideoxy-2,3-difluoroarabinofuranoside^380^

The conversion of 5-protected methyl xylofuranoside with DAST to arrive at 2,3-dideoxygenated 2,3-difluorinated araninofuranosides was investigated extensively by Sivets and Mikhailopulo. Their sequential approach is shown in Scheme 102.^380^ Starting from commercially available 1,2-*O*-isopropylidene-α-d-xylofuranose **717**, selective protection as the 5-*O*-methyl carbonate **718** allowed activation of OH-3 as the mesylate **719**. Acetonide acetolysis and methanolysis of the resulting 1,2-di-*O*-acetate, with concomitant anomeric methylation, gave **720**. Treatment with base initiated epoxide formation, at which stage the anomers were separated.^381^ The *ribo*-epoxide α-**721** was benzylated,^382^ and reacted with KHF_2_ in ethylene glycol at reflux temperature. This led to the 2-fluorinated arabinofuranoside **724** in 42% yield, alongside a small amount of 3-fluorinated xylofuranoside **723**.^383^ Hence, in this case the steric hindrance of the benzyloxymethyl substituent overrides the electronic influence of the anomeric center (cf. Schemes 51, 95A). Inversion at C-3 was achieved by oxidation to give **726**, which unfortunately also resulted in epimerization at the 2-position to give inseparable **725** as the thermodynamically most stable isomer. Reduction of the mixture then gave the 2-fluorinated ribofuranoside **727** as the major product which, compared to the initial starting point **724**, actually resulted in inversion at C-2 and not C-3. The 2-fluorinated lyxofuranoside **728** was isolated in only 22% yield (alongside 10% of **724**, not shown).^384^ Deoxyfluorination of **728** gave the 2,3-difluorinated arabinofuranoside **729**, after which a protecting group swap afforded methyl 5-*O*-benzoyl-2,3-dideoxy-2,3-difluoroarabinofuranoside **730**.^380^

**Scheme 103:**
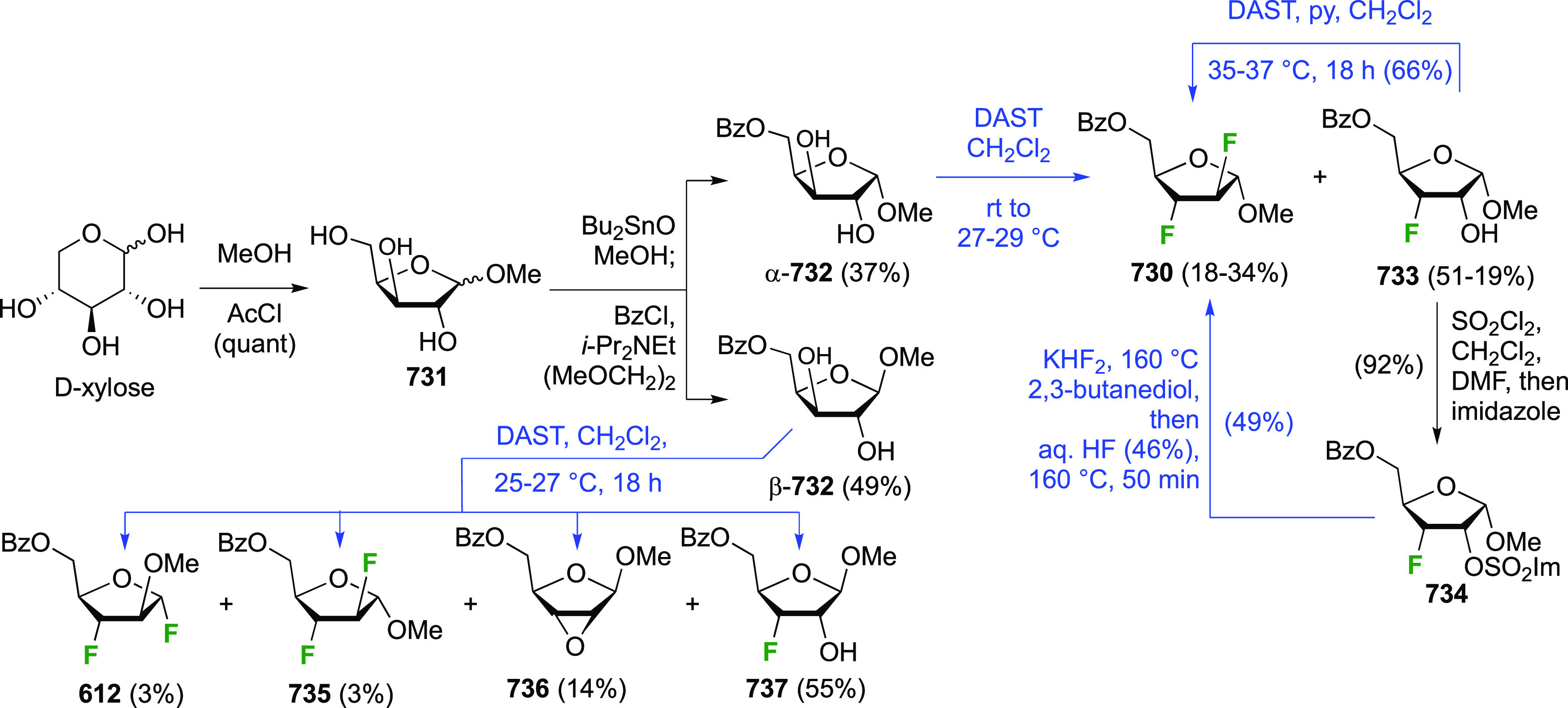
Direct Difluorination Approaches with Methyl 5-*O*-benzoyl Arabinofuranoside^346,385−387^

A direct fluorination approach was also developed, and proved much more efficient (Scheme 103). Methyl d-xylofuranoside **731** was obtained using Anker’s procedure in excellent yield.^354^ Without (possible) anomeric separation at this stage, careful benzoylation at the 5-position followed by chromatographic separation gave the two anomers α- and β-**732**.^346^ When α-**732** was subjected to DAST, the double-inversion product **730** and the OH-3 deoxyfluorination product **733** were obtained, in yields that were dependent on the reaction time. With a 10 h reaction time, 18% of **730** and 51% of **733** was obtained. A longer time (not specified in the scheme) led to **730** in 34% yield, while the yield of **733** decreased to 19%, suggesting **733** is an intermediate in the synthesis of **730**.^346,385−387^ Indeed, isolated **733** was shown to give **730** by DAST treatment in 66% yield.^346^ Alternatively, activation of the OH-2 group in **733** as imidazoylsulfonate **734** allowed fluoride displacement to give **730** in 49% yield.^385^

Treatment of β-**732** with DAST led to the isolation of four compounds, of which 3-deoxy-3-fluoro-β-ribofuranoside **737** was the major, followed by 2,3-anhydro-β-ribofuranoside **736**. Small amounts of the difluorinated arabinofuranoside **735** were isolated, and the formation of this α-anomer was explained by anomerization of an intermediate before deoxyfluorination at C-2. Finally, a rearrangement product **612** was also isolated, which arose through neighboring participation of the anomeric substituent to the activated OH-2 (not shown).^346^

**Scheme 104:**
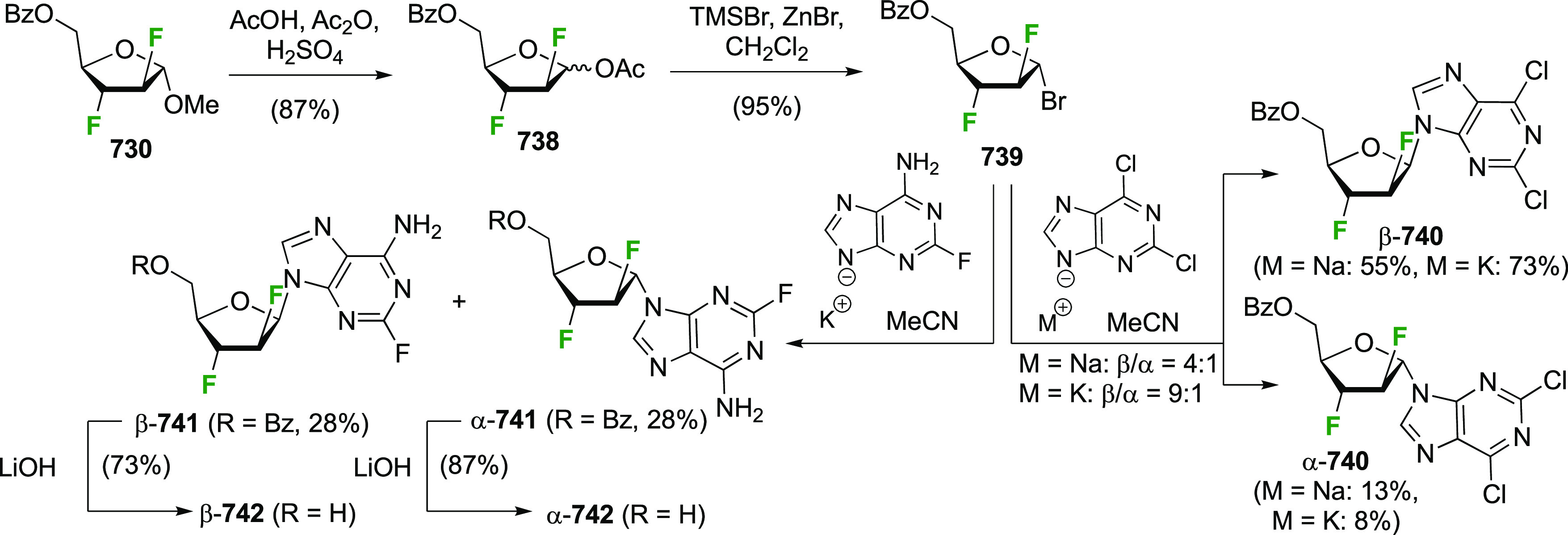
Synthesis of 9-(2′,3′-Dideoxy-2′,3′-difluoro-β-d-arabinofuranosyl) Purines^346,380,385,388^

Methyl 5-*O*-benzoyl-2,3-dideoxy-2,3-difluoroarabinofuranoside **730** has also been used to synthesize nucleosides, although direct condensation of **730** with nucleobases under SnCl_4_ activation was low-yielding.^380^ Hence, **730** was acetolyzed to give **738** (Scheme 104), which was converted to the more reactive glycosyl bromide **739**, which has generally been the substrate of choice for nucleobase introductions.^346,380,385,388^ For example, the reaction of **739** with deprotonated purines gave good yields of the corresponding nucleoside derivatives. Using the sodium salt of 2,6-dichloropurine led to a 2.9–4:1 anomeric ratio of β- to α-**740**,^385,388^ which was improved by using the corresponding potassium salt to 9:1.^388^ In contrast, the reaction of **739** with the potassium salt of 2-fluoroadenine led to a 1:1 anomeric ratio of **741**.^346^ Their deprotection then gave **742**.

**Scheme 105:**
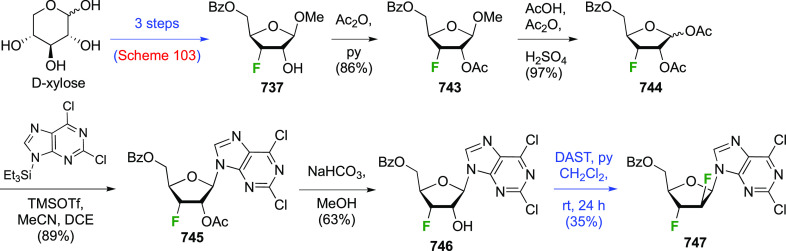
Linear Synthesis of 2′,3′-Difluorinated Arabinofuranosyl Nucleosides Starting from a C-3-Fluorinated Precursor^388^

A number of linear approaches to 2′,3′-difluorinated arabinofuranosyl nucleosides have also been reported. In the Schinazi approach (Scheme 105),^388^ this started from a 3-fluorinated ribofuranosyl precursor **737**, for which the synthesis in three steps from d-xylose was shown in Scheme 103. Acetylation of OH-2 gave **743**, and acetolysis of the anomeric position provided **744**. Coupling with silylated 2,6-dichloropurine 745 in a mixture of acetonitrile and dichloroethane (DCE) under TMSOTf activation led to **746** in excellent yield as the only isolated anomer. The acetate group was now hydrolyzed to give **747**, and deoxyfluorination resulted in the desired nucleoside β-**740** in moderate yield.^388^

**Scheme 106:**
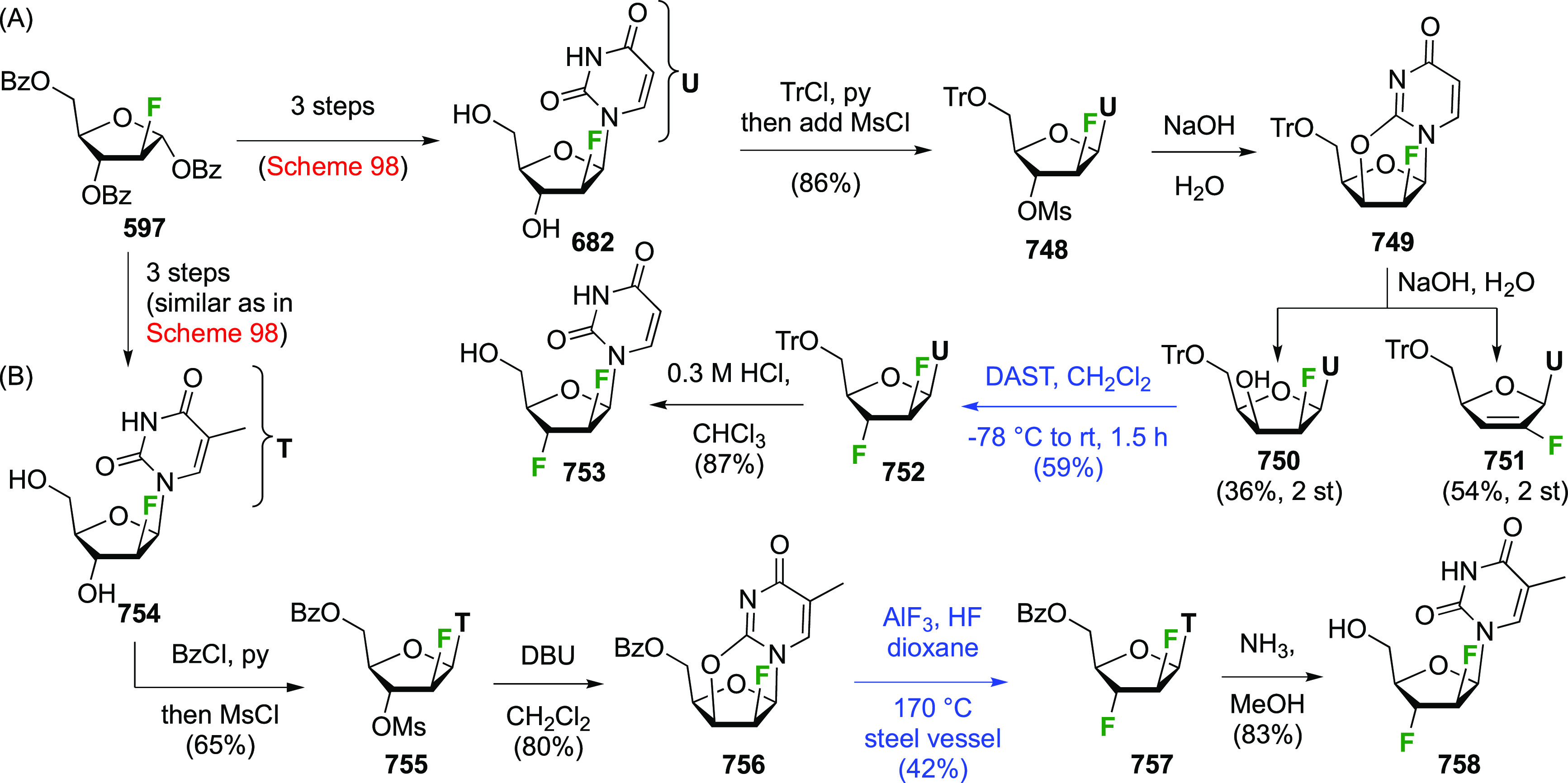
Linear synthesis of 2′,3′-Difluorinated Arabinofuranosyl Nucleosides Starting from a C-2-Fluorinated Precursor^364,375^

Alternatively, the Martin group achieved a linear synthesis using a 2-fluorinated building block (Scheme 106A).^375^ Starting from 1-(2′-fluoro-β-d-arabinofuranosyl)uracil **682**, synthesized as already shown in Scheme 98, selective tritylation at the 5′-position allowed formation of the mesylate **748**. Treatment with base caused cyclization to give the 2,3′-anhydro derivative **749**, which upon further treatment with base hydrolyzed to give the lyxofuranosyluracil derivative **750**, although this process led to a significant amount of elimination to give **751** as the major product. DAST treatment of **750** then introduced the second fluorine, which, in contrast to the second fluorination to arrive at the *lyxo*-configuration (Scheme 98),^373^ proceeded smoothly to give **752**. This was subseqeuently deprotected to give 1-(2′,3′-dideoxy-2′,3′-difluoro-β-d-arabinofuranosyl)uracil **753**.^375^

A shorter process, in which the 2,3′-anhydro opening was directly achieved with fluoride, was developed by the Watanabe group (Scheme 106B).^364^ In a similar way as shown in Scheme 98, 2′-fluoro-5-methyl-β-d-arabinofuranosyluracil (FMAU) **754** was obtained.^389^ Selective benzoylation and mesylation gave **755**, upon which treatment with DBU led to the 2,3′-anhydro **756**. Treatment of **756** with HF under AlF_3_-catalysis^390^ in a steel vessel at 170 °C gave a moderate yield of the desired difluorinated *arabino* nucleoside **757**, which was then deprotected to give 1-(2′,3′-dideoxy-2′,3′-difluoro-β-d-arabinofuranosyl)uracil **758**.^364^

#### 6.4.2 Tetrafluorinated at Positions 2 and 3

**Scheme 107:**
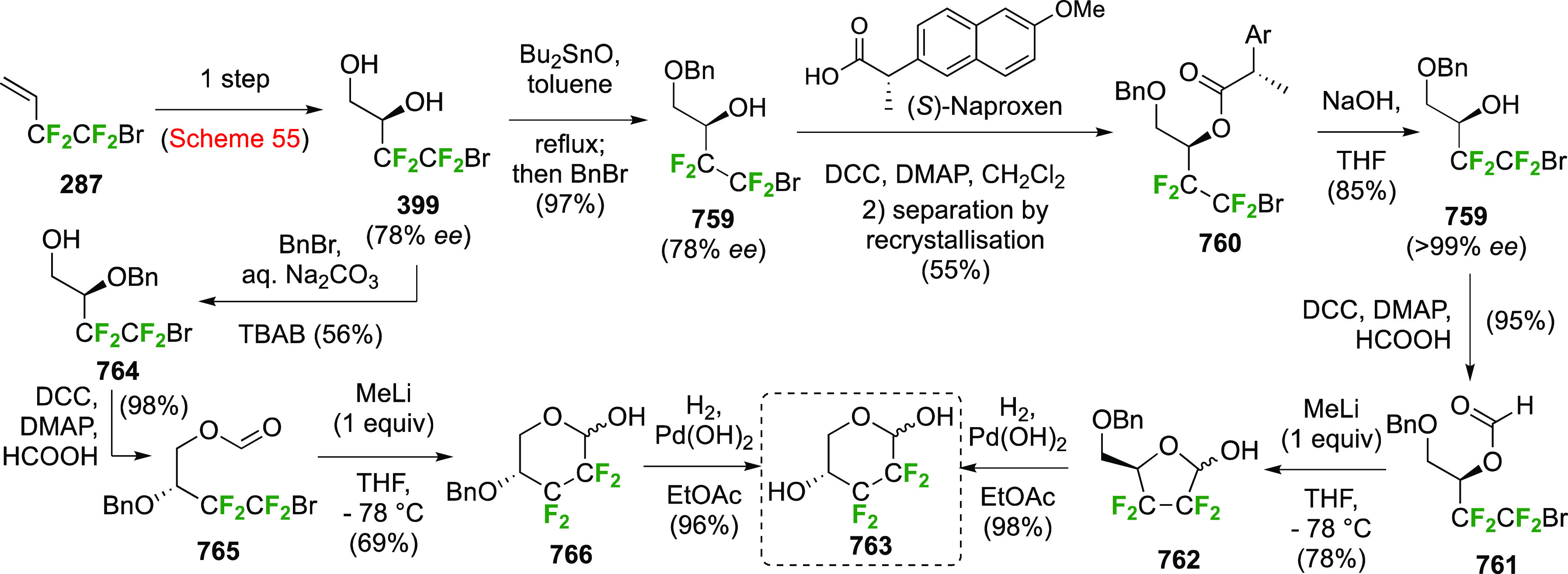
Synthesis of 2,3-Dideoxy-2,2,3,3-tetrafluorinated Pentofuranose and -pyranose^60,290^

The Linclau group developed an enantioselective *de novo* synthesis to 2,3-dideoxy-2,2,3,3-tetrafluorinated pentoses (Scheme 107).^60,290^ As shown earlier in Scheme 55, the diol **399** was obtained in 79% ee by an asymmetric dihydroxylation reaction, and could be crystallized to enantiopurity after naphthyl methyl protection and derivatization with (*S*)-Naproxen. In this case, the tetrafluorinated pentose synthesis was carried out with a benzyl protecting group.^60,288^ Hence, after Sharpless asymmetric dihydroxylation to **399**,^290^ benzylation of the primary alcohol, functionalization with (*S*)-Naproxen to effect separation of the thus formed diastereomers, and ester cleavage gave **759** in >99% enantiopurity.^288,290^ As before, the expensive (*S*)-Naproxen could be recovered and recycled. Formylation of **759** to give **761** then allowed cyclization via lithiation, giving **762** in 78% yield. Removal of the benzyl group resulted in ring tautomerization to give 2,3-dideoxy-2,2,3,3-tetrafluoro-d-*glycero*-pentopyranose **763**.^60^

Alternatively, the anionic cyclization to give a protected pentopyranose was also demonstrated. Similar to what was shown for the alkylation of **267** to give **268** in Scheme 36, deprotonation of **399** followed by alkylation led to the protection of the alcohol group adjacent to the fluorination giving **764** in reasonable yield. Nevertheless, < 2% of the dibenzylated or the regioisomer was isolated (not shown). Formylation of **764** gave cyclization precursor **765**, whose treatment with MeLi resulted in **766**. Hydrogenolysis of the benzyl group then also gave **763**.^290^

**Scheme 108:**
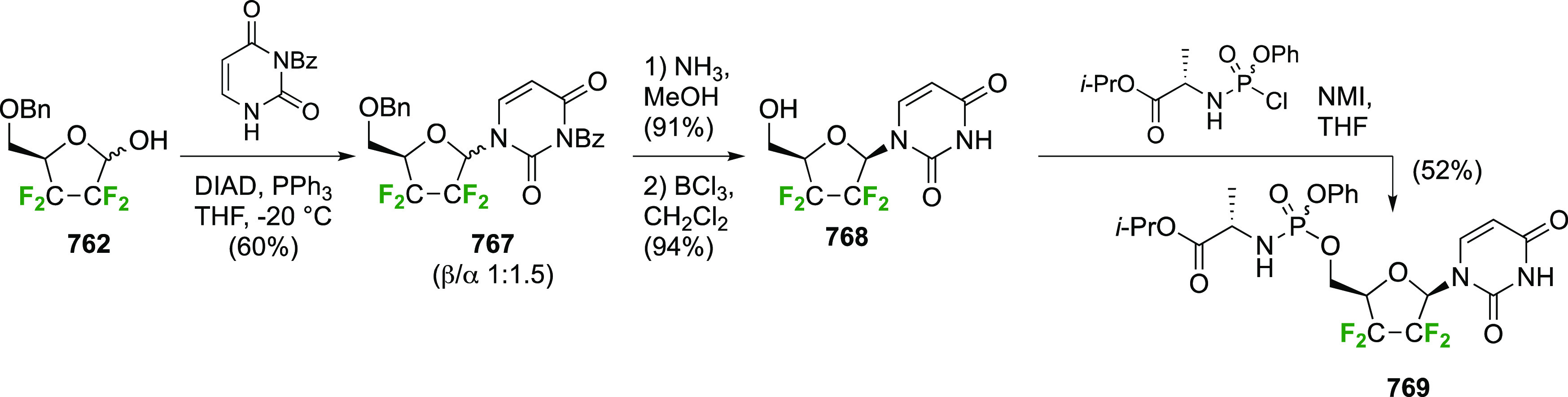
Synthesis of 2′,3′-Dideoxy-2′,2′,3′,3′-tetrafluorinated Uridine and Its Prodrug^391^

Starting from **762** (Scheme 108), the Schinazi group synthesized a number of nucleosides along with their prodrugs, illustrated here for uridine.^391^ While Vorbrüggen-type nucleobase introduction via the corresponding triflate of **762** proved unsuccessful, direct introduction under Mitsunobu conditions afforded the desired coupling products in moderate anomeric ratios. Anomeric separation and deprotections gave uridine analogue **768**, which was then converted to its prodrug derivative **769**. This worked for all five typical nucleoside derivatives (U, T, A, G, C). Conformational analysis indicated that the tetrafluorinated uracil preferred the 3′-*endo* conformation, unlike its 2′-3′-dideoxy analogue, which showed no preference between 2′-*endo* and 3′-*endo*. Unfortunately, none of the tetrafluorinated nucleosides or their prodrugs showed significant activity against a number of viruses.

### 6.5 Fluorination at Positions 2 and 4

**Scheme 109:**
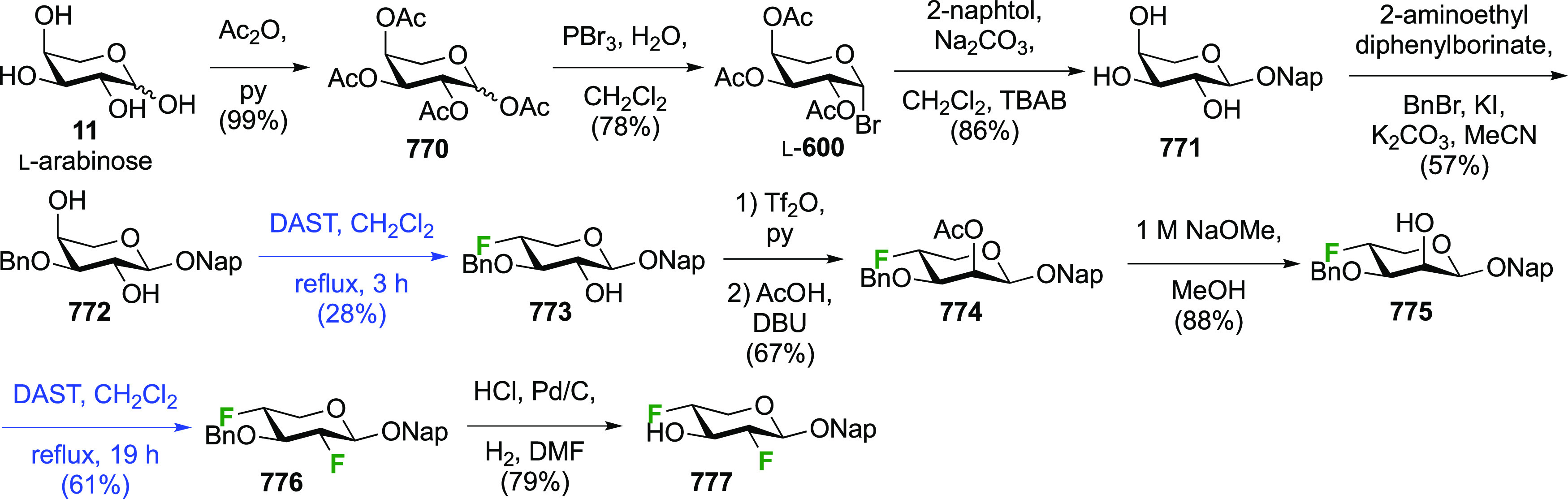
Synthesis of a 2,4-Difluorinated Xylose Derivative^393^

The synthesis of a 2,4-dideoxy-2,4-difluorinated d-xylopyranoside has been described by the Ellervik group (Scheme 109).^392^ Peracetylation of l-arabinose to its pyranoside **770** was followed by anomeric bromination to give l-**600**.^393^ Glycosidation with 2-naphthol to **771** was followed by selective benzylation at the 3-position using a borinic acid catalyst. When Ag_2_O was added, a 1:1 mixture of 3- and 4-benzylated sugars was obtained (not shown), but a metal-free version gave solely the desired 3-*O*-benzylated **772**. Reaction of **772** with DAST was selective for the 4-position, albeit in modest yield. The deoxyfluorination of the corresponding benzoate proceeded in a lower 22% yield (not shown). Inversion at C-2 proceeded best via triflation followed by nucleophilic substitution with acetate, and gave **775** after removal of the acetate group. An oxidation/reduction attempt led to fluoride elimination (not shown). The second deoxyfluorination now proceeded in good yield to give **776**, whereupon deprotection finally gave 2-naphthyl 2,4-dideoxy-2,4-difluoro-β-d-xylopyranoside **777**.^393^

**Scheme 110:**
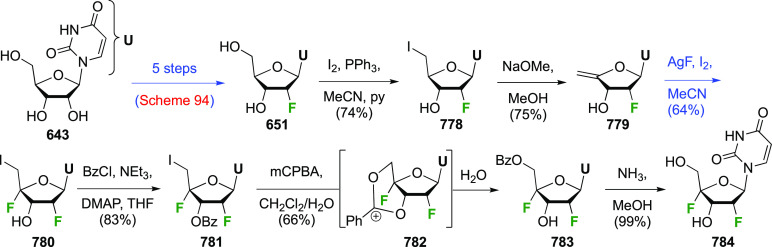
Synthesis of a 2,4-Difluorinated Uridine Derivative^394^

A number of 2′,4′-difluorinated nucleosides have also been reported. The Damha group designed 2′-deoxy-2′,4′-difluorouridine **784** as a monocyclic conformationally locked nucleoside (Scheme 110).^394^ The synthesis started from 2′-deoxy-2′-fluorouridine **651**, for which the synthesis was described in Scheme 94. Selective iodination at the primary position gave **778**, which was treated with base to effect elimination to **779**. Iodofluorination was achieved by gradual addition of I_2_/AgF in acetonitrile at 0 °C to give **780**. Displacement of the iodide with benzoate proved difficult,^395^ due to the deactivating effect of fluorine toward S_N_2 reactions.^164−166^ As a solution, the 3-position was benzoylated and the iodide oxidized to hypoiodate, which initiated intramolecular displacement from the 3-*O*-benzoate group to give intermediate **782**. The addition of water led to migration of the benzoate to the 5′-position, leading to **783** in 66% yield. Aminolysis of the benzoate group then gave **784**.^394^

**Scheme 111:**
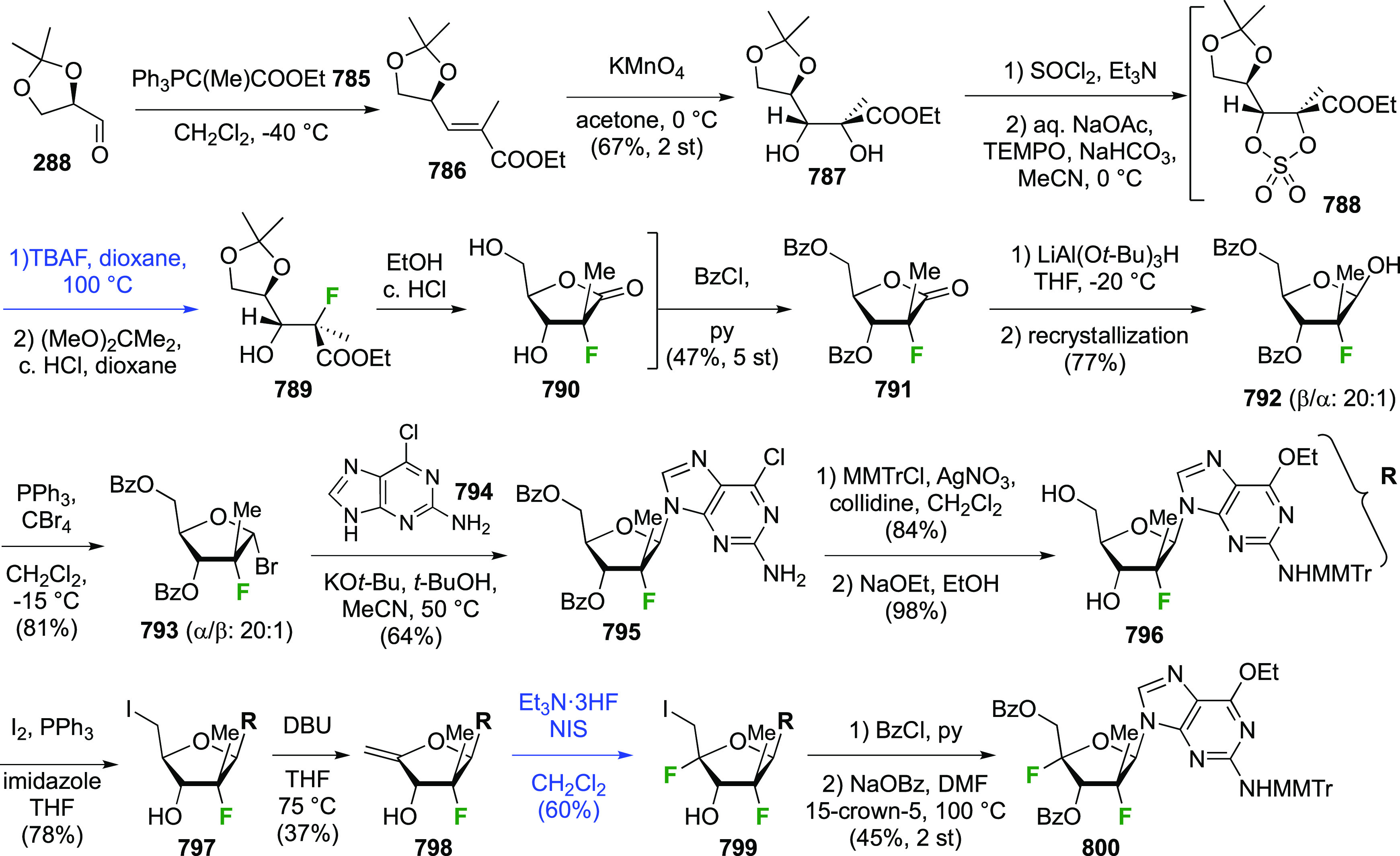
Synthesis of a 2,4-Difluorinated Nucleoside Precursor in the Synthesis of a HCV NS5B Polymerase Inhibitor^371^

The Dyatkina group reported a 4′-fluorinated analogue of a 2′-fluorinated-2′-methylated nucleoside, active against HCV (Scheme 111).^371^ The synthesis, which had been optimized on a large-scale, started with commercially available d-glyceraldehyde **288**, also easily available from d-mannitol.^396,397^ Reaction of a slight excess of **288** with the commercially available ylide **785** gave the alkene **786** in a 97:3 *E*/*Z* ratio,^398^ which was taken to the next step. Dihydroxylation was successful under OsO_4_ conditions but, aiming to avoid the use of this toxic reagent on large scale, KMnO_4_-mediated dihydroxylation in acetone was conducted instead. After crystallization of the crude reaction mixture, this gave diol **787** as a single diastereomer in 53% yield from **785**. Fluorination was achieved after activation of the diol as cyclic sulfate ester **788**, with hydrolysis of the residual sulfate requiring modified conditions (concentrated HCl in 2,2-dimethoxypropane) to prevent acetonide hydrolysis, which allowed purification of **789** via an aqueous workup. The synthesis was telescoped further by treating **789** with concn. HCl in EtOH to effect acetonide hydrolysis and lactone formation, and the resulting crude **790** was then protected as 3,5-di-*O*-benzoate **791** in 47% overall yield from **787**.^398^ The lactone was then reduced to lactol **792**,^399^ which was isolated after crystallization as the β-anomer. This proved important toward stereoselective nucleobase introduction. Bromination under Appel conditions proceeded with clean inversion of configuration, to give the α-anomeric bromide **793**. Nucleoside formation was then achieved with the potassium salt of purine **794** in a 64% yield and 14:1 β/α ratio.^399^ From **795**, fluorination at the 4′-position was achieved using similar methodology as shown in Scheme 110. After functionalization and protection of the nucleobase, leading to **796**, the OH-5 was subjected to deoxyiodination to give **797**. Elimination to **798** was followed by iodofluorination, which provided **799** as a single isomer. Following benzoylation at the 3′-position, nucleophilic substitution of the 5-iodo group with benzoate led to **800**, which was further converted to a nucleoside prodrug (not shown).^371^

### 6.6 Fluorination at Positions 2 and 5

**Scheme 112:**
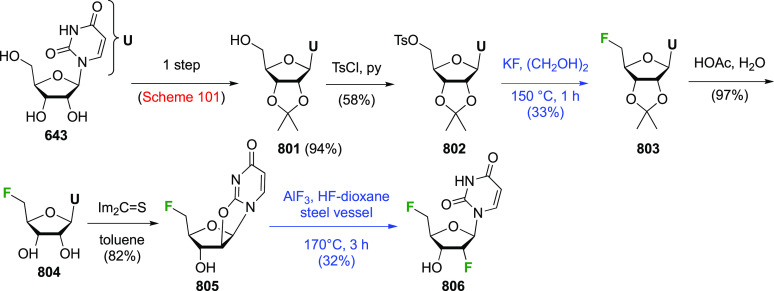
Synthesis of 2,5-dideoxy-2,5-difluorouridine^400^

Von Schütt et al. reported the synthesis of 2,5-dideoxy-2,5-difluorouridine **806** (Scheme 112).^400^ Starting from uridine acetonide **801** (cf. Scheme 101), tosylation at the 5-position allowed its substitution by fluoride to give **803**. Acetonide removal was followed by 2,2′-anhydro formation (**805**). Subsequent fluorination with AlF_3_/HF afforded the final compound **806** in modest yield.

### 6.7 Fluorination at Positions 3 and 5

**Scheme 113:**
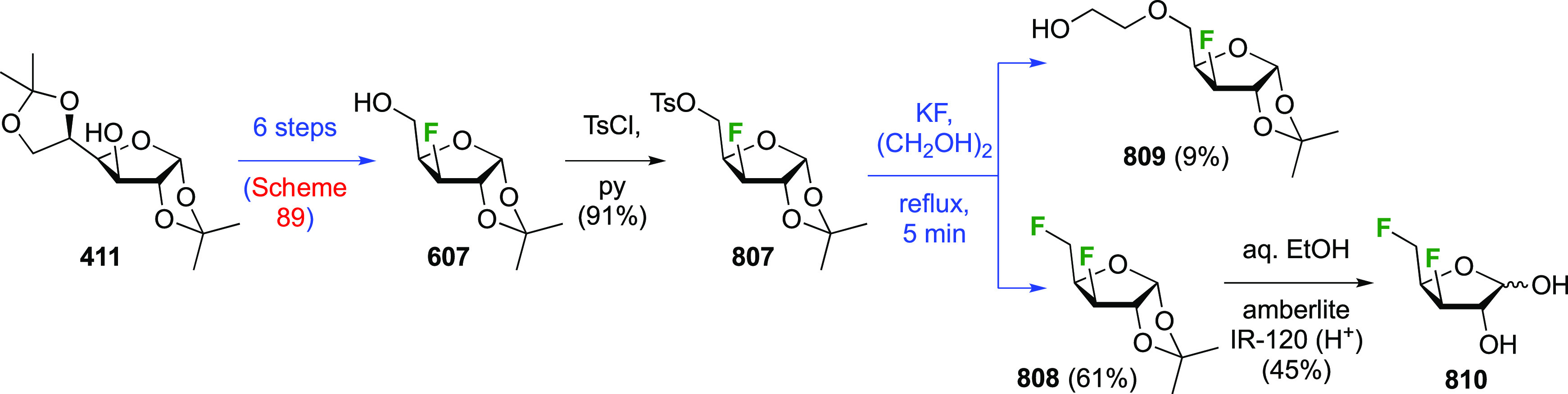
Synthesis of 3,5-Dideoxy-3,5-difluoro-d-xylofuranose^56^

Foster et al. reported the synthesis of 3,5-dideoxy-3,5-difluoro-d-xylose, which was the first reported dideoxy-difluorinated sugar derivative in the literature (Scheme 113).^56^ 3-Deoxy-3-fluoro-1,2-*O*-isopropylidene-α-d-xylofuranose **607**, synthesized in six steps from glucose diacetonide **411** (as shown in Scheme 89), was tosylated to give **807**.^401^ Nucleophilic displacement with fluoride gave the 3,5-dideoxyfluorinated xylofuranose derivative **808**,^56^ whereupon mild acid hydrolysis conditions gave 3,5-dideoxy-3,5-difluoro-d-xylofuranose **810**. The fluoride substitution was accompanied by nucleophilic substitution with the solvent leading to **809**, and by hydrolysis leading back to **607** (5%, not shown).

## 7 Ketosugars

### 7.1 Erythro-2-pentulose (Ribulose)

**Scheme 114:**
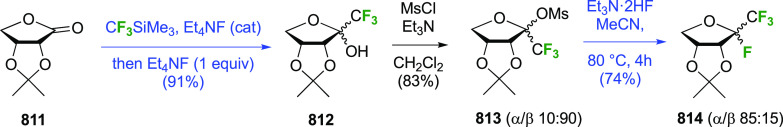
Synthesis of a 1,1,1,2-Tetrafluorinated Ribulose Derivative^402^

The Anker group reported the synthesis of the 1-deoxy-1,1,1-trifluororibulosyl fluoride derivative **814** (Scheme 114).^402^ Trifluoromethylation of the acetonide-protected erythronolactone **811** led to **812** as a mixture of equilibrating anomers.^403^ Mesylation afforded **813** with β-selectivity. This was stable to chromatography thanks to the inductive effect of the trifluoromethyl group. Displacement with Et_3_N·2HF then gave the corresponding ribulosyl fluoride **814**, largely with inversion of configuration.^402^

### 7.2 Fructose

**Scheme 115:**
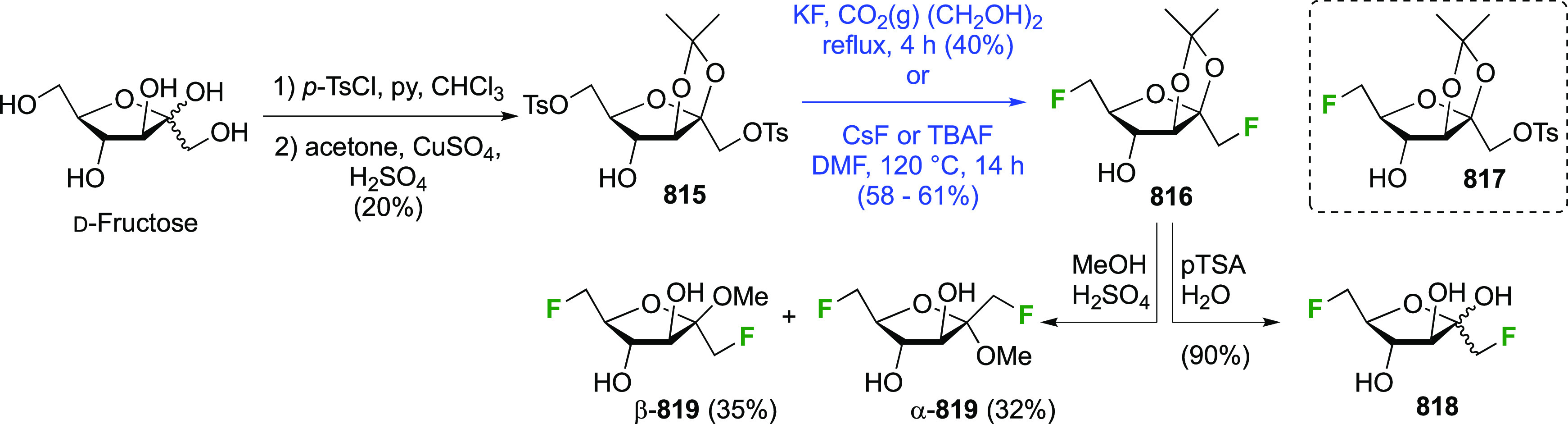
Synthesis of 1,6-Dideoxy-1,6-difluorinated Fructose Derivatives^405,406^

The synthesis of 1,6-dideoxy-1,6-difluorinated fructose derivatives is shown in Scheme 115. d-Fructose was tosylated at the primary positions, and converted to its acetonide **815**,^404^ which was subjected to tosylate displacement. Guthrie et al. reported that reaction with KF in ethylene glycol at 150 °C gave **816** in 20% yield, alongside the monofluorinated **817** in 29% yield.^405^ Pacak et al. achieved a 40% yield of **816** in refluxing ethylene glycol while bubbling through CO_2_ gas.^406^ With DMF as the solvent, reaction with LiF or CsF at 100 °C did not lead to any fluorination, while the use of TBAF at 80 °C gave 45% of **817** and 8% of **816** (not shown).^405^ However, increasing the temperature to 120 °C led to the formation of **816** in 58–61% yield with CsF or TBAF.^405^ The deoxyfluorination of the fructose acetonide 1-position is known to be difficult; it was reported to be unsuccessful with DAST and is best achieved via the corresponding triflate.^407^ Aqueous acid-catalyzed hydrolysis of **816** afforded 1,6-dideoxy-1,6-difluoro-d-fructose **818**,^406^ while methanolysis led to the two methyl fructoside anomers of **819**.^405^

**Scheme 116:**
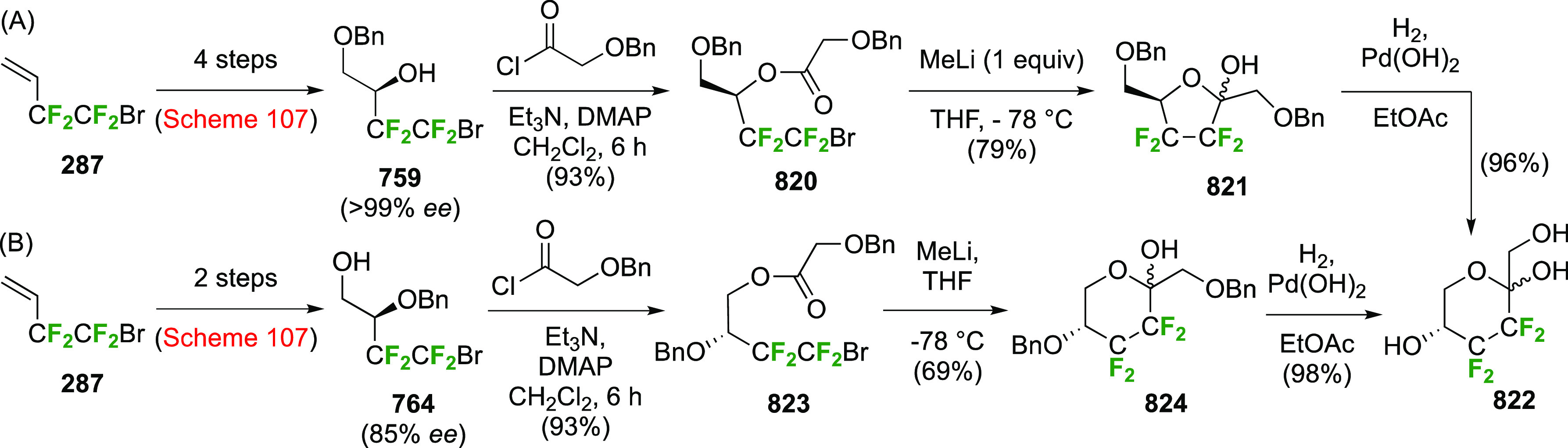
Synthesis of a Tetrafluorinated Fructose Derivative^290^

The synthesis of a 2,2,3,3-tetrafluorinated fructose analogue was reported by Linclau et al. using a fluorinated building block approach (Scheme 116A).^290^ Starting from **759**, for which the synthesis was shown in Scheme 107, ester formation with benzyloxyethanoyl chloride led to **820**, and anionic cyclization afforded the ketofuranose **821**. Removal of the protecting groups was accompanied by ring tautomerization to give the ketopyranose **822**. This fructose derivative was also obtained via the other possible anionic cyclization pathway from **764** (Scheme 116B), the regioisomer of **759**, the synthesis of which was also depicted in Scheme 107. Ester formation to obtain **823** allowed cyclization to **824**, which upon hydrogenolysis then gave **822**.^290^

**Scheme 117:**
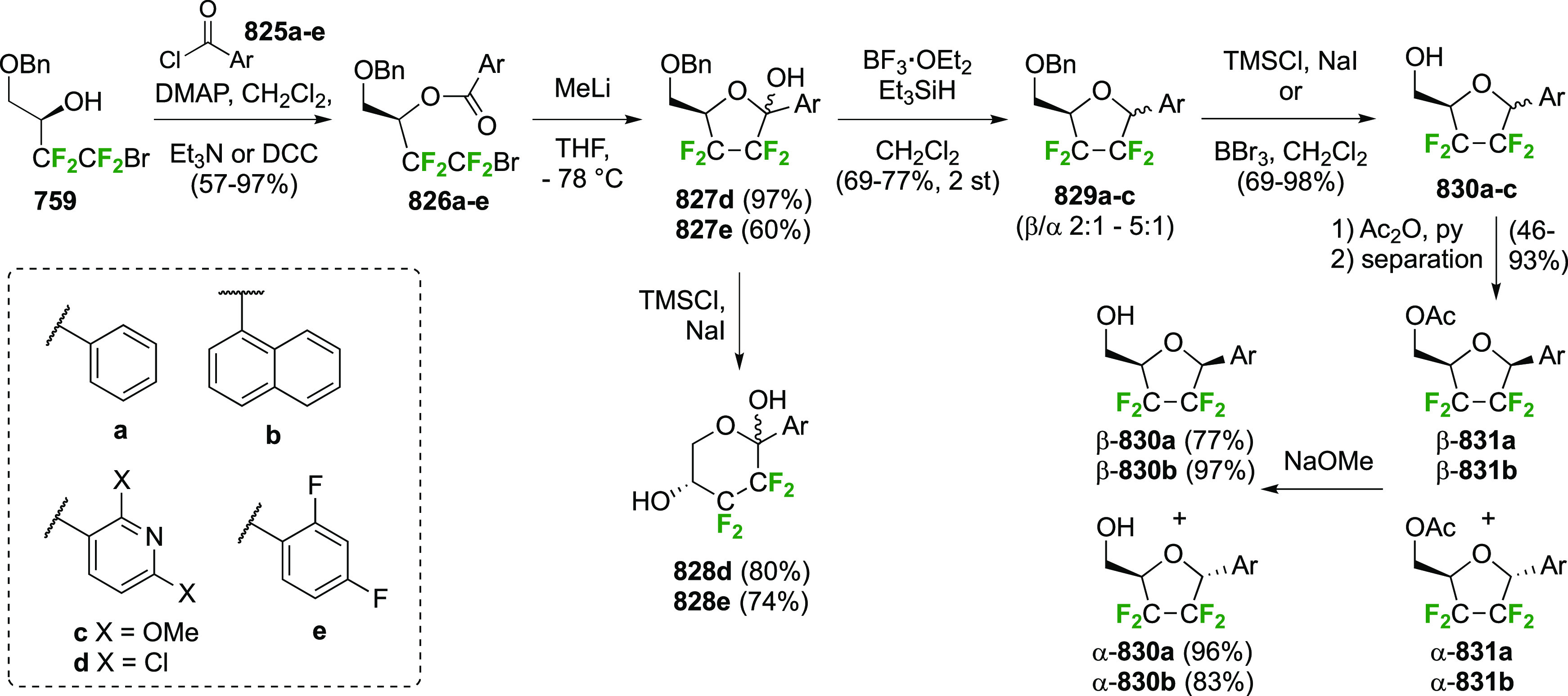
Synthesis of 3,3,4,4-Tetrafluoroaryl-C-nucleoside Analogues^408^

This methodology was used by Gouverneur et al. for the synthesis of pentaketose derivatives (Scheme 117).^408^ Here, **759** was esterified with aromatic acid chlorides **825a**–**e** to give **826a**–**e** as substrates for the anionic cyclization. MeLi-treatment of **826d**,**e**, followed by removal of the benzyl group led to the formation of ketopyranoses **828d**,**e**. MeLi-treatment of **826a**–**c**, followed by reduction of the resulting hemiacetal led to **829a**–**c**, upon which debenzylation gave the to C-nucleosides **830a**–**c**. The anomers of **830a**,**b** could be separated after acetylation to **831a**,**b**.

### 7.3 d-*Gluco*-hept-2-ulose

**Scheme 118:**
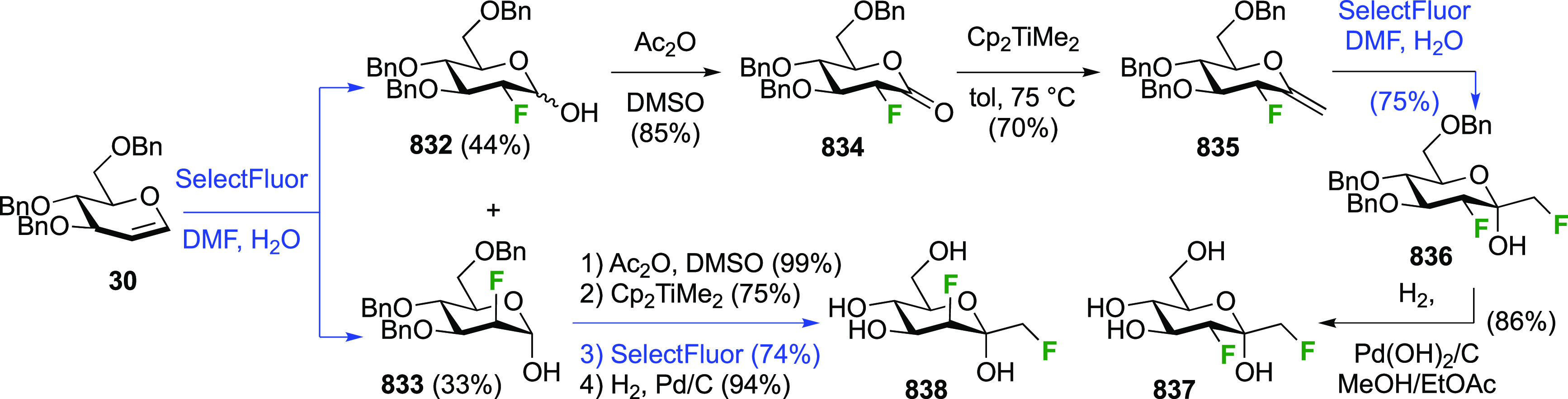
Synthesis of 1,3-Dideoxy-1,3-difluorinated hept-2-uloses^409,410^

The Thiem group reported the synthesis of a number of difluorinated hept-2-ulose derivatives.^409,410^ Starting from tri-*O*-benzyl-d-glucal **30** (Scheme 118), reaction with SelectFluor gave the separable 2-deoxy-2-fluoroglucose **832** and -mannose **833**. From **832**, oxidation to the lactone **834** was followed by a Petasis olefination to give the exocyclic enol ether **835**. Another reaction with SelectFluor then provided the 1,3-difluorinated heptulose **836** as the α-anomer, which was finally deprotected to give 1,3-dideoxy-1,3-difluoro-α-d-*gluco*-hept-2-ulopyranose **837**.^409^ A similar reaction sequence starting from **833** gave the corresponding epimer **838**.^410^

**Scheme 119:**
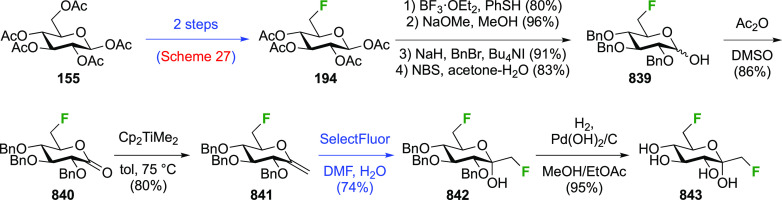
Synthesis of 1,7-Dideoxy-1,7-difluoro-α-d-*gluco*-hept-2-ulopyranose^409^

The same group also reported the synthesis of the 1,7-difluorinated hept-2-ulose **843** (Scheme 119),^409^ starting from 1,2,3,4-tetra-*O*-acetyl-6-deoxy-6-fluoro-β-d-glucopyranose **194**, for which the synthesis was shown in Scheme 27. From **195**, anomeric protection as the thioglycoside, protecting group switch to benzyl, and anomeric deprotection gave **839**. Lactol oxidation, Petasis olefination, and SelectFluor treatment then led to the formation of **842**, which was deprotected to give 1,7-dideoxy-1,7-difluoro-α-d-*gluco*-hept-2-ulopyranose **843**.

### 7.4 2-Keto-3-deoxy-d-*glycero*-d-*galacto*-nononic Acid (Kdn)

**Scheme 120:**
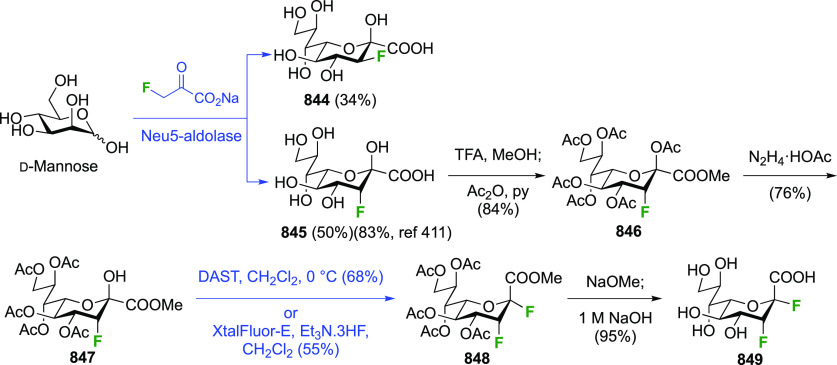
Synthesis of 3-Deoxy-3-fluoro-d-*erythro*-β-l-*manno*-non-2-ulopyranosyl Fluoride^411,412^

The synthesis of a 2,3-difluorinated Kdn derivative was reported by both the Withers and Bennet groups.^411,412^ The enzyme-catalyzed aldolase reaction between d-mannose and 3-fluoropyruvic acid sodium salt (Scheme 120) was reported by the Chen group to give both F-3 diastereomers in 84% combined yield,^413^ with the F_ax_-3 diastereomer as the major product. This aldolase reaction is a key strategy for the synthesis of 3-fluorinated sialic acids starting from d-ManNAc (see section 9). d-ManNAc is the natural substrate of the enzyme, and the reaction with d-mannose was reported to be slower; hence, an extended reaction time was needed.^412^ In contrast, the aldolase enzyme used by the Bennet group gave **845** as the only reported diastereomer in 83% yield. Protection of the carboxylic acid and alcohol groups followed by anomeric deprotection gave ketose **847**, ready for anomeric fluorination. This was achieved with DAST^412^ or XtalFluor-E,^411^ and in both cases only the formation of the desired β-anomer **848** (cf. section 9.1.1) was reported. Deprotections afforded 3-deoxy-3-fluoro-d-*erythro*-β-l-*manno*-non-2-ulopyranosyl fluoride **849**.^411^

**Scheme 121:**
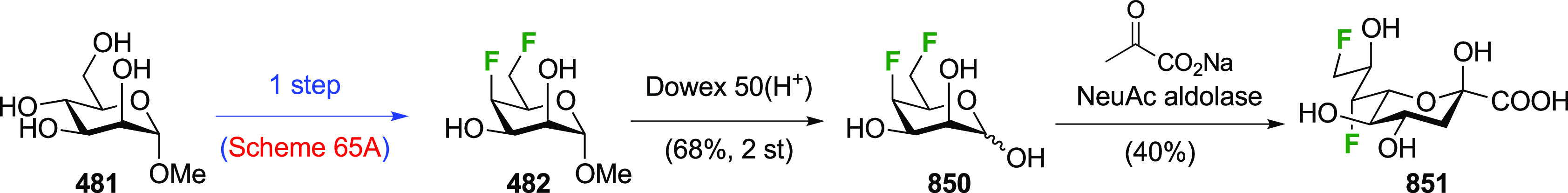
Synthesis of 7,9-Dideoxy-7,9-difluoro Kdn^333^

Finally, Neu5Ac aldolase-catalyzed aldol reaction between 4,6-dideoxy-4,6-difluoro talose **850** (Scheme 121), and pyruvic acid was reported to give 7,9-dideoxy-7,9-difluoro Kdn **851**.^333^ Only one diastereomer was reported. The talose derivative **850** was obtained from hydrolysis of **482**, the one-step synthesis of which was described in Scheme 65A.

## 8 Aminosugars

In this section, the synthesis of polyfluorinated aminosugars and their protected derivatives, including azido- or other aminofunctionalized sugars, is given.

### 8.1 Fluorination at Two Positions

#### 8.1.1 Fluorination at Positions 1 and 2

##### 8.1.1.1 Galactose Stereochemistry

**Scheme 122:**
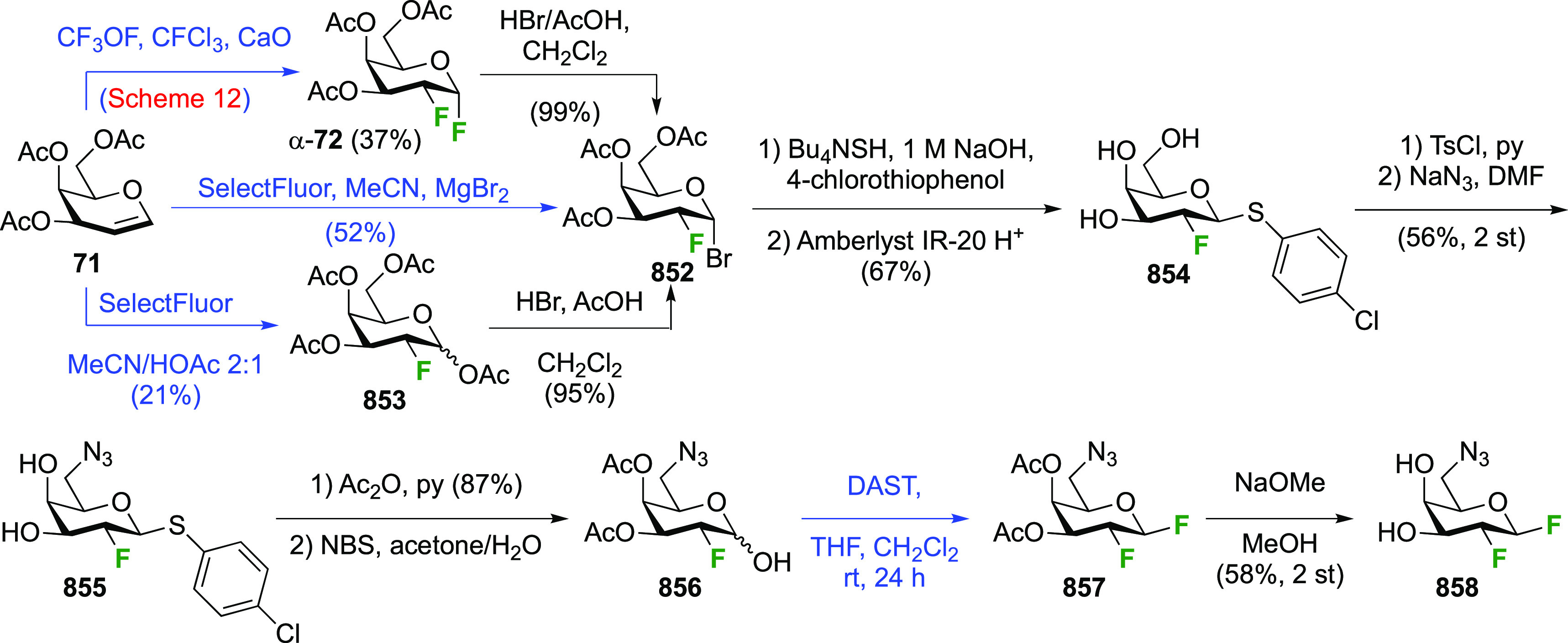
A Sequential Fluorination Approach to a 2,6-Dideoxy-6-azido-2-fluoro Galactosyl Fluoride^414^

Vocadlo and Bertozzi published the synthesis of 6-azido-2,6-deoxy-2-fluoro-β-d-galactosyl fluoride **858** (Scheme 122) as a probe for activity-based labeling of retaining glycosidases.^414^ Their synthesis involved the galactosyl bromide **852**, which was obtained from tri-*O*-acetyl-d-galactal **71**. This was a two-step procedure: first via reaction with CF_3_OF (as shown in Scheme 12) to give α-**72**, which was then converted to the glycosyl bromide **852** with HBr in acetic acid.^415^ Alternatively, the SelectFluor procedure can be used with acetic acid as the solvent to obtain **853** (cf. Scheme 9B), which can also be converted to **852** using HBr in acetic acid.^416,417^ Finally, **71** can be directly converted to **852** using Dax’ original procedure with SelectFluor and a bromide source.^194^ The anomeric position of **852** was protected as the thioglycoside,^414^ and subsequent acetate hydrolysis gave **854**. Selective tosylation at the primary position and displacement with azide gave **855**, upon which the remaining alcohols were reprotected as acetates and the anomeric position was deprotected. This led to **856**, the treatment of which with DAST gave **857** as the only reported anomer (cf. α/β ratio of 14:86 for tri-*O*-benzyl-2-deoxy-2-fluorogalactose **79**, Scheme 12), which was immediately deprotected to give **858**.

**Scheme 123:**
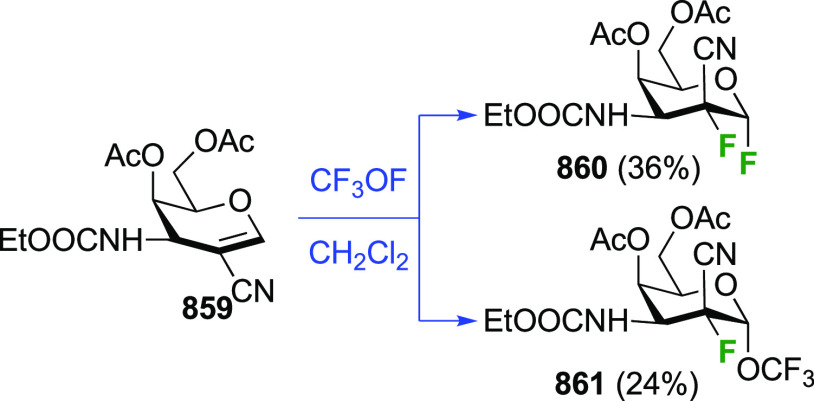
Direct Difluorination of a 3-Amino-2-cyano Functionalized Glycal^419^

The Jordaan group reported on the reaction of the 3-deoxy-3-aminoglucal derivative **859**(418) with CF_3_OF (Scheme 123), which led to the 2-cyano-2-deoxy-2-fluorogalactosyl fluoride derivative **860** alongside the corresponding trifluoromethyl galactoside **861**.^419^ Hence, the same facial selectivity compared to tri-*O*-acetyl galactal was observed, as shown in Scheme 12, although in that case products arising from reaction at the β-face were isolated.

##### 8.1.1.2 Fucose Stereochemistry

**Scheme 124:**
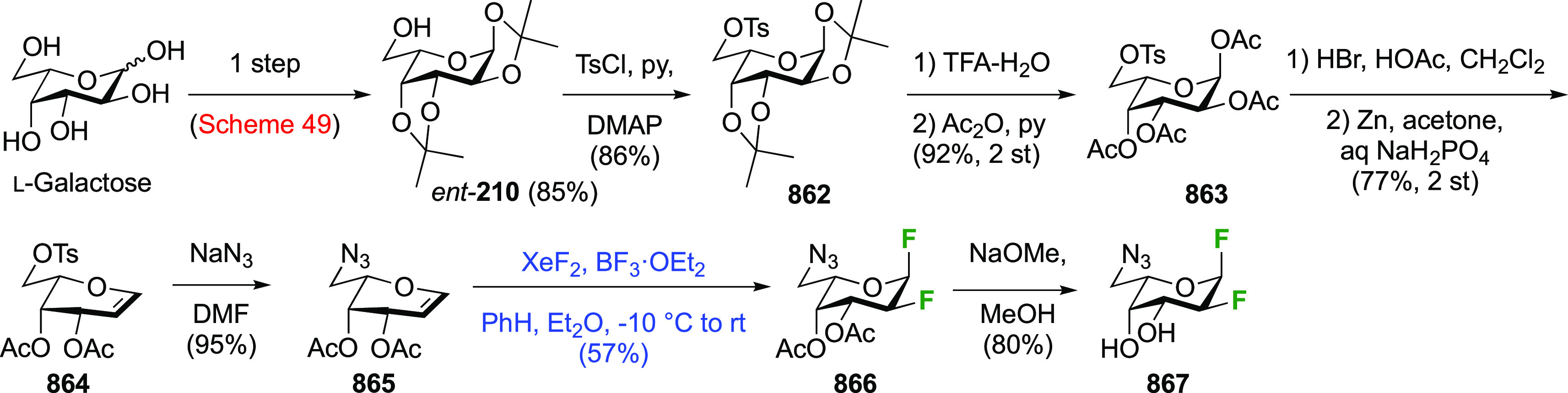
Direct Difluorination Approach to a 6-Azido-1,2-difluorinated Fucose Derivative^420^

The Wennekes group reported the synthesis of the analogous 6-azidofucose activity probe **867** (Scheme 124).^420^ Protection of l-galactose as shown in Scheme 49 led to *ent*-**210**, which was tosylated and converted to the peracetate **863**. Installation of the Δ1,2 double bond afforded the 6-tosyloxyfucal **864**, for which substitution with azide gave **865**. Interestingly, it was reported that introducing the azido group before fucal synthesis caused its substitution by bromide in the anomeric bromination step, a problem not seen with the tosylate. Direct vicinal difluoride introduction with XeF_2_ gave **866** as the only reported product. The excellent stereoselectivity is consistent with the analogous reaction on the corresponding tri-*O*-acetyl galactal **1** or di-*O*-acetal fucal **80** (Schemes 12 and 13), although in these cases other diastereomers were reported in minor amounts. Deprotection of **866** then gave 6-azido-2-deoxy-2-fluoro-α-l-fucosyl fluoride **867**.

**Scheme 125:**
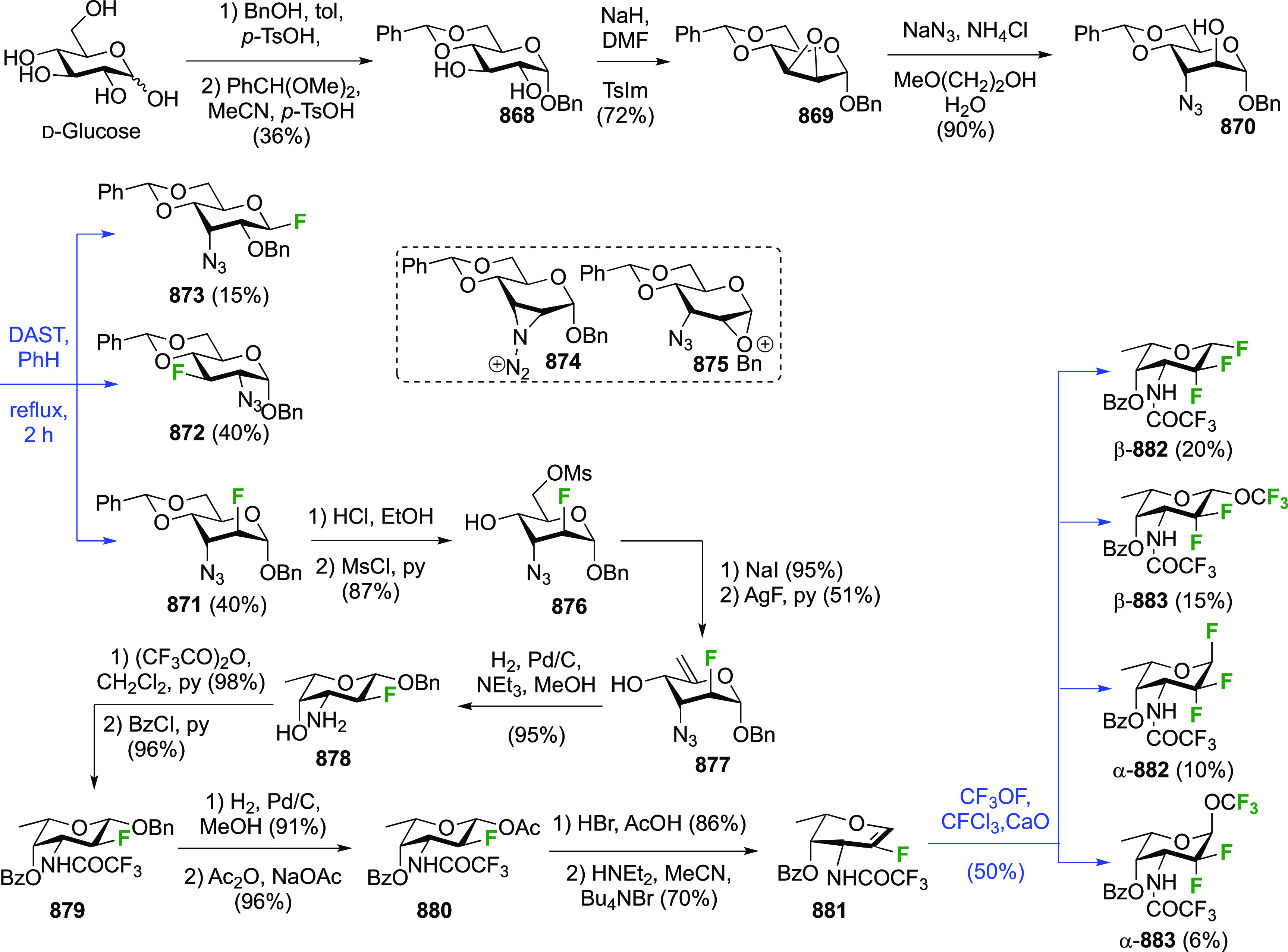
Direct Difluorination of a 2-Fluorinated Glycal to Arrive at 1,2,2-Trifluorinated Sugars^421^

The 1,2,2-trifluorinated fucosamine derivatives α- and β-**882** (Scheme 125) were synthesized by the Lukacs group as intermediates in their 2,2-difluorodaunosamine synthesis.^421^ Starting from d-glucose, anomeric protection as α-benzyl glucoside, followed by standard benzylidene protection gave **868**. In the original report, **868** was converted to **869** via the corresponding dimesylate, which gave a very low yield (2%).^422^ However, a procedure reported by the Magnusson group using tosyl imidazole, originally introduced by Fraser-Reid for this purpose,^423^ gives access to **869** in a good yield.^424^ Its azidolysis furnished azido alcohol **870**,^425^ which was then treated with DAST in boiling benzene to give the desired **871** in 40% yield. Unsurprisingly, the reaction outcome was determined by the two adjacent axial substituents, ideally positioned for neighboring group participation, leading to intermediates **874** and **875**. This not only resulted in the desired fluorination with retention of configuration (**871**, 40%), but also in the formation of the two possible rearrangement products **872** (40%), and **873** (15%). While fluoride attack at C-2 is stereoelectronically favored (chairlike transition state), it is perhaps unexpected to observe that the azido migration product **872** is formed in preference over the benzyl ether migration product **873**. Acid-catalyzed deprotection of **871** and selective mesylation at the primary position afforded **876**. Substitution with iodide was followed by AgF treatment to effect elimination to unsaturated compound **877**. Its rapid hydrogenation led to **878** with l-*fuco*-stereochemistry, with simultaneous reduction of the azide but without cleavage of the benzyl groups. Amine protection as the trifluoroacetamide and benzylation of the alcohol gave **879**, from which the anomeric benzyl was hydrogenolyzed and converted to acetate **880**.^425^ 2-Fluorofucal synthesis by anomeric bromination and elimination was achieved, and reaction with CF_3_OF under Lewis acid catalysis furnished the typical mixture of anomeric fluorides and trifluoromethyl glycosides, **882** and **883**.^421^

##### 8.1.1.3 Glucose/Mannose Stereochemistry

**Scheme 126:**
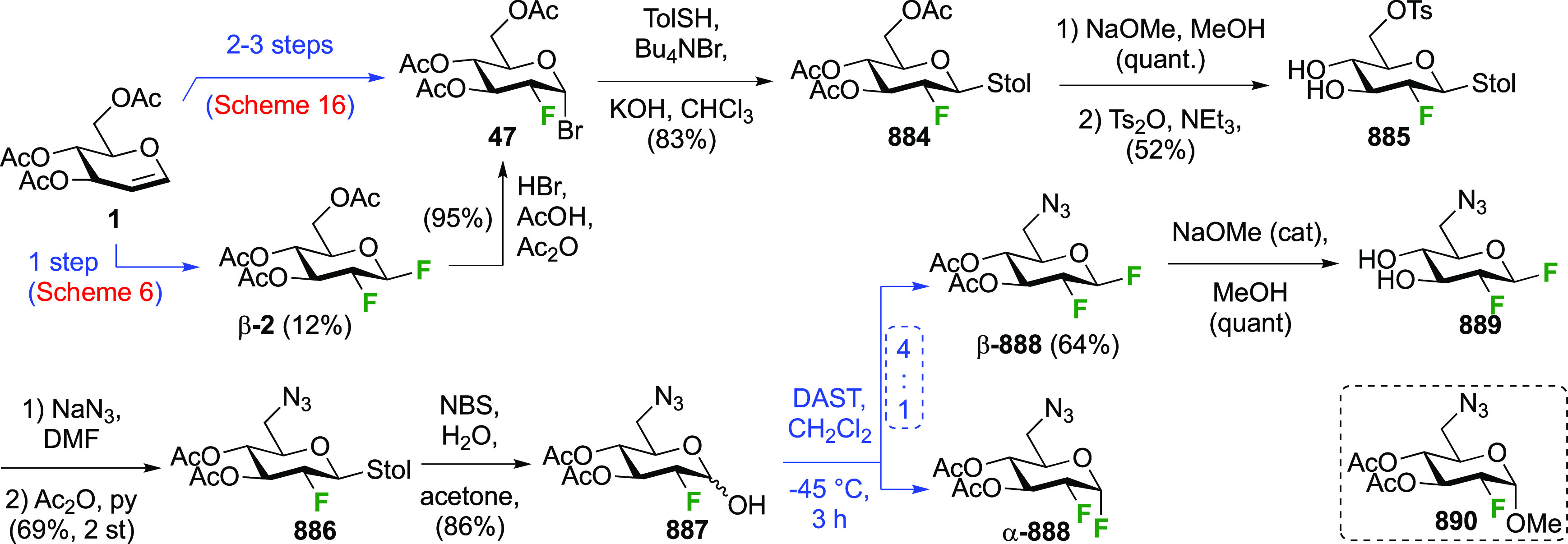
A Sequential Fluorination Approach to a 1,2-Difluorinated-6-azido Glucose Derivative^426,427^

The Van der Marel/Overkleeft and Wright groups both independently reported the synthesis of 6-azido-2,6-deoxy-2-fluoro-β-d-glucosyl fluoride **889** from tri-*O*-acetyl d-glucal **1** (Scheme 126),^426,427^ which essentially only differs in the aromatic thiol used to protect the anomeric position (*p*-tolyl vs *p*-chlorophenyl). The Van der Marel/Overkleeft synthesis is shown here. The fluorine at the 2-position was introduced first, from reaction of the glucal **1** with SelectFluor (as shown in Scheme 16) to give **47**.^426^ The Wright group synthesized **47** via β-**2**, which can be synthesized from **1** with XeF_2_ (as shown in Scheme 6) through reaction with HBr.^427^ A 2-step synthesis of **47** from 2-deoxy-2-fluoroglucose is also possible, as shown in Scheme 9 (not shown). Protection of the anomeric position as the thioglycoside **884** was followed by deacetylation and selective activation of OH-6 to give **885**, which allowed azide introduction.^426^ Reprotection of the remaining alcohols as acetates (**886**) was then required to allow, after anomeric deprotection to **887**, DAST-mediated glycosyl fluoride formation. This led to a 4:1 anomeric ratio of **888**, from which the desired β-anomer was isolated in 64% yield. Deprotection of β-**888** with a catalytic amount of NaOMe led to **889** in quantitative yield. The use of a stoichiometric amount of NaOMe was reported to lead to a substantial amount of anomeric substitution to give **890**.

**Scheme 127:**
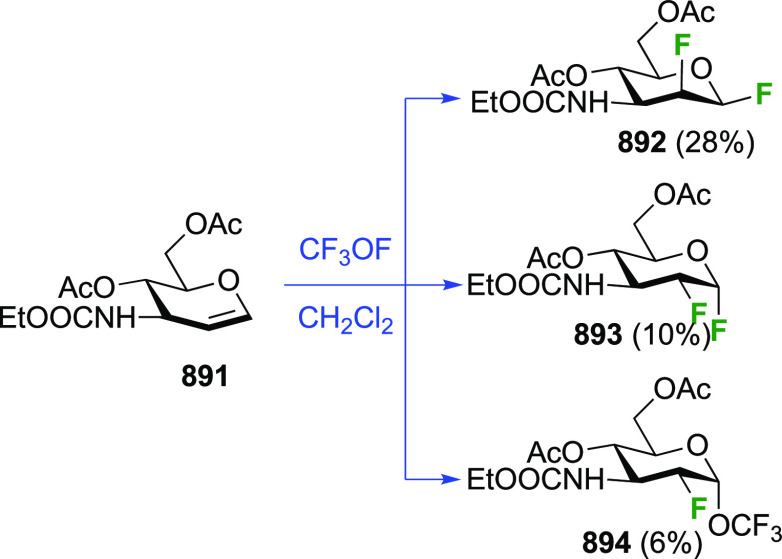
A Direct Difluorination Approach to 1,2-Difluorinated-3-amino Mannose and Glucose Derivatives^419^

The Jordaan group investigated the protected 3-deoxy-3-amino glucal **891**(428) as a substrate for reaction with CF_3_OF (Scheme 127).^419^ This led to the mannosamine derivative **892** as the major and the glucosamine derivative **893** as the minor product, next to the formation of the trifluoromethyl glucoside **894**. This result indicates a different facial selectivity compared to the corresponding tri-*O*-acetyl-d-glucal as shown in Scheme 6.

#### 8.1.2 Fluorination at Positions 1 and 3

**Scheme 128:**
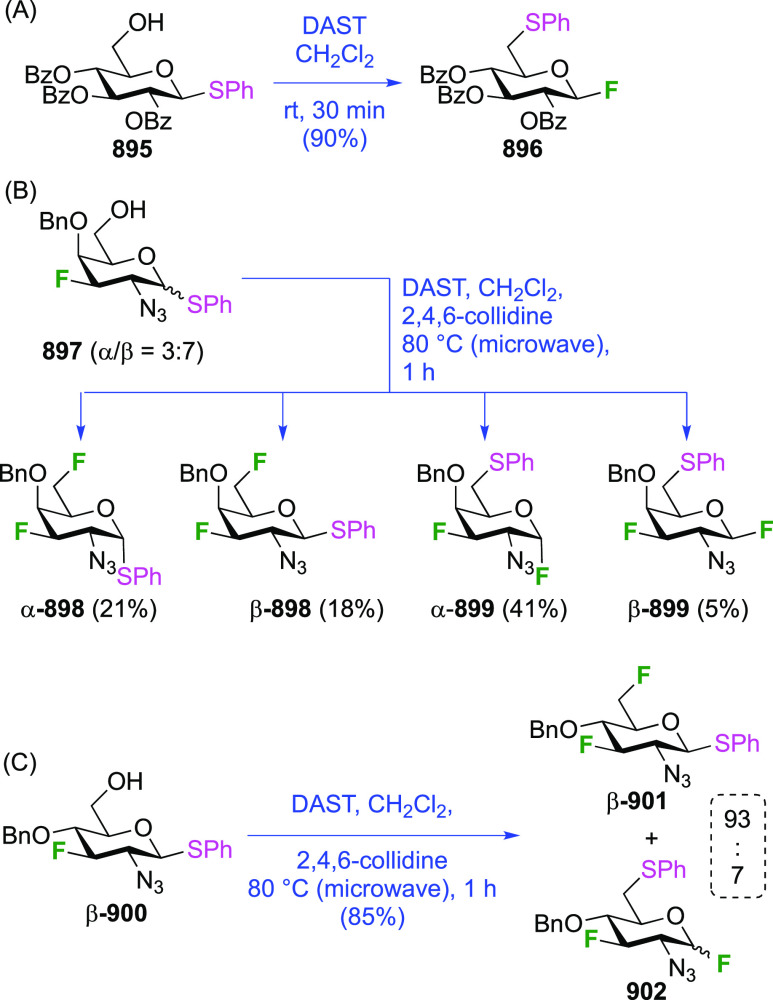
Formation of 1,3-Difluorinated-2-Azidohexopyranoses Based on a DAST-Induced 1 → 6 Migration Process^429,430^

There are no dedicated syntheses of 1,3-difluorinated aminosugars, but it is worth mentioning observations by the Karban group regarding a 1 → 6 migration process when deoxyfluorinating the 6-position of β-configured thioglycosides (Scheme 128).^429^ This process, originally described with a 2-*O*-benzoylated β-configured methyl galactoside,^145^ had been expanded by the Lin group (with β-thiophenolates), where they showed that high yields of migration can be achieved, for example in the conversion of **895** to **896** (Scheme 128A).^430^ Upon treatment of a mixture of anomers **897** (Scheme 128B, see below Scheme 139 for their synthesis), the Karban group isolated four products,^429^ with α-**898** arising from clean deoxyfluorination of the α-anomer of **897**, while β-**898**, α-**899**, and β-**899** arose from the β-anomer of **897**. The major product was the migration product α-**899**. However, subjecting β-**900**, the *gluco*-configured diastereomer of β-**897** (Scheme 128C), to the same deoxyfluorination conditions did not lead to much migration, with only 7% of **902** observed in the mixture of inseparable products. This reflects the higher ability of the electron withdrawing equatorial substituent to destabilize a positive charge at the anomeric position, as extensively investigated by the Bols group.^431,432^

#### 8.1.3 Fluorination at Positions 1 and 4

**Scheme 129:**
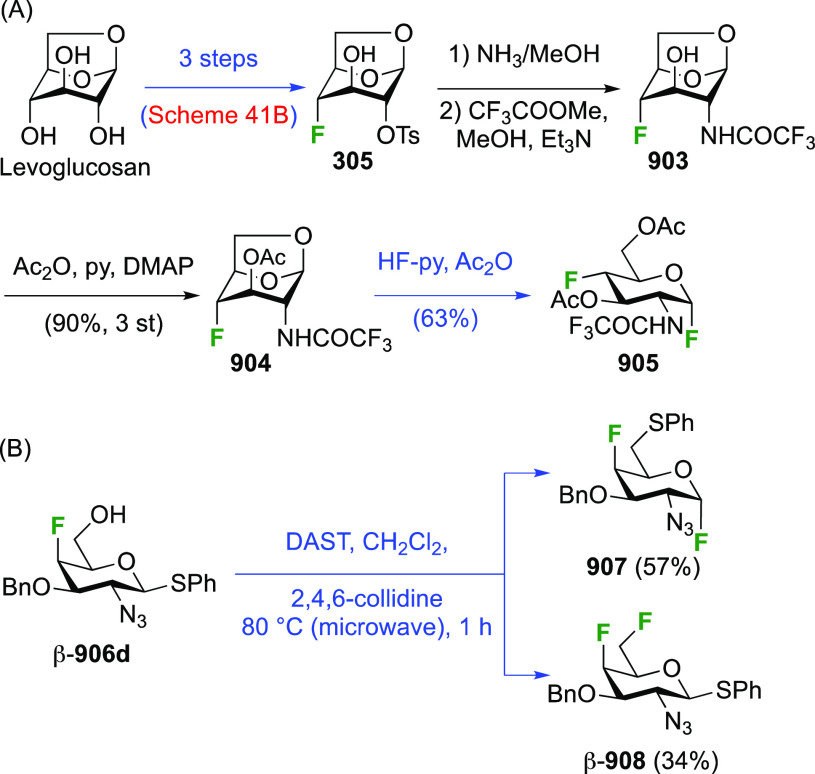
Synthesis of 1,4-Difluorinated Gluco- and Galactosamine Derivatives^294,429^

The Voznyi group reported a synthesis of a 1,4-difluorinated glucosamine derivative **905** from levoglucosan (Scheme 129A).^294^ After a 3-step conversion of levoglucosan to **305**, as shown in Scheme 41B, treatment with ammonia initiated 2,3-anhydro formation and subsequent opening with ammonia at the usual 2-position. The resulting amine was immediately protected as trifluoroacetamide **903**, followed by alcohol protection as acetate **904**. 1,6-Anhydro-bridge opening with direct formation of a glycosyl fluoride was achieved with Olah’s reagent in acetic anhydride,^433^ to yield target **905**. This transformation is usually achieved in a 2–3 step operation involving anhydro opening followed by glycosyl fluoride introduction.

Again, it is worth mentioning a 1 → 6 migration reaction (cf. Scheme 128) reported by the Karban group, now starting from 4-fluorinated β-configured thioglycoside **906d** (Scheme 129B).^429^ Hence, reaction of β-**906d** (see below, Scheme 143 for its synthesis) with DAST under microwave irradiation delivers the 1,4-difluorinated GalNAc derivative **907** in 57% yield, alongside 34% of the direct deoxyfluorination product **908**.

#### 8.1.4 Fluorination at Positions 1 and 5

**Scheme 130:**
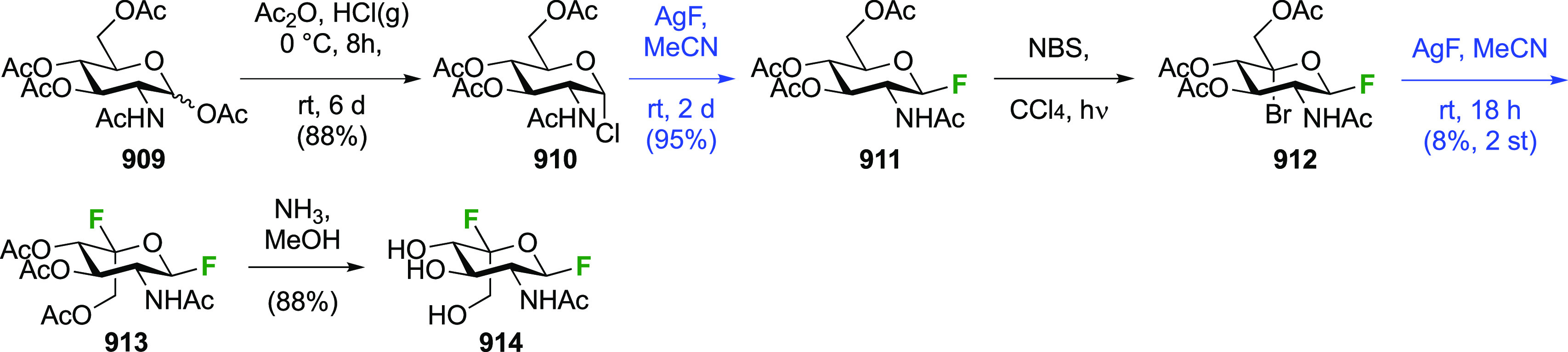
Synthesis of a 1,5-Difluorinated l-Idosamine Derivative^434^

The Withers group reported the synthesis of a 1,5-difluorinated idosamine derivative **914** (Scheme 130).^434^ Peracetylated glucosamine **909** was treated with HCl in acetic anhydride for >6 days to yield the α-glycosyl chloride **910**,^435−437^ the reaction of which with AgF in acetonitrile for 2 d at room temperature gave the β-glycosyl fluoride **911**.^438^ Radical bromination (cf. section 3.4) led to the unstable **912**, which was directly subjected to AgF in acetonitrile to effect bromide displacement with inversion of configuration to give the l-idosyl fluoride **913**. This was subsequently deprotected to yield 2-acetamido-2-deoxy-5-fluoro-α-l-idosyl fluoride **914**.^434^

**Scheme 131:**
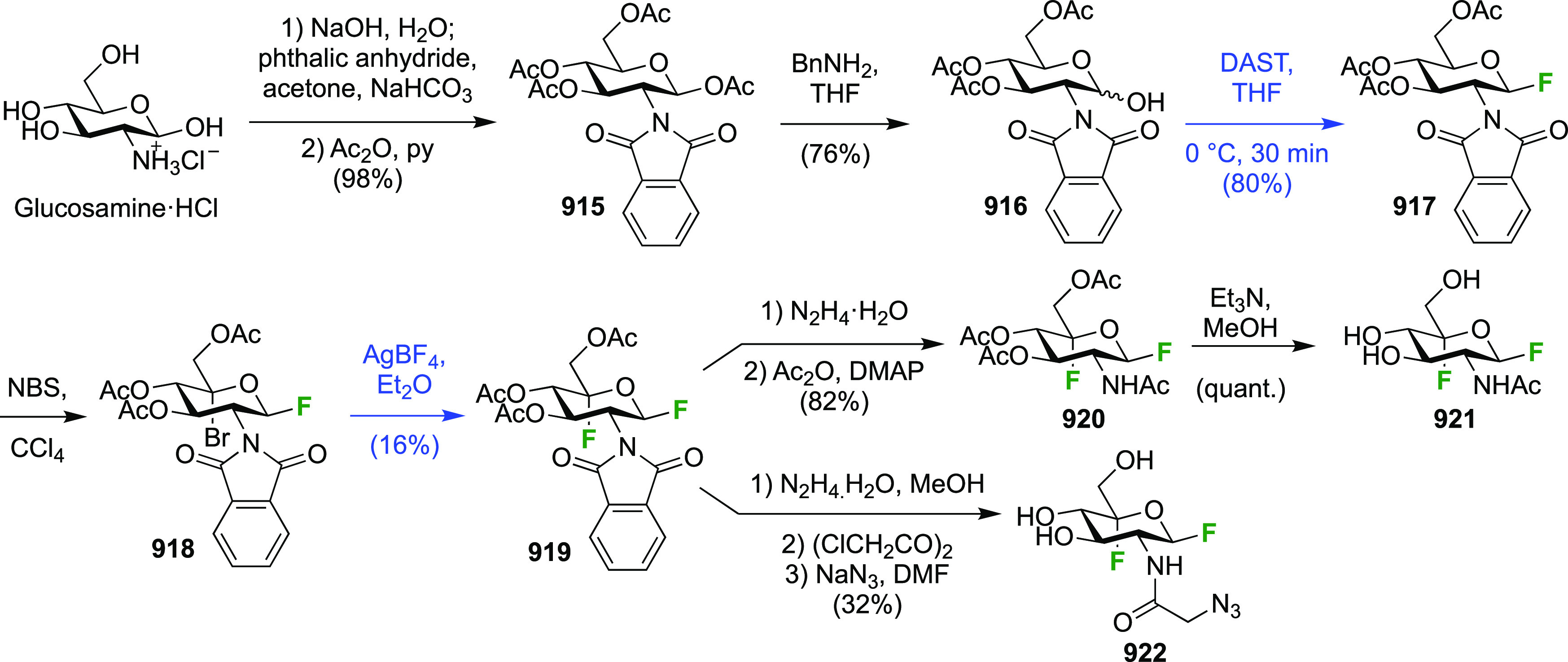
Synthesis of 1,5-Difluorinated GlcNAc Derivatives^439^

The Vocadlo group has synthesized the corresponding glucosamine derivatives **921** and **922** (Scheme 131).^439^ As already indicated in section 3.4 for the synthesis of 1,5-difluorinated derivatives, fluorination at C1 is required before fluorination at C-5. Hence, **916** was targeted as the first deoxyfluorination substrate and was obtained by anomeric deprotection of the per-*O*-acetate **915**. This can be synthesized from glucosamine hydrochloride via a temporary amine protection as the *p*-methoxybenzylidene imine in four steps,^439,440^ but a shorter, higher yielding, 2-step process as shown is now available from glucosamine.^441^ DAST-mediated deoxyfluorination of **916** gave the β-glycosyl fluoride **917** as the only reported anomer in excellent yield.^439^ Radical bromination at the 5-position was followed by retentive bromide displacement with the AgBF_4_/Et_2_O conditions to give **919** in modest yield. Amine deprotection with hydrazine was possible without affecting the anomeric fluoride group, and subsequent acetylation followed by global deprotection afforded the desired 5-fluorinated glycosyl fluoride probe **921**. A similar process using chloroacetic acid anhydride for the acetylation, and subsequent chloride displacement with azide gave **922**.

#### 8.1.5 Fluorination at Positions 1 and 6

**Scheme 132:**
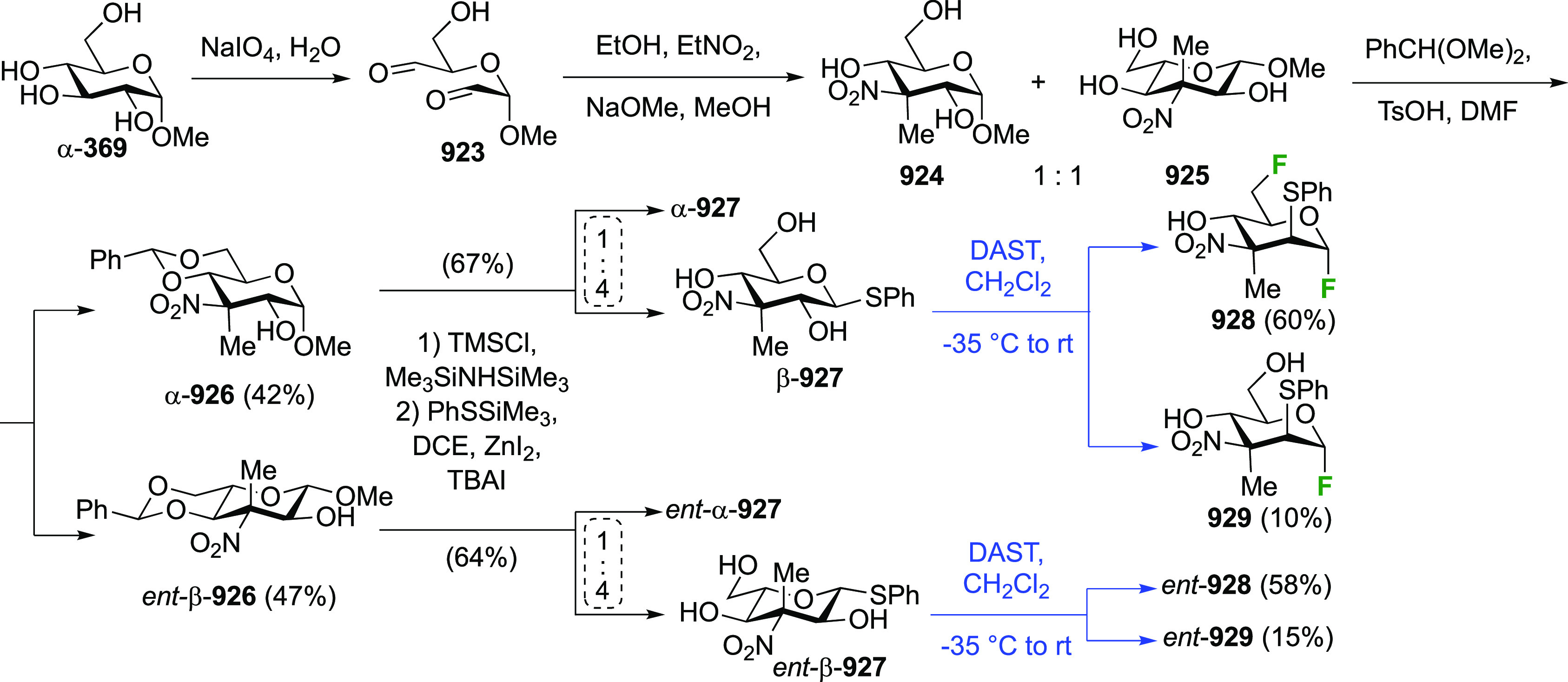
Investigation of Direct DAST-Mediated Difluorination on a Branched Nitrosugar Derivative^299,442,443^

The Cabrera-Escribano group investigated deoxyfluorination reactions on branched nitrosugars, which were synthesized by the Baer reaction.^299,442,443^ Methyl α-d-glucopyranoside α-**369** (Scheme 132) was subjected to periodate cleavage to the dialdehyde **923**, whereupon treatment with nitroethane in basic medium led to a 1:1 mixture of inseparable **924** and **925**. The synthesis of the latter involves epimerization at C-5.^444^ The stereomers were separated after formation of the benzylidene acetals α-**926** and *ent*-β-**926**. Each was then converted to its corresponding thiophenyl glycoside **927**, and this led in both cases to a 4:1 β/α mixture, with the compounds coming from α-**926** being enantiomeric to those arising from *ent*-β-**926**. Treatment of β-**927** led to a mixture with 6-deoxy-6-fluoro α-glycosyl fluoride **928** as the major product, in which the thiophenolate had rearranged to the 2-position, and **929**, in which only a reaction at the anomeric center had taken place.^442^ This result was confirmed with *ent*-β-**927** as starting material.

#### 8.1.6 Fluorination at Positions 2 and 3

**Scheme 133:**
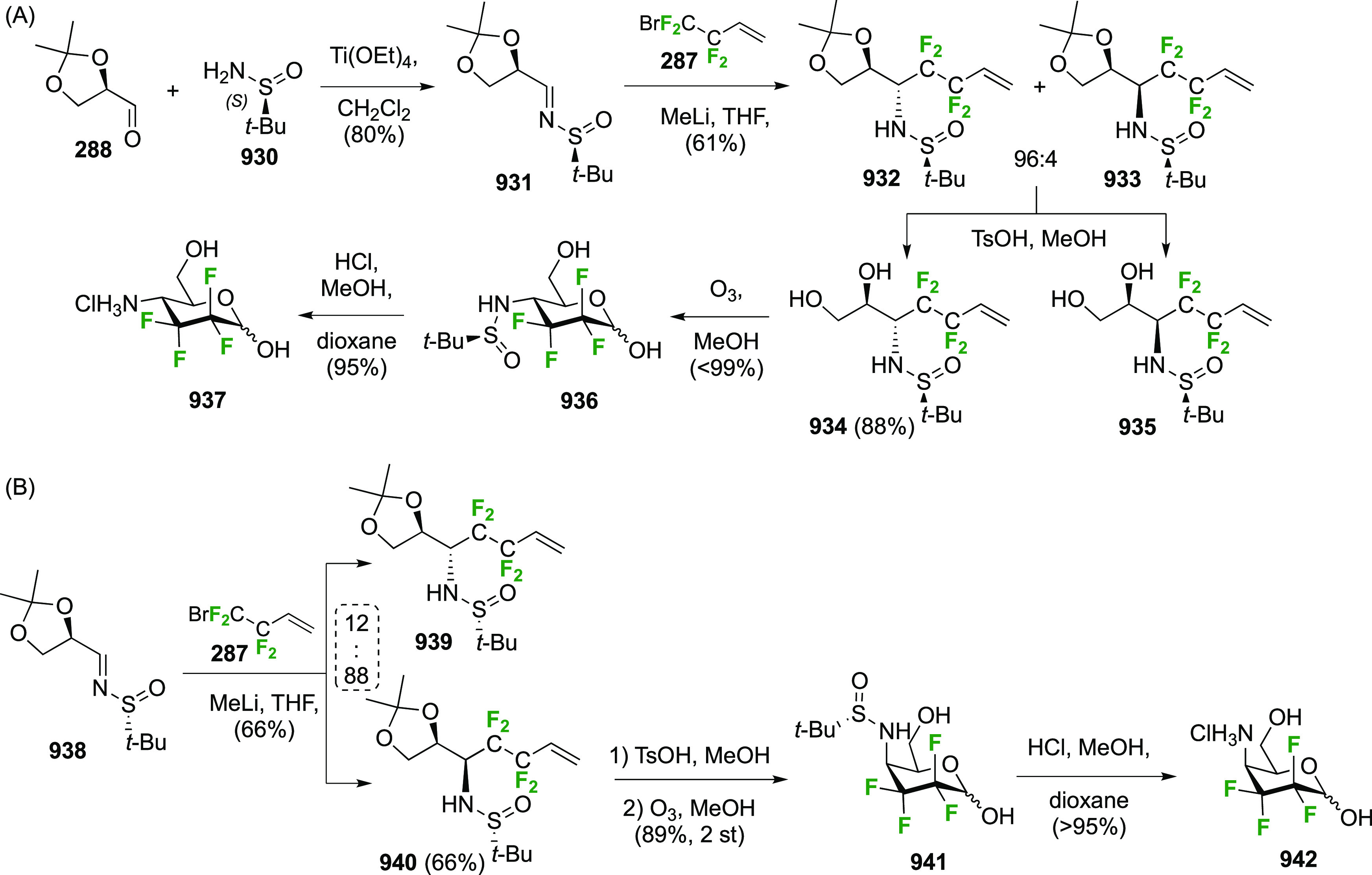
Synthesis of Tetrafluorinated Aminosugar Derivatives^445^

The Linclau group synthesized the tetrafluorinated aminosugars **937** and **938**, with the addition of lithiated **287** to the sulfinylimines **931** and **938** as the key step.^445^ This methodology had been developed by the Konno group, with a demonstration of the addition of lithiated **287** to the corresponding sulfinylimine of benzaldehyde.^446^ Hence, with **931** as substrate (Scheme 133A), synthesized from d-glyceraldehyde **288** and the sulfinamide **930**, *syn* and *anti*-adducts **932** and **933** were obtained in excellent diastereoselectivity. These could be separated after acetonide methanolysis as **934** and **935**, respectively. A minor (±3%) side product arising from S_N_2′ substitution of a fluoride by methyl lithium was also formed (not shown). The major isomer **934** was obtained in 88% isolated yield, upon which alkene ozonolysis and auxiliary cleavage gave the aminosugar **937**, which was isolated as its hydrochloric acid salt.

Based on the observation by the Linclau/Poisson groups that stereocontrol in additions to sulfinylimines derived from glyceraldehyde acetonide is exerted by the auxiliary configuration,^447^ addition with **938** was investigated (Scheme 133B) as well. As expected, the *syn*-adduct **940** was now the major stereomer, which was subsequently converted to the 4-*epi* aminosugar **942** in the same way as shown above.^445^

#### 8.1.7 Fluorination at Positions 2 and 4

**Scheme 134:**
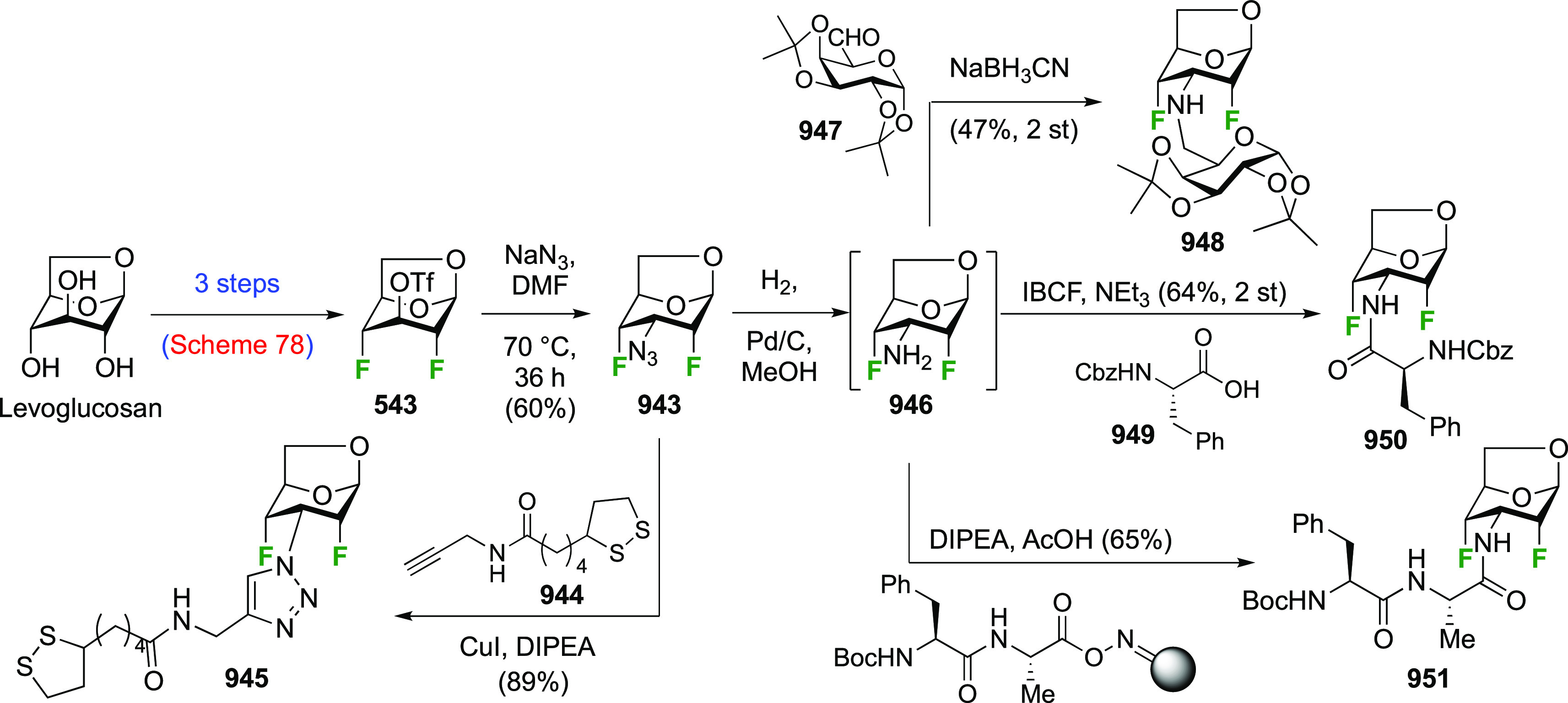
Synthesis of Functionalized 2,4-Difluorinated 3-Amino-1,6-anhydrohexopyranose Derivatives^295,448^

The Giguère group reported the synthesis of a number of 1,6-anhydro-2,4-dideoxy-2,4-difluoroallose derivatives via functionalization at C-3 (Scheme 134).^295,448^ Using the triflate **543** as a key intermediate, for which the synthesis was discussed in Scheme 78, the azide **943** could be prepared as a first handle for functionalization via a click reaction to obtain the lipoic acid fluorinated glycoconjugate **945**.^295^ The azide could also be reduced to the corresponding amine **946** as a versatile intermediate for further functionalization. Reductive amination with aldehyde **947** (derived from galactose diacetonide) gave the amino-linked fluorinated disaccharide **948**, whereas peptide coupling with Cbz-protected phenylalanine **949** using isobutyl chloroformate (IBCF) as the coupling agent yielded **950**.^295^ It was also demonstrated that oxime resin aminolysis was possible, leading to the C-terminal fluoroglycopeptide **951**.^448^

#### 8.1.8 Fluorination at Positions 2 and 5/3 and 5

**Scheme 135:**
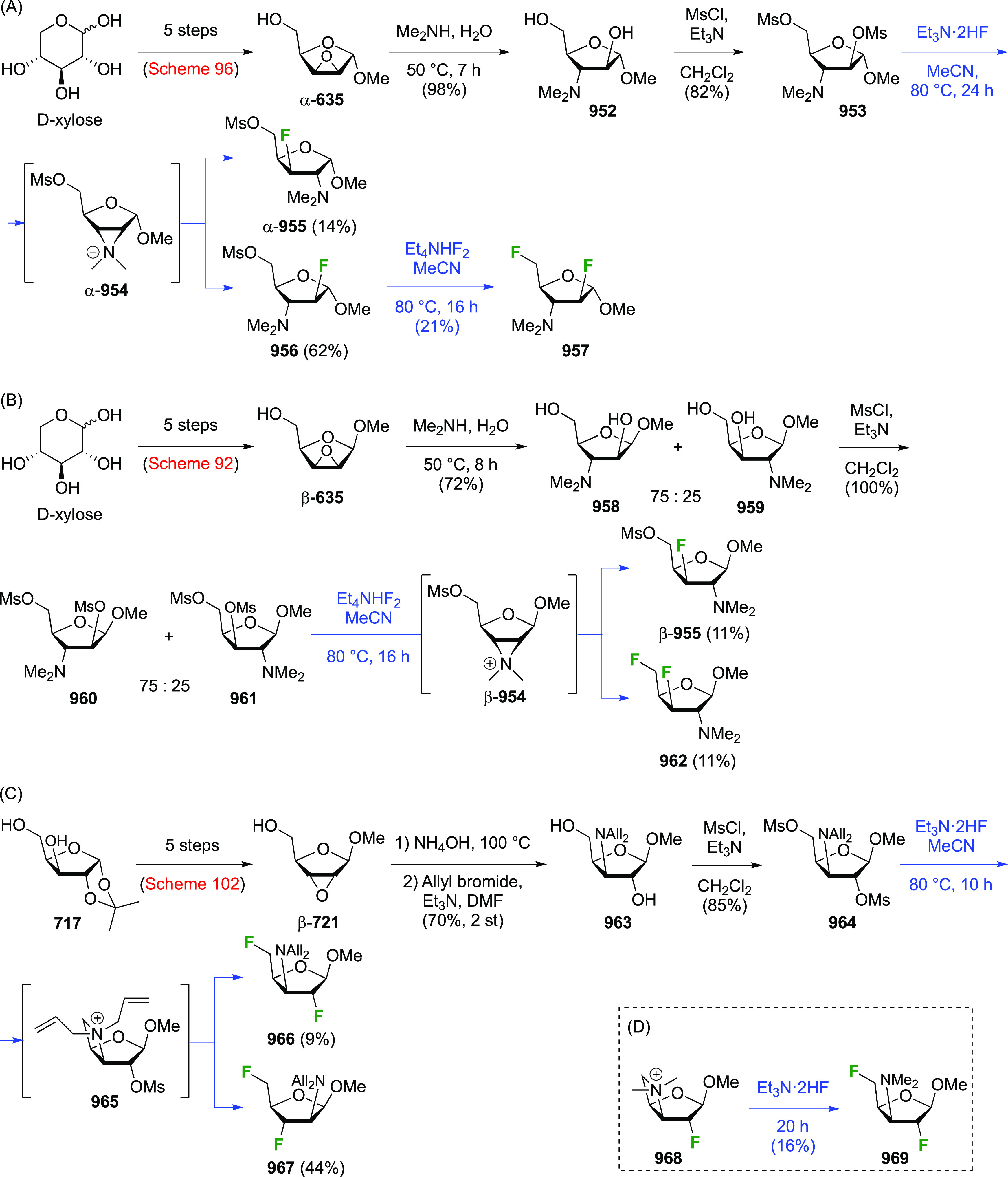
Investigations on the Fluorination of Aminopentofuranoside Derivatives^449,450^

The Anker group investigated the fluorination of aminosugars, and results toward difluorinated pentoses are given in Scheme 135.^449,450^ Methyl 2,3-anhydro-β-d-lyxofuranoside α-**635**, whose synthesis in five steps from d-xylose was discussed in Scheme 96, was converted to the *N*,*N*-dimethyl aminosugar derivative **952** with full regioselectivity.^450^ Following hydroxyl group mesylation, treatment with Et_3_N·2HF initially gave rise to formation of aziridinium species α-**954**, whereupon nucleophilic substitution with fluoride took place with moderate regioselectivity to give α-**955** and **956**. Fluorine substitution at the 5-position, reported to be difficult, only proceeded in low yield with the more reactive tetraethylammonium hydrogen difluoride to yield **957**.^450^ Direct treatment of **953** with Et_4_NHF_2_ only returned a complex reaction mixture.

With the β-anomer of methyl 2,3-anhydro-d-lyxofuranoside β-**635**, obtained in five steps from d-xylose as discussed in Scheme 92, the epoxide opening with dimethyl amine was less selective (Scheme 135B), giving a 75:25 ratio of inseparable **958** and **959**.^450^ Mesylation of this mixture led to another inseparable mixture of **960** and **961**. These converged to the same aziridinium ion β-**954** upon treatment with Et_4_NHF_2_, which underwent regioselective opening with fluoride, with some fluorination at C-5 as well, to give β-**955** and **962** in modest yields.^450^

With the 2,3-anhydroribofuranoside substrate β-**721** (Scheme 135C), obtained in five steps from the commercially available **717** as discussed in Scheme 102, regioselective epoxide opening with ammonia followed by protection as the diallyl amine led to **963**. Mesylation of the alcohols to **964** was followed by fluorination, which now proceeded first through azetidinium intermediate **965**, leading first to fluorination at the 5-position. Subsequent fluorination proceeded then via an aziridinium intermediate, giving both the F-2 and F-3 products **966** and **967**.^450^ Nevertheless, it was stressed that C-5-fluorination remained difficult. The azetidinium ion derived from the dimethylamino group, **968** (Scheme 135D), was reported to be isolable and stable, and only 16% of the 2,5-difluorinated **969** was obtained after fluorination.^449^ The enhanced reactivity of **965** was ascribed to steric strain between an allyl group and the anomeric methoxy group.

#### 8.1.9 Fluorination at Positions 3 and 4

**Scheme 136:**
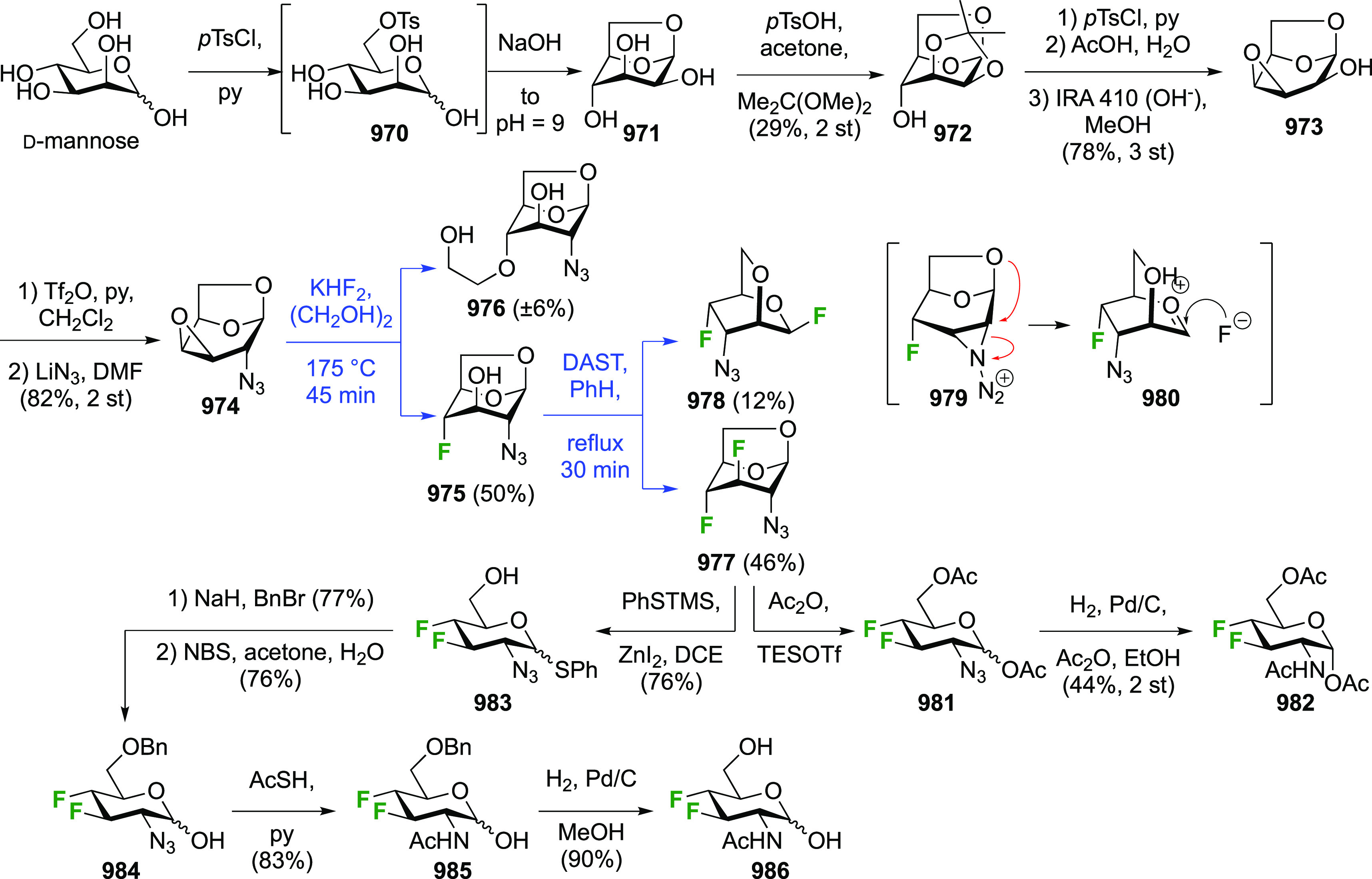
Synthesis of 3,4-Difluorinated GlcNAc Derivatives^429,451^

The Karban group reported the synthesis of 3,4-dideoxy-3,4-difluorinated GlcNAc and GalNAc derivatives using a sequential fluorination approach.^451^ The GlcNAc synthesis (Scheme 136) commenced from d-mannose, which was converted first to 1,6-anhydro-β-d-mannose **971** and then its acetonide **972** using the Fraser-Reid procedure.^452^ Tosylation of the alcohol,^453^ acetonide hydrolysis,^454^ and intramolecular tosylate substitution^455^ resulted in 1,6:2,3-dianhydrotalose **973**. Activation of the OH-2 as a triflate allowed azide introduction to give **974**, in which the use of LiN_3_ proved far superior compared to NaN_3_ (82% vs 48%).^456^ This was subjected to fluoride opening to give **975**, with the side-product **976** resulting from epoxide opening by the solvent also isolated. The second fluorine introduction by DAST-mediated retentive deoxyfluorination gave the desired **977** alongside rearrangement product **978**.^451^ This rearrangement is initiated by neighboring group participation of the azido group, leading to **979**. Fluoride attack at C-3 then leads to **977**, while a second neighboring group participation from O6 leads to **980**, upon which fluoride attack at the anomeric center results in **978**. From **977**, acetolysis to open the anhydro-bridge followed by azide reduction and acetylation afforded 2-acetamido-1,6-di-*O*-acetyl-2,3,4-trideoxy-3,4-difluoro-α-d-glucopyranose **982**. Alternatively, 1,6-anhydro opening by phenyl trimethylsilyl sulfide (PhSTMS) under ZnI_2_ catalysis afforded the thioglycoside **983**,^429^ which was then subjected to OH-6 protection and anomeric deprotection to give **984**. Azide reduction with concomitant acetylation and benzyl hydrogenolysis finally afforded 3,4-dideoxy difluorinated GlcNAc **986**.

**Scheme 137:**
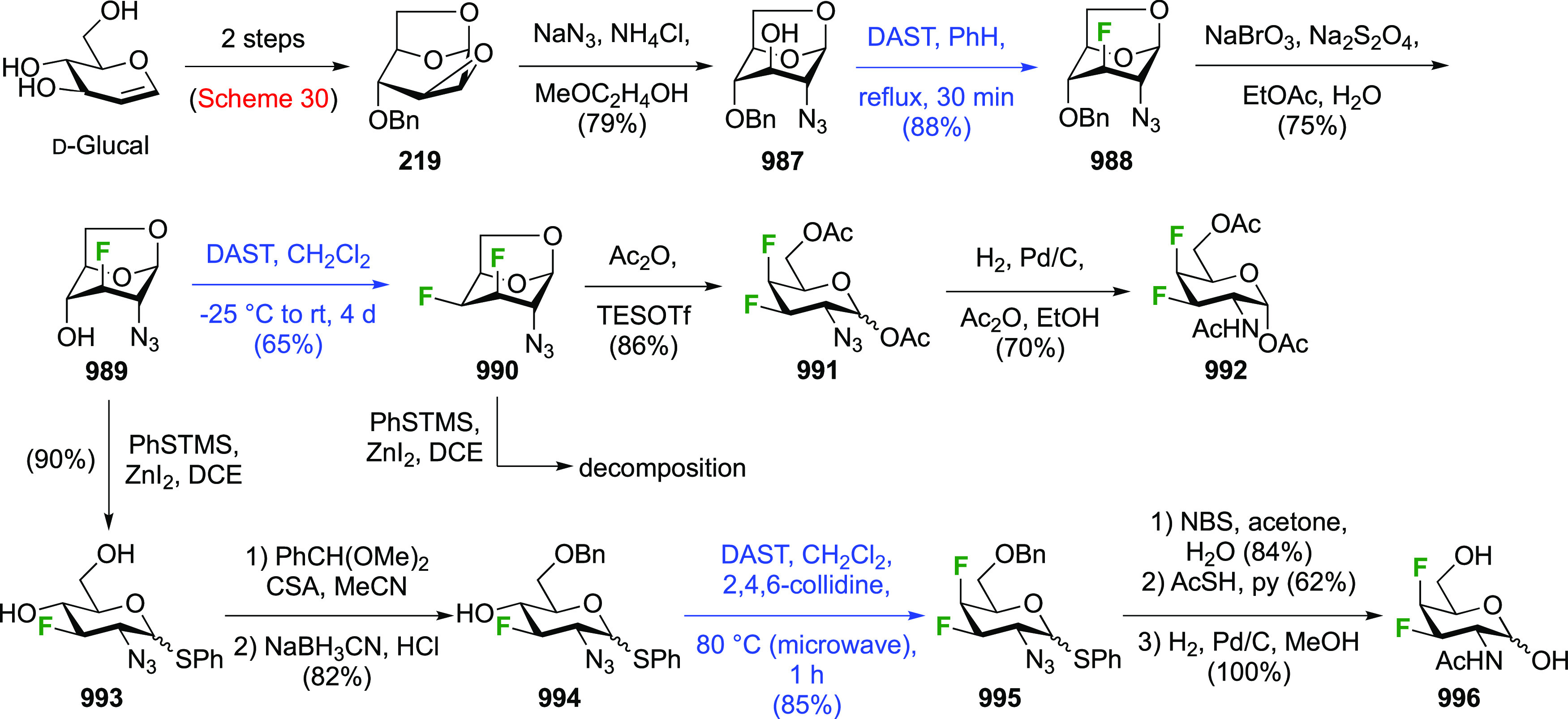
Synthesis of 3,4-Difluorinated GalNAc derivatives^429,451^

The syntheses of the corresponding 3,4-difluorinated GalNAc derivatives proceed along similar lines (Scheme 137). The 4-*O*-benzylated 1,6:2,3-dianhydromannose **219**, obtained in two steps from d-glucal as discussed in Scheme 30, was subjected to azide opening to give **987**.^457^ DAST-mediated retentive deoxyfluorination gave **988** in excellent yield without any rearrangement,^451^ in contrast to the deoxyfluorination of **975**. Presumably this is due to the availability of the benzyloxy group for neighboring group participation, possibly outcompeting the azido group (however see Scheme 125). Oxidative debenzylation then allowed a second deoxyfluorination, now with inversion of configuration, to give **990**. Acetolysis and azide reduction/acetylation then gave 2-acetamido-1,6-di-*O*-acetyl-2,3,4-trideoxy-3,4-difluoro-α-d-galactopyranose **992**.

Unlike the corresponding *gluco*-configured analogue **977** (see Scheme 136), treatment of **990** with PhSTMS/ZnI_2_ was reported to lead to decomposition.^429^ Hence, **989** was subjected to PhSTMS/ZnI_2_ instead, leading to **993**. This was then converted to the 6-*O*-benzyl ether **994** to allow deoxyfluorination at C-4, which proceeded smoothly with the expected inversion of configuration to give **995**. Anomeric deprotection, azide reduction/acetylation, and benzyl hydrogenolysis finally afforded 3,4-dideoxy difluorinated GalNAc **996**.

#### 8.1.10 Fluorination at Positions 3 and 6

**Scheme 138:**
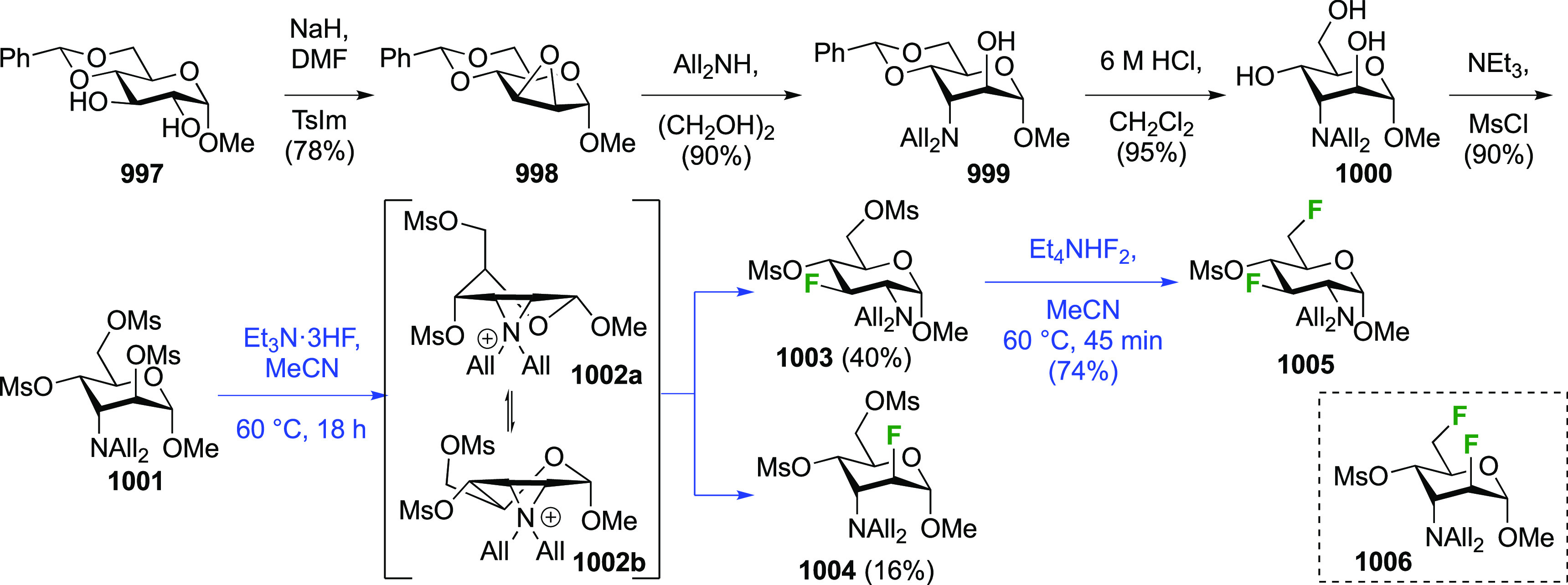
Investigations on the Fluorination of 3-Amino Altropyranoside Derivatives^461^

The Picq/Anker group used the aziridinium-mediated fluorination approach, as described in Scheme 135, for the synthesis of 2,5/3,5-difluorinated pentosamine derivatives. Conversion of **997** (Scheme 138) to the 2,3-anhydro derivative **998** using the Fraser-Reid procedure^423^ was followed by regioselective epoxide opening with diallyl amine.^458^ The resulting **999** was then hydrolyzed to give **1000**,^459^ and converted to its tri-*O*-mesylate **1001**.^460^ Treatment of **1001** with Et_3_N·3HF at 60 °C led to a mixture of the 3-fluorinated glucosamine derivative **1003** and the 2-fluorinated altrose derivative **1004**,^461^ an outcome that can be explained by invoking an aziridinium intermediate. Given that **1003** is the major product, this must primarily react via the half-chair **1002a**. Reaction of **1003** with the more reactive Et_4_NHF_2_ then gave the 3,6-difluorinated derivative **1005**. In an earlier publication, it was reported that heating **1001** with Et_3_N·3HF at 75 °C for 4 h gave **1003** in 73% yield, with no mention of any formation of **1004**,^460^ although it was later claimed that heating of **1001** with Et_3_N·3HF at 75 °C for 26 h led to a mixture of **1003**-**1006** without specification of yields.^461^

**Scheme 139:**
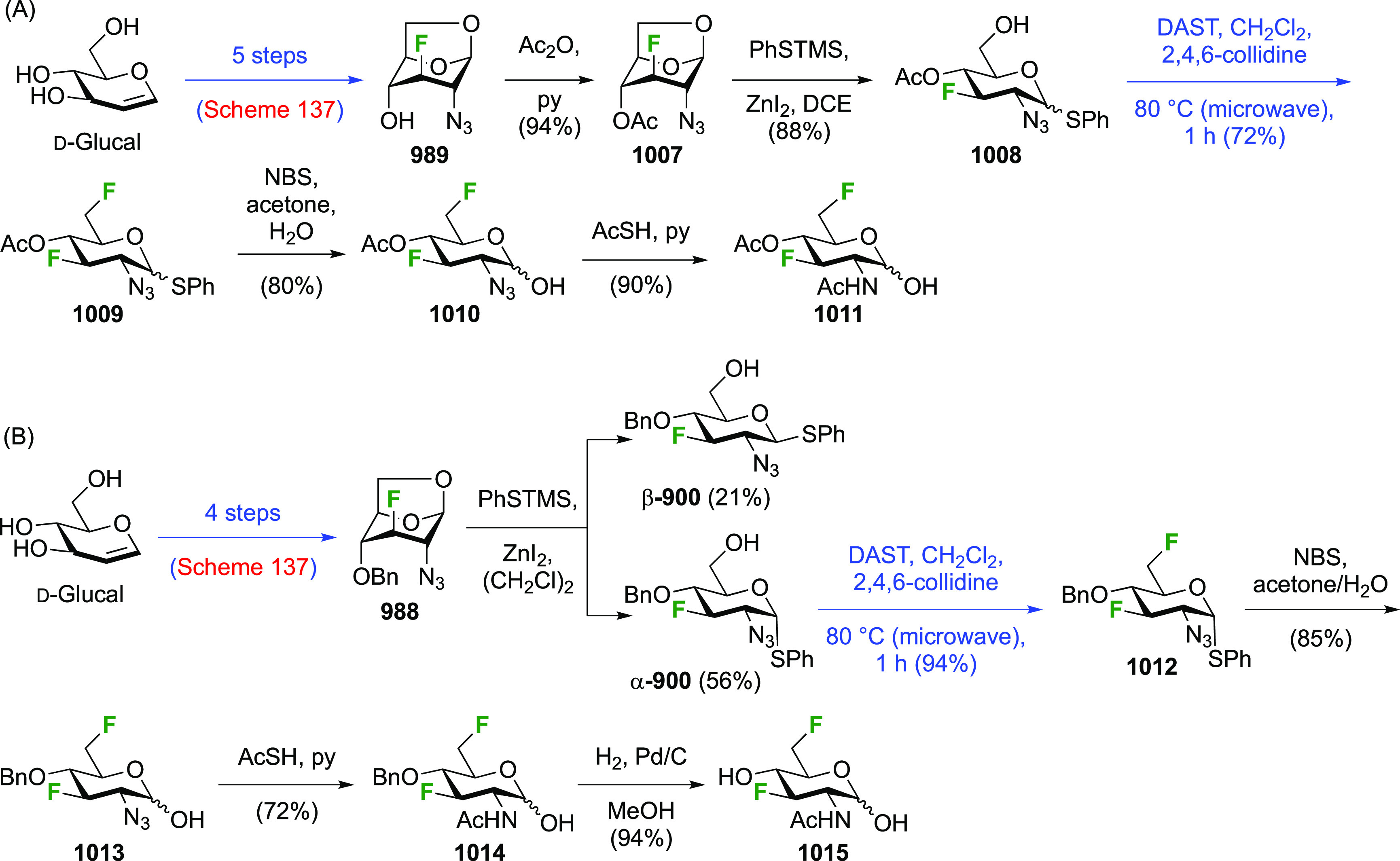
Synthesis of 3,6-Difluorinated GlcNAc Derivatives^429,463^

The Karban group reported an approach to 3,6-dideoxy-3,6-difluorinated GlcNAc derivatives using 1,6-anhydro intermediates (Scheme 139). From **989**, for which the synthesis was reported in Scheme 137, acetylation and anhydro-bridge opening with PhSTMS gave **1008** as a mixture of anomers.^462^ The available OH-6 group was deoxyfluorinated to **1009**, and the anomeric position deprotected to give **1010**. Azide reduction with concomitant acetylation then gave **1011**.^463^

Alternatively, the 4-*O*-benzyl-protected 1,6-anhydro derivative **988**, obtained in four steps from d-glucal as discussed in Scheme 137, was treated with PhSTMS to obtain a separable mixture of thioglycoside anomers α-and β-**900**.^464^ These anomers were separated before deoxyfluorination, given the 1 → 6 migration side reaction of the β-thiophenyl anomer (see Schemes 128 and 129). Deoxyfluorination of the α-anomer led to **1012** in excellent yield,^462^ and subsequent anomeric deprotection, azide reduction/acetylation, and benzyl hydrogenolysis gave 2,3,6-trideoxy-2-acetamido-3,6-difluoroglucose **1015**.^429^

**Scheme 140:**
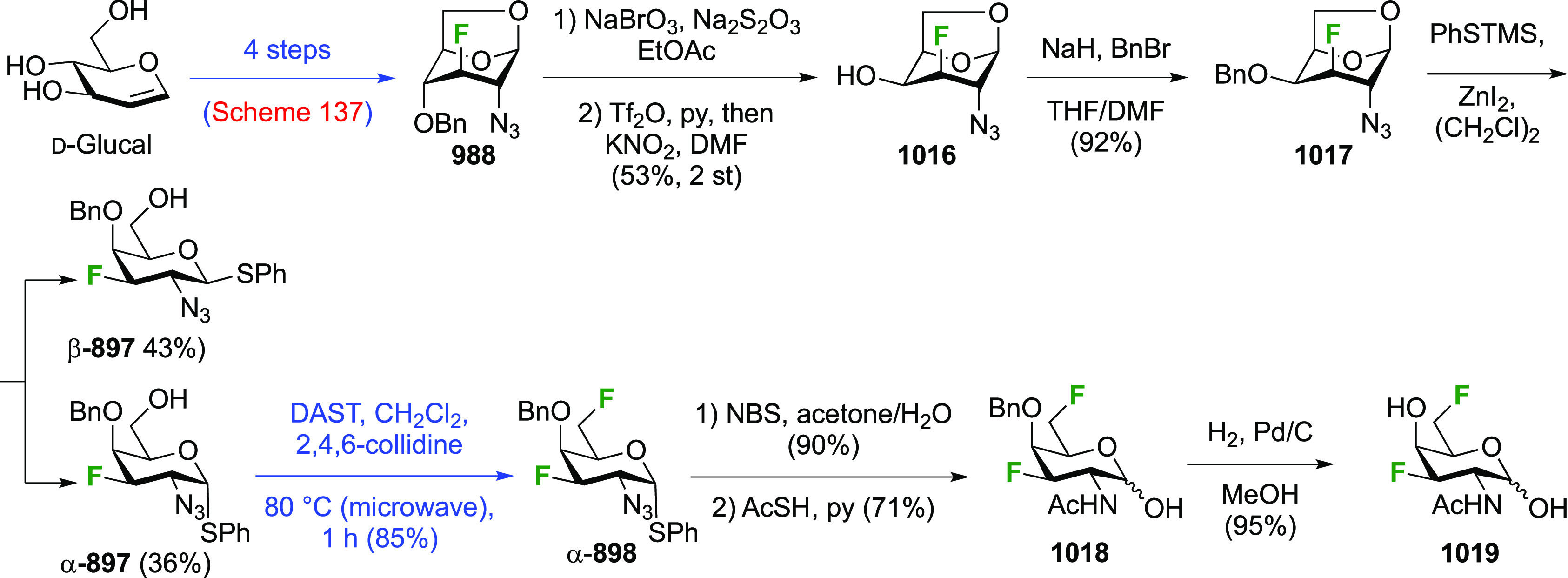
Synthesis of 3,6-Difluorinated GalNAc Derivatives^429^

The Karban group also developed similar a synthesis of 3,6-difluorinated GalNAc derivative **1019** (Scheme 140). Starting again from **988**, oxidative debenzylation followed by Lattrell-Dax inversion^276^ resulted in the *galacto*-derivative **1016**, which was protected as benzyl ether **1017**.^464^ Anhydro-bridge opening with PhSTMS gave a mixture of separable anomers **897**, and the α-anomer was subjected to deoxyfluorination to give α-**898**.^429^ Anomeric deprotection, azide reduction/acetylation, and benzyl hydrogenolysis then afforded 2,3,6-trideoxy-2-acetamido-3,6-difluorogalactose **1019**.

#### 8.1.11 Fluorination at Positions 4 and 6

##### 8.1.11.1 Galactose Stereochemistry

**Scheme 141:**
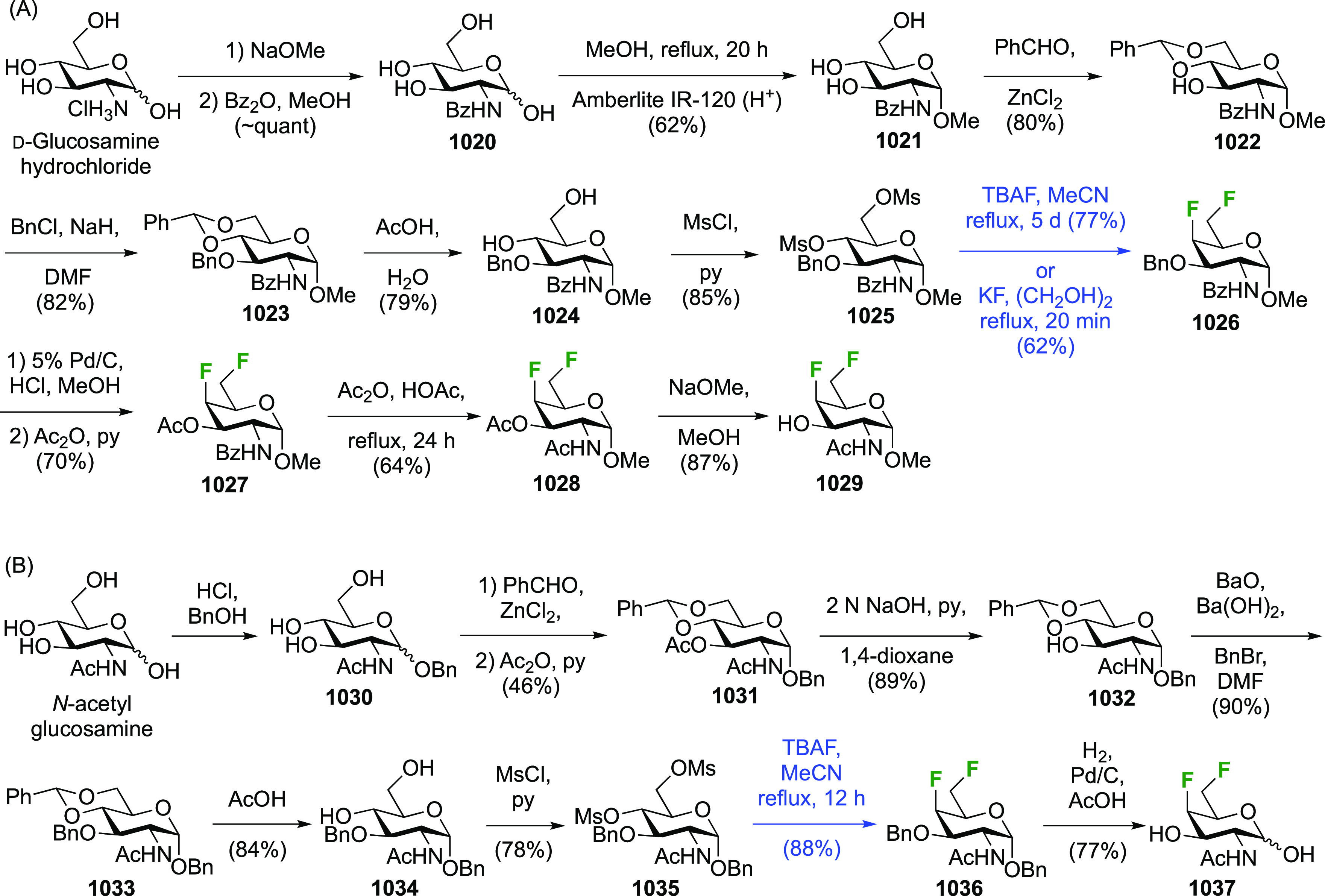
Synthesis of 4,6-Difluorinated GalNAc Derivatives via Mesylate Displacements^467,469^

The Richardson group reported the first synthesis of 4,6-difluorinated GalNAc using fluoride displacement of the required 4,6-di-*O*-mesylate, an approach already encountered in Scheme 61A for the synthesis of 4,6-dideoxy-4,6-difluorogalactose. Starting from d-glucosamine hydrochloride (Scheme 141A), treatment with NaOMe followed by benzoic anhydride gave **1020**.^465^ Its α-methyl glycoside **1021** was then obtained, and the 4,6-positions were protected as benzylidene acetal **1022**.^466^ Protection of OH-3 as benzyl ether **1023** was followed by benzylidene acetal hydrolysis to allow activation to the 4,6-di-*O*-mesylate **1025**.^467^ Fluoride substitution to give **1026** could be achieved with TBAF in refluxing acetonitrile or with KF in refluxing ethylene glycol, the latter having a much shorter reaction time. Replacing the 3-*O*-benzyl group with an acetate and the benzamide with acetamide gave, after acetate methanolysis, methyl 2,4,6-trideoxy-2-acetamido-4,6-difluorogalactoside **1029**.^467^

A similar synthesis was later published by the Korytnyk group (Scheme 141B). *N*-Acetyl glucosamine was converted to its benzyl glycoside **1030**, and further converted to its benzylidene acetal. This was acetylated for recrystallization purposes to give **1031** as pure α-anomer.^468^ The acetyl group was replaced with a benzyl, after which the benzylidene acetal was hydrolyzed to give **1034**.^469^ Reaction of **1034** with DAST was reported to be unsuccessful, owing to the low reactivity of the OH-4 group, so the alcohols were activated as mesylates, upon which reaction with TBAF in refluxing acetonitrile resulted in the formation of **1036** in excellent yield. Hydrogenolysis then gave 2,4,6-trideoxy-2-acetamido-4,6-difluorogalactose **1037**.

**Scheme 142:**
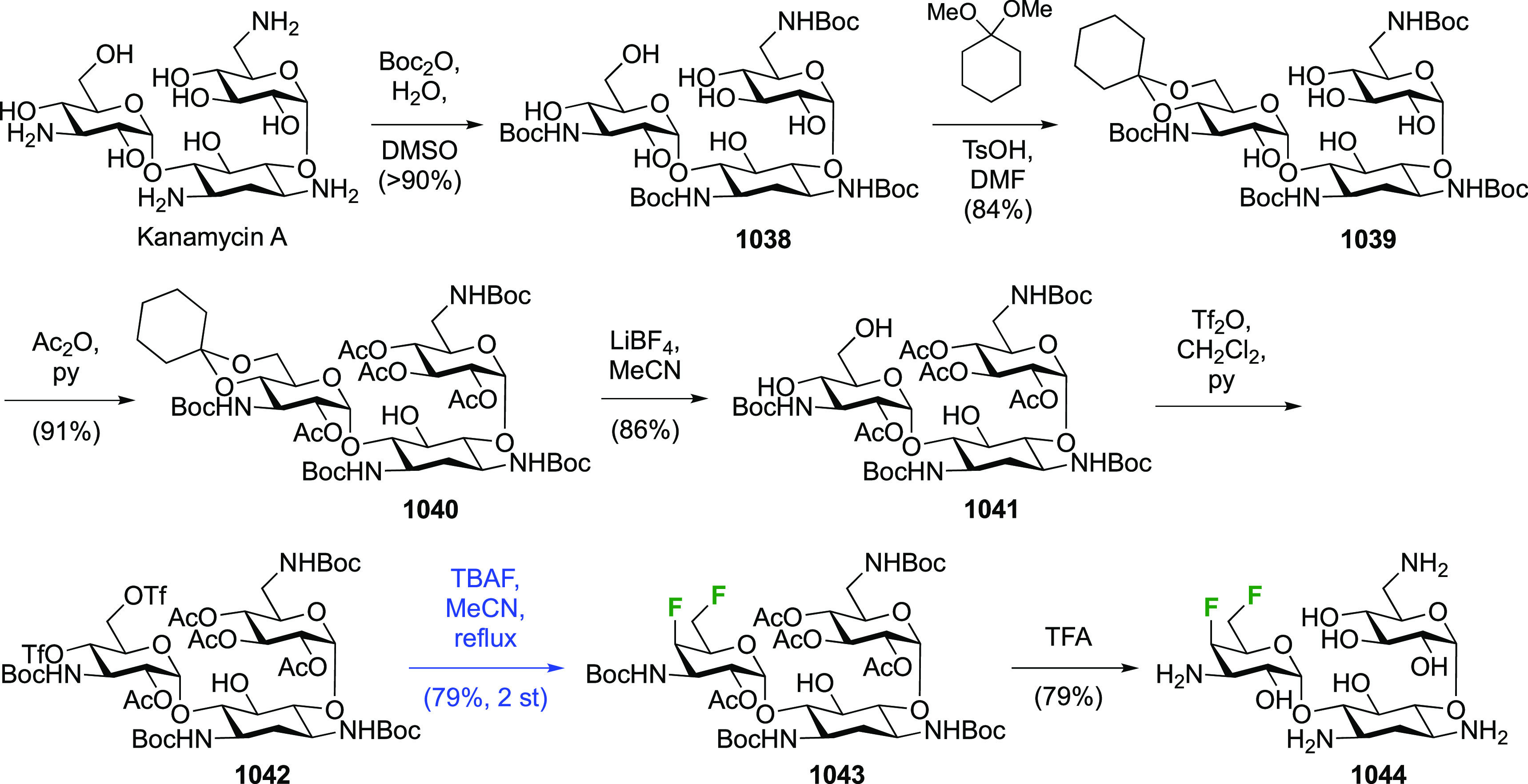
Synthesis of 4″,6″-Difluorinated Kanamycin A Derivative via Triflate Displacements^470^

The Dax group reported the synthesis of a 4″,6″-difluorinated kanamycin A derivative **1044** (Scheme 142) using a related approach.^470^ Starting from kanamycin A, the 4′′ and 6′′ positions were differentiated from the rest by first protecting the amino groups as their Boc derivatives, leading to **1038**.^471^ Cyclohexylidene acetal formation (**1039**) was followed by peracetylation of the remaining alcohol groups to give **1040**, upon which removal of the acetal group resulted in **1041**. The deprotected alcohol groups were then activated as triflates (**1042**), which allowed substitution with fluoride to give **1043**. Global deprotection then resulted in 4″,6″-dideoxy-4″,6″-difluoro-4″-*epi*-kanamycin A **1044**.

**Scheme 143:**
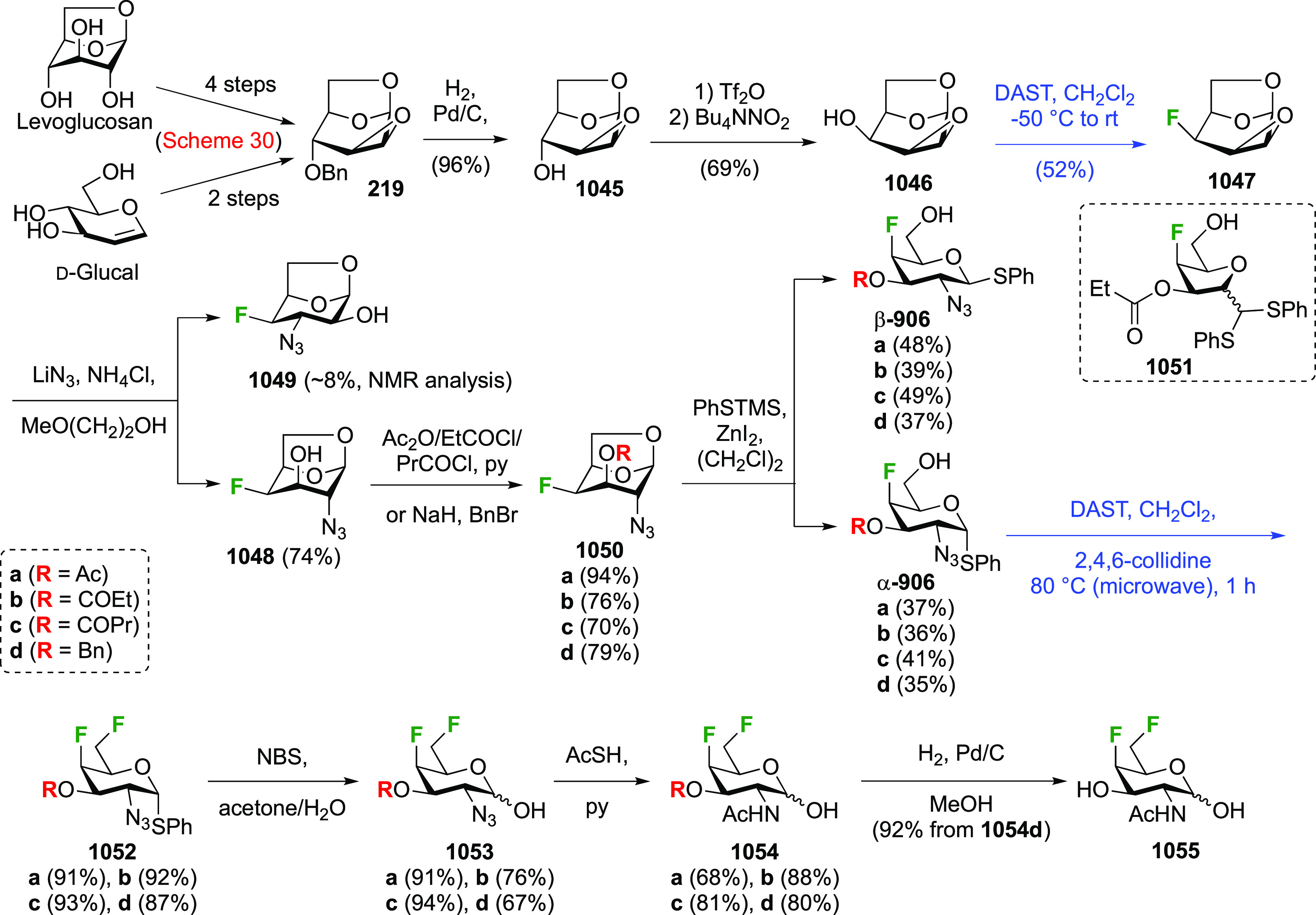
Sequential Fluorination Strategy via 1,6-Anhydro Derivatives to Access 4,6-Difluorinated GalNAc Derivatives^429,463^

The Karban group synthesized a series of 4,6-difluorinated GalNac derivatives using 1,6-anhydrosugar chemistry (Scheme 143). The benzyl group in the 1,6:2,3-dianhydro derivative **219**, obtained as described in Scheme 30, was hydrogenolyzed to give **1046**, which was deoxyfluorinated with retention of configuration to give **1047**.^146,451^ Epoxide opening with lithium azide led to **1048** in 74% yield with a small amount of regioisomer **1049** observed. The alcohol group in **1048** was now acylated to give the acetate **1050a**,^472^ the propionate **1050b**, and the butyrate **1050c**,^463^ as well as benzylated to give **1050d**.^464^ Anhydro-bridge cleavage was effected with PhSTMS to give a mixture of separable anomers **906a**–**d**.^463,464^ Interestingly, for the propionate derivative **1050b**, a rearrangement byproduct **1051** was also isolated. This is formed through activation of the azido group by ZnI_2_, initiating neighboring group participation from the endoxyclic O5 (not shown).^429^ As explained with Scheme 139, the thiophenyl anomers were separated to avoid dealing with possible 1 → 6 migration side reactions arising from the β-anomer.^430^ Hence, the α-anomers α-**906a**–**d** were subjected to the DAST-mediated deoxyfluorination conditions, followed by anomeric deprotection and azide reduction/acetylation to give the 4,6-difluorinated GalNAc derivatives **1054a**–**d** as analogues of the cytotoxic triacetylated GalNAc,^463^ and the benzyl ether **1054d**. This was then fully deprotected to give 2,4,6-trideoxy-2-acetamido-4,6-difluorogalactose **1055**.^429^

**Scheme 144:**
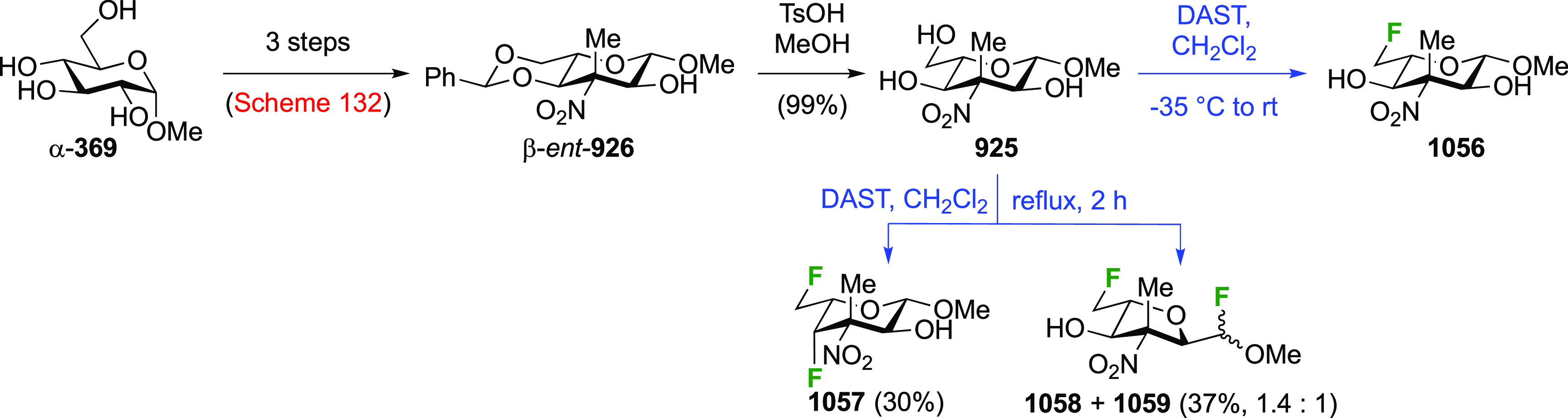
Investigations on the Direct DAST-Mediated Difluorination of a Branched Nitrosugar^299,443^

In Cabrera-Escribano’s investigations of fluorinations on branched nitrosugars (cf. Scheme 132), 4,6-dideoxy difluorination was also achieved from **925** (Scheme 144), which was obtained by benzylidene acetal deprotection of β-*ent*-**926**. Using the same DAST conditions as applied to **927**, only deoxyfluorination at C-6 took place, leading to **1056**.^442^ In refluxing solvent, however, deoxyfluorination at the 4- and 6-positions took place leading to **1057**,^443^ an outcome consistent with the Somawardhana result as described in Scheme 60A. However, ring contraction diastereomers **1058** and **1059** were also observed, with their structure reassigned in a later publication,^299^ via a similar process already shown in Scheme 43A. The isolation of **1058** and **1059** suggests that deoxyfluorination at C-4 does not precede ring contraction (cf. Scheme 43A).

##### 8.1.11.2 Glucose Stereochemistry

**Scheme 145:**
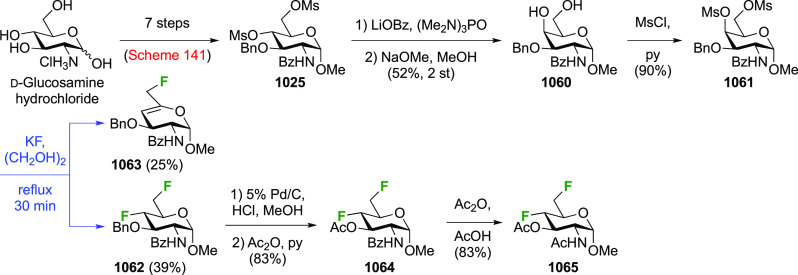
A Direct Synthesis of 4,6-Difluoro GlcNAc via Mesylate Displacement^467^

The Richardson group also reported the synthesis of 4,6-difluorinated GlcNAc derivatives using the dimesylate fluorination approach (Scheme 145).^467^ Hence, the advanced glucosamine intermediate **1025**, for which the synthesis is described in Scheme 141, was converted to its galactosamine analogue **1061** using nucleophilic substitution with lithium benzoate, followed by ester methanolysis (cf. Scheme 63 for another example of this approach) and mesylation. In contrast to the fluoride displacement of **1025** (cf. Scheme 141), reaction of **1061** with KF in refluxing ethylene glycol resulted in a significant amount of elimination product **1063**, which is due to the availability of an antiperiplanar C–H bond at the 5-position. Other fluorination conditions, such as lowering the temperature to 100 °C or using TBAF in refluxing acetonitrile, either failed to give product or returned a complex reaction mixture with **1062** formed in <40% yield. From **1062**, a protecting group change of OH-3 and conversion of the benzamide group to an acetamide afforded **1065**.

**Scheme 146:**
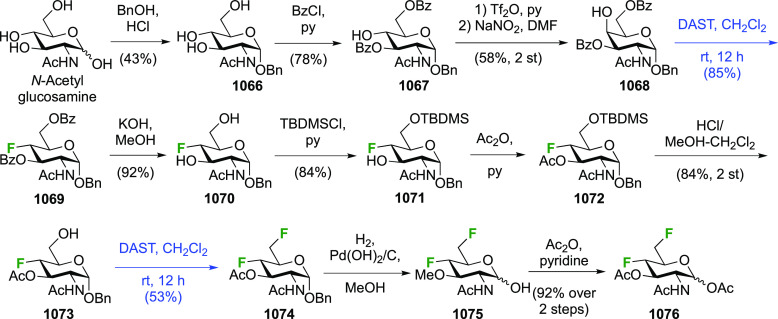
A Sequential Deoxyfluorination Approach to a 4,6-Difluorinated GlcNAc Derivative^473^

The Ling group has synthesized the peracetylated 4,6-dideoxy-4,6-difluoro glucosamine **1076** (Scheme 146) using a sequential deoxyfluorination approach.^473^*N*-Acetyl glucosamine was converted to its α-benzyl anomer **1066**,^474^ then subjected to benzoylation conditions which were selective for the 3- and 6-positions.^475,476^ Inversion of the OH-4 group in the resulting **1067** was achieved by a Lattrell-Dax reaction to give **1068**, followed by deoxyfluorination to give **1069**.^476^ Benzoate methanolysis to **1070**(476) was followed by a protecting group sequence to arrive at the free OH-6 in **1073**, which was subjected to another deoxyfluorination to give **1074**. Anomeric deprotection followed by acetylation then gave **1076**.^473^

**Scheme 147:**
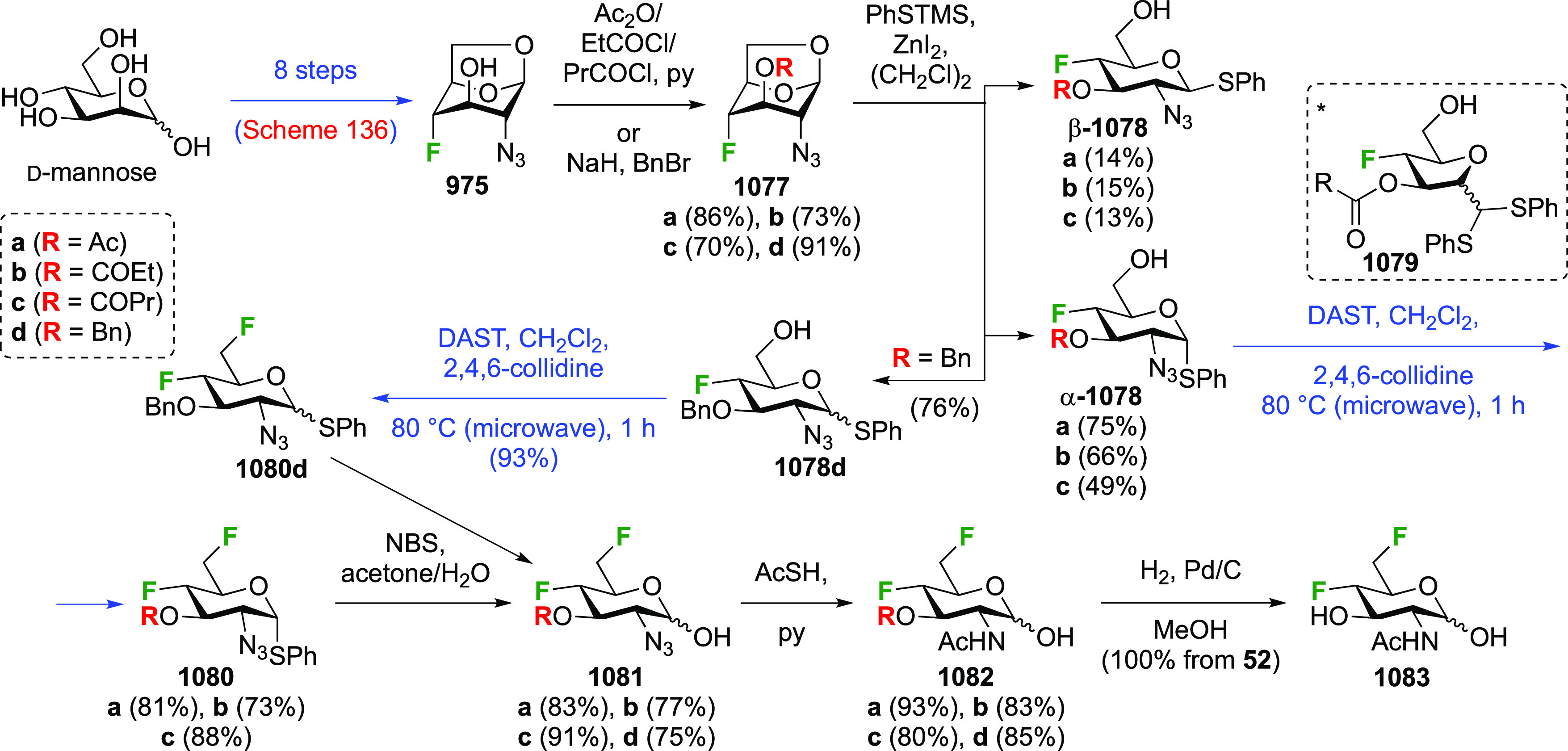
A Sequential Deoxyfluorination Approach to 4,6-Difluorinated GlcNAc Using 1,6-Anhydropyranose Chemistry^429,463^

The Karban group also used a sequential deoxyfluorination approach to obtain 4,6-difluorinated GlcNAc derivatives based on 1,6-anhydrosugar chemistry (Scheme 147).^429,463^ The key intermediate **975**, for which the synthesis was discussed in Scheme 136, was converted to the acetate **1077a**,^462^ the propionate **1077b**, the butyrate **1077c**,^463^ and the benzyl ether **1077d**.^464^ Anhydro-bridge opening of **1077a**–**c** using PhSTMS was followed by separation to obtain the pure anomers, with the α-anomer now clearly the major product,^462,463^ in contrast to the result obtained with the corresponding galacto-configured derivatives (see Scheme 143). In some cases, the anomers were contaminated by ring contraction products **1079**. The opening of the benzyl ether **1077d** led to **1078d**, the anomers of which were not separated.^429^ Fluorination at OH-6 was carried out with α-**1078a**–**c**, as discussed above to avoid complications with a possible 1 → 6 migration with the β-anomers (cf. Schemes 128 and 129), to give the 4,6-difluorinated GlcNAc derivatives **1080a**–**c** in high yield.^463^ However, when the deoxyfluorination was carried out on the mixture of benzyl anomers **1078d**, an excellent yield of **1080d** was obtained with no mention of migration issues.^429^ This is consistent, however, with the result shown in Scheme 128C in which there was a low level of migration product with the glucose-based substrate. Treatment of the thus obtained 4,6-difluorinated derivatives **1080** with NBS in aqueous medium gave the free hemiacetals **1081a**–**d**, whereupon the azide group was reduced with concomitant acetylation to give **1082a**–**d**.^429,463^ The benzyl ether was then removed via hydrogenolysis to give 4,6-dideoxy-4,6-difluoroGlcNAc **1083**.^429^

### 8.2 Fluorination at Three Positions

#### 8.2.1 Fluorination at Positions 2,3,4

**Scheme 148:**
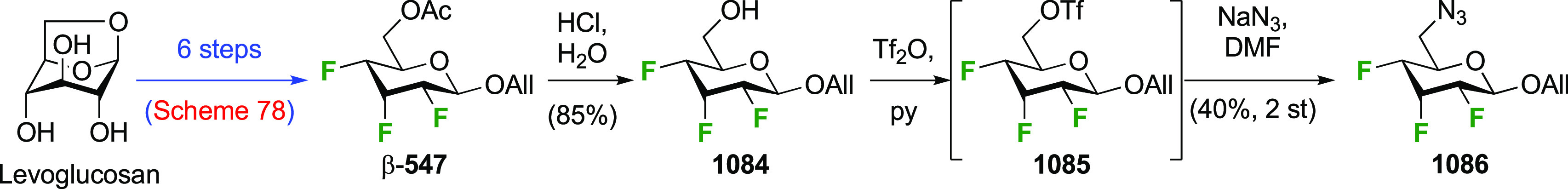
Synthesis of a 6-Azido 2,3,4-Trifluorinated Alloside Derivative^295^

The Giguère group reported the synthesis of the 6-azido 2,3,4-trifluorinated allose derivative **1086** (Scheme 148) from advanced intermediate β-**547**, for which the synthesis was described in Scheme 78.^295^ Acetate hydrolysis followed by triflate activation and azide substitution led to **1086**, which was successfully used in a click reaction with a dipeptide derivative (not shown).

#### 8.2.2 Fluorination at Positions 3,4,6

**Scheme 149:**
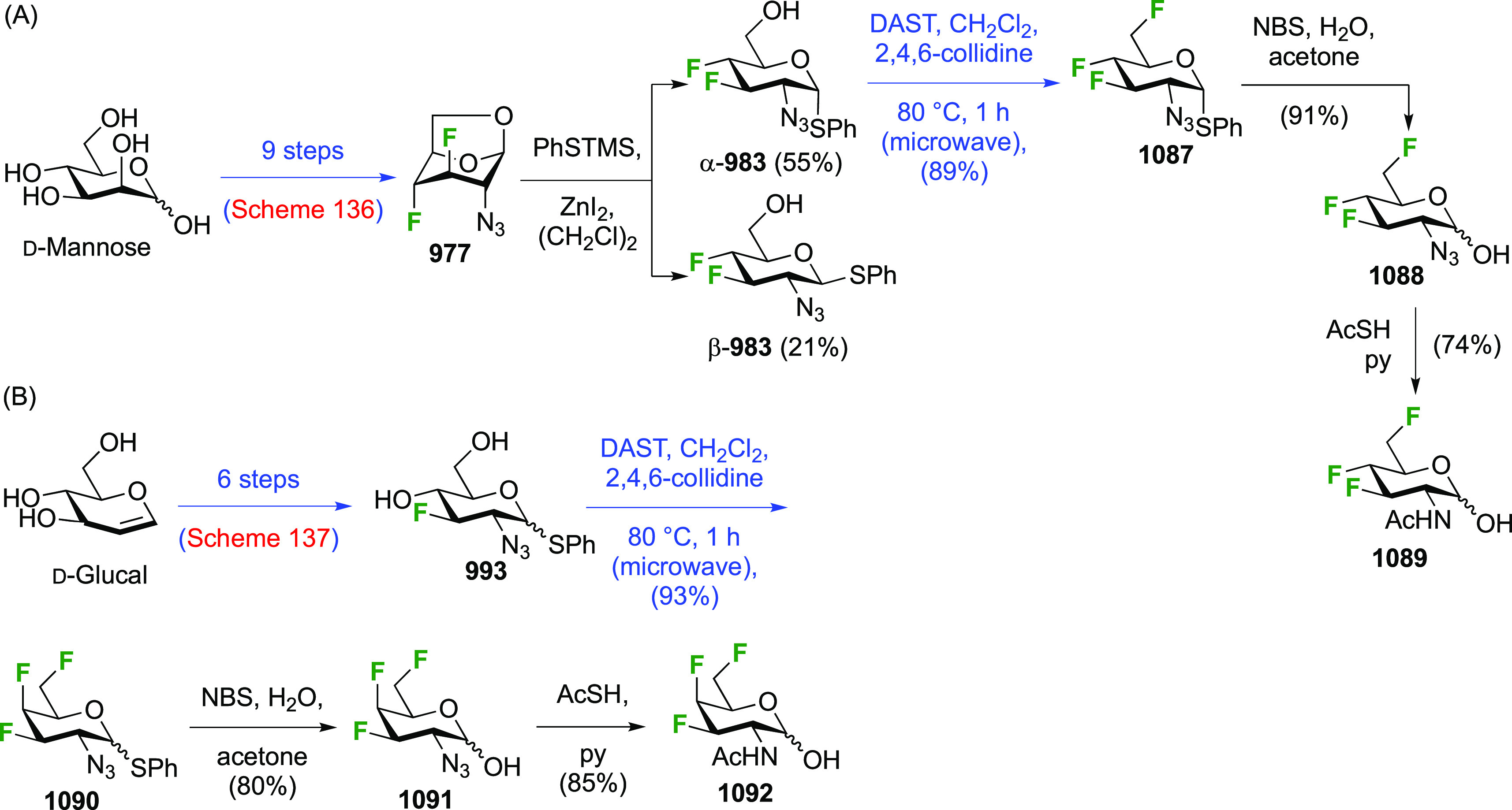
Synthesis of 3,4,6-Trifluorinated GlcNAc and GalNAc^429^

The Karban group disclosed the synthesis of 3,4,6-trifluorinated GlcNAc and GalNAc **1089** and **1092**.^429^ Advanced intermediate **977** (Scheme 149A), for which the synthesis was discussed in Scheme 136, was treated with PhSTMS to achieve anhydro-bridge opening with the formation of separable thioglycosides. The α-anomer of **983** was reacted with DAST to effect OH-6 deoxyfluorination, giving **1087**. After anomeric deprotection and azide reduction/acetylation, this gave the trifluorinated GlcNAc **1089**.

Subjection of intermediate **993**, obtained as discussed in Scheme 137, to deoxyfluorination conditions led to **1090** in excellent yield (Scheme 149B). Anomeric deprotection and azide conversion then gave the trifluorinated GlcNAc **1092**.

## 9 Sialic Acids

Fluorinated sialic acids are being extensively explored and will be discussed in this section. In particular, 2,3-difluorinated sialic acids have been widely investigated as mechanism-based inhibitors. Their synthesis is possible via direct vicinal difluoride introduction (cf. sections 3.1.1, 6.1, and 8.1.1), although hazardous reagents are required. Therefore, most syntheses of 2,3-difluorinated sialic acid analogues thus adopt a sequential approach which in all cases involves obtaining C-3-fluorinated sialic acid first, followed by anomeric fluorination. Because of the number of analogues reported, often using different methods for the first and second fluorination, these fluorination steps will be discussed separately. Selected examples from the literature that feature a single F-3 introduction are also included for discussion purposes.

### 9.1 Nomenclature and Assignment

#### 9.1.1 Nomenclature

**Figure 5:**
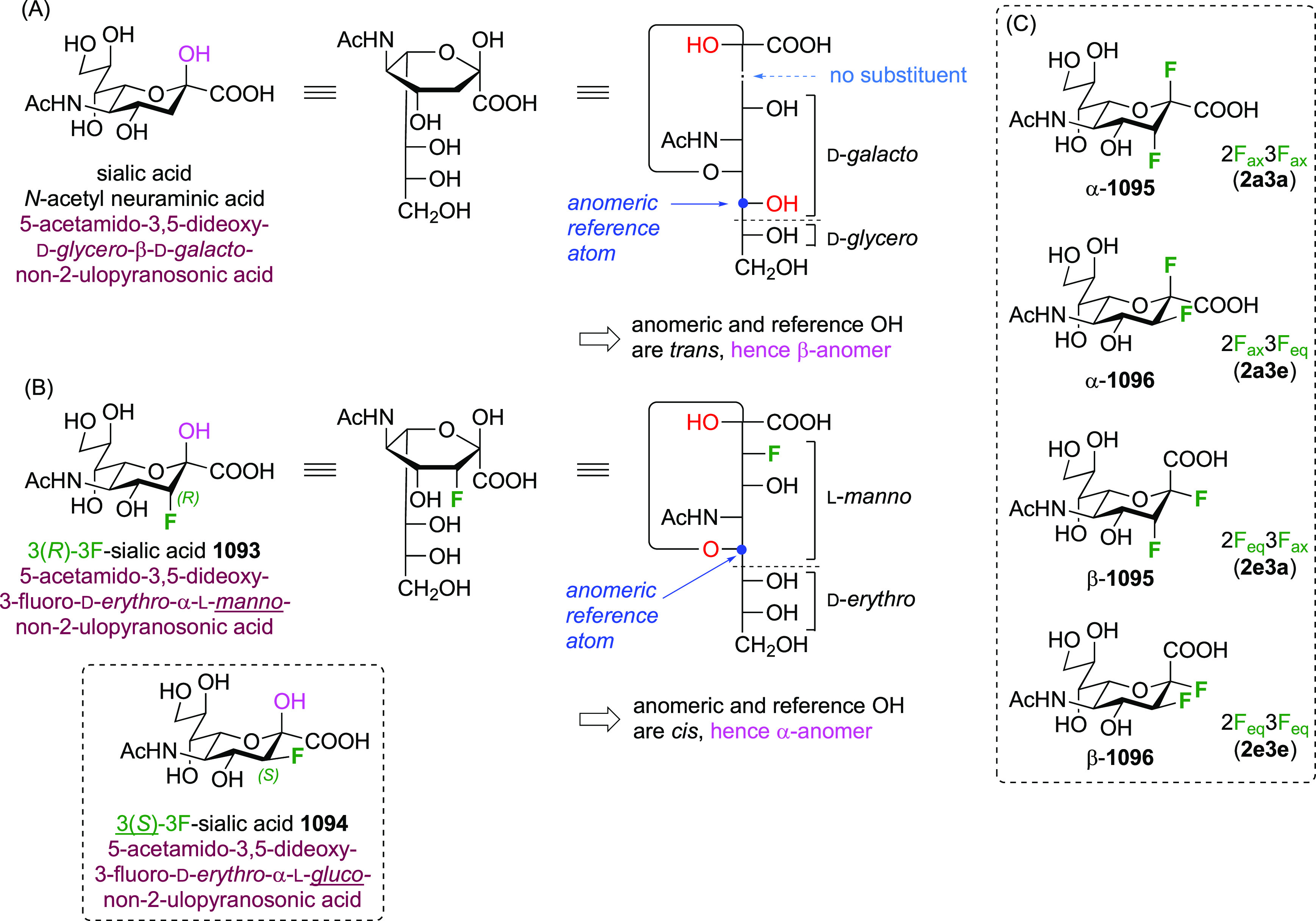
Nomenclature for sialic acid and 3-substituted sialic acids.

The nomenclature of sialic acid and its derivatives is complex and confusing, and errors can be found in the literature. Hence, this section is included in order to ensure consistent naming of derivatives. Sialic acid, or *N*-acetyl neuraminic acid (Figure 5A), is a non-2-ulonic acid derivative, or non-2-ulopyranosonic acid if the ring structure is included in the name. It has no substituent at the 3-position. For its IUPAC systematic name,^477^ the Fisher structure is considered (Figure 5). As there are more than four chiral centers, two configurational prefixes are required for the stem name. For sialic acid, these are d-*galacto* and d-*glycero*. However, when a single fluorine (or any other substituent) is introduced at C-3, as in **1093**, a new chiral center is created. Consequently, this results in different configurational prefixes. For **1093**, with (3*R*)-configuration, this is l-*manno* and d-*erythro*. For the other C-3-diastereomer **1094**, this is l-*gluco* and d-*erythro*.

The α,β-assignment of the anomeric center is nontrivial, and furthermore changes upon introduction of a (single) substituent at C-3. Consideration of the so-called “anomeric reference atom” is required. This is the highest numbered carbon atom of the group of stereocenters within the configurational prefix alongside the anomeric center, which is also involved in the heterocyclic ring. For sialic acid and **1093**, the configurational prefix encompasses four stereocenters, but in sialic acid the 3-position is ignored as it is not a chiral center. Hence, the different atoms of the configurational prefixes involved in the ring structures of sialic acid and **1093** result in a different anomeric reference atom. These are indicated in Figure 5 with a blue dot. For sialic acid, the substituents at the anomeric center and on the reference atom are *trans*, hence the anomer shown is the β-anomer. For **1093**, the two substituents are *cis*, thus this is the α-anomer. Hence, while both sialic acid and **1093** have the anomeric OH group in the same axial position, their anomeric assignment is different.

In this section, this IUPAC nomenclature will be used, even if that differs from the nomenclature used in the referenced publication.

CIP nomenclature can also be used to indicate F-3 configuration (e.g., 3(*R*) in **1093**), but this is cumbersome and not “at-a-glance”. Together with the difference in anomeric assignment compared to sialic acid, a convenient system just indicating the orientation of the fluorines on a chair conformation as axial or equatorial (e.g., 2F_ax_3F_ax_, Figure 5C), is sometimes used in the literature as well. This will also be adopted here for the ease of discussion.

#### 9.1.2 Identification

Given the occasional nomenclature errors in literature experimental sections, it is useful to include a section regarding anomeric assignment of 2,3-fluorinated sialic acids at C-2 and C-3. This is possible using NMR analysis; relevant data are shown in Table 1 for the four possible 2,3-difluorinated sialic acid compounds α/β-**1095**/**1096**.^478,479^ The configuration of F-3 is easily determined through the magnitude of the vicinal coupling between H-3 and H4: with F_eq_-3 this is a *trans*-diaxial coupling with H-4 and hence ^3^*J*_H3–H4_ will be around 8–10 Hz, while with F_ax_-3 ^3^*J*_H3–H4_ will be much smaller. Furthermore, the same is true for the vicinal F3–H-4 coupling: with F_ax_-3 this is a *trans*-diaxial coupling and ^3^*J*_F3–H4_ will be between 25 and 30 Hz, while with F_eq_-3 ^3^*J*_H4–F3_ will be 15 Hz or smaller (Table 1).

**Table 1:**
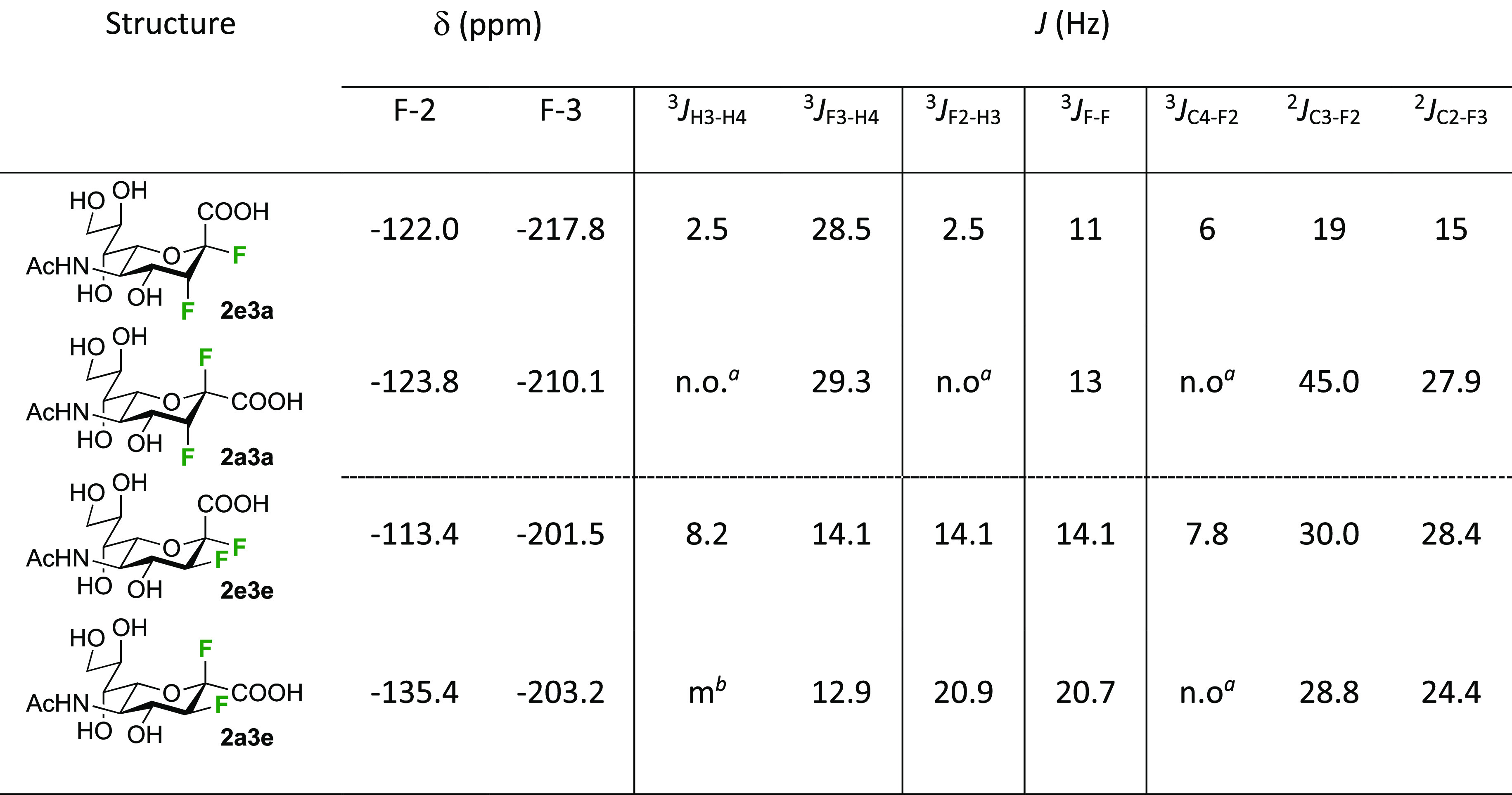
Diagnostic NMR Features of 2,3-Difluorinated Sialic Acid Derivatives. Data Taken from References (478 and 479)

a Not observed.

b H-3 and H-4 are multiplets.

Determination of the configuration of F-2 is only straightforward in the presence of an F_eq_-3 substituent, as only then are the ^3^*J*_F2–H3_ coupling constants of diagnostic value. This coupling constant is 21 Hz for **2a3e** and 14 Hz for **2e3e** (Table 1). While H3 or H-4 can be part of a multiplet, these values are typically easily extracted from the ^19^F NMR spectrum. Unfortunately, ^19^F–^19^F coupling constants do not adhere to the Karplus rule, although they do have diagnostic value to distinguish 2,3-difluorinated sialic acid derivatives: the largest (absolute) value is found for the F2_ax_F3_eq_ derivative (21 Hz), which is well above the other values.

For unambiguous assignment vicinal ^13^C–^19^F coupling constants need to be considered, which do adhere to the Karplus rule. Hence, ^3^*J*_C–F_ values will be higher for equatorial fluorines compared to their axial counterparts. With F_eq_-2 ^3^*J*_C4–F2_ values are 6–8 Hz, while with F_ax_-2 this coupling is not observed, although a value of <2 Hz is expected (Table 1). The same is seen for the ^3^*J*_C5–F3_ values, which are ∼8 Hz when F-3 is equatorial and <3 Hz when axial (not shown). Interestingly, a geminal C–F coupling can have diagnostic value as well: Wray noted that ^2^*J*_C–F_ increases with the change of an electronegative substituent bonded to the coupled carbon from a *gauche* to a *trans*-orientation with respect to the fluorine involved in the coupling.^53^ With 2,3-difluorinated sialic acids, this is especially useful for ^2^*J*_C3–F2_ (Table 1): a value of 45 Hz is found for the *trans*-diaxial F2_ax_F3_ax_ compound, whereas it has a much lower value when the fluorines are *gauche* in **2e3a**. The Wray-rule is not useful for ^2^*J*_C2–F3_, presumably as there are two extra electronegative groups at C-2, but, given the stereochemistry of F-3 is easily established otherwise, this is not an issue.

Given that in most cases F-2 is introduced after installation of F-3 with known configuration, anomeric assignment is required. A summary of diagnostic coupling constants to easily achieve this is provided inFigure 6.

**Figure 6:**
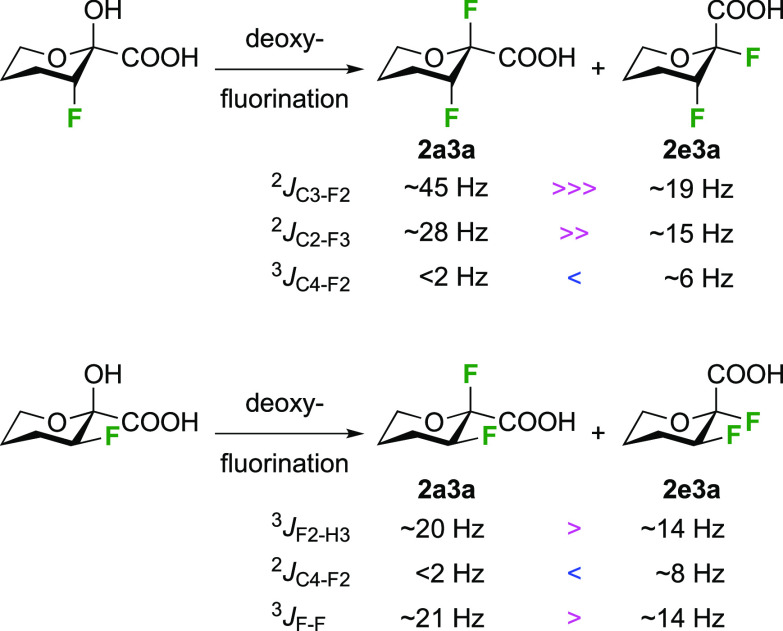
Diagnostic NMR values to assign anomeric configuration.

### 9.2 Simultaneous Fluorine Introduction at C-2 and C-3

**Scheme 150:**
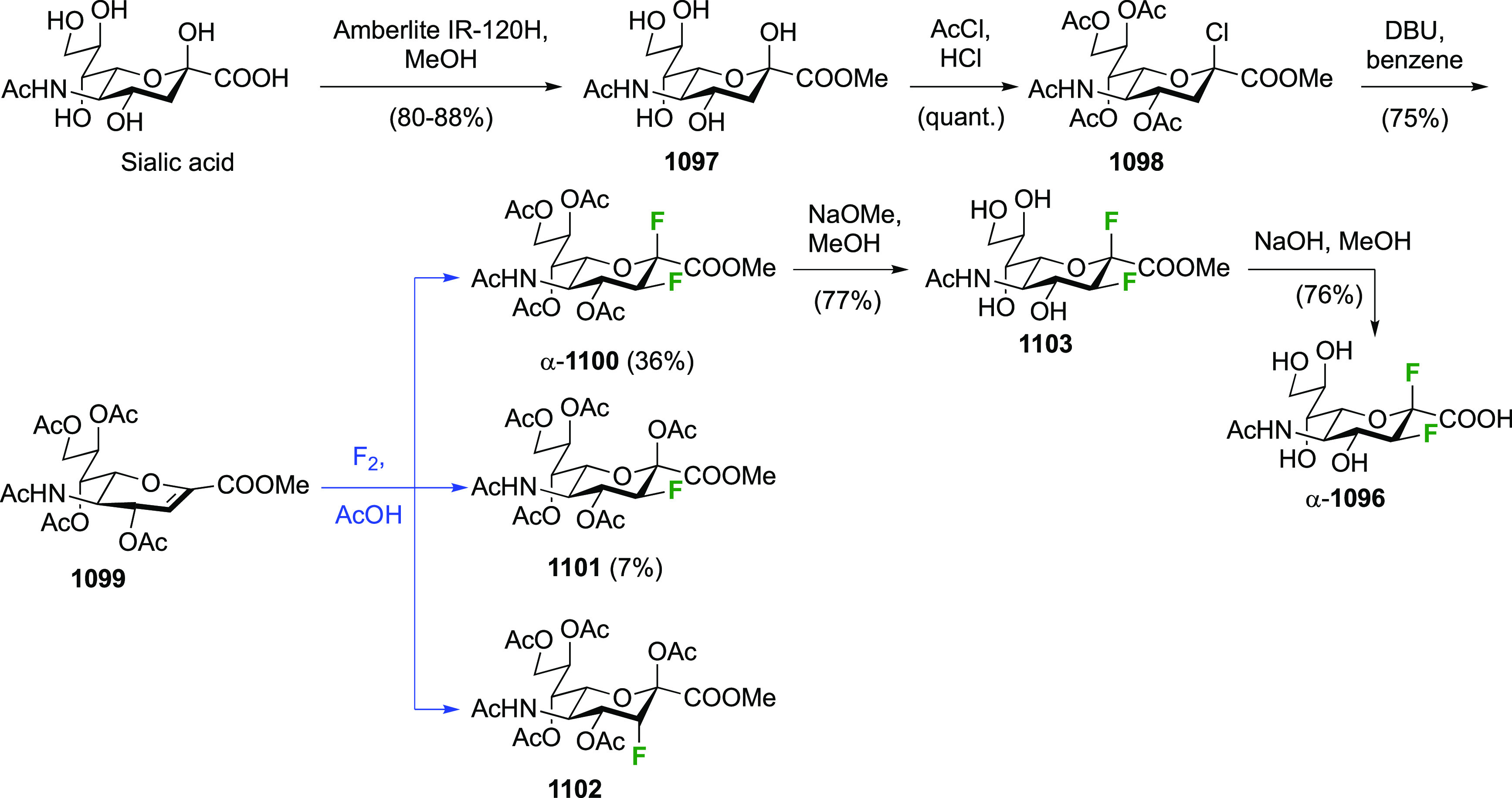
Direct Difluorination Approach with F_2_ to Give the 2F_ax_3F_eq_ Adduct^482^

The earliest syntheses of 2,3-difluorinated sialic acid analogues employed electrophilic fluorination of the sialic acid glycal intermediate **1099** (Scheme 150), which can be obtained from sialic acid by methyl ester formation to **1097**, acetylation of the alcohol groups with concomitant conversion of the hemiketal to the chloride **1098**, and finally by elimination of the latter to the conjugated ester.^479−481^ Reaction of **1099** with fluorine gas in acetic acid gave the 2F_ax_3F_eq_-difluorinated sialic acid derivative α-**1100** as the major product in 36% yield, alongside the 2-acetoxy-3-fluoro diastereomers **1101** and **1102** as side products.^482^ With AcOF as the fluorinating agent, **1101** was the major product and α-**1100** and **1102** the minor products (34%, 7.7%, 0.5% yields, respectively, not shown). Acetate methanolysis and acid hydrolysis of α-**1100** gave α-**1096**, which was reported to be a potent inhibitor against neuraminidase.^482^

**Scheme 151:**
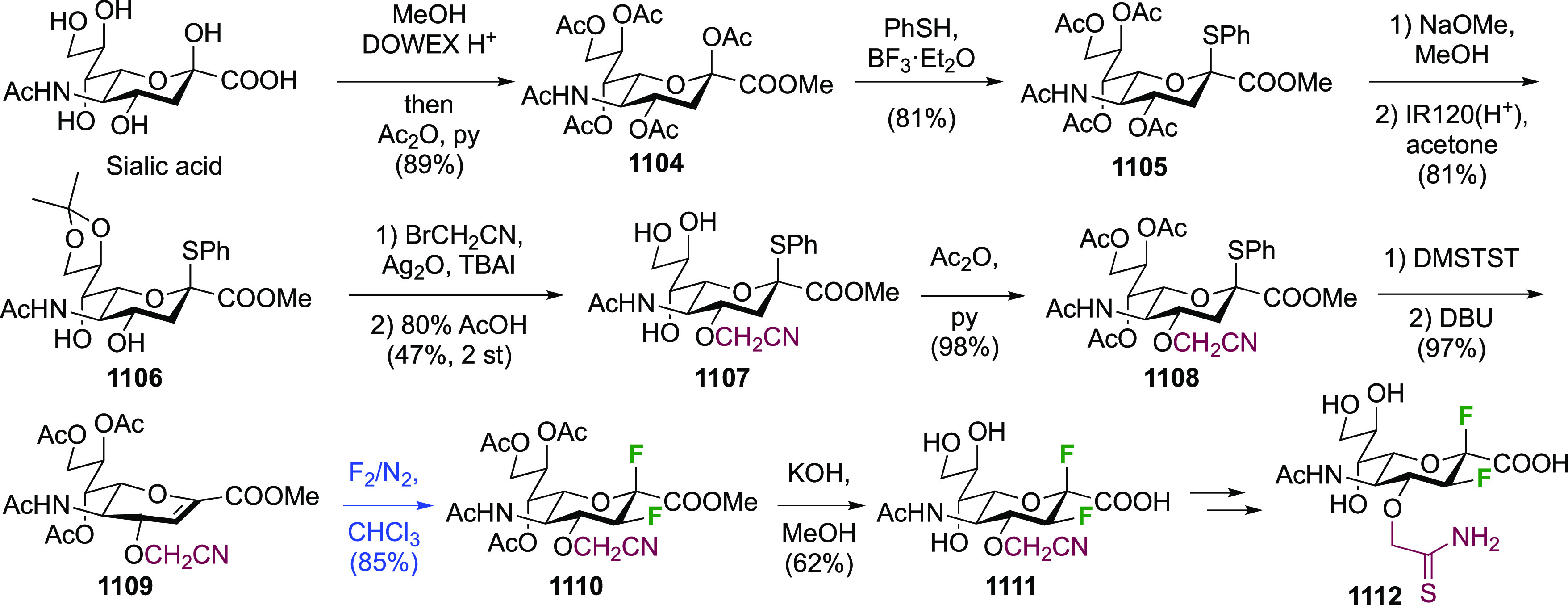
Synthesis of a 2,3-Difluorinated Sialic Acid Analogue Using a Direct Difluorination Strategy with F_2_^483^

This methodology was used by the Ikeda/Sato group on a sialic acid glycal modified at the 4-position (Scheme 151).^483^ Starting from peracetylated sialic acid methyl ester **1104**,^484^ which could be obtained from sialic acid without using diazomethane via acid-catalyzed methyl ester formation followed by alcohol acetylation,^485^ protection of the anomeric center as the thiophenolate resulted in the formation of **1105**.^486^ Deacetylation was followed by acetonide formation, which was selective for the terminal side-chain position. The resulting **1106** was selectively alkylated at OH-4, upon which the acetonide was hydrolyzed and all alcohols reprotected as acetates to give **1108**. Activation of the anomeric substituent with dimethyl(methylthio)sulfonium triflate (DMTST) allowed its elimination with DBU to give the key glycal intermediate **1109**.^487^ Reaction with diluted fluorine gas was reported to lead to **1110** stereoselectively, which after deprotections gave the 2F_ax_3F_eq_-difluorosialic acid derivative **1111**. This was further converted to the human sialidase inhibitor 5-acetamido-3-cyanomethyl-2,5-dideoxy-2,3-difluoro-α-d-*erythro*-l-*gluco*-2-nonulopyranosonic acid **1112**.^483^

**Scheme 152:**
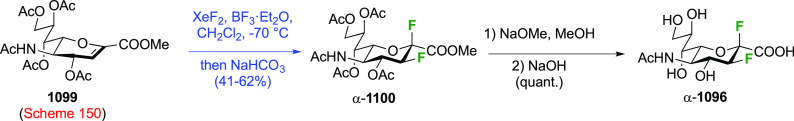
Direct Difluorination Strategy with XeF_2_^479,481^

Alternatively, reaction of glycal **1099** (synthesis described in Scheme 150) with xenon difluoride/BF_3_ also leads to *syn*-vicinal difluorination with the same facial selectivity as the reaction with F_2_, with no report of formation of F_ax_-3-containing minor products (Scheme 152).^479,481^ Acetate methanolysis and methyl ester hydrolysis then gave α-**1096**.

### 9.3 Stepwise Introduction

#### 9.3.1 Fluorination at C-3 from the Glycal

**Scheme 153:**
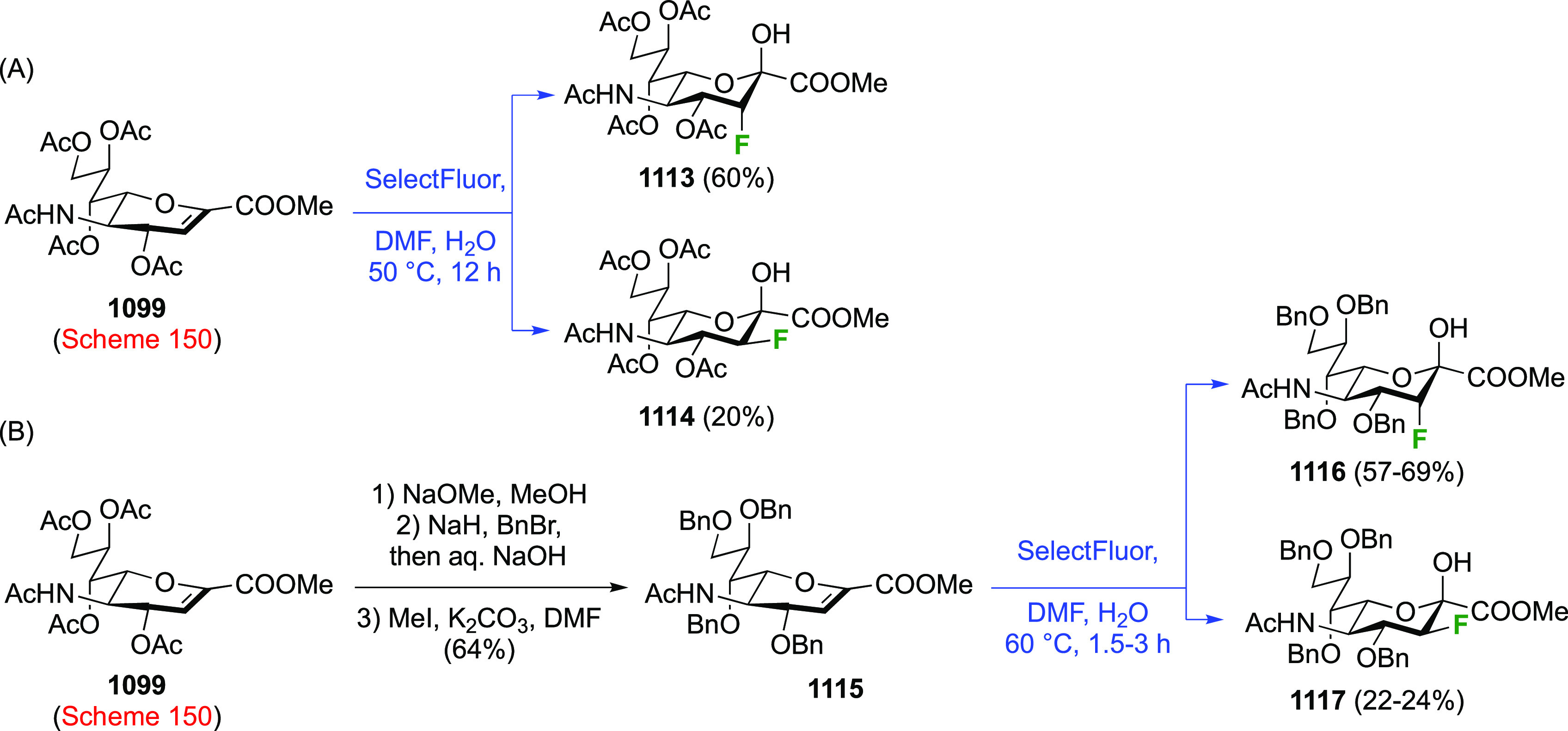
Fluorine Introduction at C-3 via Reaction of Sialic Acid Glycal with SelectFluor^170,488,490,491^

The reaction of the sialic acid glycal with SelectFluor was first described by the Wong group (Scheme 153A).^170,488^ From the peracetylated glycal **1099**, the F_ax_-3 and F_eq_-3 diastereomers **1113** and **1114** were obtained in a 3:1 ratio (isolated yields) in excellent overall yield. The Ito/Kanie group reported that the reaction on the perbenzylated derivative **1115**, obtained via a protecting group switch from **1099** (Scheme 153B),^489^ was much faster and with similar yields of the F_ax_-3 and F_eq_-3 stereomers compared to the reaction of **1099**, albeit with a slightly lower diastereoselectivity.^490^ This result has been confirmed by the Gilmour group.^491^ This group also updated the original synthesis of **1115**(489) by replacing diazomethane with iodomethane.^491^

**Scheme 154:**
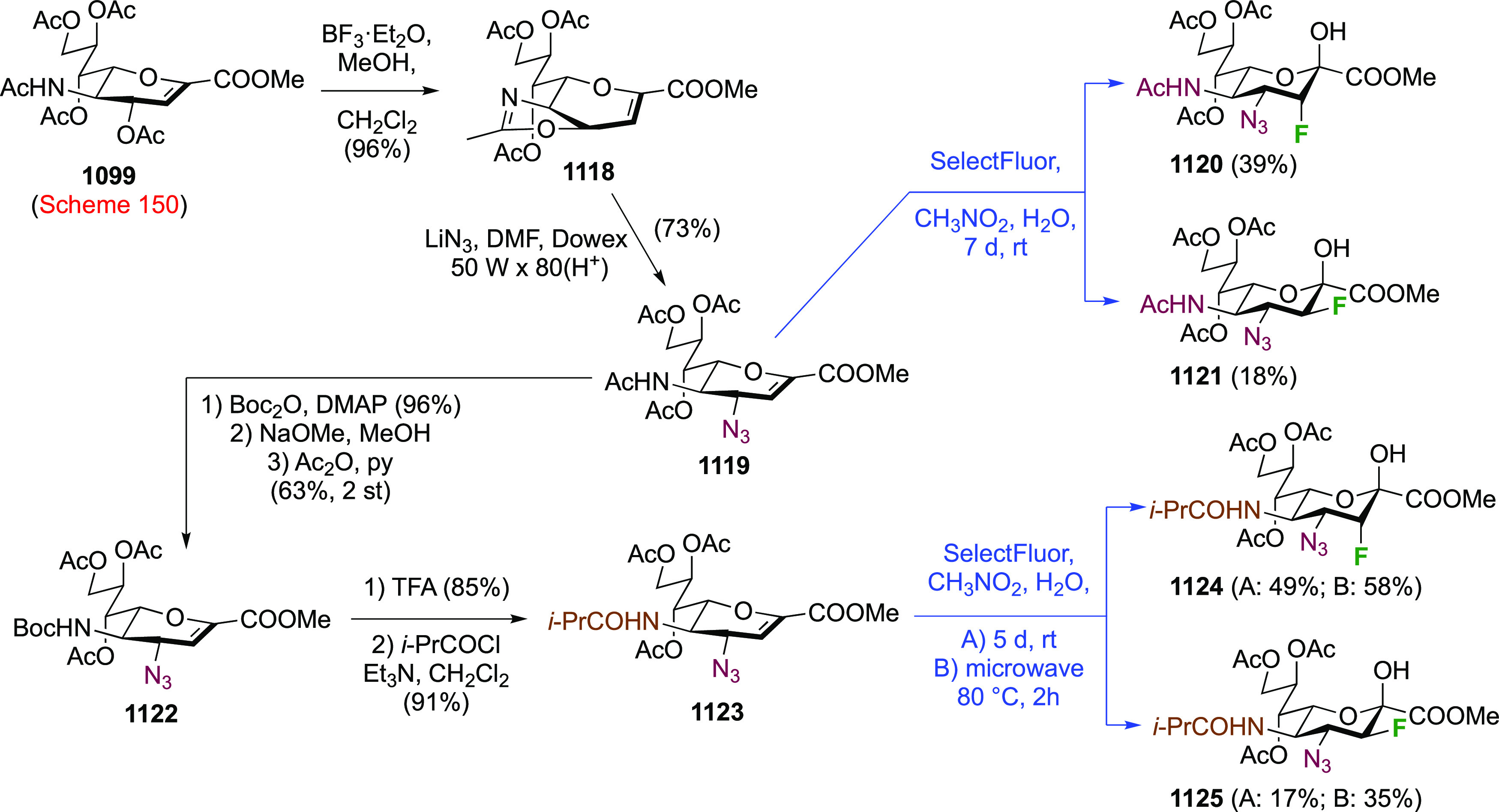
Fluorine Introduction at C-3 via Reaction of 4-Azido Modified Sialic Acid Glycal with SelectFluor^493,494^

This fluorination methodology has also been employed for 4-azido analogues, such as **1119** and **1123** (Scheme 154). The former can be obtained by Lewis acid activation of the allylic acetate in **1099**, which initiates cyclization of the NAc group to form a fused oxazoline, giving **1118**. Reaction with azide under acid activation at the allylic position provided the 4-azido group in **1119** with overall retention of configuration.^492^ Subsequent treatment of **1119** with SelectFluor in a nitromethane–water mixture at room temperature gave the F_ax_-3 derivative **1120** as the major isomer in 39% yield, alongside the F_eq_-3 **1121** in 18% yield (2.2:1 ratio).^493^ With the similar glycal **1123**, the von Itzstein group obtained similar product yields (49% for **1124**, 17% for **1125**) using these room temperature conditions.^494^ The long reaction time confirms the unreactive nature of the acetylated glycal. Von Itzstein showed that reaction with SelectFluor at 80 °C under microwave irradiation dramatically decreased the reaction time, while increasing the product yield and ratio. In 2 h, **1124** and **1125** were obtained in a 1.7:1 ratio in 93% combined yield (58% for **1124**, 35% for **1125**).^494^ Glycal **1123** can be obtained from **1119** by switching the *N*-acetyl for a Boc group, which proceeds first by Boc-protection of the amide and acetamide hydrolysis, followed by the required reacetylation of the alcohol groups to get **1122**. Amine deprotection followed by acylation with isobutanoyl chloride then provides **1123**.^495,496^

**Scheme 155:**
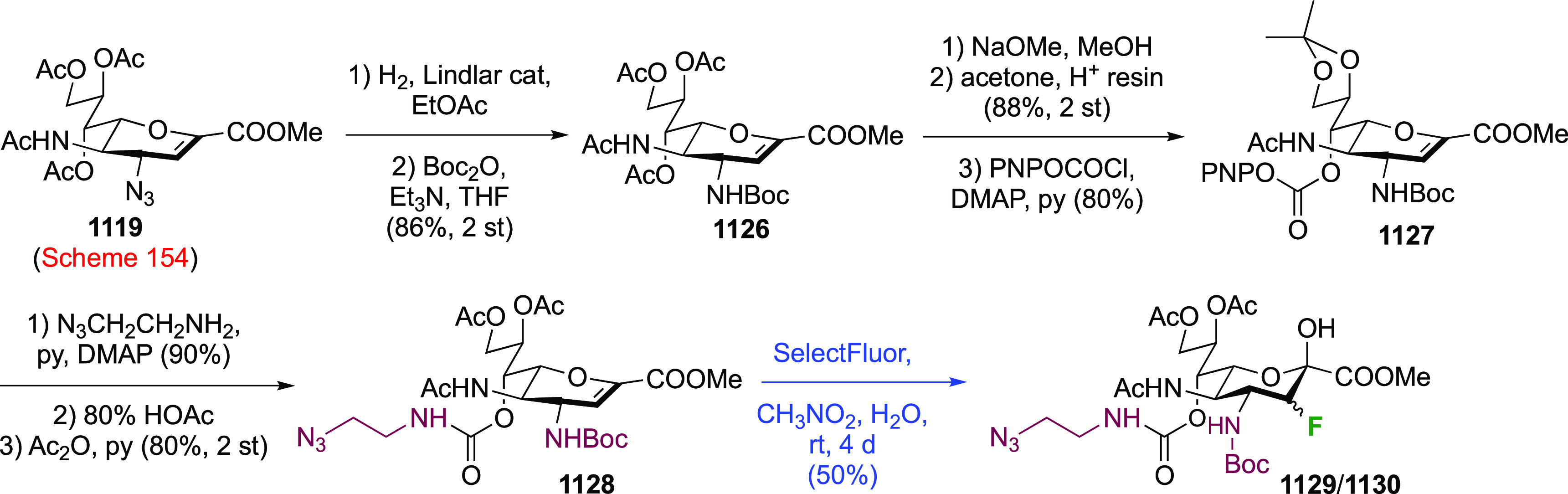
Fluorine Introduction at C-3 via Reaction of 4-Boc-amino Modified Sialic Acid Glycal with SelectFluor^497^

With a less electron withdrawing NHBoc group at the 4-position, as in glycal **1128** (Scheme 155), the SelectFluor reaction required 4 days at room temperature to give a 50% combined yield of **1129**/**1130**.^497^ Unfortunately, no ratio was reported. The glycal was obtained from **1119** by azide reduction and subsequent Boc protection to give **1126**, upon which the remaining alcohols were deprotected. After terminal acetonide formation, the OH-7 was activated to give the *p*-nitrophenyl (PNP) carbonate **1127**.^498^ Reaction with 1-amino-2-azidoethane gave the corresponding carbamate, after which the acetonide protecting group was removed and the alcohols reprotected as acetates, giving **1128**. The mixture of **1129**/**1130** was taken forward for deoxyfluorination (see Scheme 170).

#### 9.3.2 Aldolase Reaction with 3-Fluoropyruvate

##### 9.3.2.1 Unmodified ManNAc Starting Material

**Scheme 156:**
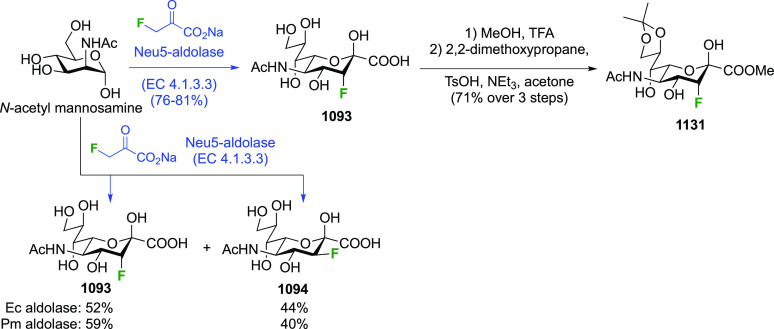
Enzyme-Catalyzed Aldol Reaction Leading to 3-Fluorinated Sialic Acid^411,413,478,499,502^

Sialic acid is biosynthesized by an aldol reaction between *N*-acetyl mannosamine (ManNAc) and sodium pyruvate, which is catalyzed by *N*-acetylneuraminic acid aldolase (EC 4.1.3.3). Following the patent literature, the Withers group reported that this enzyme effectively catalyzed the aldol reaction with monofluorinated pyruvate to give **1093** (Scheme 156).^478^ Only the formation of the F_ax_-3 stereomer was reported, in excellent yield (76%). The Bennett group confirmed this result (81% yield),^411^ as did the Watts group when they isolated the protected F_ax_ sialic acid derivative **1131**.^499^ However, using the same enzyme cloned from Ecoli K12,^500^ the Chen group obtained both F-3 diastereomers **1093** and **1094**, with the F_ax_-3 as the major product in a 1.2:1 ratio of isolated yields.^413^ A similar result was found using aldolase from *Pasteurella multocida*.^501−503^

##### 9.3.2.2 Modified ManNAc Starting Materials

**Scheme 157:**
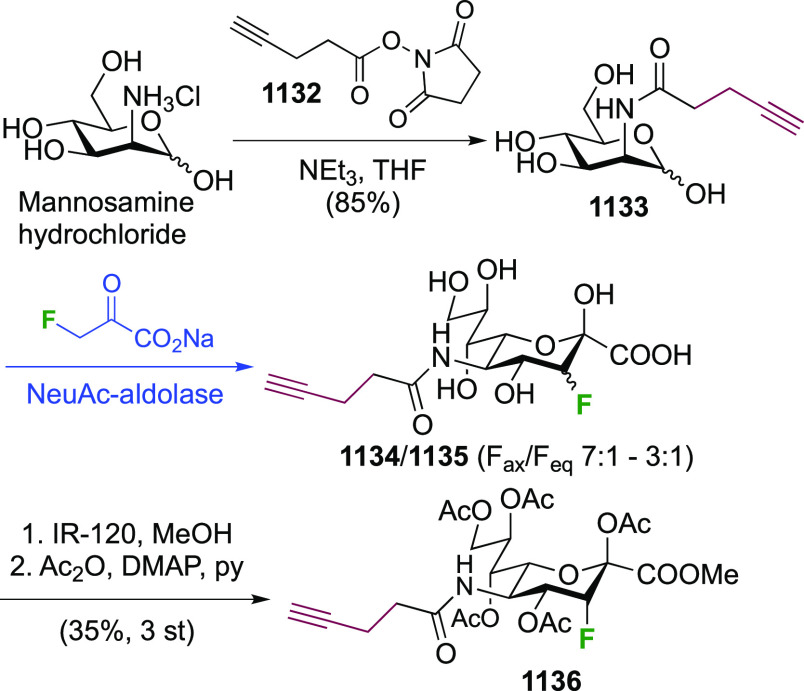
Aldolase Reaction on a Substituted ManNAc Substrate^505^

The aldolase enzyme also tolerates the use of modified ManNAc substrates, which has been exploited to produce azide and alkyne containing sialic acid derivatives for bioconjugation purposes. Starting from *N*-(pent-4-ynoyl)-mannosamine **1133** (Scheme 157), synthesized from mannosamine hydrochloride with activated pent-4-ynoic acid **1132**,^504^ the aldolase reaction with fluoropyruvic acid was reported by the Wong group to give a mixture of C-3 diastereomers **1134** and **1135** (F_ax_/F_eq_ 7:1–3:1).^505^ Purification by chromatography was possible after esterification and acetylation to afford **1136** in 35% yield over three steps.

**Scheme 158:**
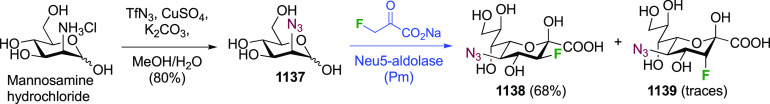
Aldolase Reaction on 2-Deoxy-2-azido Mannose^502^

The aldolase reaction with fluoropyruvate also proceeds when the *N*-acetyl group in ManNAc is modified to an azido group, as in **1137** (Scheme 158), which can be achieved from mannosamine by a diazo transfer reaction.^506^ The Chen group reported that *Pm* aldolase was efficient in catalyzing this reaction to give the F_eq_-3 product **1138** in 68% yield after chromatography.^502^ The F_ax_-3 product **1139** was formed as observed by TLC-analysis, but the yield was low and no product was isolated. The Ec aldolase was reported not to work efficiently with this substrate.

**Scheme 159:**
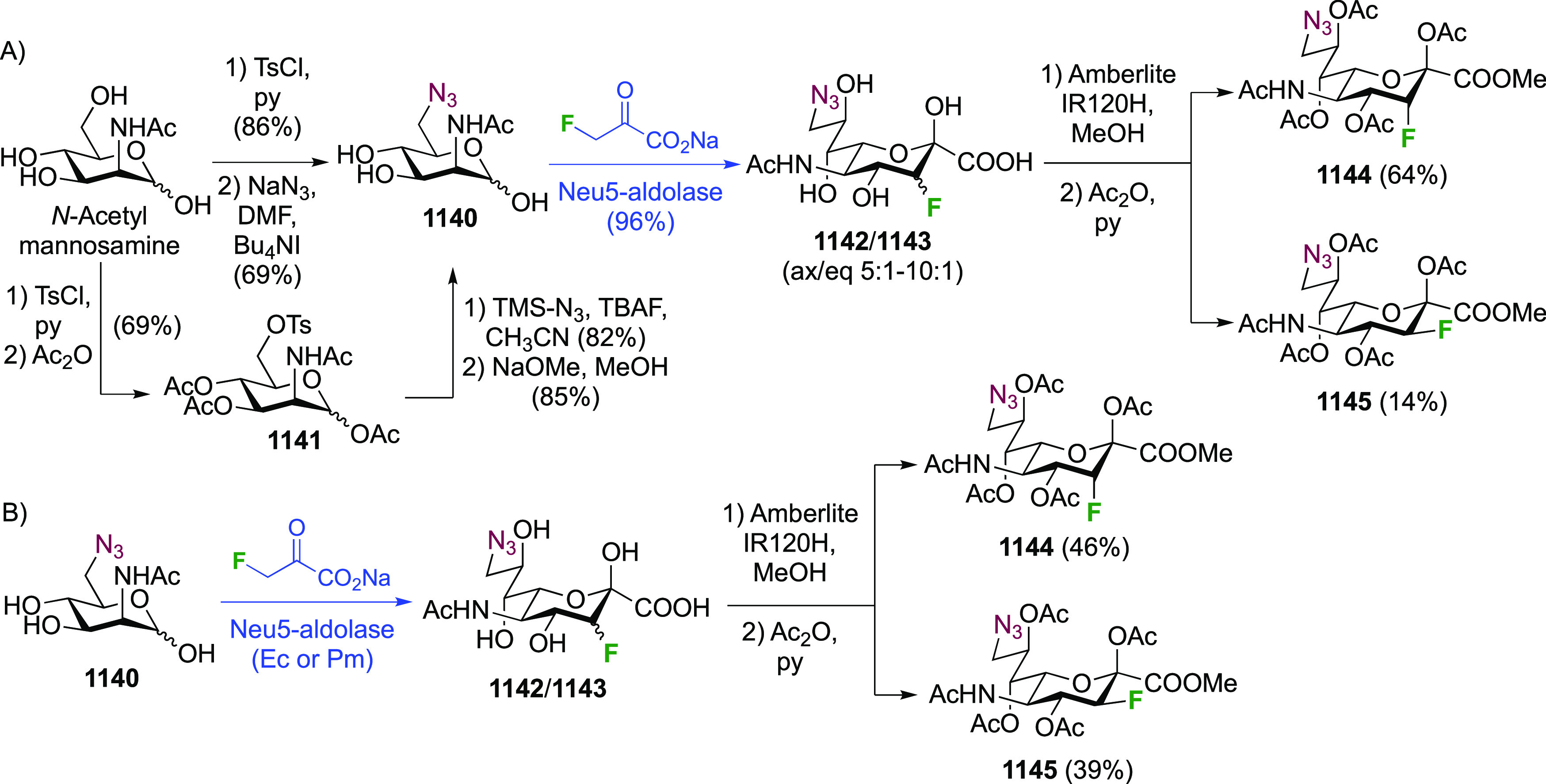
Aldolase Reaction on 6-Deoxy-6-azido ManNac^502,508^

The 6-deoxy-6-azido ManNAc substrate **1140** (Scheme 159A) can be obtained from ManNAc either in two steps, involving selective OH-6 tosylation and displacement with sodium azide,^503,507^ or in four steps when alcohol protection is included (via **1141**).^508^ The Withers group reported that aldolase reaction of **1140** with fluoropyruvate gave, after ester formation and alcohol acetylation, the F_ax_-3 isomer **1144** in 64% isolated yield and the F_eq_-3 isomer **1145** in 14% yield (4.6:1 ratio).^508^ The Chen group also investigated **1140** (Scheme 159B).^502^ With their enzymes, **1144** and **1145** were obtained in a much lower ratio (46% and 39% yield, 1.2:1 ratio), regardless of whether Ec or *Pm* aldolase was used.

#### 9.3.3 Deoxyfluorination at C-2 toward 2,3-Difluorinated Sialic Acid Analogues

In all cases, a DAST-type deoxyfluorination reaction was employed to achieve the formation of 2,3-difluorinated sialic acid analogues from 3-fluorinated sialic acids.

##### 9.3.3.1 With Unmodified 3-Fluorosialic Acid

**Scheme 160:**
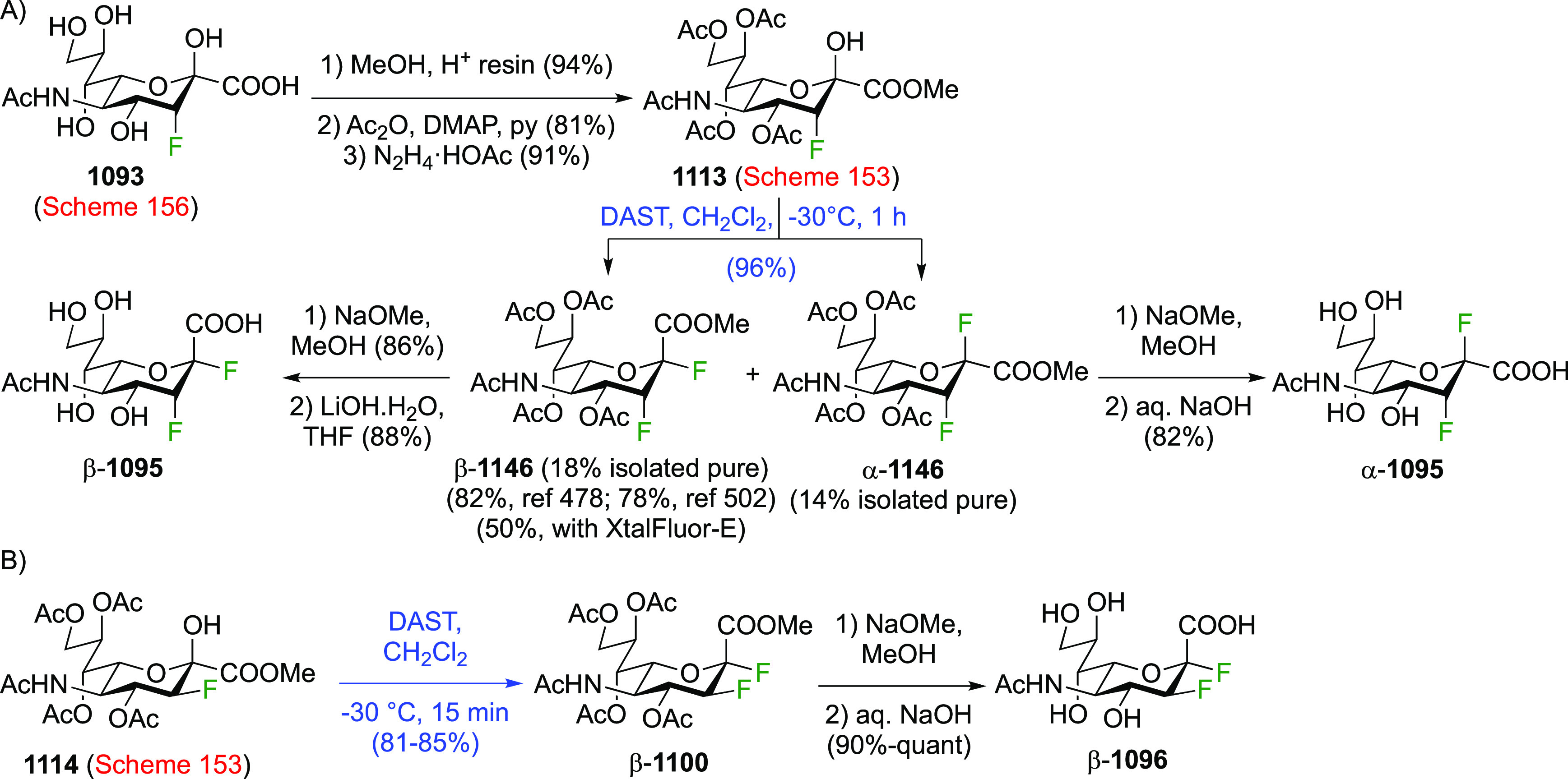
Anomeric Deoxyfluorination on 3-Fluorinated Sialic Acids^411,478,479,502^

Selective deoxyfluorination of the anomeric hydroxy group requires full protection of the other alcohol groups. Hence, 3-fluorosialic acids obtained from aldolase reactions, such as **1093** (Scheme 160A), require carboxylic acid and alcohol protection, followed by selective deprotection of the anomeric alcohol. From **1093** this sequence gave **1113**, which can also be obtained as the major product from the reaction of the sialic acid glycal (**1099**) with SelectFluor (as shown in Scheme 153). A number of publications mention that from **1113** β-**1146** as the only isolated deoxyfluorination product,^478,502^ including when the safer DAST alternative XtalFluor-E was used.^411^ However, the Withers group reported a full experimental process showing that deoxyfluorination of **1113** gave a mixture of both anomeric sialyl fluorides in 96% combined yield.^479^ Samples of pure anomers β-**1146** (18%) and α-**1146** (14%) were isolated, with the remaining material in mixed fractions. Full deprotection of each anomer gave the 2F_eq_3F_ax_ and 2F_ax_3F_ax_ sialic acid derivatives β-**1095** and α-**1095**.^411,478,479,502^

When an equatorial F-3 substituent was present, in **1114** (Scheme 160B), only the β-deoxyfluorination product β-**1100** was reported in 81–85% yield. Deprotection then gave the 2F_eq_3F_eq_ sialic acid derivative β-**1096**^479,502^

**Scheme 161:**
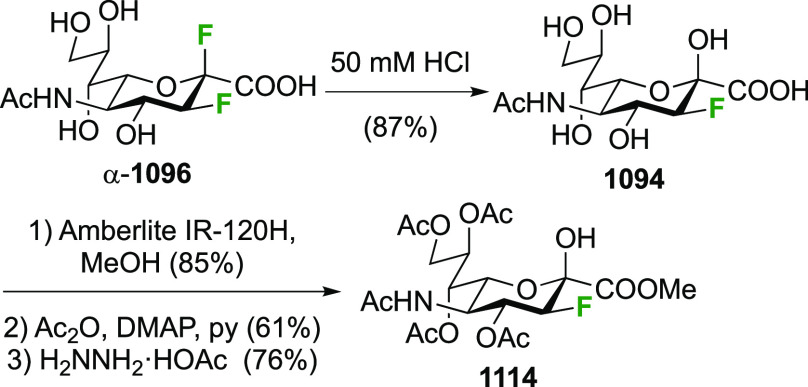
Alternative Synthesis of F_eq_-3 Sialic Acid^479^

The F_eq_-3 stereomer **1114** can be obtained from the corresponding aldolase adduct (cf. Scheme 156) by the usual protection conditions (not shown),^502^ but not all available aldolase enzymes allow its synthesis. In such cases, **1114** can be obtained as shown in Scheme 161 from the XeF_2_ reaction product α-**1096** (cf. Scheme 152).^479^ Hydrolysis of the sialyl fluoride α-**1096** led to F_eq_-3 sialic acid **1094**, which was then submitted to the carboxylic acid protection, alcohol protection, and anomeric deprotection sequence. Alternatively, **1114** can be obtained as the minor isomer from the SelectFluor-mediated fluorination of the sialic acid glycal **1099** (cf. Scheme 153).

##### 9.3.3.2 With NAc-modified 3-fluorosialic acids

**Scheme 162:**
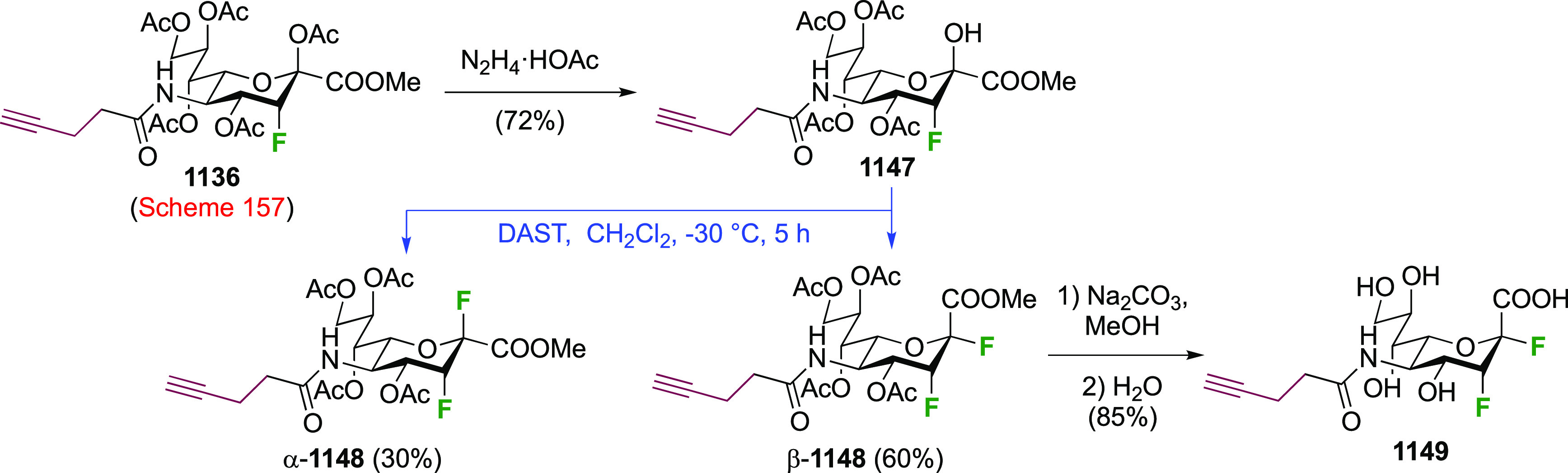
Deoxyfluorination of (Protected) *N*-(4-Pentynoyl-Substituted F_ax_-3 Sialic Acid^505^

The Wong group reported the synthesis of **1149** as a cell-permeable probe for sialidase imaging and identification (Scheme 162).^505^ Compound **1136**, obtained as described in Scheme 157, was selectively deprotected at the anomeric position to give **1147**. In accordance with the results described in Scheme 160, DAST-mediated deoxyfluorination led to the formation of both anomers of **1148**, here in a 2:1 β/α ratio. The desired β-anomer was then deprotected to give **1149**.

##### 9.3.3.3 With Deoxygenated 3-Fluorosialic Acids

The synthesis of a series of deoxygenated 2,3-difluorinated sialic acids has been reported by the Watts group.^499^ In all cases, deoxygenation reactions were carried out starting from isopropylidene-protected F_ax_-3 sialic acid **1131**, for which the synthesis is described in Scheme 156.

**Scheme 163:**
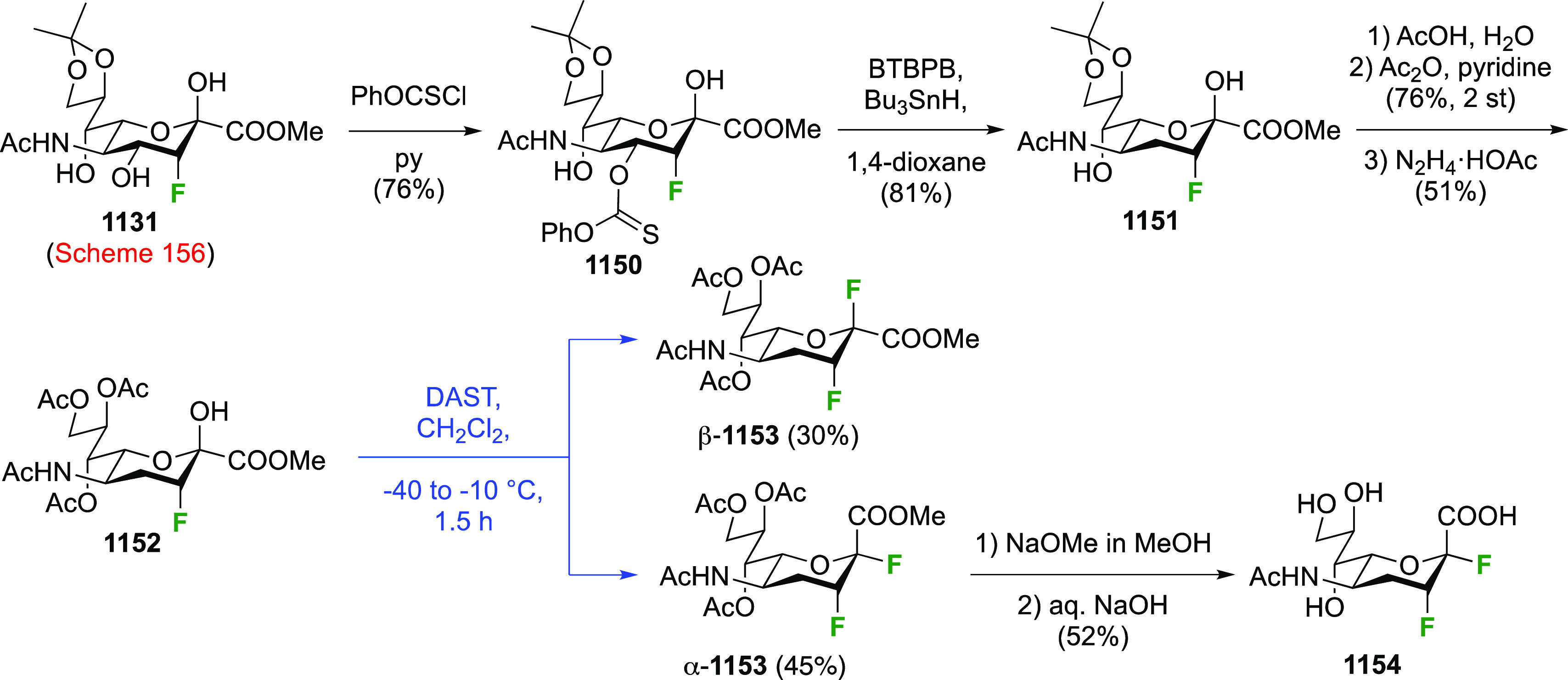
DAST-Mediated Deoxyfluorination of (Protected) 4-Deoxy-3-F_ax_ Sialic Acid^499^

Deoxygenation at the 4-position was achieved by selective reaction of **1131** (Scheme 163) with phenyl chlorothionoformate to give the thiocarbonate **1150**, with OH-7 being too sterically hindered to react. Reduction with tributyl tin hydride under 2,2-bis(*tert*-butylperoxy)butane (BTBPB) initiation afforded **1151**, which was then functionalized to allow anomeric fluorination by acetal hydrolysis, peracetylation and anomeric deprotection to give **1152**. DAST-mediated deoxyfluorination led to the α-anomer of **1153** as the major isomer in a 1.5:1 ratio. The configuration of β-**1153** was proven using X-ray crystallographic analysis. Full deprotection of α-**1153** afforded 5-*N*-acetyl-2,3,4,5-tetradeoxy-3-fluoro-d-*glycero*-α-d-*galacto*-non-2-ulopyranosonic fluoride **1154**. Note that the deoxygenation at the 4-position alters the configurational prefixes and anomeric reference atom.

**Scheme 164:**
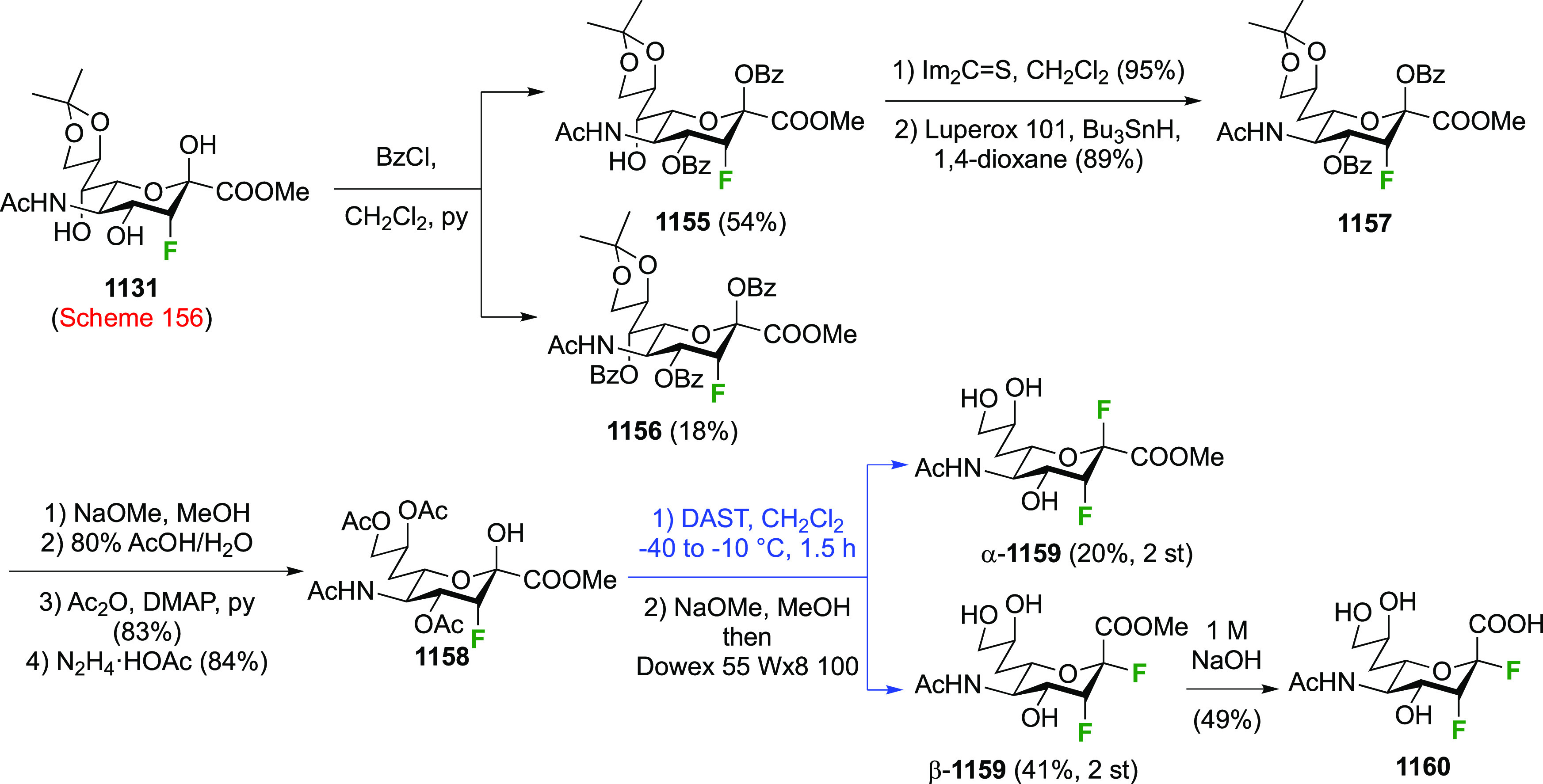
DAST-Mediated Deoxyfluorination of (Protected) 7-Deoxy-3-F_ax_ Sialic Acid^499^

For deoxygenation at the 7-position (Scheme 164), **1131** was benzoylated to give the 2,4-di-*O*-benzoyl product **1155** in 54% yield, alongside the fully benzoylated **1156**. As reaction with phenyl chlorothionoformate led to an inseparable 1:1 mixture of the desired thiocarbonate and a rearranged byproduct (not shown), **1155** was instead reacted with 1,1′-thiocarbonyldiimidazole. Subsequent tributyl tin hydride-mediated reduction with a commercial initiator gave **1157**, which was then converted through a series of protecting group manipulations to give the required deoxyfluorination substrate **1158**. This reaction led to a 2:1 ratio of anomers, again with the desired anomer (β-**1159**) as the major product. Its deprotection then yielded 5-*N*-acetyl-2,3,5,7-tetradeoxy-3-fluoro-d-*glycero*-β-l-*manno*-non-2-ulopyranosonic fluoride **1160**. Note that the deoxygenation at the 7-position alters one of the configurational prefixes.

**Scheme 165:**
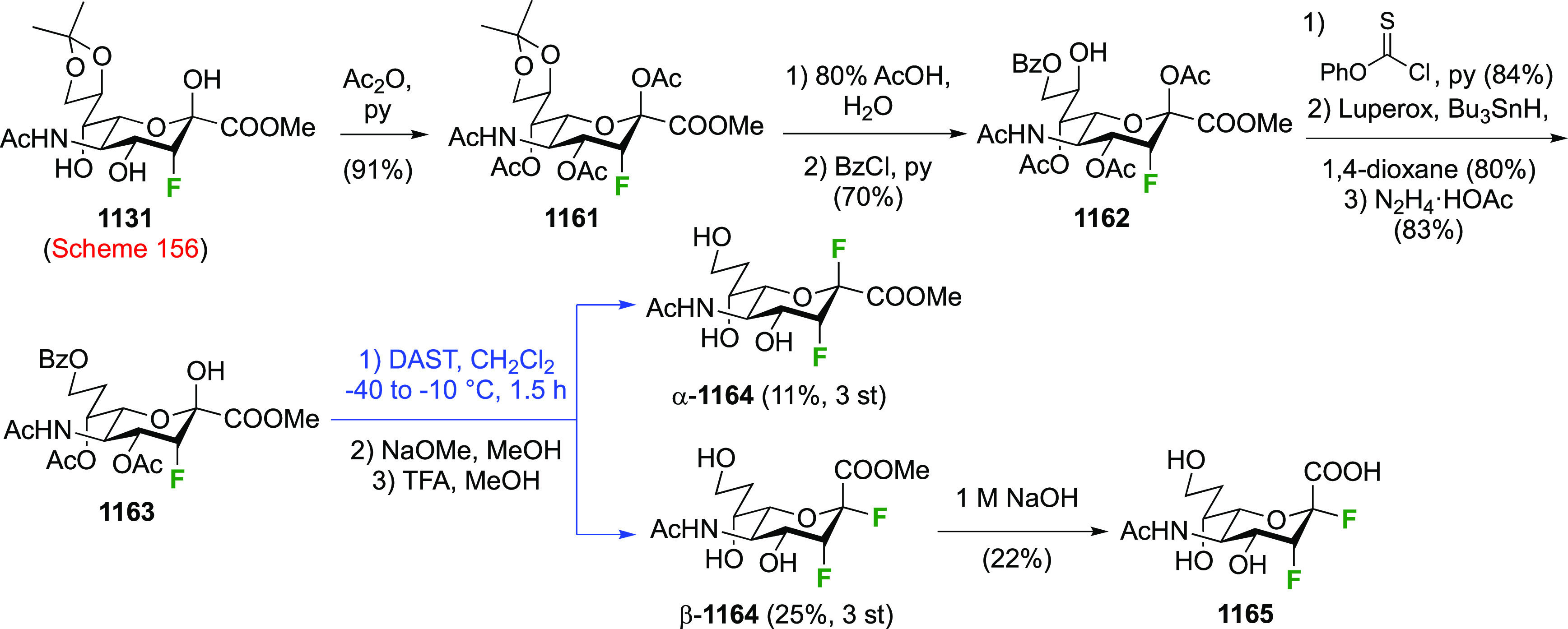
DAST-Mediated Deoxyfluorination of (Protected) 8-Deoxy-3-F_ax_ Sialic Acid^499^

The synthesis of the C-8-deoxygenated derivative **1165** is shown in Scheme 165. Starting from **1131**, full protection to give **1161** was followed by acetonide hydrolysis and selective protection at the primary position to give the OH-8 unprotected **1162**. Deoxygenation, followed by anomeric deprotection resulted in **1163**, which was subjected to deoxyfluorination to give a mixture of anomers **1164**. These could be separated after ester hydrolysis and reprotection of the carboxylic acid. Deprotection of the desired major β-anomer gave 5-*N*-acetyl-2,3,5,8-tetradeoxy-3-fluoro-d-*glycero*-β-l-*manno*-non-2-ulopyranosonic fluoride **1165**.

**Scheme 166:**
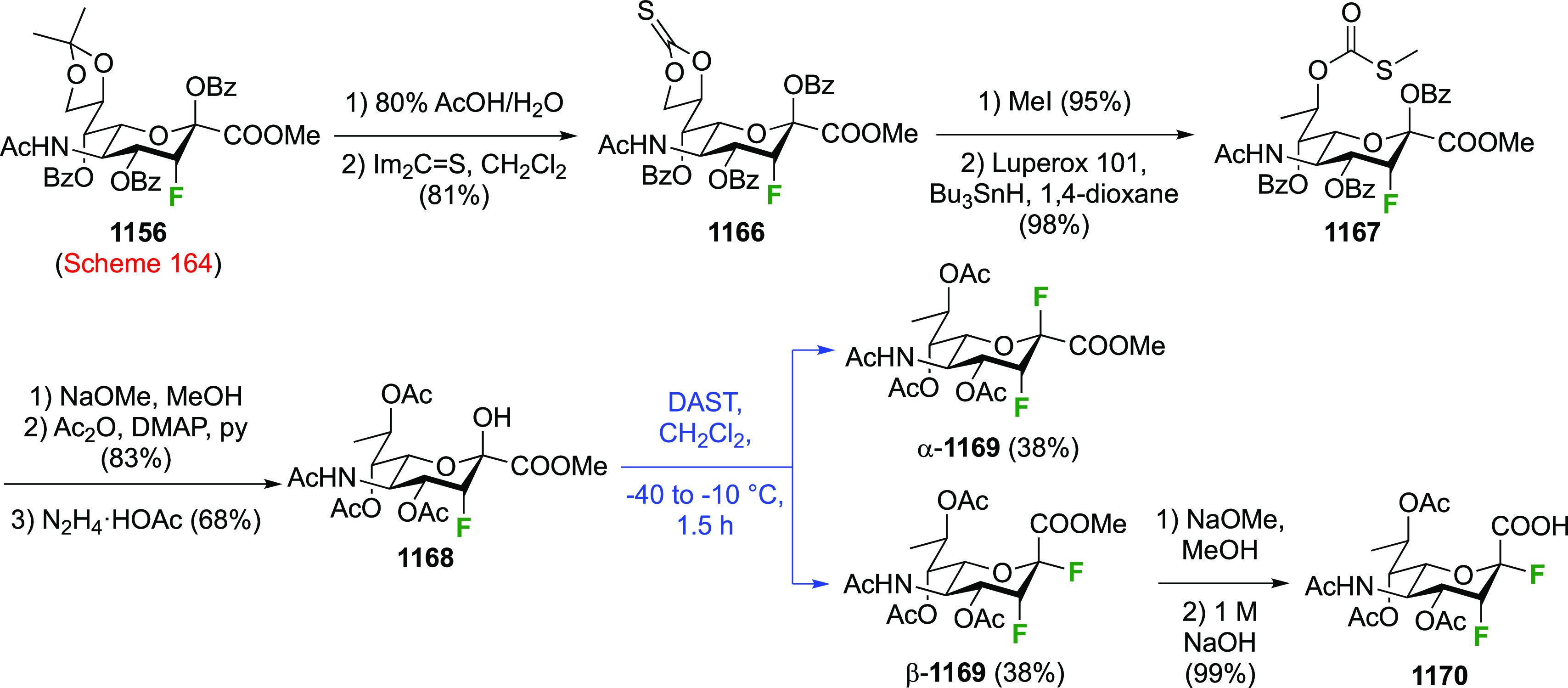
DAST-Mediated Deoxyfluorination of (Protected) 9-Deoxy-3-F_ax_ Sialic Acid^499^

Finally, the 9-deoxy derivative **1170** was synthesized from the tribenzoate **1156** (Scheme 166), obtained as a byproduct from the protection of **1131** as explained in Scheme 164. Acetal hydrolysis was followed by installation of the cyclic thiocarbonate **1166**. Reaction of the thiocarbonyl group with iodomethane allowed the released iodide to react at C-9, which was then reduced with tin hydride. The resulting 9-deoxy derivative **1167** was fully deprotected at the alcohol groups, and then peracetylated to allow selective deprotection of the anomeric position, to afford **1168**. Interestingly, deoxyfluorination gave a 1:1 mixture of anomers **1169**, and the desired β-anomer was deprotected to give 5-*N*-acetyl-2,3,5,9-tetradeoxy-3-fluoro-d-*erythro*-β-l-*manno*-non-2-ulopyranosonic fluoride **1170**.

##### 9.3.3.4 With Azido-Substituted Sialic Acid Derivatives

A series of azido-modified 2,3-difluorinated sialic acids have been synthesized.

**Scheme 167:**
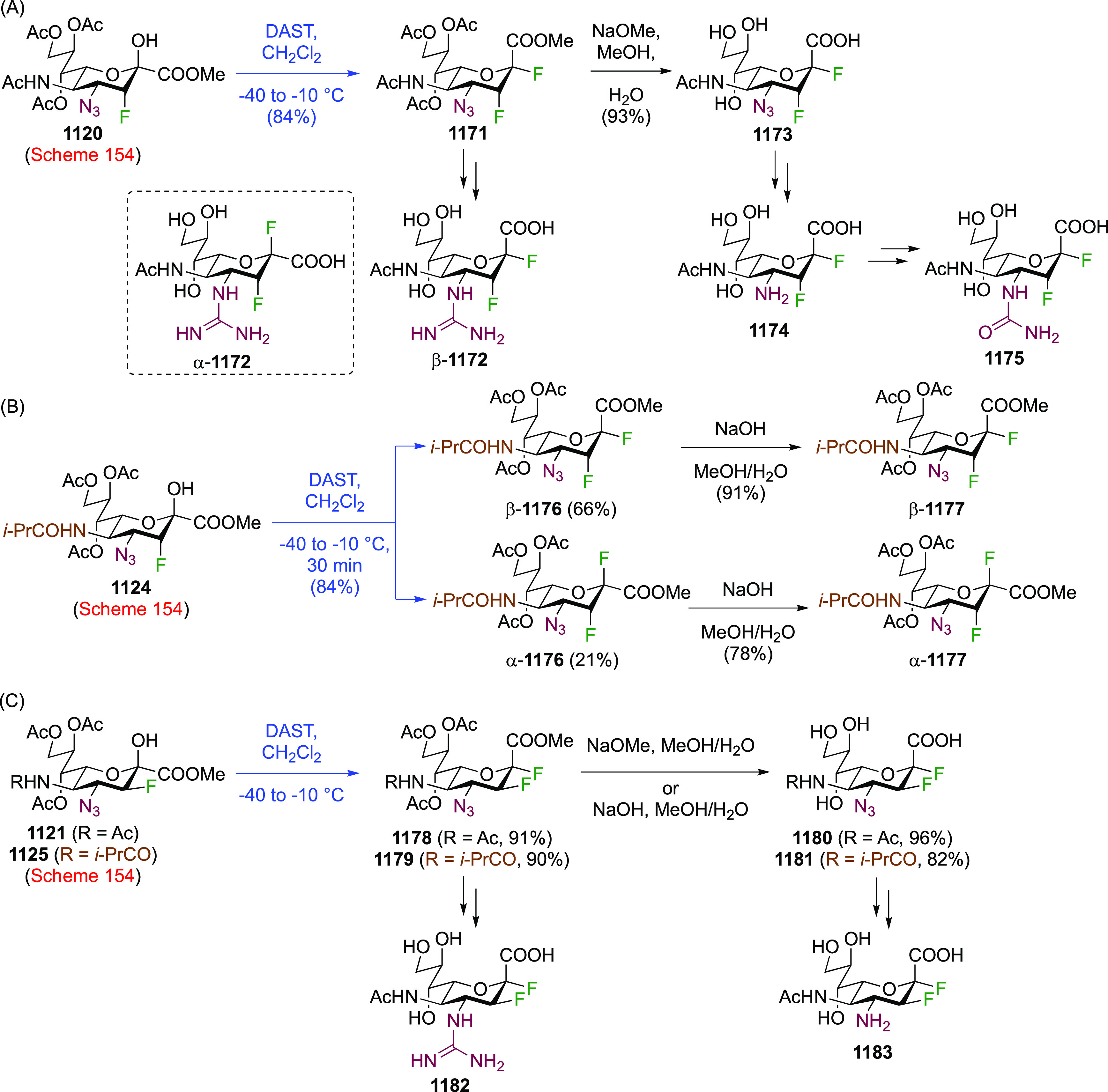
Deoxyfluorination of (Protected) 4-Deoxy-4-azido-3-fluoro Sialic Acids^493,494,509^

The Withers group reported the synthesis of a series of 4-substituted 2,3-difluorinated sialic acids (Scheme 167A).^493,509^ Deoxyfluorination of **1120**, for which the synthesis was described in Scheme 154, gave the β-anomer **1171** in excellent yield.^493^ No formation of the α-anomer was reported. Compound **1171** was then converted to derivatives β-**1172** and **1175**. The synthesis of α-**1172** was also described,^509^ although the deoxyfluorination reaction leading to the corresponding 2F_ax_3F_ax_ isomer was not provided. The von Itzstein group reported that DAST-mediated anomeric deoxyfluorination of the similar **1124** (Scheme 167B), in which the acetamido group is replaced by an isobutyramido group, yielded both anomeric fluorides of **1176** in a 3:1 ratio, with preferential formation of the β-anomer.^494^ Their deprotection gave the 2,3,5,8-tetradeoxy-3-fluoro-5-isobutyrylamido-d-*erythro*-l-*manno*-non-2-ulopyranosonic β- and α-fluorides **1177**.

With an equatorial F-3 substituent (Scheme 167C), both the Withers and von Itzstein groups reported that deoxyfluorination only led to the β-anomers, regardless of the amido group.^493,494^ Hence, **1121** and **1125** were converted to **1178** and **1179**, which after deprotection gave **1180** and **1181**. The acetamido derivatives were then converted to the neuraminidase inhibitors **1182** and **1183**.^493^

**Scheme 168:**
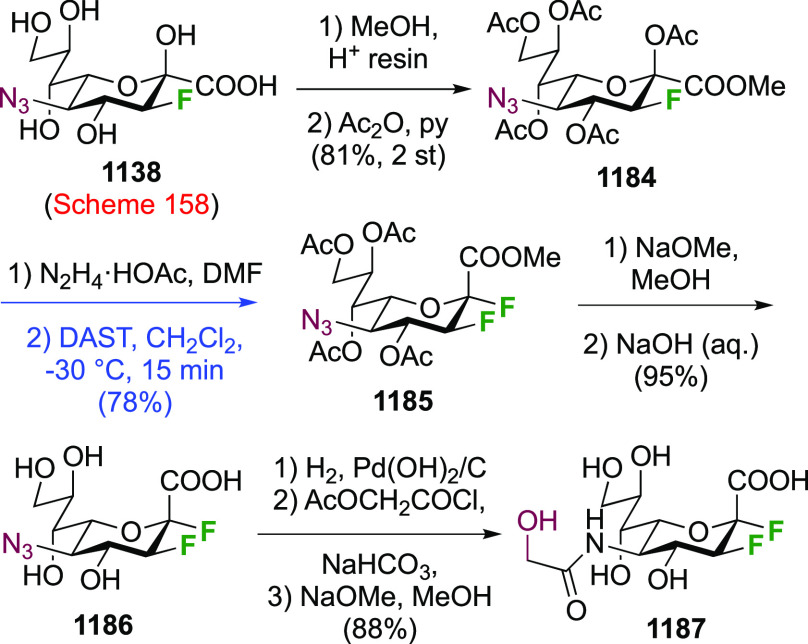
Deoxyfluorination of a (Protected) 5-Deoxy-5-azido-3-fluoro Sialic Acid Analogue^502^

The synthesis of a 2,3-difluorinated sialic acid with a modified NAc group was reported by the Chen group (Scheme 168).^502^ In contrast to the Von Itzstein approach shown in Scheme 167B/C, this was achieved from the corresponding 5-azido neuraminic acid derivative **1138**, for which the synthesis was described in Scheme 158. Protection of **1138** to give **1184** was followed by anomeric deprotection, which allowed deoxyfluorination to give **1185** as the only reported anomer in excellent yield. Full deprotection then gave 2,3,5-trideoxy-5-azido-3-fluoro-d-*erythro*-β-l-*gluco*-non-2-ulopyranosonic fluoride **1186**. Azide reduction, amide bond formation with acetyloxyethanoyl chloride, and acetate methanolysis then provided the 2,3-difluorinated *N*eu5Gc analogue **1187**.

**Scheme 169:**
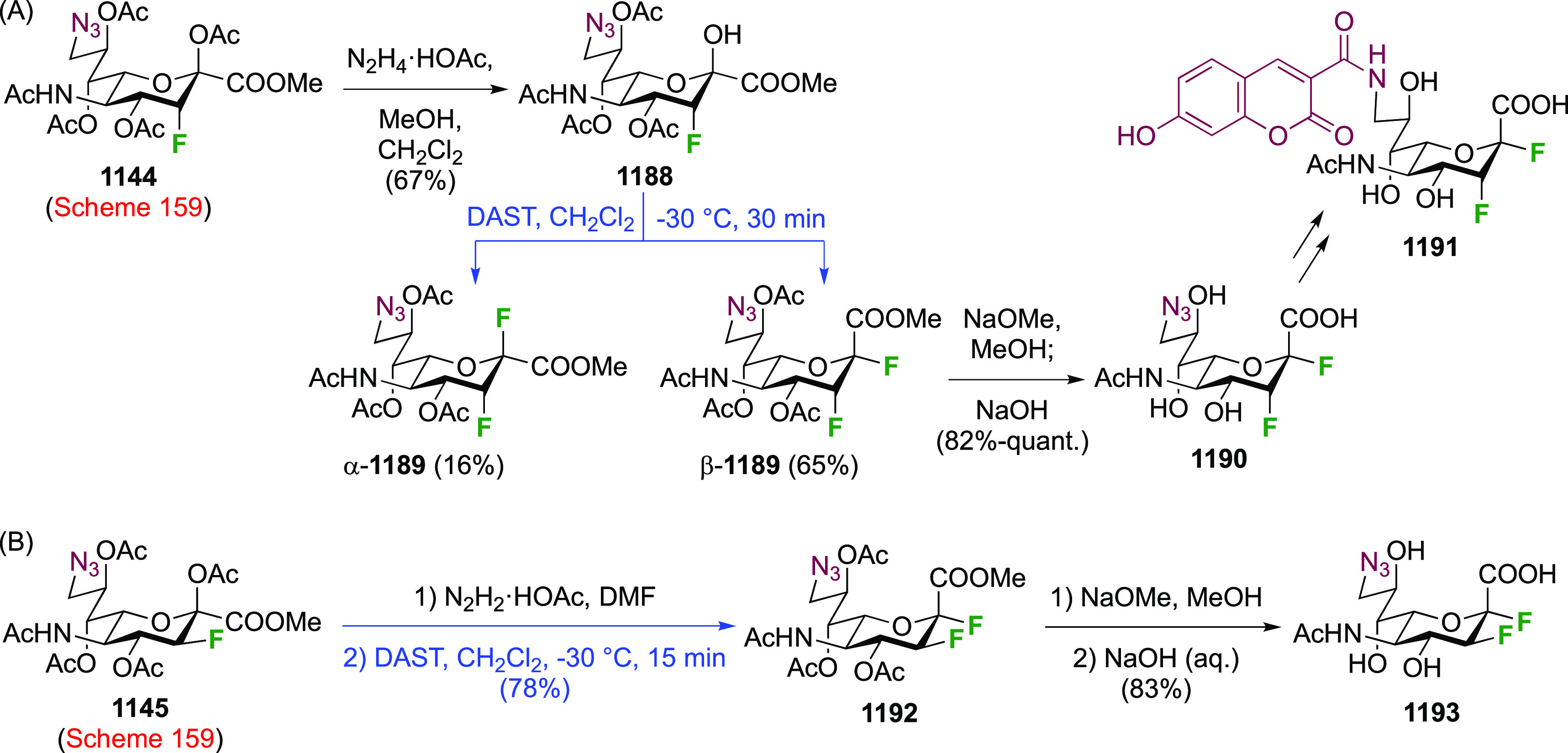
Deoxyfluorination of (Protected) 9-Deoxy-9-azido-3-fluoro Sialic Acids^502,508^

The synthesis of 9-azido-2,3-difluorinated sialic acids has been reported by both the Withers and Chen groups (Scheme 169A).^502,508^ Anomeric deprotection of **1144** (synthesis described in Scheme 159) resulted in **1188**, whereupon DAST treatment formed both anomers of **1189** in a 4:1 β/α ratio of isolated yields.^508^ The Chen group reported only the formation of β-**1189** in 74% yield from **1144** (not shown).^502^ The desired β-anomer was then deprotected to give **1190** and converted to a number of probes, including the 7-hydroxycoumarin derivative **1191**.^508^

Starting from the F_eq_-3 sialic acid derivative **1145** (Scheme 169B), anomeric deprotection and deoxyfluorination gave **1192** as the only reported stereomer. Deprotection gave 5-*N*-acetyl-2,3,5,9-tetradeoxy-9-azido-3-fluoro-d-*erythro*-β-l-*manno*-non-2-ulopyranosonic fluoride **1193**.^502^

##### 9.3.3.5 With Amino-Substituted Sialic Acid Derivatives

**Scheme 170:**
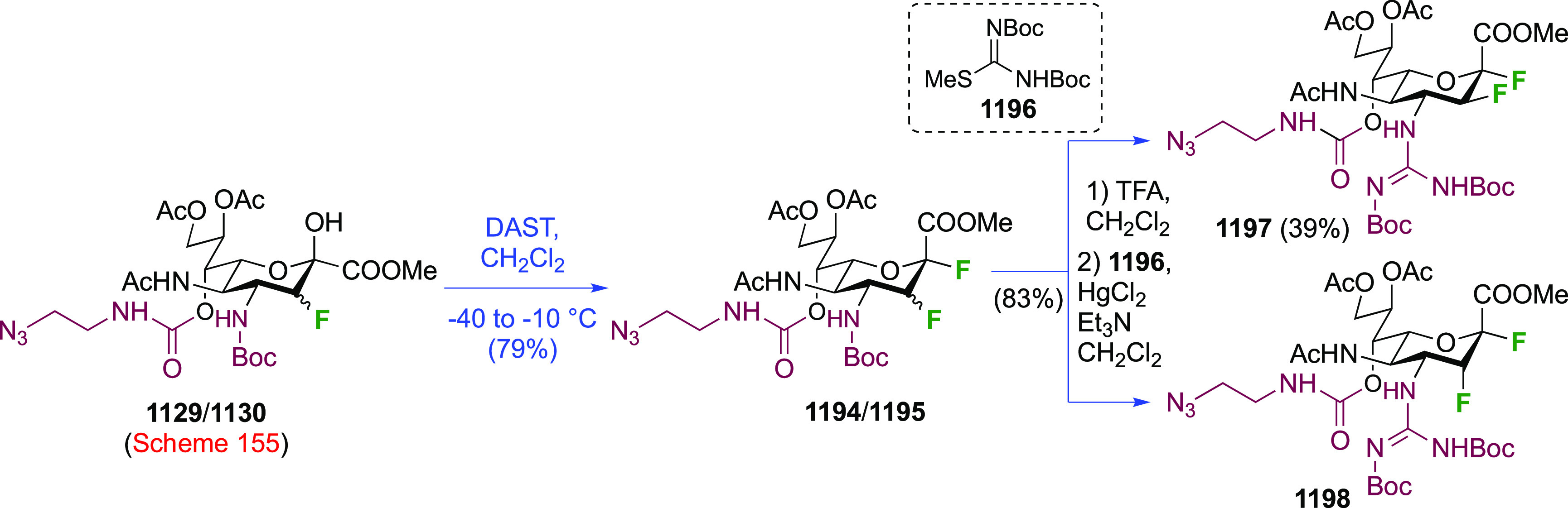
Deoxyfluorination of (Protected) 4-Deoxy-4-*N*-Boc-3-fluoro Sialic Acids^497^

Instead of a 4-azido group, 2,3-difluorinated sialic acids have also been synthesized with a Boc-protected 4-amino group (Scheme 170).^497^ Yang et al. described a β-selective deoxyfluorination of the F_ax_-3/F_eq_-3 mixture **1129**/**1130**, for which the synthesis is described in Scheme 155. This led to a mixture of F-3 diastereomers **1194**/**1195** that were not separated at this stage. Amine deprotection and subsequent introduction of a protected guanidine group led to **1197**/**1198** in 83% yield. Separation was possible at this stage, but only the yield of **1197** was reported (39%). Both **1197** and **1198** were then converted to multivalent zanamivir analogues (not shown).

## 10 Glycoside Formation

This section is organized according to deoxyfluorination type, and not by mechanism. A number of examples have already been mentioned in preceding sections, for example, when anomeric functionalization was required as a protecting group. These are reproduced here for the sake of completion.

The larger electron withdrawing effect of fluorine compared to that of an OH group results in a destabilization of the transition states of anomeric C–O bond forming reactions, which is pronounced when fluorination is adjacent to the anomeric position.

### 10.1 Donor with Fluorination at Positions 2 and 3

**Scheme 171:**
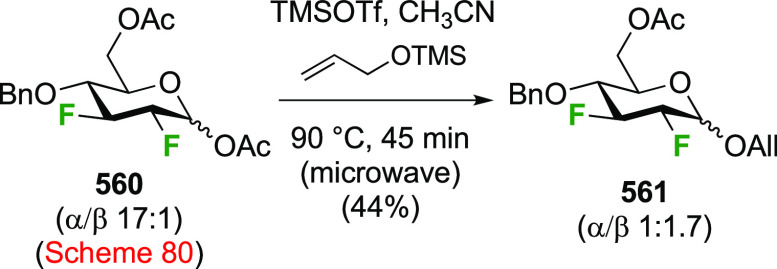
Glycosyidation of a 2,3-Dideoxy-2,3-difluorinated Sugar Derivative^272^

The Giguère group reported the glycosidation of the anomeric acetate **560** (Scheme 171) as allyl glycoside protection in order to enable subsequent fluorination at the 6-postion (see Scheme 80).^272^ They reported that glycosidations starting from the corresponding glycosyl bromide failed, but TMSOTf-catalyzed allylation using allyl trimethylsilane proceeded to give a 44% yield under microwave heating conditions. The glycosylation failed when allyl alcohol was used, or when conventional heating was employed. Starting from a predominantly α-configured acetate, a 1:1.7 α:β mixture of **561** was obtained.

**Scheme 172:**
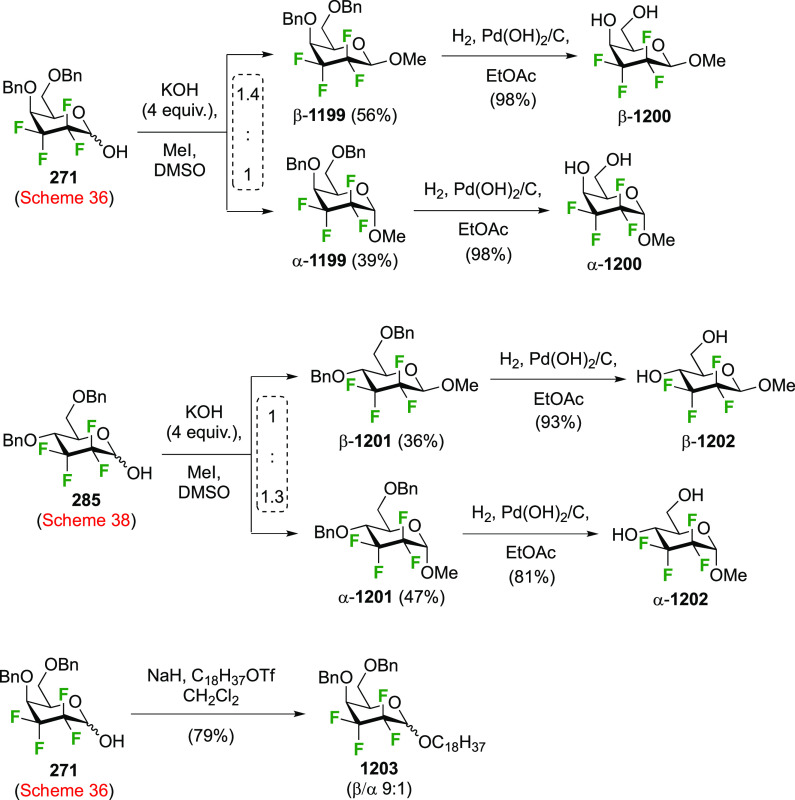
Glycosidation of 2,3-Dideoxy-2,2,3,3-tetrafluorinated Sugar Derivatives^287,288^

For the tetrafluorinated donors **271** and **285** (cf. Schemes 36 and 38), the Linclau group explored an anomeric alkylation strategy (Scheme 172). This is a comparatively infrequently used glycosylation method in which the hemiacetal is deprotonated, and then reacts as a nucleophile with an electrophilic acceptor.^510^ Fluorination will facilitate the deprotonation step, as the electron withdrawing effect stabilizes the conjugate base. Following precedent by the Fried group on a noncarbohydrate fluorinated cyclic hemiacetal,^511^ reaction of **271** with KOH and MeI gave the methyl glycosides in excellent yield with a modest anomeric ratio.^287^ With the corresponding C-4-epimer **285**, the α-anomer of **1201** was obtained as the major product, although the conditions were slightly different.^288^ Subsequent benzyl hydrogenolysis gave the deprotected methyl glycosides **1200** and **1202**.^288,512^

With NaH as base, dichloromethane as solvent, and a long chain alkyl triflate, **271** gave an inseparable anomeric mixture of **1203** with the β-anomer as the major product.^287^

### 10.2 Donors with Fluorination at Positions 2 and 4

**Scheme 173:**
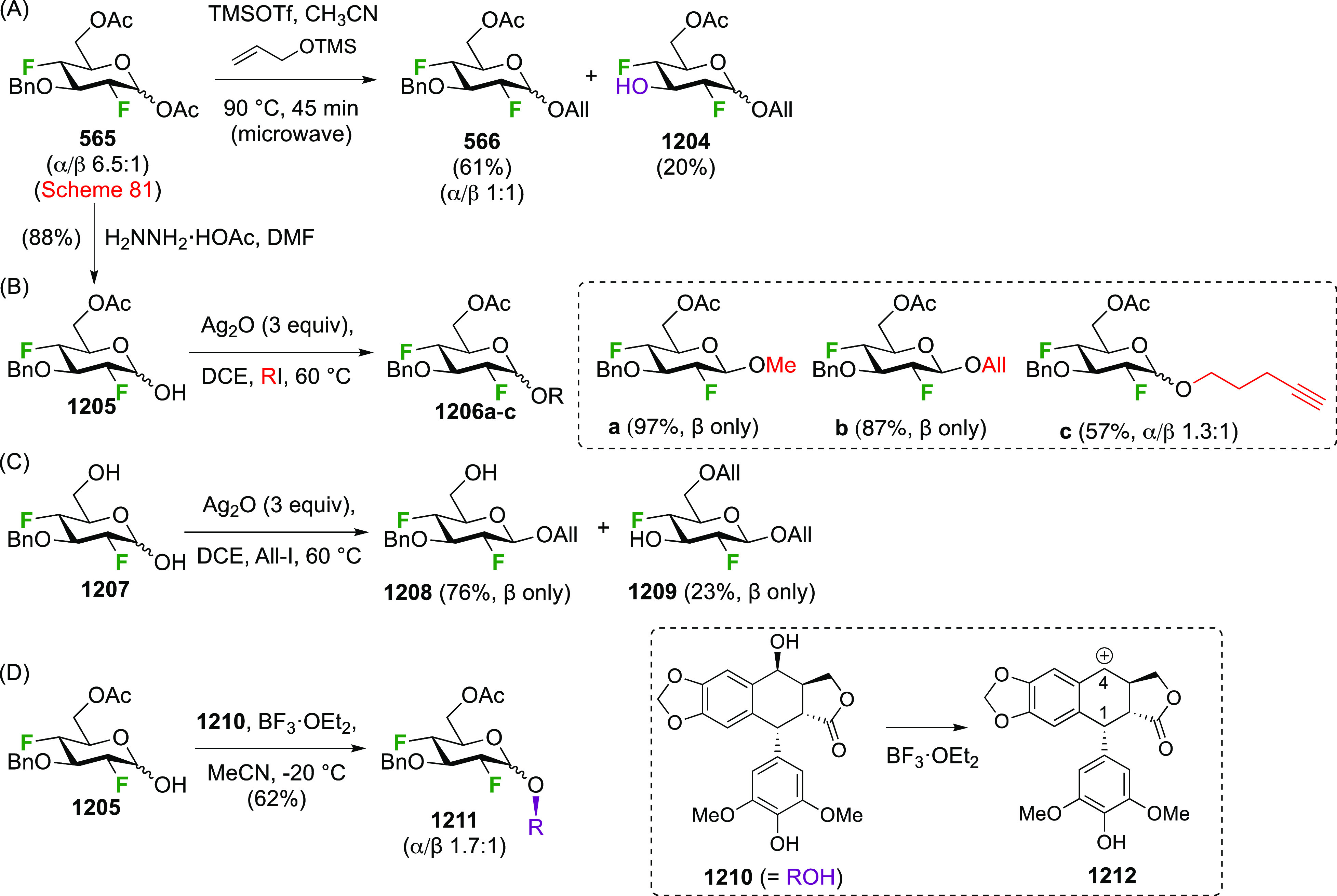
Glycosidations with 2,4-Dideoxy-2,4-difluorinated Glucose Donors^272,513^

The Lewis acid-mediated allylation described in Scheme 171 has also been applied to 2,4-dideoxy-2,4-difluorinated glucose donor **565** (Scheme 173A), which had also already been described above as an anomeric protection reaction (see Scheme 81).^272^ This reaction gave a 61% yield of **566**, alongside 20% of a partially deprotected glycosidation product **1204**. The Giguère group also investigated anomeric alkylation methods.^513^ With Ag_2_O as base (Scheme 173B), excellent yields and anomeric selectivities were obtained for the glycosidation of **1205**, obtained from **565** by hydrazinolysis, with methyl and allyl iodide to give **1206a**,**b**. Glycosylation of 1-iodo-4-pentyne, however, gave only a modest anomeric ratio of **1206c**. With a free OH-6 group, such as in **1207** (Scheme 173C), the reaction also worked well, with **1208** obtained in 76% yield, although the OH-6 group was partially allylated leading to **1209** in 23% yield. An interesting result was obtained with a known direct glycosidation^514,515^ using a strong Lewis acid (Scheme 173D): reaction of **1205** with the podophyllotoxin derivative **1210** gave **1211** in good yield. This glycosidation was thought to proceed via the benzylic cation **1212** and, while the anomeric selectivity was relatively modest, complete facial selectivity for the reaction with **1212** was reported.^513^

### 10.3 Donors with Fluorination at Positions 2 and 6

**Scheme 174:**
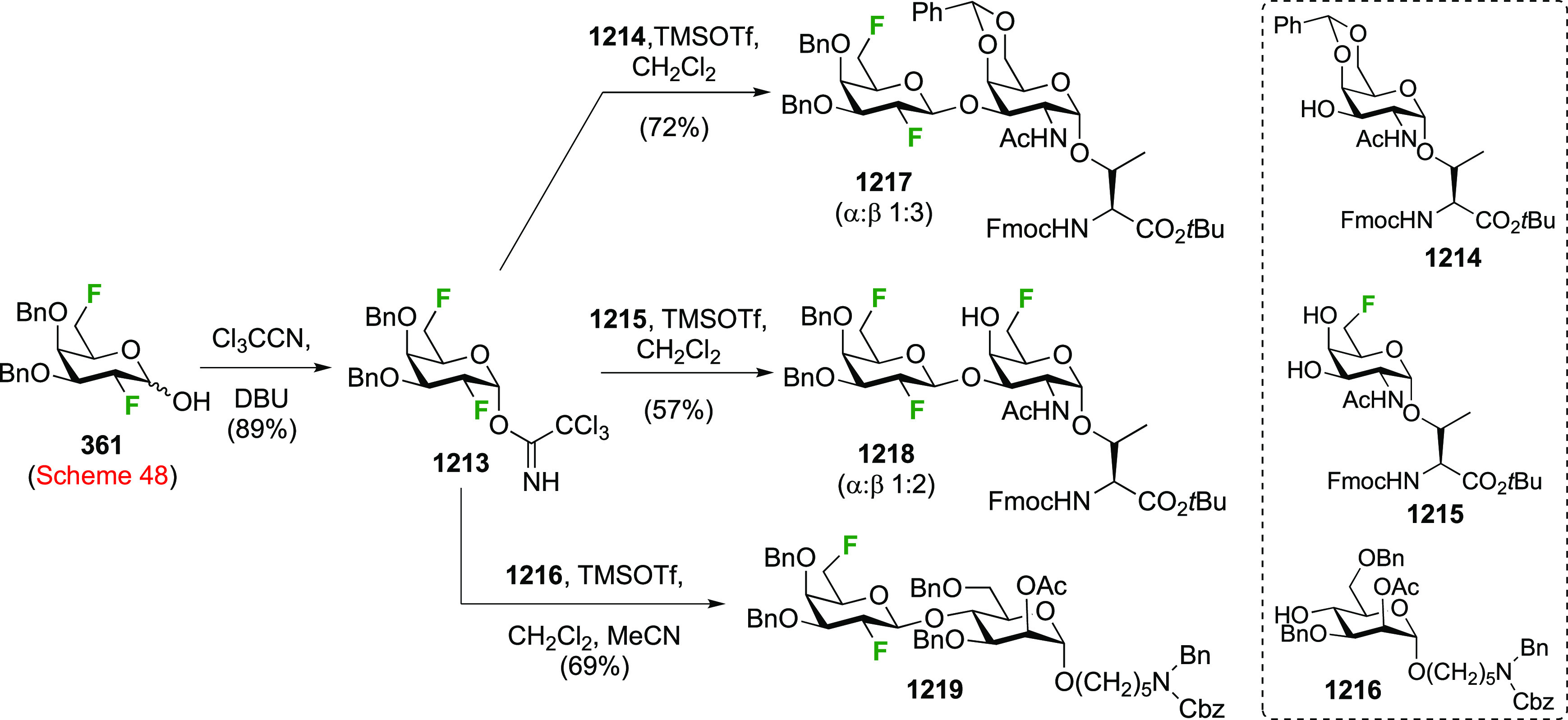
Glycosylation of a 2,6-Dideoxy-2,6-difluorinated Galactose Donor^43,516^

The Hoffmann-Röder group achieved a number of glycosylations with the 2,6-dideoxy-2,6-difluorinated galactose donor **1213** (Scheme 174).^43,516^ This trichloroacetimidate donor was obtained from **361** (see Scheme 48) using standard conditions. Coupling with T_N_ derivatives **1214** and **1215** gave the fluorinated T_F_ antigen analogues **1217** and **1218** in good yield with moderate β-selectivities. Reaction with **1215** also led to a small amount (<15%) of a 3,4-bisglycosylated product (not shown). All attempts to increase the β-selectivities were fruitless.^43^ However, complete β-selectivity was achieved for the glycosylation of **1213** with **1216**,^516^ under conditions which exploited the “nitrile-effect” by using a mixture of dichloromethane and acetonitrile at low temperature.^517,518^ A trichloroacetimidate rearrangement side reaction was successfully suppressed by using an inverse addition procedure, where donor **1213** was added to a solution of **1216** and TMSOTf in CH_2_Cl_2_/MeCN at −78 °C. The yield of this reaction was slightly lower compared to glycosylations with nonfluorinated or C-2-monofluorinated donors, which was attributed to its lower reactivity. The disaccharide analogue **1219** was further converted to fluorinated *Leishmania* cap trisaccharides (not shown).^516^

**Scheme 175:**
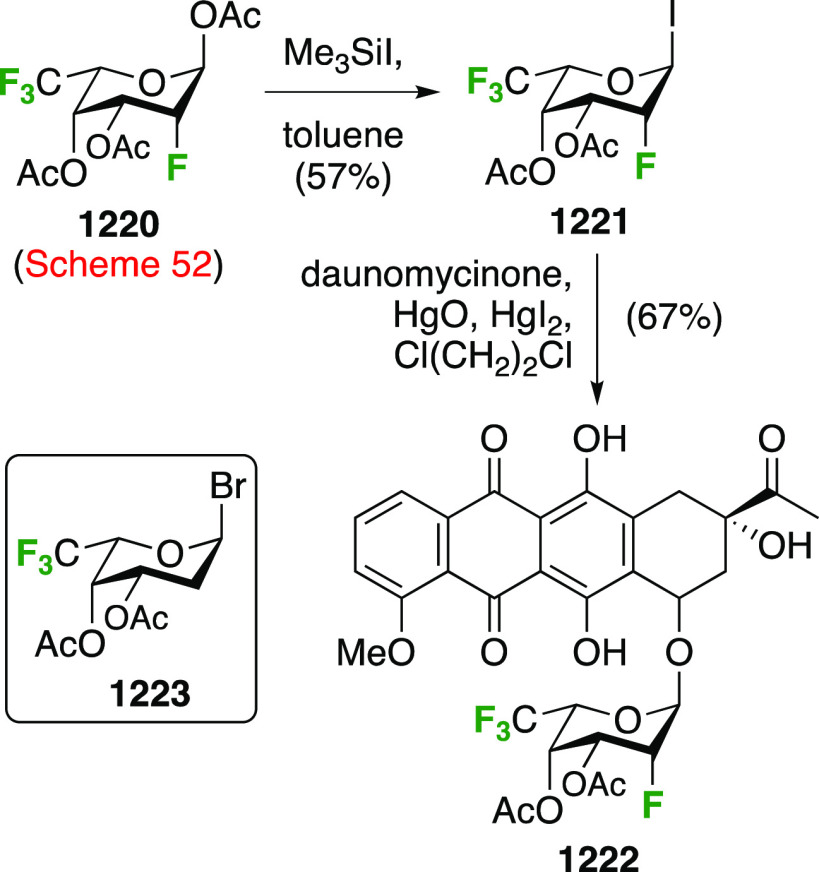
Glycosidation of a 2,6-Dideoxy-2,6,6,6-tetrafluorinated Galactose Donor^318^

Takagi’s group reported glycosidation of a 2,6,6,6-tetrafluorinated donor derived from **1220** (Scheme 175) with daunomycinone as the acceptor.^318^ Compound **1220** was synthesized by acetylation of **386**, for which the synthesis was described in Scheme 52.^318^ They found that activation of the anomeric center of **1220** was difficult, which was again attributed to the electron withdrawing effect of the fluorines. Both conventional bromination of **1220** (30% HBr in AcOH or TiBr_4_ in CH_2_Cl_2_/EtOAc) and ethyl thioglycoside formation (EtSH, BF_3_·OEt_2_ in CH_2_Cl_2_) only returned starting material. However, based on the observation that phenyl thioglycosidation [PhSSiMe_3_, Bu_4_NI, ZnI_2_ in Cl(CH_2_)_2_Cl] gave a mixture of the phenyl thioglycoside and the glycosyl iodide **1221**, the synthesis of the latter was successfully achieved with Me_3_SiI (in toluene at 80 °C) in reasonable yield. This glycosyl iodide could be isolated after flash column chromatography and could be stored for a few days at −30 °C, again testimony to the fluorine electron withdrawing effect. Coupling of **1221** with daunomycinone under Koenigs–Knorr conditions successfully and selectively gave the α-l-glycoside **1222** in 67% yield. Interestingly, glycosidation of the corresponding glycosyl bromide donor **1223** without C-2-fluorination and with a similar acceptor led to a 1:1 ratio of diastereomers,^519^ suggesting the directing effect of the axial fluorine in **1221**. The glycoside **1222** was then further converted to doxorubicin-type analogues (not shown).^318^

### 10.4 Donors with Fluorination at Positions 3 and 4

**Scheme 176:**
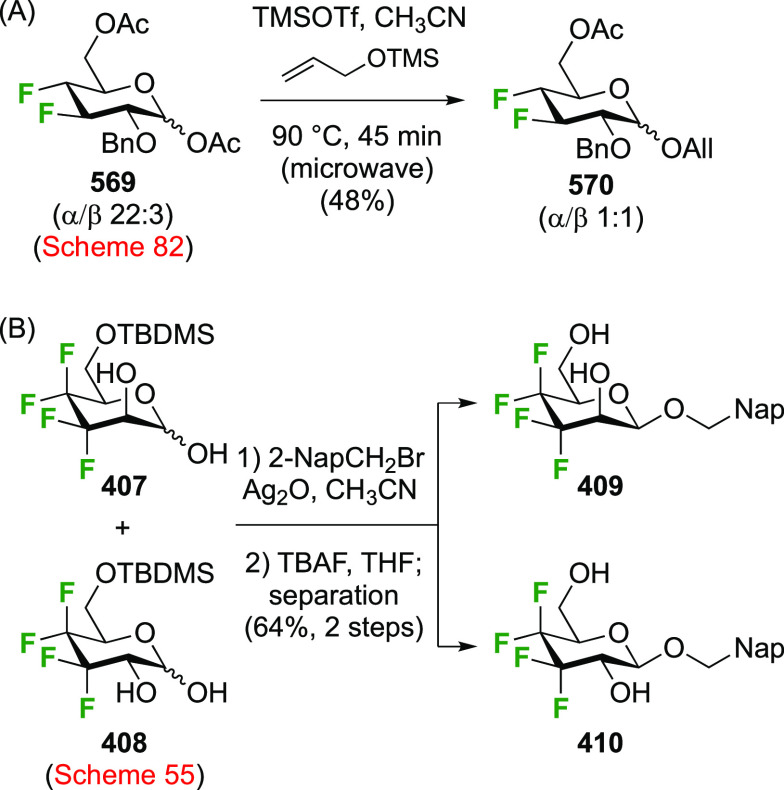
Glycosidations of Donors with Deoxyfluorination at the 3 and 4-Positions^272,288^

The Giguère group also employed their Lewis acid-catalyzed allylation for the anomeric protection of **569** (Scheme 176A), as mentioned before in Scheme 82 with the synthesis of 3,4,6-trideoxy-3,4,6-trifluoro-α-d-glucopyranose.^272^ A 48% yield of a 1:1 ratio of anomers **570** was obtained.

The Linclau group employed an anomeric alkylation for the functionalization of the tetrafluorinated **407** and **408** (Scheme 176B) to effect their separation, as explained above in Scheme 55. This anomeric alkylation was reported to be highly β-selective, with less than 3% of the α-anomers detected (^19^F NMR analysis). The amount of alkylation at the 2-postion was also very small, and only observed for **407** (not shown).^288^

### 10.5 Donors with Fluorination at Positions 3 and 6

**Scheme 177:**
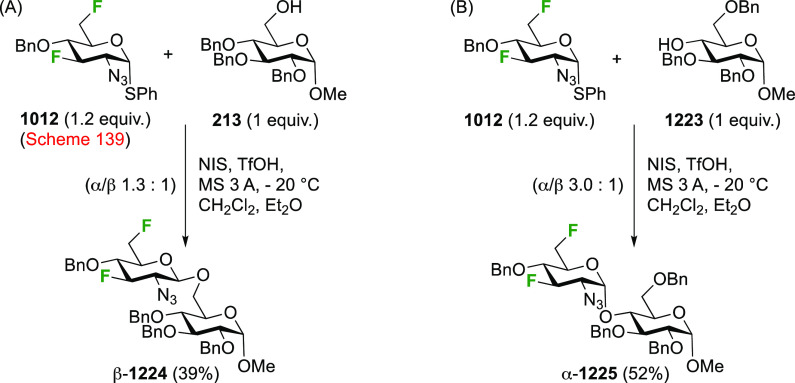
Glysosylation of a 3,6-Difluorinated Donor^462^

The Karban group reported two glycosylations with donor **1012** (Scheme 177), for which the synthesis was described in Scheme 139.^462^ Under *N*-iodosuccinimide (NIS) activation, donor **1012** reacted with acceptors **213** and **1223** to obtain anomeric mixtures of disaccharides **1224** and **1225**, respectively. Anomeric separation proved difficult, further complicated by byproducts, and only the isolated yield of the anomers shown could be provided. The obtained anomeric ratios from this 3,6-difluorinated donor were higher with the less reactive acceptor **1223**, but overall these glycosylations had a lower α/β ratio compared to glycosylations with the corresponding 3-fluorinated donors with ester groups at the 6-position, which was attributed to the α-directing effect of 6-*O*-acyl groups.

### 10.6 Donors with Fluorination at Positions 4 and 6

**Scheme 178:**
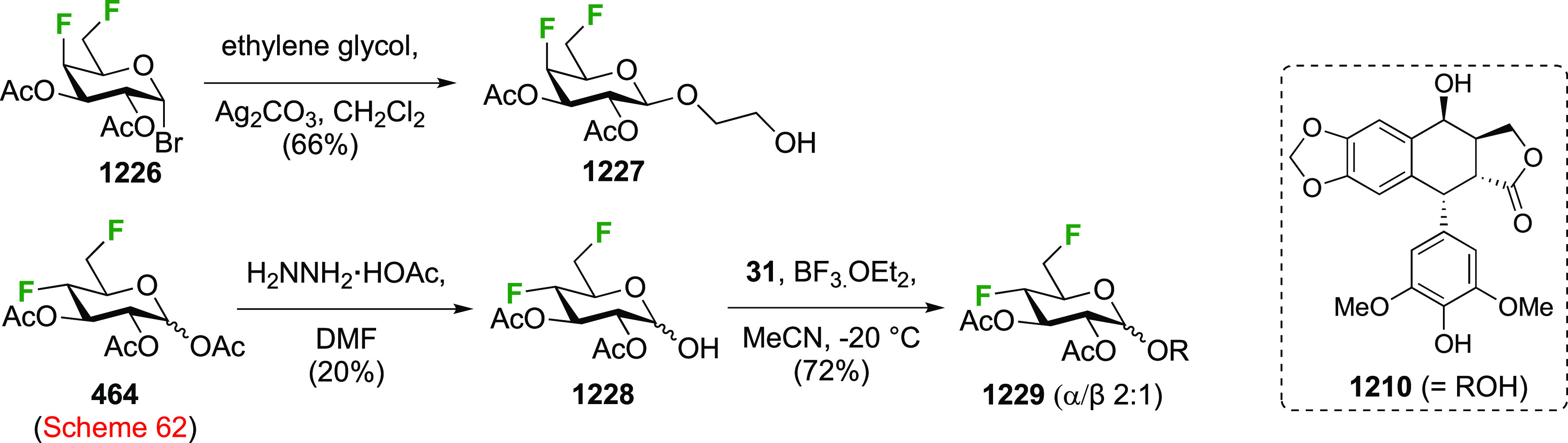
Glycosylations of Donors with Deoxyfluorination at the 4 and 6-Positions^513,520^

Lucas et al. achieved glycosidation of the 4,6-dideoxy-4,6-difluorinated galactosyl bromide donor **1226** with ethylene glycol (Scheme 178), en route to carbohydrate-oligonucleotide conjugates.^520^ Only the β-anomer **1227** was reported. The Giguère group also demonstrated their BF_3_·OEt_2_-catalyzed glycosylation of podophyllotoxin derivative **1210** with the 4,6-dideoxy-4,6-difluorinated glucose donor **1228**, as already discussed in Scheme 173. This gave **1229** with modest anomeric selecvtivity but with retention of the alcohol configuration of the aglycon.^513^

### 10.7 Donors with Fluorination at Positions 2, 3, and 4

**Scheme 179:**
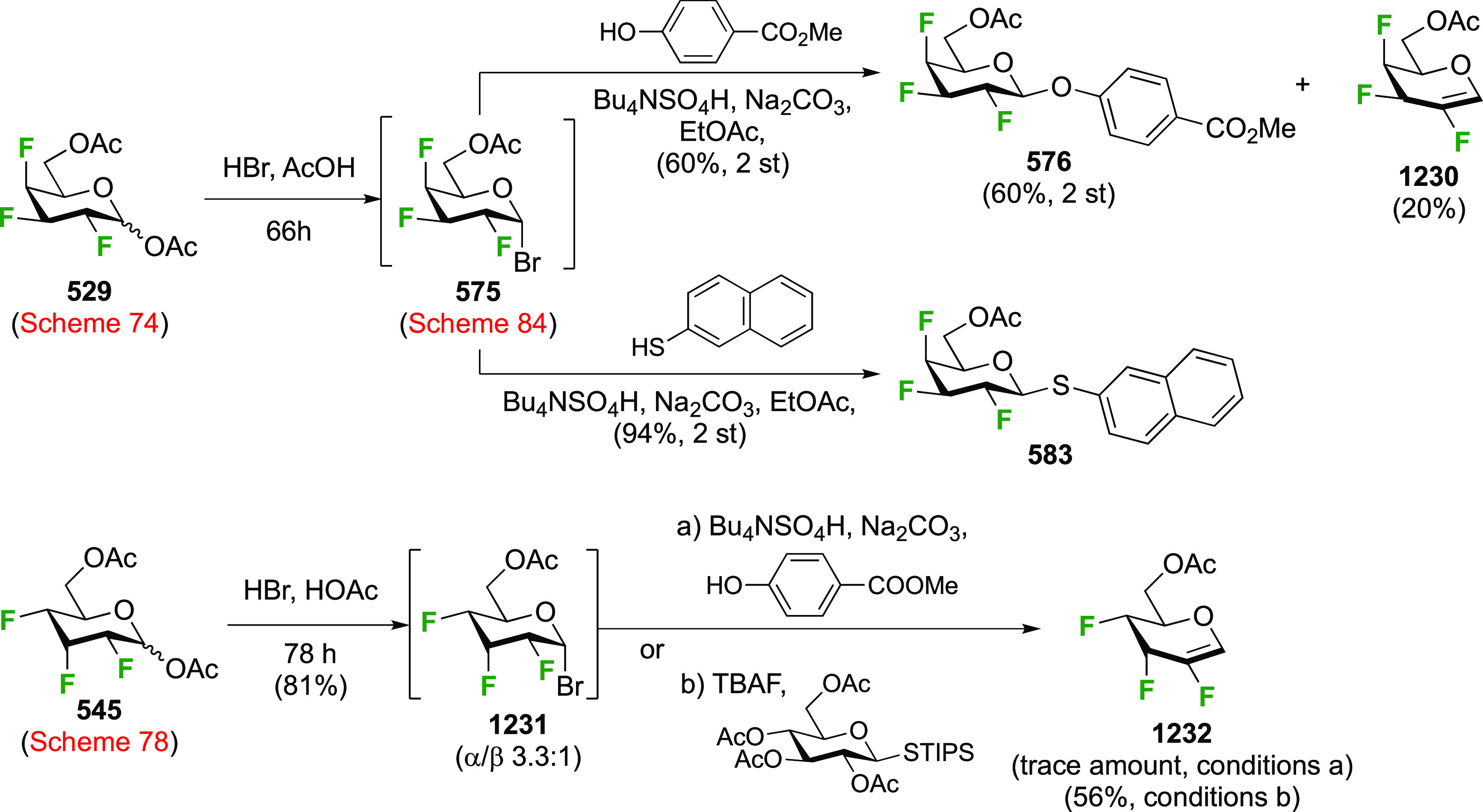
Glycosidation of a 2,3,4-Trideoxy-2,3,4-trifluorinated Galactose Donor^273,275^

Glycosidation under phase-transfer conditions of the trifluorinated galactosyl bromide donor **575** (Scheme 179) had been described as part of the synthesis of 2,3,4,6-tetradeoxy-2,3,4,6-tetrafluoro-α-d-galactopyranoside derivatives (Scheme 84).^273,275^ Displacements of the bromide by both nucleophiles gave the products **576** and **583** with clean inversion of anomeric configuration. In the case of reaction with deprotonated methyl 4-hydroxybenzoate, 20% of the E2-elimination side product **1230** was also isolated.

In contrast, nucleophilic displacement attempts on the corresponding trifluorinated allosyl bromide **1231**, generated from **545** under the usual conditions, met with failure.^295^ Reaction with methyl 4-hydroxybenzoate or with a glycosyl sulfide precursor led to the formation of the allal derivative **1232**. The difference in outcome was attributed to the availability of the C-2–H bond in **1231** compared to that in **575**, with the axial F-4 in **575** hindering the E2 process.^295^ Presumably, the antiperiplanar F-3 in **1231** increases the reactivity of H-2 toward elimination as well.

**Scheme 180:**
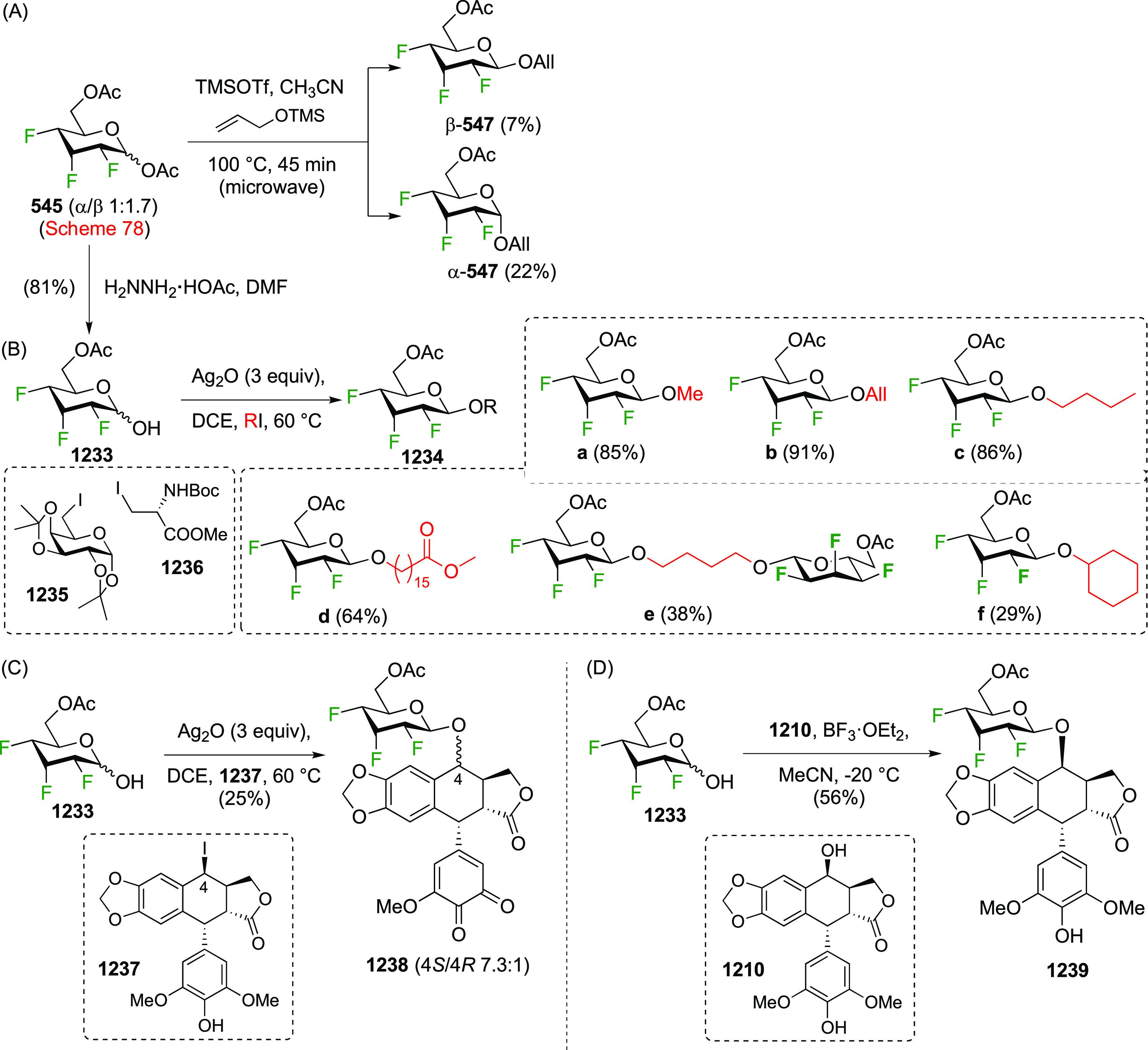
Successful Glycosidation Methodologies for 2,3,4-Trideoxy-2,3,4-trifluoroallose Donors^295,513^

Investigations toward alternative glycosidation methodologies for this donor were successful and are described in Scheme 180. Microwave irradiation of **545** at 100 °C (Scheme 180A) with allyloxytrimethtyl silane under Lewis acid catalysis yielded the separable allyl alloside anomers **547** (cf. also Scheme 78), with the α-anomer isolated in 22% yield and the β-anomer in 7% yield.^295^ However, an anomeric alkylation strategy from the reducing sugar **1233** (Scheme 180B), obtained from **545** by hydrazinolysis, using primary alkyl iodide electrophiles and Ag_2_O in dichloromethane resulted in excellent yields of the 2,3,4-trifluorinated allosyl glycosides **1234a**–**c** as β-anomers only.^513^ Product **1234c** was accompanied by 5% of the corresponding 6-deacetylated byproduct (not shown). Reaction of **1233** with methyl 16-iodohexadecanoate and 1,4-diiodobutane gave **1234d**,**e** in a lower yield. Unfortunately, other primary iodides such as **1235** and **1236** failed to give any product. With more hindered iodides, such as cyclohexyl iodide, the yield dropped further to 29% (**1234f**), and reaction with cholesterol iodide gave no product (not shown). However, reaction with secondary iodide **1237** (Scheme 180C) did give a 25% yield of the corresponding glycoside **1238** (with concomitant oxidation to the *o*-quinone), although in a 7.3:1 ratio at C-4 in favor of the 4*S*-stereomer (retention of configuration). As discussed in Scheme 173, the corresponding cation **1212** (not shown here) reacts with complete facial selectivity. Indeed, with alcohol **1210** as the acceptor, trifluoroallosylated podophyllotoxin derivative **1239** was obtained with complete retention of stereochemistry at C-4. The stereochemical outcome for the reaction of **1233** with **1237** could indicate the occurrence of an S_N_2 reaction as a minor pathway.^513^

**Scheme 181:**
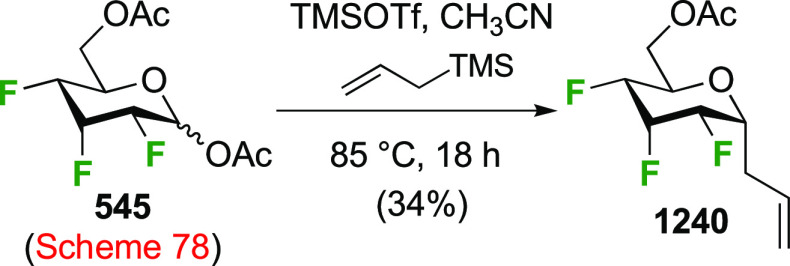
C-Glycosidation of a 2,3,4-Trideoxy-2,3,4-trifluoroallose Donor^295^

Finally, the Giguère group also established that the reaction of **545** with allyl trimethylsilane under TMSOTf catalysis at 85 °C (conventional heating) led to the C-glycoside **1240** with complete α-selectivity (Scheme 181).^295^

## 11 Conclusion

There is a large body of synthetic work for the synthesis of polyfluorinated carbohydrates, with most of the positional combinations for dideoxy difluorination of pentoses and hexoses exemplified and, at least for glucose, many of the trideoxy trifluorination combinations. Dideoxy difluorination methodologies at positions 1 and 2 of pentoses and hexoses (positions 2 and 3 for sialic acids) have been extensively investigated given their applications. Applications in nucleoside chemistry have led to a large body of work toward 2′,3′-dideoxy difluorinated pentoses.

By and large, the fluorination methodologies used in polyfluorosugar synthesis are also used in the synthesis of monodeoxyfluorinated sugars. The opening of epoxides, DAST-mediated deoxyfluorination, and reaction of glycals with SelectFluor are the most common methods, and the use of 1,6-anhydrosugars has proven particularly useful for controlled fluorine introduction at positions 2–4, despite the possible rearrangements, not least because of the possibility for deoxyfluorination at the 3-position with retention of configuration.

Many older syntheses described above, dating from the pre-DAST/selectFluor era, will be easily further optimized, and in this regard further synthetic advances will undoubtedly be possible with more recently developed fluorination agents and methodologies, as well as by considering the updated Richardson-Hough rules with the use of triflate leaving groups.^119,120^

The glycosidation of polyfluorinated sugars is an area where further advances are sorely needed to exploit their full potential as bioactive compounds or carbohydrate materials, both regarding glycoside formation with other sugars as well as with aglycons, including biomolecules. The modification of glycosyl donor reactivities by polyfluorination is naturally even more pronounced than with monofluorinated sugars, and perhaps the development of other methodologies than the traditional acid-catalyzed/electrophile-induced or base-mediated anomeric glycosidation will provide extra opportunities. A recent example by the Gilmour group allowing glycosidation of a 2,2-difluorinated reducing sugar appears very promising.^521^ Establishing efficient protocols to employ polyfluorinated sugar donors in an automated glycan synthesis setting will be another key advancement. It is worth pointing out that enzymatic glycosyl formation with polyfluorinated donors has not yet been achieved.^522^ Much work also remains to be done regarding establishing reactivities of hydroxyl groups in fluorinated sugar acceptors, both in chemical and enzymatic glycosylations.

Despite the large body of work involving 1,2- and 1,5-difluorinated carbohydrates, 2,3-difluorinated sialic acids, and with 2′,3′-diflluorinated nucleosides aside, there is still relatively little work to date on the investigation of biological activities of polyfluorinated carbohydrates, especially as part of glycans and multivalent constructs. This is largely due to the lack of efficient glycoside formation methodologies, and hence there are many opportunities for further development in this area.

Finally, the past few years have seen interesting results regarding how fluorination, including polyfluorination, influences key pharmaceutically relevant properties, such as lipophilicity. Clearly this is an area with great future perspectives, especially as new glycosyl formation methodologies become available. The first lipophilicities of disaccharides have only recently been reported by the Karban group.^523^ It will also be of interest to explore whether glycan conformation^524^ will be significantly influenced by polyfluorination.

In summary, the synthesis of polyfluorinated carbohydrates has reached an advanced state, with the synthetic frontier now being their efficient conversion into glycosides. Achieving this will unlock their potential in chemical biology, and medicinal and materials chemistry.
